# Proteomic analysis of exudate of *Cercospora armoraciae* from *Armoracia rusticana*

**DOI:** 10.7717/peerj.9592

**Published:** 2020-08-06

**Authors:** Haining Wang, Songhong Wei, Xiaohe Yang, Wei Liu, Lijun Zhu

**Affiliations:** 1 Department of Plant Protection, Shenyang Agricultural University, Shenyang, Liaoning, China; 2 Jiamusi Branch of Heilongjiang Academy of Agricultural Sciences, Jiamusi, Heilongjiang, China

**Keywords:** Proteome, Exudate, *Cercospora armoraciae*

## Abstract

**Background:**

*Cercospora armoraciae* causes leaf spot disease on *Armoracia rusticana*. Exudation of droplets, when grown on PDA, distinguishes this fungi from other members of the genus *Cercospora*. The role this exudate plays in the virulence of this pathogen has not been elucidated. To explore this, we characterized the transcriptome of *C. armoraciae* and the proteome of exudate associated with this plant pathogen.

**Methods:**

Virulence of three strains of *C. armoraciae* was evaluated in greenhouse assays. De novo sequencing was applied to assemble transcriptome from these strains. Nano-HPLC-MS/MS analysis was used to identify proteins in the pathogen exudate. Identified proteins were functionally classified and annotated using GO, KEGG, and COG/KOG bioinformatics analysis methods.

**Results:**

When treated with the exudate of *C*. *armoraciae* strain SCa-01, leaves of *A*. *rusticana* showed yellowing and necrosis of the leaves and similar symptoms to plants inoculated with this fungi. A total of 14,937 unigenes were assembled from *C. armoraciae*, and 576 proteins comprising 1,538 peptides, 1,524 unique peptide, were identified from the exudate. GO annotation classified 411 proteins (71%) into 27 functional categories, namely, 12, seven and eight biological process, cellular component, and molecular function subcategories, respectively. KEGG analysis assigned 314 proteins to 84 signaling/metabolic pathways, and 450 proteins were annotated against the COG/KOG database.

**Discussion:**

Transcriptome and GO analysis of *C*. *armoraciae* found most proteins in the exudate. GO analysis suggested that a considerable proportion of proteins were involved in cellular process and metabolic process, which suggests exudates maintain the metabolic balance of this fungi. Some proteins annotated to the phenylalanine metabolism, which suggests that the exudates may enhance the virulence of this pathogen. Some proteins annotated to the phenylalanine metabolism, which suggests that the exudates may enhance the pathogenicity of the pathogen. Also some proteins were annotated to the peroxisome metabolic pathway and the fatty acid biosynthesis pathways. These pathways may confer antifungal, antioxidant and antimicrobial activity on the exudates.

## Introduction

*Armoracia rusticana* Gaertn., Mey. & Scherb. (Brassicaceae), horseradish, is a perennial vegetable well known for its spicy roots (Kroener & Buettner, 2017). In Europe, the plant is also used locally for medical purposes, including treatment of edema, toothache, stomach, gout and rheumatism, and to promote perspiration (Bladh & Olsson, 2011). *Armoracia rusticana* is native to Eastern Europe but can now be found worldwide (Papp et al., 2018).

Cercosporoid fungi, one of the largest groups of hyphomycetes, are plant pathogens responsible for numerous economically devastating plant diseases. Although distributed worldwide, these fungi are especially abundant and diverse in tropical and subtropical areas (Guo, 2016). More than 5,000 names have been published (Groenewald et al., 2013). *Cercospora armoraciae* causes leaf spot disease on *A*. *rusticana*. The disease initially manifests as scattered, elliptical or irregular, taupe lesions surrounded by brown margins that eventually coalesce and rupture. As the disease progresses, almost 90% of leaves become infected, which threatens production.

Droplets from *C*. *armoraciae*, when grown on PDA, distinguishes this fungus from other members of the genus *Cercospora*. Many fungi produce exudates (Liang, Strelkov & Kav, 2010; Aliferis & Jabaji, 2010; Wang et al., 2017a), which contain diverse biomolecules (Cooke, 1969; Jones, 1970; Colotelo, 1971). These components relate to various functions of pathogens. For example, carbohydrates in the droplets may contribute to long-term survival of sclerotia (Daly, Knoche & Wiese, 1967; Willetts, 1971; Chet & Henis, 1975; Willetts & Bullock, 1992), while phenolic acids exuded by *Rhizoctonia solani* influence its antifungal, phytotoxic, and antioxidant activities (Aliferis & Jabaji, 2010). An understanding of exudate compositions should thus help elucidate pathogen functions and modes of action.

Transcriptome analysis is a rapid, accurate, cost-effective method for study non-model organisms (Qiao et al., 2013). To identify the genes expressed during exudation and the particular genes that correspond to proteins which found in the exudate, we constructed a library of transcripts. We then used the nano-HPLC-MS/MS analysis method to evaluate protein components in the exudate. A total of 14,937 unigenes were assembled and 576 proteins were identified from the exudate from *C*. *armoraciae*.

## Materials and Methods

### Fungal materials

*Cercospora armoraciae* strains (SCa-01, SCa-02 and SCa-03) used in this study were isolated from infected *A*. *rusticana* in ShenYang Botanical Garden, Liaoning Province, China, and maintained and subcultured on PDA plates at 25 ± 1 °C. To harvest pure mycelia of *C*. *armoraciae*, a piece of cellophane was placed under the mycelial plugs before transfer. The samples were stored at −80 °C in a 1.5 mL DNase/RNase free microcentrifuge tube (Sangon Biotech, Shanghai, China). After 20 days, mixed exudate was collected from hyphae using a capillary tube (20 µL), and the liquid was stored at −20 °C in a 1.5 mL microcentrifuge tubes (GEB, Torrance, CA, USA).

### Pathogenicity assays

Pathogenicity assays were performed on strain SCa-01 and its exudate. The fleshy roots of *A*. *rusticana* were grown under greenhouse conditions (temperature, 25–28 °C; relative humidity 75%; and natural daylight) in August. After 1-month cultivation, the same growth vigour of second youngest leaf was selected for inoculation with colonized PDA blocks (diameter, 5 mm) and a cotton ball moistened with 100 μL exudates on the adaxial surface of wounded leaves. All treatments were covered with parafilm to maintain moisture. Five replicates were used. Both treatments were placed at high relative humidity (~95%) for 24 h and then transferred the greenhouse. After 5 d and 10 d, the size of the lesions within each leaf piece was observed.

### Library construction and sequencing

Total RNA was extracted using Trizol reagent kit (Invitrogen, Carlsbad, CA, USA) according to the manufacturer’s protocol. RNA quality was assessed on an Agilent 2100 Bioanalyzer (Agilent Technologies, Palo Alto, CA, USA). RNA samples were then purified using Dynabeads^®^ Oligo (dT) 25 (Life, Carlsbad, CA, USA). Then the enriched fragments of mRNA were transformed into short fragments by fragmentation buffer, and the fragments were retranscribed into cDNAs by random primers (Grabherr et al., 2011). DNA polymerase I, RNase H, dNTP and buffer were used to synthesize the second strand of DNA. Then the fragment was purified with 1.8× Agencourt AMPure XP beads, repaired, poly(A) tail added, and connected to the Illumina sequencing connector (Illumina Hi Seq 4000 system) (Zhu et al., 2018).

### Raw read filtering

Reads were further filtered through fastp (version 0.18.0). The parameters were as follows: (1) reads with linkers; (2) readings with more than 10% unknown (“N”) nucleotides; (3) low-quality reads with >40% bases and a *Q* value less than 20.

### De novo assembly

Transcriptome de novo assembly was synthesized using the short reads assembly program-Trinity (Version 2.1.1) (Grabherr et al., 2011). The Trinity package contains three modules: Inchworm, Chrysalis and Butterfly. Inchworm uses a greedy k-aggregate-based approach to assemble readings to generate sets of linear overlapping groups. Chrysalis aggregates the corresponding overlapping groups, which correspond to the parts of alternatively spliced transcripts or other unique parts of the byline homologous genes, and then constructs the de Bruijn map of each related overlapping group. Butterfly analyses the paths between read and read pairs in the context of the corresponding de Bruijn graph, and outputs a linear sequence of homologous transcripts for each variable splicing and from the side-line homologous genes.

### Protein extraction

Total protein was extracted from the exudate with the cold acetone method (Liang, Strelkov & Kav, 2010). The resulting powder was dissolved in 2 mL pyrolysis buffer (8 M urea, 2% SDS), 1 protease inhibitor mixture (Roche, Basel, Switzerland). The mixture was treated by ultrasound on ice for 30 min and centrifuged at 13,000×*g* for 30 min at 4 °C. The supernatant was transferred to a new tube. For each sample, the protein was precipitated overnight with cold acetone at −20 °C. The precipitate was washed three times with acetone and dissolved in 8 M urea by ultrasonic treatment on ice. Protein quality was examined by sodium dodecyl sulfate polyacrylamide gel electrophoresis.

### Protein digestion

The protein concentration in supernatant was determined by BCA protein assay kit (Pierce, Rockford, IL, USA). The 100 μg protein was transferred to the new tube, and the final volume was adjusted to 100 μL with 8 M urea. In the protein solution, 11 μL of 1 M DTT (DL-dithiothreotol) was added and kept at 37 °C for 1 h, then transferred to 10 K ultrafiltration tube (Merck Millipore, Billerica, MA, USA). To remove urea, the sample was centrifuged three times by adding 100 Mm TEAB. A total of 120 μL 55 mM iodoacetamide was added to the sample, kept in room temperature for 20 min and protected from light. Then the proteins were digested overnight with sequence-level modified tryptase (Promega, Madison, WI, USA).

### nano-HPLC-MS/MS analysis

The sample was divided into three equal parts and resuspended with 30 μL solvent C (C: 0.1% formic acid water; D: ACN containing 0.1% formic acid). The sample solution was separated by nanoLC and analyzed by on-line electrospray tandem mass spectrometry. The experiment was carried out on an Easy-nLC 1000 system (Thermo Fisher Scientific, Waltham, MA, USA), which is connected to the Q-Exactive mass spectrometer (Thermo Fisher Scientific, Waltham, MA, USA) equipped with an online nano-electrospray ion source. The peptide sample (10 μL) was loaded onto a trap column (Thermo Scientific Acclaim Pep Map C18,100 um × 2 cm) at a flow rate of 10 μL/min for 3 min, then followed by a linear gradient from 3% D to 32% D in 120 min to separate with the analytical column (Acclaim PepMap C18, 75 μm × 15 cm). The column was re-equilibrated for 10 min with a flow rate of 300 nL/min under initial conditions. The electrospray voltage of the inlet of the mass spectrometer was 2 kV. Mass spectrometer operated in data-related acquisition mode to automatically switch between MS and MS/MS acquisition. Full scan MS spectrum (m/z 350–1,550) with mass resolution of 35 K was obtained. Then continuous high energy collision dissociation MS/MS scanning was performed with resolution of 17.5 K. The dynamic exclusion time was set to 20 s. Data are available via ProteomeXchange with identifier PXD019663.

### Database search

Proteome Discoverer 1.2 (Thermo Fisher Science, Waltham, MA, USA) was used to convert mass spectrometry data into MGF files. The Mascot search engine (Version 2.3.2; Matrix Science, London, UK) was used to identify proteins by comparison to the reference transcriptome (accession number: SRR9163995). We searched the Mascot database for protein identification by using the *C*. *armoraciae* reference transcriptome in NCBI nr/Swiss Prot/Uniprot/IPI databases. The search parameters of Mascot were set to 0.050 Da for fragment ion mass tolerance and 10.0 ppm for parent ion tolerance.

### Protein functional annotation and enrichment analysis

GO (http://www.geneontology.org/), KEGG (http://www.genome.jp/kegg/pathway.html), and COG/KOG (https://www.ncbi.nlm.nih.gov/COG/) databases were used to annotate the protein functions and pathway.

## Results

### Pathogenicity tests

**Figure 1:**
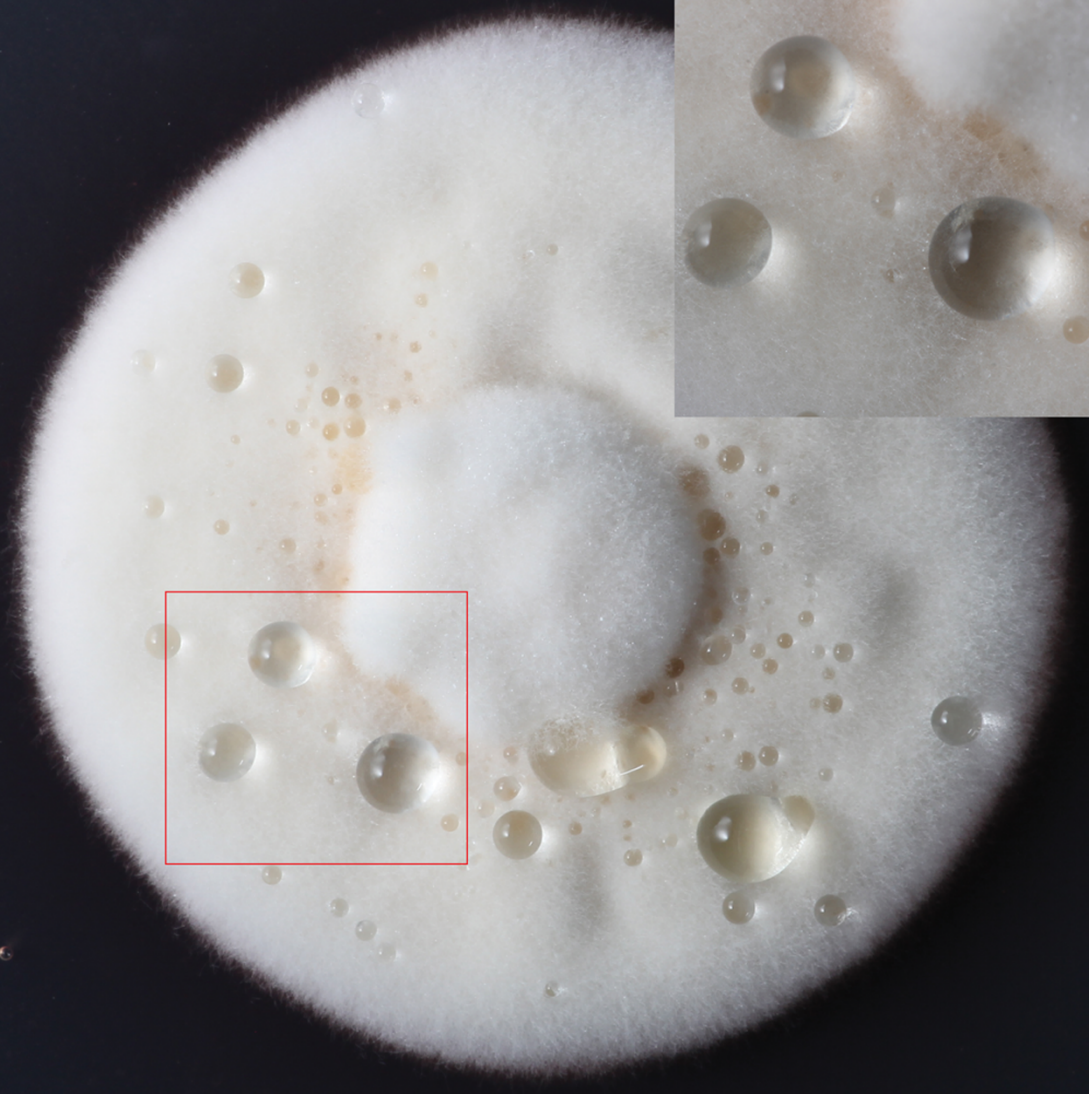
The formation of exudate droplets *C. armoraciae* accompanying with mycelial growth.

**Figure 2:**
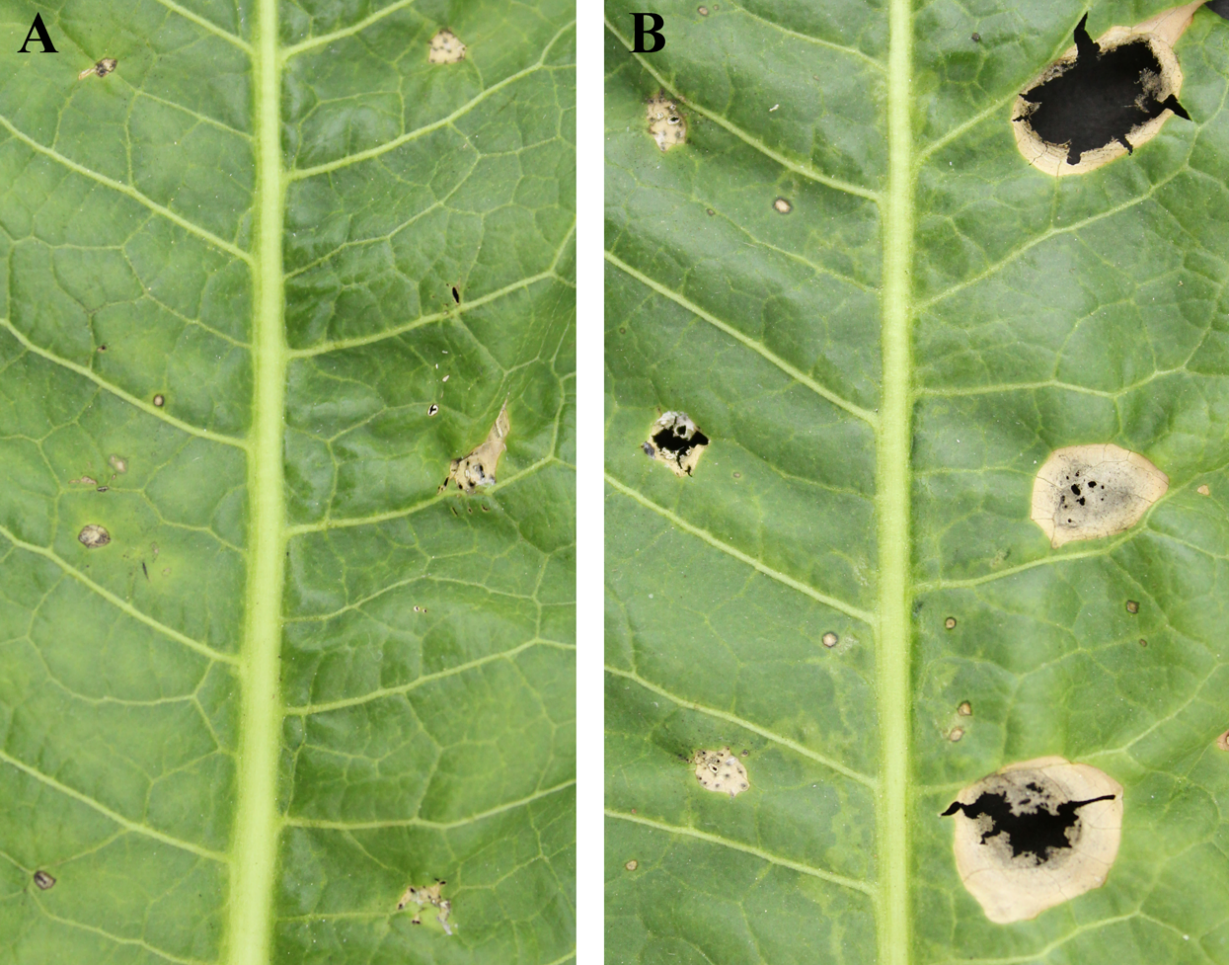
Pathogenicity tests results depicting different leaf spot lesions on *A*. *rusticana* with pathogen inoculated and exudate inoculated respectively, after 5 d (A) and 10 d (B). Left side of leaf: exudate inoculated site. Right side of leaf: pathogen inoculated site.

Exudation from *C*. *armariaciae* appeared to damage horseradish plants in potted plant trials (Fig. 1). At 5 d-inoculation, yellowing and necrosis of the leaves was observed, and exudate infected site also showed pitchy edges, but there was no obvious size difference between pathogen and exudate inoculation site (Fig. 2A). At 10 d-inoculation, there were significant differences in necrotic spots caused by pathogens and their exduate. The pathogen inoculated lesions coalesced with a taupe brown center, which showed the similar symptom in the field. Exudate inoculated site of leaves did not expand significantly (Fig. 2B).

### Illumina sequencing and de novo assembly of the *C*. *armoraciae* transcriptome

By Illumina sequencing, there were 51,659,554 raw reads obtained before filtering. Of them, 50,648,514 clean reads were combined and used for transcript assembly after filtering low-quality reads, resulting in 14,937 unigenes. The length of all transcripts ranged from 201 to 23,020 nucleotides, with a N50 length of 2,441 nucleotides. The raw data of RNA-seq were available at the national center for biotechnology information sequence read archive (SRA): accession number SRR9163995.

### Protein identification

There were 576 proteins identified in the exudate including 1,538 peptides, of which 1,524 were unique (Table S1).

### GO classification

**Figure 3:**
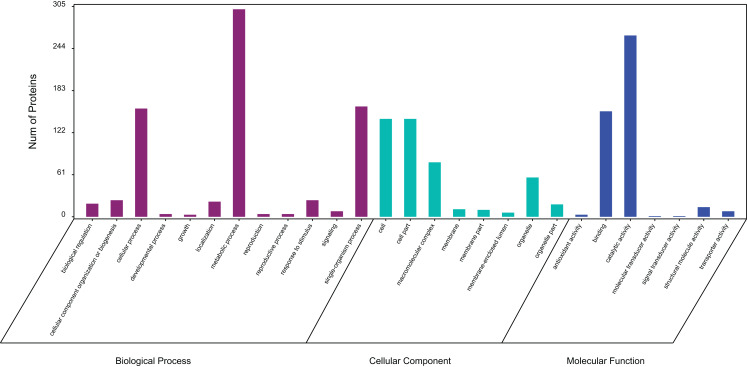
Gene ontology (GO) classification of exudate components from *Cercospora armoraciae*. Biological process (green bars), cellular component (red bars), and molecular function (blue bars).

In a GO analysis, 411 proteins (71%) were assigned to 27 GO functional groups. Among the 27 functional groups, the GO subcategories could be classified into 12 biological processes, 7 cellular components, and 8 molecular functions (Fig. 3). Metabolic process (301), cellular process (157) and single biological process (160) occupied the first three terms in the category of biological processes. While the cell (142) and cell part (142) were equally the most abundant terms in the cellular component category. Among the molecular function category, catalytic activity (263) and binding (153) accounted for an important proportion.

### KEGG pathway analysis

Exudate proteins from *C*. *armoraciae* were assigned to biochemical pathways labeled with the KO number of the homologous/similar protein. Among them, 314 proteins were categorized into 84 signaling/metabolic pathways (Table 1). The top five most highly represented pathways were protein processing in ribosome (72 members, path: ko03010), carbon metabolism (53 members, path: ko01200), biosynthesis of amino acids (34 members, path: ko01230), starch and sucrose metabolism (26 members, path: ko00500), and glycolysis/gluconeogenesis (19 members, path: ko00010). Sixteen pathways were associated with amino acid synthesis (path: ko01230, ko00290, ko00220, ko00400, ko00300), metabolism (path: ko00270, ko00330, ko00350, ko00380, ko00260, ko00460, ko00250, ko00360, ko00340, ko00410), and degradation (path: ko00310, ko00280). In addition, 15 pathways were related to carbohydrate metabolism: starch and sucrose metabolism; glycolysis/gluconeogenesis; pyruvate metabolism; glyoxylate and dicarboxylate metabolism; citrate cycle; galactose metabolism; amino sugar and nucleotide sugar metabolism; fructose and mannose metabolism; pentose phosphate pathway; pentose and glucuronate interconversions; inositol phosphate metabolism; ascorbate and aldarate metabolism; C5-branched dibasic acid metabolism; propanoate metabolism; and butanoate metabolism. Energy metabolism and lipid metabolism pathways also accounted for a large proportion of the annotated pathways.

**Table 1:** Pathway enrichment of exudates from *Cercospora armoraciae*.

No.	Pathway	All genes with pathway annotation (314)	Pathway ID
1	Ribosome	72 (22.93%)	ko03010
2	Carbon metabolism	53 (16.88%)	ko01200
3	Biosynthesis of amino acids	34 (10.83%)	ko01230
4	Starch and sucrose metabolism	26 (8.28%)	ko00500
5	Glycolysis/Gluconeogenesis	19 (6.05%)	ko00010
6	Pyruvate metabolism	17 (5.41%)	ko00620
7	Glyoxylate and dicarboxylate metabolism	16 (5.1%)	ko00630
8	2-Oxocarboxylic acid metabolism	16 (5.1%)	ko01210
9	Citrate cycle (TCA cycle)	14 (4.46%)	ko00020
10	Cysteine and methionine metabolism	14 (4.46%)	ko00270
11	Arginine and proline metabolism	14 (4.46%)	ko00330
12	Tyrosine metabolism	14 (4.46%)	ko00350
13	Methane metabolism	14 (4.46%)	ko00680
14	Galactose metabolism	13 (4.14%)	ko00052
15	Tryptophan metabolism	12 (3.82%)	ko00380
16	Glycine, serine and threonine metabolism	11 (3.5%)	ko00260
17	Cyanoamino acid metabolism	11 (3.5%)	ko00460
18	Amino sugar and nucleotide sugar metabolism	11 (3.5%)	ko00520
19	Protein processing in endoplasmic reticulum	11 (3.5%)	ko04141
20	Fructose and mannose metabolism	10 (3.18%)	ko00051
21	Alanine, aspartate and glutamate metabolism	10 (3.18%)	ko00250
22	Phenylalanine metabolism	10 (3.18%)	ko00360
23	Peroxisome	10 (3.18%)	ko04146
24	Valine, leucine and isoleucine biosynthesis	9 (2.87%)	ko00290
25	Proteasome	9 (2.87%)	ko03050
26	Pentose phosphate pathway	8 (2.55%)	ko00030
27	Oxidative phosphorylation	8 (2.55%)	ko00190
28	Arginine biosynthesis	8 (2.55%)	ko00220
29	Purine metabolism	8 (2.55%)	ko00230
30	Lysine degradation	7 (2.23%)	ko00310
31	Glutathione metabolism	7 (2.23%)	ko00480
32	Glycerolipid metabolism	7 (2.23%)	ko00561
33	Phagosome	7 (2.23%)	ko04145
34	Pentose and glucuronate interconversions	6 (1.91%)	ko00040
35	Histidine metabolism	6 (1.91%)	ko00340
36	Glycerophospholipid metabolism	6 (1.91%)	ko00564
37	Nitrogen metabolism	6 (1.91%)	ko00910
38	Fatty acid degradation	5 (1.59%)	ko00071
39	Valine, leucine and isoleucine degradation	5 (1.59%)	ko00280
40	Beta-Alanine metabolism	5 (1.59%)	ko00410
41	Other glycan degradation	5 (1.59%)	ko00511
42	Thiamine metabolism	5 (1.59%)	ko00730
43	Riboflavin metabolism	5 (1.59%)	ko00740
44	RNA transport	5 (1.59%)	ko03013
45	Endocytosis	5 (1.59%)	ko04144
46	Inositol phosphate metabolism	4 (1.27%)	ko00562
47	Glycosphingolipid biosynthesis—globo series	4 (1.27%)	ko00603
48	Pantothenate and CoA biosynthesis	4 (1.27%)	ko00770
49	Sulfur metabolism	4 (1.27%)	ko00920
50	Phosphatidylinositol signaling system	4 (1.27%)	ko04070
51	Ubiquitin mediated proteolysis	4 (1.27%)	ko04120
52	Ascorbate and aldarate metabolism	3 (0.96%)	ko00053
53	Pyrimidine metabolism	3 (0.96%)	ko00240
54	Phenylalanine, tyrosine and tryptophan biosynthesis	3 (0.96%)	ko00400
55	N-Glycan biosynthesis	3 (0.96%)	ko00510
56	C5-Branched dibasic acid metabolism	3 (0.96%)	ko00660
57	Nicotinate and nicotinamide metabolism	3 (0.96%)	ko00760
58	Aminoacyl-tRNA biosynthesis	3 (0.96%)	ko00970
59	RNA degradation	3 (0.96%)	ko03018
60	Taurine and hypotaurine metabolism	2 (0.64%)	ko00430
61	Selenocompound metabolism	2 (0.64%)	ko00450
62	Glycosaminoglycan degradation	2 (0.64%)	ko00531
63	Sphingolipid metabolism	2 (0.64%)	ko00600
64	Propanoate metabolism	2 (0.64%)	ko00640
65	Terpenoid backbone biosynthesis	2 (0.64%)	ko00900
66	Degradation of aromatic compounds	2 (0.64%)	ko01220
67	mRNA surveillance pathway	2 (0.64%)	ko03015
68	Spliceosome	2 (0.64%)	ko03040
69	Protein export	2 (0.64%)	ko03060
70	Cell cycle—yeast	2 (0.64%)	ko04111
71	SNARE interactions in vesicular transport	2 (0.64%)	ko04130
72	Regulation of mitophagy—yeast	2 (0.64%)	ko04139
73	Fatty acid biosynthesis	1 (0.32%)	ko00061
74	Aflatoxin biosynthesis	1 (0.32%)	ko00254
75	Monobactam biosynthesis	1 (0.32%)	ko00261
76	Lysine biosynthesis	1 (0.32%)	ko00300
77	Various types of N-glycan biosynthesis	1 (0.32%)	ko00513
78	Arachidonic acid metabolism	1 (0.32%)	ko00590
79	Butanoate metabolism	1 (0.32%)	ko00650
80	One carbon pool by folate	1 (0.32%)	ko00670
81	Porphyrin and chlorophyll metabolism	1 (0.32%)	ko00860
82	Fatty acid metabolism	1 (0.32%)	ko01212
83	Ribosome biogenesis in eukaryotes	1 (0.32%)	ko03008
84	Sulfur relay system	1 (0.32%)	ko04122

### COG/KOG functional annotation

**Figure 4:**
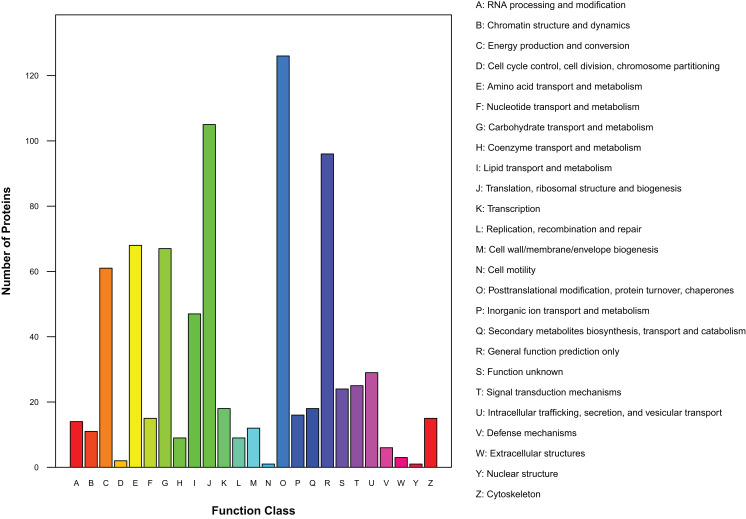
Distribution of COG/KOG functional classes of exudate proteins from *Cercospora armoraciae*.

There were 450 proteins annotated against the COG/KOG database (Fig. 4). In all functional ontologies, O group (posttranslational modification, protein turnover, chaperones) had the most protein. J (translation, ribosomal structure, and biogenesis) and R (general function prediction only) groups also contained relatively more proteins. Y (nuclear structure) and N (cell motility) were two functional groups with less protein. In addition, 24 proteins were categorized as functionally unknown proteins.

## Discussion

We performed de novo sequencing to profile the transcriptome and nano-HPLC-MS/MS analysis to study the proteomics of *C*. *armoraciae* exudate. This approach provided preliminary information needed for molecular profiling and functional studies of this, and related, plant pathogens that traditional methods of protein research cannot provide (Zhang et al., 2013; Lin et al., 2017; Wang et al., 2017b). Searching and annotating exudate proteins in the transcript database identified 576 proteins; however, lack of a reference genome limits the effective information obtained from only a single identification of a protein of its exudate.

Although many hyphomycetes exudate droplets under artificial culture conditions (Sprecher, 1959; Colotelo, 1978; Stefan et al., 2010), the role of exudates in the ecosystem is unclear. We speculate that exudation of droplets accompanies the entire process of colony growth and development and plays a vital role in plant–fungal interactions and maintenance of the physiological balance of fungi (Cooke, 1969). Guttation droplets of *Metarhizium anisopliae* serve as a water reservoir for maintaining the constant growth of aerial hyphae (Stefan et al., 2010). Exudate decreases during development of Sclerotinia (Liang, Strelkov & Kav, 2010; Wang et al., 2017a), which may reflect partial re-absorption of exudate into the fungus (Colotelo, 1978). In the case of *Sclerotinia* and *Sclerotiorum* species, excess nutrients, water, and metabolites of exudates translocate to mature sclerotia to sustain increased metabolic activity (Georgiou et al., 2006; Cooke, 1971; Punja, 1985).

As inferred from the GO analysis, some proteins were related to growth, development, and reproduction, which suggests exudates participate in the whole life history of the pathogen. Cell and cell part are the two most important cell component GO terms, and many proteins are involved in the formation of organelles and membranes. KEGG analysis indicated that 16 of 84 identified pathways were related to amino acid synthesis, metabolism, and decomposition; among these pathways, phenylalanine metabolism is associated with the shikimic acid pathway and enhances pathogenicity (Southerton & Deverall, 1990). KEGG analysis also identified 15 pathways related to carbohydrate metabolism, including 3 (glycolysis/gluconeogenesis, citrate cycle, and pentose phosphate) that cooperate to increase the adaptability of organisms (Kruger & Schaewen, 2003). Two identified pathways, the peroxisome metabolic pathway and the fatty acid biosynthesis pathway, may confer antifungal, antimicrobial, and antioxidant activities on exudates and protect the pathogens. Exudates of *R*. *solani* significantly reduce the spore germination of *Stachybotrys elegans*, *Heterosporium solani*, *Fusarium sporotrichioides*, and *Trichoderma virens* relative to the control (Aliferis & Jabaji, 2010), and exudates from *Sclerotium rolfsii* and *R*. *solani* also contribute to antioxidant activities (Pandey et al., 2007; Aliferis & Jabaji, 2010). COG/KOG analysis uncovered 24 proteins of unknown function, such proteins also account for 32% and 23% of exudate proteins of *Sclerotinia sclerotiorum* and *S*. *ginseng*, respectively (Liang, Strelkov & Kav, 2010; Wang et al., 2017a).

## Conclusions

This first-ever detailed classification and annotation of proteins of exudate from *C*. *armoraciae* supports the hypothesis that exudates enhance virulence.

## Supplemental Information

10.7717/peerj.9592/supp-1 Supplemental Information 1 De novo assembly sequences of *Cercospora armoraciae*.

10.7717/peerj.9592/supp-2 Supplemental Information 2 List of proteins identified with LC-MS/MS in the exudates of *Cercospora armoraciae*.

## Additional Information and Declarations

### Data Availability

The following information was supplied regarding data availability: The raw data of RNA-seq are available at the National Center for Biotechnology Information Sequence Read Archive (SRA): SRR9163995. The mass spec data are available in ProteomeXchange: PXD019663.
