# Supplementary material for: Proteomic analysis of exudate of Cercospora armoraciae from Armoracia rusticana
Source: PeerJ. 2020 Aug 6;8:e9592. doi: 10.7717/peerj.9592 (PMC7512140; doi:10.7717/peerj.9592)
Supplement: Supplemental Information 2 [file peerj-08-9592-s002.docx]

**Supplementary Table S1 List of proteins identified with LC-MS/MS in the exudates of *Cercospora armoraciae***

| **GroupID^a^** | **Accession^b^** | **Score^c^** | **Mass^d^** | **Cov^e^** | **Seq^f^** |
| --- | --- | --- | --- | --- | --- |
| 1 | Unigene0001807 | 5235 | 45302 | 37.8 | MKFSLAGTAFAALVSFTAALPHQKDDITSLATEQAVQANYDVVIVGGGPAGLSAASALARVRRSVLLIDSADYRNQYTRHVHDVLGYDGVTAAYLRWRAREQIIQYPTVNLTNGTVSKIESTSNENVTSFCITANFSSNGNTTFTARKVVLATGLRDIIPTSPGFAENWGKGIYWCPWCDGYEHADQPYGILGPLDKSGNTPLEILTLHKDVIIFANGTDTPENRAAVQKKLPDFDIWYDVHGIKVDNRTITSLERLQDASAQYHDPTKPTSPEYDLFRLNFVEGPSVERAAFITNWDIEQRSKIGQEAGVQIWGDQLATNASNGMITNVPGIYGIGDANADNSTNVPHAMWSGKRAAVSIHVKLETENAQYQISQAKAKGLTKRELSERDVWDIVNPRDDALYVEDFDR |
| 2 | Unigene0007268 | 4208 | 14909 | 50 | LPQNAGNSAGSNMCDASTFNNGADLGVPQANTQDCTDLVEGLDKGRNWSVSKSWARLSTQGTCAFSVRVVAGSDNGLVGGADLADLVNDSINKFQQSGQVSCKGQFGQVVSAEGEVDCNARSGGSQVRVEWVIAASSFNPSN |
| 3 | Unigene0010666 | 3995 | 17093 | 70.7 | MQFSTALTALAGLLAYSSIAAASPLAEPQNAGSGNSPSNRCNTRAVTGNQVVKGTAPTWNDCYALADKFQNEDFFRKSIKVEGTYKQTYGTCGFSITAKAGNFQLGKDDAADLIRSTAGDLGAKSGAYQTGLVPCDTPGTAGGDRGRQTGWTIGNLAAPGPKQG |
| 4 | Unigene0006111 | 3251 | 70687 | 55.8 | LATLGTAFPRVHGDSHVHRRQAGNDTDYEYVIVGSGPGGGPLAARLAIAGHRVLLIEAGGDFGDNVNQTVPARNLQSAEDPNIKWDYFVTHYKDVERQKKDIKMAYRLSDGSFYHGLYPPEGAEPLGILYPRASALGGCAEHNAVVTTYPHKSDWTYLQTLTGDDSWAPEKMREYFKKLEHAQYPVGPGHGKDGWFHTSLTELTLVAADFKVLSVVLSAASALGKSDLISTVLNTVTGLANVLLTDLNADTPDRDQQEDLFQIPQGIDAGSSTRSTPRKLILDTANAVNEDGSRKYVLDIKLNTLATKVRFDNSTKPRAIGVEYLEGKQLYGASANPTSGGRPGYVAAAKEVIVAGGTFNTPQLLKLSGVGPREELEGFGIPVVHDLPGVGTNMQDRYEIAIVGQAETKLSISKDCTFGYDSTDPCLDRWQEGNSAFARGPYATGGVAVGVTKKSSGASEDEDPDLFIIGAPGLFGGYYPGFAYDAVRPGNIWSWLALKAHSHNNAGTVTLRSADPHDVPQIDFNSFDTGNTTDGGDEKDLQAATEGLMFGREAFDKLIPLDGTFTEIQPGRNVTGEDGLKEWARDNAWGHHACCSAPIGADGDPMAVLDTNFRVRGVDGLRVVDASAFPKIPGTFIALPIYMISEKAADVIINGN |
| 5 | Unigene0014417 | 3089 | 20556 | 48.5 | PTEMLEARQAVGDSASELENGACRPLILIFARGSTQPGNLGDIGSATCNQLKTAYGSTGVACQGVGGAYSASLAANILPEGTTSGSWNEAIRLFNLARSKCPNSKVVAGGYSQGAAVITASVRRLPAASKTNLAGIVMFGSTRTDEEGGRIPGYPAANTLNICASNDGVCDGGLDVSPGHSAYADDVPTAVSFLRGRVTA |
| 6 | Unigene0002688 | 2984 | 63860 | 31.2 | MPSTRSLIFGLGAIASSYAQQQTEYDYIICGGGTAGLAVANKLSEANNTVLIVEAGVNGSTSSWSYRSTPQTYAKDRVLDLPAGRTVGGSSQINGKVYSRPNAESIDDWGRVNNGDWSWNTLLPFYKSSETLDVPSEDLADAGYTYNPDYHGTSGPLNVSFPAQGTRAYWELLKNASAAFDISVNRDFNGGKAEGLATYPAAFTINGEREQTRESAREAYYLPIVSRTNLELLDETVCLRIVFADAQGENATLTATGVEIANTNNQTTITARKEVILAAGVYRSPGILEFSGVGNKALLANYSIETKVDLPGVGENLQDQLQGNIYYERVNSSNITFPSPVGDEITTPYLIHATYEQIFGDDAGDFQERTNSSLSEYANTISEGINGTLSADQILNSLRVQYEAIFSTLIPSVEIFSGQSLNSTHLNLEFWPLIPLGRGNVHISGGNPERAGDSPNVNVNWGILEFDWEMLTASARFVRNMFKTEAFAAEVQAETRPGFSNVSEDASDAEWKEYWQDNFRAGWHGVGTAAMLPREWGGVVNTNLTVYGTANVRVVDASVIPFQLGGHPTATVYALAERAASLIVNGTSG |
| 7 | Unigene0010700 | 2445 | 16527 | 64.3 | STLKPASTYHKYHQSLVKMKVIAIASLFAASVMAQQWGNREEWNRDSIRVSVYQGPGFWGEQRWDCDGNEEGCYKAQCTCNRFDWNTWQLVQDDVLGERACNDLRSTYPNLVWQMGLGCIDLDRRGIEPHGMARACERQS |
| 8 | Unigene0013488 | 1871 | 75332 | 47.9 | MTIPDEVDIIVCGGGSCGCVVAGRLANLDHKLQVLLIEAGESNLNNPWVFRPGIFPRNMKLDSKTATFYYSRPSEWLSGRRAIVPCAHILGGGSSINFMMYTRASASDYDDFQAKGWTTKELIPLMKKHETYQRACNNRDLHGFDGPIKVSFGNYTYPVMQDFLRAAESQGIPVTDDLQDLVTGHGAEHWLKWINRDTGRRSDSAHAYIHSTRQHYENLHLAVNTKVDKVVFEGTKAVGVRTIPTKPLHPSENHSRTFRARRQIIVSGGTLSSPLILQRSGIGDAQKLRKAGVKPLVDLPGVGQNFQDHYLFFSLYRAKPHVESFDDFVRGDKEVQEKVFNQWQLDGTGPLATNAIEAGVKWRPSQKDLDDMARSPFPEFIKGWDSYFKDKPDKPVMHWAVVAGWFGDHMHVPPGKYFSMFHFLEYPFSRGFTNIVSPNPYEAPDFDAGFMNDRRDMAPMVWGYIKSRETARRMDAFAGEVANNHPFYAYDSPARCHDMDLATTNAYAGPNHITSNLVTGAWTNPLPAPSRQPEPSFLNSNQQALHDDLQYSRDDLLAVEEWVKRHTETTWHSLGTCSMAPREGNSIVKHGVLDERLNVHGTKNLKVADLSICPDNVGCNTYSTALLIGEKCAVLTAEDLGYTGRALDMRVPDYQVNREIVGLSRL |
| 9 | Unigene0009474 | 1314 | 35950 | 36.3 | QETLSPLLTTIKNRPELSIFYSLVSSTGGTSGIPGPALEERFNDPNNSLRFTAFAPTNDAFSHISQDTIAALTTPQSYQLLYSILINHIAPGEPTFSDLQRQGTVHAIGGFDISFDAQGELLTNANTTSESERSRNHQATFIKGQDGQPIRIPASNGAIFMIDHVLDGLFTYFGVDEADSGNQGVPEAIEHTGSMRDILETTPELSTLSDILKGLRADFVTRLSLASSDLAVNNRTVFLAPSNTAFEALPSEAVQKALQPSNYDLSAFLLRFGLGEVAGADAETGRMKVKSQSGFVILVEPGRANNARVEKRICAHNGCVWVMGRWLDPLW |
| 10 | Unigene0009686 | 1290 | 28335 | 65.3 | MVRFTDLSVHALAACLMSSAQARVDHVANGLNARQADMAAAYSLPPLGYAYDALEPFFDKETMEIHYTKHHQTYVNNLNNLLKDNELSTLAVDDLVTRLAEVQDQIPSVNRTGVRNNAGGHSNHSFFWKQLKLNTTLEGDLKEAIDKNFGSFEEFQKQFEKAATSVFGSGWAWLVHQEDGTLKVVSTANQDSPLMGKQISGAEGTPIIGLDVWEHAYYLKYQNRRPDFIKAYLSVVNWEFAGQRYSEVKKC |
| 11 | Unigene0014388 | 1277 | 18919 | 64.9 | MRLITLLLGLPHLALGASLQARSLPDVDAGCNICHPGQPAYAKWEADVKAVPQDMIDEAKQSFSRSYPENFSPPLCGQRGVNCIHLEPGFVWERNPASLIQDPVGKYSMTDGRTTYVFLYWNHMQKFKSTSGSVNVYPAHCVTELVVNSGAKATVDGKSYLPGGGYKNSGK |
| 12 | Unigene0002555 | 980 | 81325 | 34.3 | CVVATLTLHPGCEAAAVSPHLIERQSDTLSSGIKRPPQALHVFNETIGSINKTVEDYAQWEKRWVDNLAKSLTVDNATFDNLALPWAQHEAEAALRIGYLTALLGFHPDKDLRDAVDAISTTLTNISQQTLTNEQLFKLFNTVYDKQLNDTKLDAESRLYLEKVPQDFNSSGLGIEAGPRRDRYLEISRRLQVLSNEFSAEVVNDNTTLWFTREELPGVKPDMLDALIKGDGPNQGKLGVLINDPSTDDVSSYCTNETTRKSIDIGSNHIAPSNIGRLEEALALRDEQARIVGQPDFATWALGDMVAKTPAAVQDLQQKVQDAMSPKLPKEMDTLKQMKNKTGGDASHLYTWDVGLYRRLVIEEVSALDLDKQKEYFPAAQTAHRILDLYAELFGIKFVNIGGKDLDKLSPTGNGTELTWHPDVEVFAVWDSKDANEFVGFLYLDIYFREGKGDGAFMVPLEPGFDVSSGKRNYPVAGLFTNFRKTDANATRPSLMSQDEVSTLMHEAGHGMHQLLSKTRFSRFHGVNAPLDWVEMPSQLMEEFAYAPEVLKRLSQHYTRIDPKYAEAWKKNNTELPPETLPDEAISDMVKTRVAFSARQQIFQITYGKIDQIYHTPKSQEDAKKINSTKIWNDELFAALKTEGRGINGGHGQTTFTHIMGGYQAKYYSYLWSRVYAIDVYYTAFKANPINPEAGRRWREEVLKVGGATDDFQGVLDKFLGHK |
| 13 | Unigene0013853 | 929 | 68407 | 29.5 | PGKKGTLMFNRLSPASSVLYIANSDGTNPRRLLSNSTAGFEYHATFSPDGKYIVYTSEKRGAGQADLYRVRVNGTGIEELVATDAVDDQGVLSPDGNLLAYVSSENGWKANIWVKDLRTGETRNLTNTPSVKGQSWSPDGYFKPAWSPDGQWIALSSDRNTGWYGHGDGAGWENTQELSIYAIRPDGTDFRLIATKPGYALGSPKWSQDGRRIVYYELERSHTYSVRSSPLWNDAANQIWSVDFETEETRQETETSYLKVSPQYVNDELGIGYLVKSHQDAAGVYYTNNQNKSFTFASLGLPINNPSWSPDGKQVVFEVLNLDPFNPDREQFPFDKDWIPKLGQQFPTTSPKGDKIAFPDPWRGSVVVANVSDLAHEDNVPLKNLTIPAAGPIRNLTNNLQAPLEVLQPAWSPDGEWIAFGAGAYFNQRRQNAAAIYRVRSDNSSLPERLTDLANNAGFPSYSHDGNSIVYRQWGPPGRAGYGLYAYDSAKNVTTRLTEGTMSDNFPSFSPDGSRIVFTRRMNVTNFDICTIKPDGTDLQVLTTSGANDAHATWTYDGRILYSSGASGWKAEAATYDQAEQPYGQLWIMDADGSNKYQIADSLWEDAMPIYLPGVYL |
| 14 | Unigene0013037 | 904 | 32150 | 41.9 | RGFNYGANGPSGPRAQGDFEAEFNRAKTLPGTAPFTSARLFTMIQANTVNDPSSAIPAAIATNTSLLLGLWASAGQDAFNQELEALRRALEQFGQPFIDLIDGISVGSEDLYRITPTGIANKAGIGASPDDLVKYIQQTRDKLNGHDNLKSKIGHVDTWTAWVIETNYPVTTACDWIGMDSYPYYQKEVPNSIDQAKELFFQSYNTTKNVSQGKKVLVTETGWPVQGPDFGLAVANVDNAKRYWDEVGCALFSEPVDTWWFTLDDSKQNPEEISFSVVKPGLGDPIWDLKC |
| 15 | Unigene0008507 | 783 | 9907 | 39.8 | LLAGLAAAQGAQKPNPYFGGCDHIPIGQDAPYYNCYWLTKGGKDDCEALVPNCMSPCFRAGSQDAKSGYQVVAENDGHNVCRCRCKLN |
| 16 | Unigene0005523 | 773 | 60275 | 35.1 | LVFGLASATQYGNNWVPVKYDPPQVEKNFQDPGIKLISPAFIKNDTIPPGFSDGTAGPTSQVILDVYIQALADRNPWIKYSPVEYESEEGRDFPYILLTNGQQPNFTYSPSGGNVTNPLWSGNVTADQSQGNGSGDKLRIWIQGGVHGNEPGGDQAVMALLGKMNANQTWTNQILQNADIMVLPRYNPDGVAYFQRTYASNFDPNRDHTKLARQQSRDIKQTFSDFAPHIAVDLHEYGANTLYSGSYVPGADAMFSAAKNLNIHAGIRNMSEEVFAPAIAAHLEKQGFRWEPYATGASDGDTRNSTIILQEAGSDAKIGRNSMGLSQAIVFLFETRGIGIADQEFARRTATGLALLEATINTAIANAAEIRQVVEESIEDFVQSKDKIVITDTPQNATVRNWTLIHYQTGELQQIPVAFFATTPTIANLTRARPEAYLIPKGWKDLIPRLEASGLEVETLDQAFRGPVEALTIETAGFNEPAYFEGAVLATVTTNSTTKNIELPAGSFLVSTRQKNAGLAFAALEPENIDSYVSFGVVPLAAGDEYPIYRVMA |
| 16 | Unigene0005522 | 773 | 60275 | 35.1 | LVFGLASATQYGNNWVPVKYDPPQVEKNFQDPGIKLISPAFIKNDTIPPGFSDGTAGPTSQVILDVYIQALADRNPWIKYSPVEYESEEGRDFPYILLTNGQQPNFTYSPSGGNVTNPLWSGNVTADQSQGNGSGDKLRIWIQGGVHGNEPGGDQAVMALLGKMNANQTWTNQILQNADIMVLPRYNPDGVAYFQRTYASNFDPNRDHTKLARQQSRDIKQTFSDFAPHIAVDLHEYGANTLYSGSYVPGADAMFSAAKNLNIHAGIRNMSEEVFAPAIAAHLEKQGFRWEPYATGASDGDTRNSTIILQEAGSDAKIGRNSMGLSQAIVFLFETRGIGIADQEFARRTATGLALLEATINTAIANAAEIRQVVEESIEDFVQSKDKIVITDTPQNATVRNWTLIHYQTGELQQIPVAFFATTPTIANLTRARPEAYLIPKGWKDLIPRLEASGLEVETLDQAFRGPVEALTIETAGFNEPAYFEGAVLATVTTNSTTKNIELPAGSFLVSTRQKNAGLAFAALEPENIDSYVSFGVVPLAAGDEYPIYRVMA |
| 17 | Unigene0002965 | 772 | 16009 | 37.8 | MQFTTTVFALLAAASASVATPVPAEAATNMMAAATPQWTIENFTRTCNGNDSNCKYSFGINTNDGQKVTGCGYSVNGKPASRATYQNIQCGAFRIGSTWSGQFGVGQGFQTLSVVKDRQIVYPAYTDKQLVNGKTVKPNQSYAPQNLP |
| 18 | Unigene0014042 | 754 | 13384 | 48.4 | MQFSTPIFAGLLAAAANAAPGPIVGAAVQARQNSQDFCGASPFYDRYDERTKLGWNLPKVSDCQRLAENVANDGNKWKVTSSPNNIASSGTCTFRVSLGSASEEKVYIGNKDVADIILTSIAQY |
| 19 | Unigene0001124 | 710 | 59887 | 25.9 | LWTLLPALARLTAAEFGVAGQLFALQGGSDVNNAKLAWAAVSGASTYRVEQQQGTGGYQTVATVPGTTHDVYDLGAESTHSFRVTALDGNSQVDQSSVATLAPYTAQDTYSTHDNTQPSSTRLKSKLEDNGVYYRYNYERYSNGSFNRFVEQTSSNGYDFTGDKTVLTGVTLCAPADYSCKLEAITFLKHPSTNQFVMYAHYEKSQDYSLGWIAVAHKDPTADAFTFDGAYRPLGHDSRDLNFFNDGDSAWIVTSTDTNTNNNIYSLTSNWTAVDKFLVQVNKGGHREAPAVAKNNGVYYLFTSRAAGWLPSQPQYISASDMAGPWSAPMNVGNTATFGSQSGGVSQLSSGQLAMNSNRWSSNWPTKGGQTRQLMLPISEGSGGFISYHYYRTVQYSDDITTKGQGVYGVQTGRILSDGKPSSSSGGDASIASANDGIQNDPANVFVPSGVPFWYQIDLQNAHAISQVDLSTKLVQGSETFYKYNVTGSTDGQTFELLADQTNAVDVGFSAAFPTSTQQFRYVRINVESVVNNVNGQAANWAVGIHEVTVYGN |
| 20 | Unigene0009202 | 692 | 73496 | 28.3 | RMRVVQRLDFKMLAKAFVAALSLAASVNAVPRSHLKFHQRRAIDNATDYEYVIIGSGPGGGPLASRLAIAGHKVLLIEAGGDYGDNYNQSVPTYSLKSAEDDLIKWDYWVRHYEDLERQKKDTKMTYRNPDGSLYVGLYPSEGAEPLGILYPRTGALGGCSEHHALITTYPWRADWTYIQQLAGDDSWGPDEMREHFVKLEKAQYPVGAGHGTDGWLRTSLTNLLLVARDFKVLSIVLSAAAAMGKDGLLSSLLSTVTSLTNVLLTDVNANTDPEENVYQVPLSIDADTSKRSGTRAFILDTANAKNDDGTRKYHLDIALHTLATKIRFDESGDVPKAIGVEYLAGERLYKADPYPSASDGTAGFVAASKDVIISGGSFNTPQLLKLSGIGPKDELDQFGIKVIKDAPGVGSNMQDRYEAAIVAETAEKFPISADCTFGYDGQADPCLEKWINGKSVLDRGPYTTSGAAVGVVLKTSVAQDHPDIFIIGTPGVFNGFYPGFAYDSVKSGKKWSWLALKAQPQNNAGTVKLRSTDPRDVPEILFRSLDTGNTTDGGDERDLQALYEGLLWGREAFDKLIPLDGTFTEANPGRNVSSEADLKEYIKNELWGHHACCTAAIGPDGDPNAVLDSNFKVRGVERLRVVDASVFPKIPGTFISLPIYMISEKAAEAIING |
| 21 | Unigene0007303 | 679 | 81403 | 37.1 | MAECPVKHSNVGGGGTRNRDWWPNELRTTILRQHTERTDPFGGKFDYAQAFKSLDYAGLKKDLHALMTDSQDWWPADFGHYGGLFVRMAWHSAGTYRVFDGRGGGGEGQQRFAPLNSWPDNVSLDKARRLLLPIKLKYGNKISWADLFLLTGNVAIESMGLQTFGFAGGRADTWEADESVYWGGEQTWLGNEVRYSDGKEGLAEHGVVDGDQSKKGHTDIHSRDLEKPLGASHMGLIYVNPEGPDGVPDPLAAARDIKTTFGRMAMNHEETVALIAGGHAFGKTHGAGPSDNLGVEPNAAPIEQQGFGWKNGYKSGKGADTITSGLEVTWTSTPVKWSNKYLEYMYKYDWELTKSPAGANQWVAKTDDKIIPHAYDPNIKLKPTMLTTDLALRFDPEFDKISRKFLENPKLLDDAFARAWFKLLHRDMGPRSRWLGPEIPKEVSIWEDPIPDPPAQTISESDAEALKKQILSSGVAPQKLISVAFAAASSYRGSDKRGGANGARIRLEPQRSWQVNQPAQVQEVVSALEKIQKDSGKQVSLADLIVLAGCAAIEQASGLKVPFTPGRTDASQEQTDVESFSHLEPAVDGFRNYGQSTNRVTTEQFLVDRAHLLTLSAPETTALLGGLRVLNQNWDGSQHGVFTKQPGTLTNDFFKNLLDNNTEWKSTGGEVFEGVDRKSGEKKWTATRADLIFGHHPEFRAISEVYGAADGGAKFKQDFVNVWVKVANADRFDLTSAA |
| 21 | Unigene0007301 | 679 | 82199 | 36.8 | MAECPVKHSNVGGGGTRNRDWWPNELRTTILRQHTERTDPFGGKFDYAQAFKSLDYAGLKKDLHALMTDSQDWWPADFGHYGGLFVRMAWHSAGTYRVFDGRGGGGEGQQRFAPLNSWPDNVSLDKARRLLLPIKLKYGNKISWADLFLLTGNVAIESMGLQTFGFAGGRADTWEADESVYWGGEQTWLGNEVRYSDGKEGLAEHGVVDGDQSKKGHTDIHSRDLEKPLGASHMGLIYVNPEGPDGVPDPLAAARDIKTTFGRMAMNHEETVALIAGGHAFGKTHGAGPSDNLGVEPNAAPIEQQGFGWKNGYKSGKGADTITSGLEVTWTSTPVKWSNKYLEYMYKYDWELTKSPAGANQWVAKTDDKIIPHAYDPNIKLKPTMLTTDLALRFDPEFDKISRKFLENPKLLDDAFARAWFKLLHRDMGPRSRWLGPEIPKEVSIWEDPIPDPPAQTISESDAEALKKQILSSGVAPQKLISVAFAAASSYRGSDKRGGANGARIRLEPQRSWQVNQPAQVQEVVSALEKIQKDSGKQVSLADLIVLAGCAAIEQASGLKVPFTPGRTDASQEQTDVESFSHLEPAVDGFRNYGQSTNRVTTEQFLVDRAHLLTLSAPETTALLGGLRVLNQNWDGSQHGVFTKQPGTLTNDFFKNLLDNNTEWKSTGGEVFEGVDRKSGEKKWTATRADLIFGHHPEFRAISEVYGAADGGAKFKQDFVNVWVKVANADRFDLTSAASKSKPRL |
| 21 | Unigene0007302 | 679 | 82199 | 36.8 | MAECPVKHSNVGGGGTRNRDWWPNELRTTILRQHTERTDPFGGKFDYAQAFKSLDYAGLKKDLHALMTDSQDWWPADFGHYGGLFVRMAWHSAGTYRVFDGRGGGGEGQQRFAPLNSWPDNVSLDKARRLLLPIKLKYGNKISWADLFLLTGNVAIESMGLQTFGFAGGRADTWEADESVYWGGEQTWLGNEVRYSDGKEGLAEHGVVDGDQSKKGHTDIHSRDLEKPLGASHMGLIYVNPEGPDGVPDPLAAARDIKTTFGRMAMNHEETVALIAGGHAFGKTHGAGPSDNLGVEPNAAPIEQQGFGWKNGYKSGKGADTITSGLEVTWTSTPVKWSNKYLEYMYKYDWELTKSPAGANQWVAKTDDKIIPHAYDPNIKLKPTMLTTDLALRFDPEFDKISRKFLENPKLLDDAFARAWFKLLHRDMGPRSRWLGPEIPKEVSIWEDPIPDPPAQTISESDAEALKKQILSSGVAPQKLISVAFAAASSYRGSDKRGGANGARIRLEPQRSWQVNQPAQVQEVVSALEKIQKDSGKQVSLADLIVLAGCAAIEQASGLKVPFTPGRTDASQEQTDVESFSHLEPAVDGFRNYGQSTNRVTTEQFLVDRAHLLTLSAPETTALLGGLRVLNQNWDGSQHGVFTKQPGTLTNDFFKNLLDNNTEWKSTGGEVFEGVDRKSGEKKWTATRADLIFGHHPEFRAISEVYGAADGGAKFKQDFVNVWVKVANADRFDLTSAASKSKPRL |
| 22 | Unigene0005357 | 664 | 14914 | 45.9 | LLVSAIATLANAYHKPQDTPTWGALLSPNGDLPVTRGKPFDITWNPQYSDPKYDASQQTVSLVLCRGNSNTCNPDPTAIVEGVKATDNKWTWNVPCDLPAGEKNTDTGYGMLIIVDQDGQFQYSTQFSVLQGDTC |
| 22 | Unigene0005356 | 664 | 14914 | 45.9 | LLVSAIATLANAYHKPQDTPTWGALLSPNGDLPVTRGKPFDITWNPQYSDPKYDASQQTVSLVLCRGNSNTCNPDPTAIVEGVKATDNKWTWNVPCDLPAGEKNTDTGYGMLIIVDQDGQFQYSTQFSVLQGDTC |
| 23 | Unigene0004958 | 619 | 85577 | 16.6 | NNHDLAAQLDKRQAGQVITTGARGLGDGRVHVRLEINELANNRPDMWSLYIRTMAQWKEAAKDDWTGYWGISKIHGVPRMDWDGVQRCDDCDGADGYCTHDSVHFPAWHRAYMALFEQELIKHALEVADSFDGAFGDRLKAAAQALRAPFWDWAANPDQGSTALPPFISGQQVTIEGPNGQETVDNPLYSYHFTDPSDMEFAVFVDWPDTFRWPNSNNKDAHSQEQQTVDAFSNINGNLQDQVYQLLTQCKSYLGFATDASGDQRCANSLEGIHNTIHTNAGGSGANGVSGGHMTFLPLAAFDPIFYLHHANVDRLFTLWQTANPSTYGASQVAPHSTWTIAQGSNQDADSPLMPFRKAADQYWTTNDVRDFAATFAYTYPEFIVGDGQRNTIVDYINQLYGSSPSLTASALSARAEPVDGSNTEGGNGGRGLEAPQGYGGSSSSSSSPASSETGGSGSGTGIGIGIGPISISIGLPWNKPTGGASGPYPTVTGAWNGTKTAGYPAPTGTGAPLNPAFIAPNGSVYQYQCNIETPRYALNGSYSVYVFDGQPGTNDTSEWLGEKNCIGQIGVLAGGDMAHEVFSAGSVPITRYLQKLYLSGKISALSEDVVIPYMKKNLNWIIVYDGERVNPESLNGYKASFLSGLLSPVLDGELPSWSNLLPQVDVTKDKVGGITEAVEGLLGGVGDALSDAIGDIGGNIGGIIGGLIPGGNGNGNSNAPQGYGSASSAPSYPGKTEDSPVPATTAPPSQNPPSYPSPPEEEEEVTTTVYTTQYVTYCPCTESTAAPQQPTATAYAKQY |
| 24 | Unigene0004570 | 591 | 37088 | 33.3 | MTVRREWGDMPSEAKQDYLRAIHCLKELPGKTDRNPDTGVPGARSRWDDFTVAHMRNVNDVHRSPWLAVWHRQFVWRLEKALQEECGYKYGHPYWHWSKYLDQDVNTWPMFDNSPDSISGNGTQTGPLCACVNEGPLANWTVTLGPFPGSWGCSANPRDDGLGYNPRCIDRTFQPSNLRSNEYSDVVFVIDNSTDADSFGRHVELEQPSVHNYPHIFMGGTQIDVTFSSQDPWFYFHHFMLEYVFNLWQSRDWDARTASLPSPDTFTENRRRDGWALPVPAPTLDSTLWLQEVFENTTVREAMWPIKNQYCYRYE |
| 25 | Unigene0012195 | 584 | 56563 | 25.6 | NAEWDVIIVGAGPGGIVTADRMSEAGKRTLLLEQGGGSYYSTGGRERPEWLSNTELSRVDVPGLYSSIFSGDSKLLCGDKQNGGFGGCTIGGSSAINAELFIQPPASDFDTYYPEQWKSKDMKSAIEKVKAKQPYTNTPSADGKRYMQSGYDASQKWLVNGAGLKNVAVNGKPNEKDGVFGRTNYAYERGQRSGPAKTYLTSALARPNFSLKSGVQVKRVQRNGGKATAVEATENGQTTTYRLSQTGRVILSGGSFFSPQLLMMSGIGDPASLSNLTQNKLLDMKATAWINNTAVGDNLYDNPNTFIVLSGPSVKSYNFNYNSPIASDKDLYLKSRSGPYSSAGPTGLLWDKVKQADGSSIAVQGTVNVAGSFDYTGNDTITLNVYATSGAKSKSRLVVNEKGLPGMASDFLFSNPADADAVATVIHKIFQALPASGLTSLNLPATSSKDEILKYITSPSKYTKGYVNHWSGSCKIGTCVDQNTKVKGMENLHVVDSSITEAFTTNPMFGIVAVAERASELILALDKK |
| 26 | Unigene0012676 | 581 | 25022 | 22.4 | SQQHTRUHTIQTNLLHTUFIMKASVICASVLAGFAAAAPEASKKYNGTPSKPTYDKDCDKNGYYTGAKGFPIPFTSTYRVKAVPEEVVNTMNAKTGGLPGAVGFYDLGINSELDTICYYIRLYNIRGEYVSPARTATHIHESARGQSGPPRIAFPNPQPVAGKNYRVSVGCLTGPFTTGVVNNMTMADTGAGFRLAQIEANPKGFNADVHTNFADPSQGINATAGAVRGQLA |
| 27 | Unigene0001938 | 578 | 17291 | 28.3 | FXNITQPNIKMQFKNIVATIAAFAVAGVVASPVDVVARAEQAPSKQQVIDSANAYMKEHAADKLSEADVALAAKAAQQFGSCWCGCPPLGPPPPPFYPGQDCPPQLRFYLGAARNYWDYPELWQDGYSKQTCVGLINLASCNDGPFSGLNILSGVGSIL |
| 28 | Unigene0003346 | 537 | 13980 | 25.9 | MKFTAALLSIAALATATTVSYDEGYDDAARSLTAVSCSDGNTGLITKGYSTQGSIPTFPNIGGSWEIAGWGSPQCGACYTVSYQGRSINVVGIDRSGDGLNLSKAALNTLTGGRAVEVGRIDAQVTRVDSGVCGL |
| 29 | Unigene0010220 | 505 | 71430 | 21 | MRSTTTAAAVLAAAPLACGWNLPNVFKRQNDQCDTITTEKIKNLPNNALFTRWRPQSHFIAPAGWMNDPAGFMYDPVRDEYHGHYQWHPNHINWGNISWGGAVSKDLINWEDQGGWRDAEALALGPTGNGSHDGLGIFSGSALSVNLQGEQDGTLLLFYTGAQFLPTKWLFPYTRGTESQSMALSTDGGKTWERLENNPVINASTNEPPMNWDLTGFRDPFIEPNPELDALRGVEKHFYSVWGSGIKGIGPRMPLWAAPANDLTNWTFLGSLWEPAQNTSLGPILSTGQYAYNFELSGFFRLKDRAGEDHWYTNMGTEGGGGPDVPFHTNAQWQLWNEGEVVRRQNGSVEFIPTIGGAGDWGLGYAATSFNDTKNDRRVQYAWIKEDNLGDQALFSAPQQGFQGALTIPRELFVHEVDGVTNTTELTEAKFVTVKNGTAFTLGVRPVEDVADGLREGAAYSDFAPRTYDRSTILQSNTTSHVSIKATVKNATGPVGLTIGASPDGEEFTNIIYEPSNYTILVDRRQSTTMDLFNRETITGYFQPYKLLSTNETEEISFDVWVDGSAVEIYVNERFALSARIYPSKTCSTGWGVFVGEDSEAEFGKIEAWDGTKNVWPQRPANSSSELVIDTPEETNNGAWWIGN |
| 30 | Unigene0014595 | 504 | 19984 | 41.5 | ALLGLAGSALAAPAPQQEDQIPTFGQPFGIEAEGKGLSYAQISAVNGRLMIGGTQTATCERGERQDLATFTLHSDHRLYMYTNSNPVQNVWVDASVMGQGNAGYSSPDRLGYRSSIDPFYISKKGALTFDHIPAKACPGDEKGTYFIWFSSAKKPAEQEGCIKVQLKTYYAPARIACNYTESA |
| 31 | Unigene0005491 | 475 | 19318 | 29.5 | AILAAAAYLPSTLGCIWFNATLHEGANVANSAEEAINLKNEKTTGGQVGDVRLDVYFWDDKDNDDIVEPLKDAFCTGVDLKPYADNTWVVPCTPEEKQVELDIVYNPDITAGGVLISVFKYKNPRVQAEASNNPDKDLIYTFHTDSNDGKGEFFEDKIFCRDCTGSAGQPVKC |
| 32 | Unigene0003767 | 460 | 46387 | 40 | IREARPHEQAEASLPLRLQPAPKKLHSYLUARVAALLGQLESPFKTTLSSFTMGKVLLVLYDGHEHAKQEPRLLGTTENELGLRKWIEDQGHTLVTTSDKEGENSKFDQELVDAEVIITTPFHPGYLTAERLAKAKNLKIAVTAGIGSDHVDLNAANKTNGGITVAEVTGSNVVSVAEHVVMTILVLVRNFVPAHEQIASGDWNVAAVAKNEYDLEGKVVGTVAVGRIGERVLRRLKPFDCKELLYYDYQPLSPEKEKEIGCRRVENLEEMLAQCDVVTINCPLHEKTRGLFNKELLSKMKKGSWLVNTARGAIVVKEDVADALKSGQLRGYGGDVWFPQPAPKDHPLRYASYTTWGGGNATVPHMSGTSIDAQARYSAGVKNILEEYFSGRQNYRPEDLIVHQGDYATKAYGQRNKA |
| 32 | Unigene0003768 | 460 | 45853 | 40 | MVFARSALRAARPASSLLSQRATTSFAHRGASLSKTAGLGGIRTLTATSRQQGKVLLVLYDGHEHAKQEPRLLGTTENELGLRKWIEDQGHTLVTTSDKEGENSKFDQELVDAEVIITTPFHPGYLTAERLAKAKNLKIAVTAGIGSDHVDLNAANKTNGGITVAEVTGSNVVSVAEHVVMTILVLVRNFVPAHEQIASGDWNVAAVAKNEYDLEGKVVGTVAVGRIGERVLRRLKPFDCKELLYYDYQPLSPEKEKEIGCRRVENLEEMLAQCDVVTINCPLHEKTRGLFNKELLSKMKKGSWLVNTARGAIVVKEDVADALKSGQLRGYGGDVWFPQPAPKDHPLRYASYTTWGGGNATVPHMSGTSIDAQARYSAGVKNILEEYFSGRQNYRPEDLIVHQGDYATKAYGQRNKA |
| 33 | Unigene0002816 | 459 | 14694 | 8.8 | XLRNSSSITFKMLFTTVAFATAALFSPLALAQLGRSGTSPSEDSIARADYCQCVGSFAFPDNSPNQPLTELVCSVFSGKRIDVDGYAQCFGVGPADTGFSNSCASYGDNGAKGARCCRQGQAYKDCHVIIPGINGK |
| 34 | Unigene0007243 | 439 | 26213 | 37.4 | LTPALAAGAAVSKRIVGGELATLGQFQYLVSITGLYDGTDAYSQICGGALLNPTTVLTAAHCVQWVVDAPKDLNVRAGTLTFDQGGVKSVVESYVQHPEYKNSNHDFAILKLATPIEESATISYAKLQQEDVDPIAGAVATVAGWGLDSDSGLRLPDLYWVNIPVVDRQECKRVFAAHQNEITDEMWCAGTKEGGKGDCSGDSGGPVTIDGVVAGVVSWSLGCASAEYPSVYAKVSKAIPFIKAHL |
| 35 | Unigene0014793 | 438 | 61224 | 23.9 | MPGFSQANELPAWKALMEHHDKLGRGMVLKSEFEKDPQRFEKYSRTFANEADGTEILFDFSKNFITEDTLPLLVKLAQEAKLEELRDDMFKGEKINFTEQRAVYHVALRNVKNEPMQVDGKSVVEEVNSVLDHMKEFSEQVRSGEWKGHTGKPIDTIVNIGIGGSDLGPVMVTEALKPYGKKGMKLHFVSNIDGTHIAEALADSDRETTLFLIASKTFTTAETVTNATTAKKWFLEKASESDVAKHFVALSTNDKEVSKFGIDTKNMFGFSDWVGGRYSVWSAIGLSVALYIGFDNFKQFLAGAQAMDHHFKTAPLEQNIPVIGGLLSVWYSDFFGAQTHLVSPFDQYMHRFPAYLQQLSMESNGKAITRSGDYVKYTTGAILFGEPCTNAQHSFFQLLHQGTKLIPADFIIAAKSHNPVENNKHQHMLASNYFAQAEALMIGKTPETVKNEGAAAELVPHKVFLGNRPTTSILAEQITPGTLGALIAYYEHVTFTEGAIWNINSFDQWGVELGKALAKSIQTELDNPGESSQHDSSTSGLINAFKKKAGIP |
| 36 | Unigene0004717 | 416 | 54262 | 23.3 | MARVLVAIVALARLASAIIGEQDFSAEDIIVRDVAILGAGASGTYAAIRLREDYNLSIAVVEKDDHIGGHTDTYTDPETRKPVDYGVLSFWDYGPARNYFARLGVETVPAPQDSGITAYVDSETARNLTNYHVPAFSDVLKSIAAYGNESAKYDDILVPGYWNFPSGDDIPADLLLPYGEFSQKYGVDNFYPMLQIIAGVGVGGVRDIPLLYVIGFGLGHPAIQSLLQNGLFVPKSGSNAEIYQNAYARIKEDVLLSSSVVSAERDEHDGVRLVVQTADNCKKLIKAKKLLVTAPPSIGNLESLDLDDQESAVFVSSTPRPIHVGLAKTSAIPRNYSVQYVSSKVAPDKYLDMYQDIKYDLKIQSTGPEELQLFRVLLETTPDLPLTEEEAKAYITSQVQKLAAAGTLNSVKPPDVGFGSGDAQTADGGAVTVEFKAFKSHSSVMWRLPADEIKAGFIHNLYALQGHRATWYTGSLWCTDFSSNVWAFTDTVLERMV |
| 37 | Unigene0005411 | 401 | 38982 | 24 | FIRDIPKVELHIHIEGTLTPRLRWDLAQKNNVTLQYKTFEELQASYDELANLPQENYLPAFLEGYYGGMDVLLHEDDFYQLAIDYYDKSVSLNVRYSEIYFDIQAHTRRNVSVAAVMNGFLRAKKEAKVKYNYDSTFILAFLRELSVESAQEHYDLAAPWRGTLFTAVGLDSDERQRPPILFDSVYRQARKDGLKLTAHCDVNQEDTYEHIRQAALEVGGGGLDRIDHGLNAAEKPELIEAIKIRGIGLALCPAAYSLIANSSFIFPRMRTLYDAGIPITVNSDDPTYMRNYYVSEALQMTQDETPFSREEVVQLQRNAIEISWADPIVKANIAAELEAFA |
| 37 | Unigene0005410 | 401 | 38982 | 24 | FIRDIPKVELHIHIEGTLTPRLRWDLAQKNNVTLQYKTFEELQASYDELANLPQENYLPAFLEGYYGGMDVLLHEDDFYQLAIDYYDKSVSLNVRYSEIYFDIQAHTRRNVSVAAVMNGFLRAKKEAKVKYNYDSTFILAFLRELSVESAQEHYDLAAPWRGTLFTAVGLDSDERQRPPILFDSVYRQARKDGLKLTAHCDVNQEDTYEHIRQAALEVGGGGLDRIDHGLNAAEKPELIEAIKIRGIGLALCPAAYSLIANSSFIFPRMRTLYDAGIPITVNSDDPTYMRNYYVSEALQMTQDETPFSREEVVQLQRNAIEISWADPIVKANIAAELEAFA |
| 38 | Unigene0000010 | 400 | 56863 | 21.9 | VLPFILGSSIARCNALALRDNARVAAQSPVVFNLGVDRIREISEYVLAQDQAVFNDILSTITPETATFNNTLAPWSRHRDEVAGLITAFGYITDPVAYNATNDFFAARSPFLDNVTNSEEYWTLVKAVYDNLANENQLWTPEGRLATMFHQSRVEAGLSIPKGPDRDRYSTLVQEQQNLTQAFNTNAQQPAAPPNLFFSREELGGVDPQLLATFANGTGADAGKLGINVLTQASAVIPVAKNQRVRQELAIGYNRLARDNVDILKQAYAGRDEMAQLLGYPNYATARLQDTIAGSPQNALALINEIGDTLKPSAADSVDQLNIIVKRDNSTASAYRWDIEYARIAIDEAQTNLTDDNFGSYFVPTFTVPTALQLFGQALGFDLQLVHGSELDALSPTGKGADILPAPQASLYEVRNSGSNDFAGYFYTDILARPGKISNSTQGTAIQRAHVSVNGTRTYPSVILNGGYYPTQHLSAEALANLFWQIGVAYWHLSCESEFGDLCGDAGAPADFSQMPGQMME |
| 39 | Unigene0013751 | 395 | 59107 | 16.8 | MKVSTLALLATPLLADASQSSLRRVTQPPSSGPQYTTDLFEGTRLKEEQSVLNNSDLTYTDRNGKKNWSPLSALRAGEKGPLLLQDAALIDTLATFNRERVPERVVHARGAGAHGFFEATTDYAANFSAASVFQKGTRTSVTMRFSTVGGARGSADQARDPRGFAIKFRTKEGILDWVFNNTPVFFIRDPAKFPRFIHTQKTDPAKNSRDWNTFWSWPAQFPEALLQFLRIFSDLGTPYGFRHMDGWSGHTYKLVKDDGSWVYTRVYLSTDQGVRNMTASEAAQISGENDAWATADLYDHIEAGEYPSWTVGIATMTPEQAEEYRYDVLDLTKDWLGVEYQEIGRIFLTQNPDNYHAEIEQSHFTPANIVPGWEPSNDPVLQSRLFAYGDAGRYRIGVNAEDIPVNCPLTSVANFDRDGHLSSTGNQGSRLNFPAEYLDPINVIDRPGTAIESPLEGDSTVQWLSSIDEDIDYEQPRLFYQGFSQTDKDHLYSNIAGTLVNVNHQEVLDALFVQFGKIDQALLSGVQTA |
| 40 | Unigene0004728 | 385 | 53879 | 25.9 | LSVELETPTSGKYTQPTGLFINNEFVKGVDGKTFEVINPATEEVITSVHEATEKDVDIAVAAARKAFEGPWRQETPENRGKLLNKLADLFEKNLDLLASVEALDNGKAQAMAKVDISMCAGCLRYYGGWADKIEGKVVDTSPDTFNYIKKEPIGVCGQIIPWNFPLLMWAWKIGPAIATGNTVVLKTAEQTPLGGLVAATLIKEAGFPPGVVNVISGFGKVAGAAIASHMDVDKVAFTGSTVVGRQILKAAAGSNLKKVTLELGGKSPNIVFDDADIDNAISWVNFGIFFNHGQCCCAGSRIYVQEGVYDQFIQRFKERAAKNVVGDPFAKDTFQGPQVSQVQFDRIMNYIKAGKDAGATVEIGGNRKGDKGYFIEPTIFSNVTEDMQIVQEEIFGPVCSISKFKTKEEVIKIGNSTTYGLAAAVHTKNLNTAIEVSNALKAGTVWVNTYNTLHHQLPFGGYKESGIGRELGENALDNYVQYKTVSIRLGDALFG |
| 41 | Unigene0006098 | 369 | 47492 | 21.8 | SVQLETPHSGKYEQPIGLFINGEFVPSSKGRKFETINPTTEEVITSVYEGDEEDIDKAVVAARKAFEGPWKKVTPEDRGKLLNRLAQLFDENNDLLASIEALDNGKSITMAKVDINLCSGCLRYYGGWADKIEGKVVDVAPDTFNYIRKEAIGVCGQIIPWNFPLLMWSWKVGPAIAAGNTVVIKTAEQTPLSALVAARLVSEAGFPPGVINIVSGFGRTAGAALASHMDVDKIAFTGSTVVGRTILKAAASSNLKKVTLELGGKSPNIVFNDANIDNAISWVNFGIFYNHGQCCVAGSRIYVQEGIYDEFVKRFAERARANKVGDPFAKDTFQGPQVSKVQFDRVMSYINSGKAAGAKVETGGTRKGDKGYFIEPTIFSNVTEDMQIQQEEIFGPVCSIAKFKTKEDAIRVGNNTNYGLAAAVHTQNLNTAIEV |
| 42 | Unigene0012711 | 338 | 29330 | 38.7 | STAAFLAYLITFVQADADDIAAGHHIIWSYPGPNIPHDLVAAARDGKVSGVIFYSENIDKANDLPGQIRNLQNVYRQSPSYPGYPLLLVTDQEGGIVNRLPGGPSTSAKSIGSSSDPLSSATQAGQTVAQIFSKFGINGDLAPVLDVHRNENDFTDREQRSFSSDPKIVSSSAAAWISALQSQNYPATAKHFPGLGAAKADQNTDLKPVTINLSADELRGKDMLPYIAAINQSHVKMIMTSWAVYPALDSANPAGLSRKIVQGELRQRLGFRGV |
| 43 | Unigene0007874 | 321 | 14791 | 28.7 | SDTGAAVEISVENPGFREGETVYTIKQIDLFNLLPPGEDNGVDVRQSIDWGFGPVKVTGFADTSTLEIGIQLVVVGITVANLFGNLQDGVVASIDLFAVKGHVKFFLKNGKEVWIELDLQVVFDGHFTKTEKIFSL |
| 44 | Unigene0001416 | 307 | 96898 | 15 | MLSKAVLGAVLAAVAASVAPEGNDTAPFPNTTYPDAISPDPAVAQGTRSNQTSPPKYPSPWSTGLGDWEAAYKRATEIVAQLTLEEKVNITTGSGWQSEECVGNTGSVPRLGIRSLCFQDSPTGMRYTDFVSVFPSGVNVGATWSKALAHARGVAMAEEFRDKGVHGFLGPVAGPIGRSPAGGRNWEGFSPDPYLTGALFAESIKGVQSTGQIAVGKHYIGNEQEHFRQTPETSGFGLANITYPGSSNIDDVTLHELYAWPFADGVRAGLASIMCSYNRVNNSDGCQNSYLQNYVLKNELGFQGFIVSDWQATHSGVSAILAGLDVSMPGDIVFNDGLSYFGPNLTIAVLNGTVPQWRLDDMVVRVLAGWYYVDGDSEENNKPTNFNSWTKDTYGAVHRYAGPQYGYELINDHVDVRKEHGRLIREIGSASTVLLKNVNHTLPLTGKEKLTTVFGDDAGPNLAGPNGCSNRACAQGSIAIGWGSGTAEFPYLITPDAAIQREVTDHYGAYESILSNGALPQIQALARRAGQVSGVCIAFGAANAGEGFVAPDSNYGDRNNLTFWQGADQMLRNVTANCNNTILVVHSPGAVEVEEYKDHPNVTAILWAGMPGEQSGNSLADVLYGRVNPGAKLPYTIGRNRSDYGTDVLYYINGDPPQFDFQEGNFIDYRTFDQRGIEPVYEFGFGLSYTTFNYSNIEVRDTGAGPYVPQSGSTQEAPTFGTVDMDPAAHVFPSENFTRVPYFIYPYLNSSNLSESYGHTDFGDNSFIPADGLSGTAQPLLPAGGAPGGNPSLWDILFVVSVDITNTGDRDGDEVAQLYVSLGGPYDPKVVLRGFERVNIPKGKTVTVDFHLTRRDLSNWDSGKQDWVVRTENRKRVFVGSSSRQLLLESDLEL |
| 45 | Unigene0004668 | 306 | 101524 | 13.5 | MPRLFSPLTAVAGLLGSVTAQSYTNSSSESVQVRWIGSTPEYHSGTTFGLPWARGKHFLNSTRFSASGDIDLQSWATAYWTDGSLKWTAHAIPETENIADEYTITASSGSPRTSQSAGIAVTDSDGSVTIDTGKISASFPKSGGVVVENIETASGKVVGQNGRLVLHSQSGVADNAEDRTNSSINYSNFESKVDNVTTSRDNQARALVTVRGTHTLASGADHIDWLPFILRFYLYANSDAIRIVHTLVFDGNSSTDFISGIGLRFDVPLAEEELYNRHVRIAGVDSGLLNEAVKGITGLRRDPGQNVRTAQYEGDETPTPDTWDPRVGNATRRGWIPNWNDYSLTQLSPDGFTLKKRTQAGQSWLNIPGSTRSGGLAYLGGATQGGLAVALRDFWKKYPTGLDIRNAATDVGEVTVWIYSPEAQPLDLRPYHDGLGQENYADQLDALEITYEDWEDGFDTPYGIARTNEFYIHAFEATPPRDRLSALVTHANNPPVLFTEPDYIHSTKAAGTWWAPPSTNTSSALAARIESNLDFLATFYQTEVEQRRWYGLFDFGDIMHTYDVDRHQWRYDIGGYAWDNSELSPDLFFWPYFLRTGREDVYRFAEALTRHTGEVDVYHIGQWKGLGTRHGVQHWGDSAKQARISTAQYRKIFYFISGGDERVGELVEETLDTDKTYETLDPNRKVRTDGWVPAPGNRATIGLGTDWSGLAASWLLEWERQGPRAEEAKLKLTNTIIGIVSLKNGFVTGSGLYNNTDGTLDPPPTDPDNNGVVAVSHLSAAFGLGEVVAELIEHYGDELPEGFEQVWLDYCYYYRAPAAEQQARYGGNWTRTATLRQDHSRLLAYGYYRTGNETWAQRAWSEFEADGLNTTDTWATTRINGSAVLAPVDEVPWLSTNDAANYGLAAISNLAYIPEFL |
| 46 | Unigene0009720 | 306 | 22049 | 28.6 | MQYTTSVLVALLPALGTATRFRSPGASDSAPQAAMLGASNVQQKPNGQVPDSNGNWQNSYSGGNGNDQWRHGNTNAGIAPGVNDCGFSSFIEITQPAGNNPLVTDCQALVASIQQDYEWTVTSQGQTLVYKGSCAFNAVTSSGQDPSLTGKVGNADIIDLVTDSIKKYAKDGTVGCRGGYSLYVSSAGAMPCDSPDAPSGKQVSIDWTLT |
| 47 | Unigene0001824 | 295 | 15935 | 26.6 | MVKAVAVLRGDSNIKGTVTFTQENEGSATTVEWDISGHDANAERGMHVHAFGDNTNGCTSAGPHFNPHNKEHGAPEDSERHVGDLGNFKTDGQGNAKGSVQDKLIKLIGPESVLGRTVVVHAGTDDLGKGGHAESKKTGNAGGRPACGVIGIAA |
| 48 | Unigene0001089 | 287 | 49594 | 11.1 | MKAVLSAGALAFASLACAKAPPRKSLGSLPPIVSEGNAFWTEGGERFYIRGVAYQPGGAADARDPLLNLEQFREDVKAFIDLGINTIRIYTVDNSGDHDEAMKLLDEAGIYLALDVNTPKASLNRENIDSLHASYNDVYLQSVFATIDTFSKYNNLLAFFSANEVINARNNTNAAPYIKAVTRDMRNYIRANSPRPIPVGYSSADVAENIESQALYFACGEDEIARSDFFAFNDYSWCDPSDFRKSGWDAKVRTYSNYSLPLFLSEFGCITNRRDWNEIAALYSDDMTPVYSGGLAYEYTLEANGYGLVEMGEDGKATPNGDFDRLKEAYAKTPAPSGDGGARRGDRTVPECPAESEEWQVSTTLLPEMPKDAEKFLKDGAGTPPGLEVETQWAGTPSETDPDLSNGVSSTPAEGTNYSGGNSSSSSGGDDSSAAASTANPIVFMTVVVAAVAVAIGM |
| 49 | Unigene0003366 | 282 | 49961 | 23.7 | MTTNNVSEPEFEQAYKELASTLENSSLFEKHPEYKTALKVAAIPERVIQFRVVWEDDKGECQVNRGFRVQYNSALGPYKGGLRFHPSVNLSILKFLGFEQIFKNALTGLSMGGGKGGADFDPKGKSDNEIRKFCVSFMRELSKHIGADTDVPAGDIGVSPREIGWMFGTYRAERNRWEGVLTGKGGSWGGSLIRPEATGYGLVYYVQHMIQYASGGKESFQGKRVAISGSGNVAQYAALKAIELGATIVSLSDSKGAIIATEGEGITPEVVNYIADLKVKRKSLTALSESTEYKDKYKYIEGARPWVHVGKVDVALPCATQNEISGEEAEALVAAGCKFTAEGSNMGSTQEAIDVFEASRKEKKTEGIWYGPGKAANAGGVAVSGLEMAQNSARLSWTSEEVDQKLKGIMEAAFKNCLETAKEYVPAGDGELPSLVAGANIAGFSKVAAAMRDQGDWWAY |
| 50 | Unigene0005375 | 280 | 21651 | 10.7 | KRQVTCATGNGLYILVARGSNQPVGEGTVGPVANLVEAQVAGSYSHAIDYPATIIALDSNYVTSVVDGIEDTKQSIEDYVAACGSNSRIALIGYSQGGNVMTDTLAGGTGKPAPIAEQYRQNIVGVAVFGDPRFNVGQPYSRGTSTRSGIFARQSSLAALGTWANVLVSYCDENDPFCASGFDLDVHSATVAKYAQQAADFIIGLA |
| 51 | Unigene0008487 | 277 | 57818 | 16.9 | HLIMLAQLGGIAQAAYVWPSKYDRLDDLLYLQGGYIRNGELSDQVRTCDFGAGAPGIQKSAEWLRTAFHDAATHDTATKLGGLDASIQFELDRAENKGAALNNTLADISSSYNIRDSASDLLALAMVMAVERCGDLVVPLRFGRVDATKEGIAGVPEAHTDLETAQKRFETMGFSQEEMITLVACGHTIGGVHSVDHPEIVTEGGVSEENVARFDTTTGNFDNAVVNEFLNNTTKNPLVVAKNDTLNSDKRIFTSDKNQTMTKLSDPAYFKSQCESLLERMINTVPGNVQLTEPYQPADVRPTISSYQFRGDKIELAGRVRVRTTESTGRNASDIEVTLLPKDSTQTISTTMATFKGGSSSGYNREIFNWYEFNSTLDAGATKAFDIQLKTRSTGEVVKNDNAGAGGFPLNADIFWQRESSCMAFDQSTQTRGFSVIAAIREQVADNAAPQIVVVQRETMLGSHMPRLVQEKIEMKRTEKVVGGYVFYQADATLRDQSVSGTMFDIVVGESKLEFLETNDVASNAC |
| 52 | Unigene0013867 | 276 | 10311 | 25 | MQFTSTSLLIASVAMLAAAKPAPQGFNPGESKFVNAPTKQSCNEVPGGNGYYCDSAANLYYCTAGYPQLCQSCGRGMNFNQPSGCTASDDGKAGNC |
| 53 | Unigene0011015 | 268 | 18098 | 14.6 | MIGITNLFILATAALATVIPRETSAVAQIHLSIDEINSGLATISNDIKNFNATAILSDLESLSSTILQAGRDVAGLNGTVSESEALVVTYYVRNAMQPAVDETILDLRKAKQKLNRAGVLEDVAKKLEDLGSFVIVLGAALLSKTPESKKADAQEVLDGIVTTFRDAVKAL |
| 54 | Unigene0004910 | 266 | 49764 | 16.1 | MGLLGSFKDRIKSLVHRSSRKKPQGDSGRPAGDPASSTITKELEQLVSLWRPSQAQQKDIVTRDQAPLKTQNRAVQNVQAATTSGTLQIVLENQSNNADMYAYITGQAIDNNGALFLLQSDARTPYYPSSPSSTGQPIPVNISIPLGAPGNRVNATIPRIAGGRIWFSQGSQLTFLLNPGPGLVEPSVFNPSDPNYSRNFGFCEFTFNSAQVFVNISYVDFVSNIPIALTLQDTSGGTQHVSGMRANGLATVSQGLRDQTARDGRRWSSLIVQSNGRDLRALSPNSGITNNPDWFATYWTDYVNSVYSRYRNEDLIIDTQAQWGEVRGRVGNDGVLDCGDGSRFPRPNAKDIFGNSTGPFATGGNAKTNAIIPRLAAAFNRSTLLLSNQTPNGTNPSQYYQNSPTNHYARLVHAANVDGIGYAFPYDDVTPDGGKPQEGAIFSFSPSRLLVTAGGNNAHA |
| 55 | Unigene0011157 | 265 | 9322 | 29.5 | MKISIALTLAFAVSYSSAFCRRNTPVVCCEQFKGKPCCSAPESCCDEDCDISVGIDKRDLLELEYVHPSRDLHKEWRR |
| 56 | Unigene0010218 | 255 | 83583 | 12.6 | GNVFAGASLPYGMAKAVADTDSDSRQGGFTTDGANITGFSGMHDSGTGGNPSLGLFPLFPFSSCEGDEINGCEFPKKDRSEPYVNTSLKASPGYFSIDLQSGVRGEMTAAFHTALFQFTFPESGNGSPVIYLDLSDLSDSRQDNATISVEDSGRMTGYGRFEPSFGQEKYIAYFCADFKGGAVRDTGVVVNSRGSADVKDLKISRGINGFPLPGGGFVRFSDRTPVIARVGYSFISSEQACQLAESEIPDFDFRATQKSAVDAWTEKISVISVETNDVDEDTLKNFYSGVYRTFINPQNYTGVQPVVDANTIYFDSFYCIWDHFRSQFPFLVLVDPLAMEQIIQGLLTLYDIQGWLPDCHMSLSKGYTQGGSNADVVMADAYSKLNSTNIDWNKVYEAVVRDAEEEPYDWCCQGRGGLDSWKSLHYIPVEDFDYKGFGTHTRSISRTLEYSYNDYCISTIAQGLGKQGDVEKYQQTSGNWRNLFRADQESNLFNSSTSTGFTGFFQPKYLNQTWGRQDPLYCSNIDNSPTKSCSLQNTASETYESSIWEYSFYVPHDMAQLIATLGGPETFVRRLEYLHDQNITYIGNEPSFLTVYQYHYAGRPALSATRAHFYVPRFFSPTLGGLPGNDDSGTMGAFLAFTMMGLIPNPGQNVYFIIPPFFEEVRVKHPVTGNTAVIRNVNFDGGNYKNVYIQSATLNGEDYTRNWIGHEFFTEGKELVLTLGRNESQWGTRVEDLPPSLSDYQF |
| 56 | Unigene0010217 | 255 | 87244 | 12 | MAHSLATVVLAAQLASAALVTEPLQYVDQLVGTQNGGNVFAGASLPYGMAKAVADTDSDSRQGGFTTDGANITGFSGMHDSGTGGNPSLGLFPLFPFSSCEGDEINGCEFPKKDRSEPYVNTSLKASPGYFSIDLQSGVRGEMTAAFHTALFQFTFPESGNGSPVIYLDLSDLSDSRQDNATISVEDSGRMTGYGRFEPSFGQEKYIAYFCADFKGGAVRDTGVVVNSRGSADVKDLKISRGINGFPLPGGGFVRFSDRTPVIARVGYSFISSEQACQLAESEIPDFDFRATQKSAVDAWTEKISVISVETNDVDEDTLKNFYSGVYRTFINPQNYTGVQPVVDANTIYFDSFYCIWDHFRSQFPFLVLVDPLAMEQIIQGLLTLYDIQGWLPDCHMSLSKGYTQGGSNADVVMADAYSKLNSTNIDWNKVYEAVVRDAEEEPYDWCCQGRGGLDSWKSLHYIPVEDFDYKGFGTHTRSISRTLEYSYNDYCISTIAQGLGKQGDVEKYQQTSGNWRNLFRADQESNLFNSSTSTGFTGFFQPKYLNQTWGRQDPLYCSNIDNSPTKSCSLQNTASETYESSIWEYSFYVPHDMAQLIATLGGPETFVRRLEYLHDQNITYIGNEPSFLTVYQYHYAGRPALSATRAHFYVPRFFSPTLGGLPGNDDSGTMGAFLAFTMMGLIPNPGQNVYFIIPPFFEEVRVKHPVTGNTAVIRNVNFDGGNYKNVYIQSATLNGEDYTRNWIGHEFFTEGKELVLTLGRNESQWGTRVEDLPPSLSDYQF |
| 57 | Unigene0002876 | 254 | 36354 | 28.5 | MFAARKVSQSVLGAAVQRRAFSATASNASKVVVLGAAGGIGQPLSLLLKLNPRVSELGLYDVRMAPGVAADVGHINTKSTVVGYEAGEAGLAAALKGANIVVIPAGVPRKPGMTRDDLFNTNASIVRDLAKAAAENCPDAHMLIISNPVNSTVPITAEVFKKAGVYNPKKLFGVTTLDVVRASRFISQIKNNDPANENITVVGGHSGETIVPLLSQAGHNLEGEERDAYVKRVQFGGDEVVKAKDGAGSATLSMAMAGARFTESLLKAAQGEKGVVEPTYVDSPLYKDQGVNYFASKVTLGPNGVEEIHPVGKVTEHEQKLLDVCLKDLKKNIEKGEQWVKENP |
| 58 | Unigene0008135 | 252 | 59036 | 11 | VFPNATATYFAPGVPTDAPIPGNYTSYLRPRVHFTPPRYFMNDPNGMHRSPDGTWHLYYQYNPITPVAGNQHWGHATSQDLYTWTNQKIPFFPPNDYTYLFSGSAVVDTNNTSGFFPDQDNGVVAIYTAAEYPNGQQGVQYQAIAYSRDGGYSFEAYEGNPVLNLNLTQFRDPHVIWHAATSRWVMTVAFATEFVIGIYTSPDLKEWEFASNFTSHGYLGVQYECPNLVQVPMRGQDEPVWFLLISINPGGPRGGSVSQYFPGEFNGTHFVPYDGATRLTDFGKDNYAAQFFYGTEAGQAPIALGWASNWQYTSLVPTGLIENGQFRSAMTVPRTFELANISMRGYDLVSAPYNIEAVLDNELAYNSSLGNGSVISYFADKVESGAIYLEANITGLTSTSRRGTANFTISSSVTGEYIRGGIKVAGEPGIWIDRGNLLGFSENPYFSDKFSEEGTFVADEGSWDLKVVYDNSIIEVFLNGAQAVGTLSVFPSRQLDTVAVFVGGIPANASSSVGIWGLKDTWAPQADGNGTVQGNVTQ |
| 59 | Unigene0004170 | 235 | 67122 | 8.5 | MRGILPVALLVCSASAIWPLPKEYQHGNDVLWINKDKVDVEYHQSGSVSLPEYPPEEYLLTHSEQNTKREEGTGSASKIVQNAVQRTYDTLFSKNFVPWMLRPRLSNYEPDTNSSGTYITTITIQQTGADPDDVKKPDTEIDESYSLEVTTDGKVTVTAKTSIGILWGLTTFTQLFFQHSNGGVYTDLAPVSIKDEPKFKWRGLNVDTSRTFKPLSDLYAMIDALSYNKMNRLHWHVTDAQSWPLEVPALPDLMPKGVYEPSQKYTVDDVRKLQEYGSLLGVEVAMEIDNPGHTSSIWFSDPDLIAAFNKQPDWTTYCAEPPCGSLKLNSTKVYDFLETLLDDLLPRLKPLTSYFHLGGDEVNKNTYLLDDTVQSNESSVLQPLMQKYMDRNMNQTKSYGFTPLVWEEMLLDWNLTLPKDTIIQAWQSDEAVAKITAQGYRVITGNYNYWYLDCGKGQWIDFAPSNAAGFWPFLDYCAPYHNWRAVYSYDPLHGVPENSTHLILGGETHIWSEQTDAVNFQQMVWPRTSAAAEVLWSGAKDAQGQNRSQIEASPRLAEMRERLVARGMRAEPVHMPFCTQNGTQCAYPS |
| 59 | Unigene0004169 | 235 | 65010 | 8.8 | MRGILPVALLVCSASAIWPLPKEYQHGNDVLWINKDKVDVEYHQSGSNTKREEGTGSASKIVQNAVQRTYDTLFSKNFVPWMLRPRLSNYEPDTNSSGTYITTITIQQTGADPDDVKKPDTEIDESYSLEVTTDGKVTVTAKTSIGILWGLTTFTQLFFQHSNGGVYTDLAPVSIKDEPKFKWRGLNVDTSRTFKPLSDLYAMIDALSYNKMNRLHWHVTDAQSWPLEVPALPDLMPKGVYEPSQKYTVDDVRKLQEYGSLLGVEVAMEIDNPGHTSSIWFSDPDLIAAFNKQPDWTTYCAEPPCGSLKLNSTKVYDFLETLLDDLLPRLKPLTSYFHLGGDEVNKNTYLLDDTVQSNESSVLQPLMQKYMDRNMNQTKSYGFTPLVWEEMLLDWNLTLPKDTIIQAWQSDEAVAKITAQGYRVITGNYNYWYLDCGKGQWIDFAPSNAAGFWPFLDYCAPYHNWRAVYSYDPLHGVPENSTHLILGGETHIWSEQTDAVNFQQMVWPRTSAAAEVLWSGAKDAQGQNRSQIEASPRLAEMRERLVARGMRAEPVHMPFCTQNGTQCAYPS |
| 60 | Unigene0007716 | 235 | 54869 | 13.8 | LLFLTASAAPQDAAAAAAPPSRPVVSSTNCNGKNYVYEELAGYGFVEDNARDRFGDTIGGIGSSIAVDRRSWVRLPGNRYTGLLYALPDRGWNTEGTLNYQNRIHKFLITFQPNTSASVSSPAPPNLQFRYLDTILLTDPAGNPAVGLDGGAMGPYLTYPGYNFAFPSANYTDDGFGGAGSGGYRLVIDAEGLALGIDGTYWISDEYGDYTYNFSPSGRMIGAIRPPDAFIPQRNGAQSFSSDNAPRYNPDLRPTPRNPTTGRANNQGLEGLSINPQGTKLYALAQSALRQDSGSGNPSTRNARFLVYDTSGRNRRAPRLEAEYVVQLNNVRPGDATSNVARQSEIHYISPTQFLVLARDSNAGRGAASTTSVYRQVDVFDISRATNIVGTYDSTTGSVAPGGVLLPNVTPAQYCSWLDYNVNSQLNRFGVRNGGDQSDGLLNEKWESLALLPVNPLNQGSDGEFYILSFSDNDFITQDGYLKGGEFRYSDAGGFSLENQALIFKVR |
| 61 | Unigene0003206 | 234 | 39568 | 24.2 | MAQSVLSTKAGVIYGDDVKNLFKYAQEKKFAIPAINVTSSSTVVASLEAARDAKSPLILQMSQGGAAYFAGKGVGNKNQEASIAGAVAAAHYIRAVAPSYGIPVVLHTDHCAKKLLPWLDGMLDEDEKFFKANGVPLFSSHMIDLSEESVKENIETTAAYLKRAAPMKQFLEMEIGITGGEEDGVNNEDVDNNSLYTQPEDIYEIYKTLAPISPYFSIAAGFGNVHGVYKPGNVKLRPELLQKHQKYVQEKEGTKDEKPVFLVFHGGSGSSKQDYLDAISYGVVKVNLDTDLQYAYTEGIRDYMISKKDYVSSQVGNPDGEDKPNKKYYDPRVWVREGEKTMSKRIQVALTDFNTANQL |
| 62 | Unigene0001041 | 234 | 78803 | 10.3 | MRFHRLLDFALLVTTLAVPAQQHRHDDTYKVEDHHHKHEGTKRKRQCCDNLNQPGIYDYIVVGSGPGGGPVASRLARAGFSVLLIEAGTDTSGYQDVQVPSFHPRASELPRQLWAYFVERYEDPAQQLRDTKQVYLVPDDPSYFGAPFTGAGHCPPDPWGITQLLPNGRYYTGQYPPPGATRLGLLYARGYALGGSTQMSAMAMVTPHEEDWVGLGEITGNYSTFDPHMMREYFVKLENCKYILNSVLGHGFTGWLDISATPLTLVLRDLKLASLIAATALAVGKAGGFLTQLITSISVFANILAADLNAPGLSRDYASDVWQVPNAVSPDRHSIRSGVIDLIRSTVQEGYPLYVQLDTMVTKVLMDTSGPIPRAYGVQYEYGPNLAYMSTLSTKQHGIPGYAYARYDVILSGGVFETPKLLKLSGIGPAEELSYWGIPVIANLPGVGTNMQDRYELGSVGLATTPFAAFKDCRYSVEPDPCLDEYRAGTNPVEQGPYVSNGLAVGTTLSTSVNDGPTPDIFIVAVPAYFAGYYPGYATCATLDALHWTFVILKMKSRNNAGYVRLRSLDPLDQAEIQFNNFAIGGDLDAQAVAEGIQRARDIYSSVIPLDGTLTEVVPGLEYQGAALIQYIRDNSWGHHACCTARLGAYGDPFAVVDFDFRVMGVSGLRIVDGSVWAKGIGYYPTVPLYMLSERAADVILGQQSVPLGLNDLNNFEA |
| 63 | Unigene0013187 | 234 | 47456 | 12.8 | MGISKIHARSVYDSRGNPTVEVDIVTETGLHRAIVPSGASTGSHEACELRDEDKSKWNGKGVTKAVANVNEKIAPALIKENLDVKDQSAVDAFLNKLDGTPNKTTLGANAILGVSMAVAKAAAAEKRVPLYAHISDLAGTKKPYVLPVPFMNVLNGGSHAGGRLAFQEFMVVPSEAPTFTEAMRQGAEVYQKLKTLAKKKYGQSAGNVGDEGGVAPDIQTAEEALDLITEAIEAAGYTGQIKIAMDVASSEFYKTEEKKYDLDFKNPDSDKSKWVTYEQLADQYRALAKKYPIVSIEDPFAEDDWEAWSYFYKNTDFQLVGDDLTVTNPTFIKKAIETKACNALLLKVNQIGTITEAIQAAKDAYGATWGVMVSHRSGETEDVTIADIAVGLRAGQIKTGAPARSERLAKLNQILRIEEELGSNAVYAGNNFRTAVNI |
| 64 | Unigene0001053 | 223 | 34250 | 22.5 | MHSSIARVALAGLSVLASITSAAPLEQRQANLRPVISTDFPDPSIIKDGDTWYAFASQSLYDFRDIKIQVATSKDFATWTLMEGYDALGTMAPWSRSDVWAPSVHRRDDGKYLMYYSGVTTTGGDGRFHCVGTAVADTILGPYQSNTDEPFACPIDQGGALDASYFRDGDRHFALYKIDANALGNGGSCGNTVEPILTTPIVIQEVQADGTTKIGEPITILTNSVYDGPLVEAPYMIRNADGVYVLFFSSWCYTTDKYSVSTAYSNSPTGPFEKAELPLFVTGTQGLIGPGGGSIADDGTSWAMHGYESRERVGGR |
| 65 | Unigene0010887 | 214 | 53552 | 19.8 | IVAILAFSGTGTCNVAASYKGPSTKGCASACTDLSKSLPGKVTTVANAAAFQQIQSGYYSTFNSDLAPACFVLPTTAQDVAAVVKAAKKAQCPFAVKGGGHTFFAGANSIQDGISIDLQQLNQVTVNKDGSASIGPGNRWGRVYETLDPLGLTVMGGRVSTVGVGGLLVSGGLSFLHPSQGLGVDNIRSYQLVNAEGKVLEVNQKNNPDLFWALRGGGNNFGIVTRFDVNTVKHQNFWGGGINYNISELEPLMKAYNRLAQPETQDPKATTWFAPVYFPKTGLWIINTEPVYAEPVSTTPEIFKPFMESPNQLSNSIRITNMSSLAQDTPPSGGNTVAEWDITMKMNTEVVHYMHQRIRDGAMNHPALDQVAFPVQILGNTTLSHTKNRGGNALGLSESDGPLLIVQLGLSWPAGTSAADDEAIYALGDALMRDVKQWSVKKGLEHRYIYMNYAGRSQNVFEGYGAKNHARLRQVAKKYDPDGVFQKLVPGGKKVF |
| 66 | Unigene0007855 | 210 | 22927 | 37.5 | MASLQRLLVSFMVILTFAFVFFAQSVDAQKGPKITHKVYFDITHGDEELGRIVIGLYGKTVPKTAENFRALATGEKGFGYEGSTFHRVIKDFMIQGGDFTNGDGTGGKSIYGNKFDDENFKLKHSKKGLLSMANSGKNTNGSQFFITTVLTSWLDGRHVVFGEVLEGMDIVHKIENAPKDGRDRPSQDVKIAKSGELEVPEEGIRAEL |
| 67 | Unigene0001502 | 206 | 17111 | 40.5 | MSANEQTFIAIKPDGVQRGLIGDIISRFEKRGFKLAAIKLVSPSQEHLEKHYEDLSSKPFFKGLVTYMASGPICAMIWEGRDVVKTGRVLLGATNPAASSPGTIRGDYAIDVGRNVCHGSDAVESAQKEIALWFKPEEIQSWKQAQHDWIYEK |
| 68 | Unigene0006628 | 202 | 110132 | 9.7 | PYPLIRRQNDTNSNCPGYRASDISTNANGLTAKLTLAGTPCNIYGKDIEDLTLTVEYQTDTRLHVLIEDAAQQVYQVPGSVFPRPASSGSPNTSSELVFEYIEEPFSFSVKRRSTDEVLFDSSAASLIFEDQYVRLRTSLPENPNLYGTGEHTDPFRLNTTDYTRTAWNRDAYGAPSGTNLYGTHPIYYDHRGTNGTHGVFLLNSNGMDFKIDTTGGQHLEYNIIGGVLDFYFLAGPSPVEVAQQYSDVSQKSALQPYWGLGFHQCKYGYRDVYWVAEVVANYSAAGIPLETMWTDIDYMHLRWVFTLDEDRFPLSLMRQLVDHLHANQQHYIVMVDPAVAYQDYDAFNNGVEQEVFMTRNNGSIYKGVVWPGVTAFPDWFHPNTQPYWNEEFQSFFDAETGVDIDALWIDMNEPANFCNYPCANPELEAQLAGNPPQPPPIRLGSPRPIPGFGPDFQPVCKSVVTFNVNASTFFGENILVFGSAITIGGGDDTAGAAPLGADNYPIWSAAIDLPANTEVTYQYIRSQPGGVFIYETENRTFTTGDCGSTSSTDDTITTSSPPQSKLVRRLSEFSYKTHHLTPIFKRQVAGDMKGLPGRDLINPAYKIDNEAGSISNKTADTDIIQYGGWTQYDTHNIYGSQMSEASRISMLARRPGLRPLIITRSTFAGAGAHVGKWLGDNLSNWQNYLISISQNLEFAAMYNVPLVGPDVCGFGGDTTELLCARWAVLGAFTPFYRNHAQNDASDQEFYRWPLVTEAAKKAIDIRYRLLDYFYTAFYVQNQTGTPVIQPMFYHYPEDSNTFGLGYQYFYGPGILVAPVTTENSTTATHYLPDDVFYDFYTHDKIIGTGETVTVEDVDYTEIPLYYKGGSIIAQRSESANTTTELRKKDFDIIIAPSANGTAYGELYLDDGVSLVQDTWSYMKFEYFSNGRFTITGRFGYDTDVFIQSITVLGLGSGSGSTGASRTKVVNNRISLTQEYSGIV |
| 69 | Unigene0006824 | 201 | 42744 | 22.6 | YNIVVFGGMYTKSIITILPCPLIKYAGDHCGPEVSSSENQLKSVLLTIAHUVTAEALKILDVVDKSNADIHFNIQEHPLGGASIDAHGEPLTDAALAAAKAADAVILGAIGGPKWGTGKVRPEQGILKLRKEMGTYGNLRPCFFASESLVKSSPLKEEVCRGVNFNIVRELTGGIYFGDRVEDDGSGFALDTEPYSRAEIERVTRLAAHLALAEDPPAPVWSLDKANVMATSRLWRKTVTEVMEKEFPQLKLGHHLIDSAAMLMVKNPRALNGVIVTSNLFGDIISDEASVIPGSLGLLPSASLTASPDGKSKCNGIYEPIHGSAPDISGQGIVNPVAMLLSLGMMFKYSLQQPALAQKIDEAVRNVIESGVRTKDIGGSSKTSEVGDAVAKELAALLK |
| 69 | Unigene0006826 | 201 | 36366 | 26.4 | ILDVVDKSNADIHFNIQEHPLGGASIDAHGEPLTDAALAAAKAADAVILGAIGGPKWGTGKVRPEQGILKLRKEMGTYGNLRPCFFASESLVKSSPLKEEVCRGVNFNIVRELTGGIYFGDRVEDDGSGFALDTEPYSRAEIERVTRLAAHLALAEDPPAPVWSLDKANVMATSRLWRKTVTEVMEKEFPQLKLGHHLIDSAAMLMVKNPRALNGVIVTSNLFGDIISDEASVIPGSLGLLPSASLTASPDGKSKCNGIYEPIHGSAPDISGQGIVNPVAMLLSLGMMFKYSLQQPALAQKIDEAVRNVIESGVRTKDIGGSSKTSEVGDAVAKELAALLK |
| 69 | Unigene0006825 | 201 | 38821 | 24.7 | PTYNIVVFGGDHCGPEVTAEALKILDVVDKSNADIHFNIQEHPLGGASIDAHGEPLTDAALAAAKAADAVILGAIGGPKWGTGKVRPEQGILKLRKEMGTYGNLRPCFFASESLVKSSPLKEEVCRGVNFNIVRELTGGIYFGDRVEDDGSGFALDTEPYSRAEIERVTRLAAHLALAEDPPAPVWSLDKANVMATSRLWRKTVTEVMEKEFPQLKLGHHLIDSAAMLMVKNPRALNGVIVTSNLFGDIISDEASVIPGSLGLLPSASLTASPDGKSKCNGIYEPIHGSAPDISGQGIVNPVAMLLSLGMMFKYSLQQPALAQKIDEAVRNVIESGVRTKDIGGSSKTSEVGDAVAKELAALLK |
| 70 | Unigene0012375 | 193 | 79628 | 13.3 | MSGETFEFQAEISQLLSLIINTVYSNKEIFLRELISNASDALDKIRYEALSDPSKLDSAKDLRIDIIPDKENKTLTIRDTGIGMTKADLVNNLGTIARSGTKQFMEALSAGADVSMIGQFGVGFYSAYLVADRVTVTSKHNDDEQYTWESSAGGTFTIVQDTEGEQLGRGTQIVLHLKDEQTDYLGESKIKEVVKKHSEFISYPIYLHVLKETEKEVPDEDAEETKEEDSDNKPKVEEVDDDEEEKKEKKTKKVKESKIEEEELNKTKPIWTRNPQDISQEEYGAFYKSLSNDWEDHLAVKHFSVEGQLEFRAILFVPKRAPFDLFETKKTKNNIKLYVRRVFITDDATDLIPEWLSFVKGVVDSEDLPLNLSRETLQQNKIMKVIKKNIVKKTLELFQEIAEDKENFDKFYTAFSKNIKLGIHEDSQNRSALAKLLRYQSTKSGEETTSLQDYVTRMPEHQKQMYYITGETIKAVEKSPFLDALKAKNFEVLFLVDPIDEYAFTQLKEFEGKKLVDITKDFELEETEDEKKKREEEEKEFESLAKSLKNVLGDKVEKVVVSHKLVGAPCAIRTGQFGWSANMERIMKAQALRDTSMSSYMSSKKTFEISPANPIIKELKKKVETDGESDRTVKSITTLLYETSLLVSGFTIDEPADFAERIHKLVSLGLNVDEDVETEAEAAAPAPTEGATESAMEEVD |
| 71 | Unigene0000170 | 193 | 60603 | 10.6 | MRTDLPAVFALSVASANAFSLPWKRQAGSDNPYVPTSTSCPSSASVRVADSLNPDEESYAQERLSRASDDLSSWLGRTLDGFTSDRLPKLALALSGGGTKAGLTTAGAVYGLDGRESSDSPVAGLLQGMTYVSALSGGSLTLSGIMSNNFAQVSTLRDELLEESYQNVFPAPLANADAIRADVGNKTAAGYPASLIDIYGKVISYNFISATGGNSLHWSNVTSQSSFQDHSAPYPIITITQVNVAAGMCSPNETSAIWEVAPLEFGSWDSNVDAFYPTQYMGSVAGNSSQCTTGFDNTGFITAISSNILIDDTCSTGNESTDSLNSLAGTVNTAFSGVSLVNPYGIVPNPFYQSSEAGPIRDQQVLFGTDGGDSGQAIPVFPFLQPERNIDVIVLVDAATQQPGNITNGTWLYNTYTSAQEKNLTRMPQVPTPSEFVAASLSSRAQFYGCNDNNTATLIYLPNTELGDAPTGFQQTAEQVNATFEGGVAMVTQSQQNSTEAQEWAACLACAIVHKSVEAADLPEVCTGCLERYCWPRSNETEGGGSGGNGDGHGTDSGSGSGSGSENGAAGLILGA |
| 72 | Unigene0007616 | 188 | 22426 | 19.2 | MLFSAATTAIALLASTVAAVPASLTIMADNPNLEITQLVVNSSATGNTLLQFNVKNPEPLAAKGTAVCRGEWDSVKKDWPISGKALRCDKSQSVTWYIDSWTDATNFVIAIQDRFKDPSIGEPPHDRMTTFSKATINESFVSYNHKNEKPDASQDTTAGTNAGTKAGTGTNAGAGAGGSANAPVDTQAEGSSDFVSGTQMPDVTIYAAVYAVSA |
| 73 | Unigene0002664 | 188 | 34629 | 16.9 | MVKAVVAGASGGIGQPLSLLLKASPLVDHLSLYDVVNTPGVTADLSHISSIATIDGYLPADDGLKKALTGADIVVIPAGIPRKPGMTRDDLFKINAGIVQGLVQGIAETCPDAFILIISNPVNSTVPIAAEVLKKAGKFNPKKLFGVTTLDVVRAETFVQALTGTKDPAKTVIPVIGGHSGETIVPLFSLAKPAVNIPADKLDALTNRVQFGGDEVVKAKDGAGSATLSMAYAGFRFAEKVIKAAKGESGVVEPTFVYLPGVNGGDEIHKETGLEYFSVPVELGKDGAQKAVNVLKDVNDYEKKLLTKCYEGLKGNIEKGVDFVHNPPAKS |
| 74 | Unigene0000807 | 186 | 68972 | 10.7 | MEKVDTTERLKALRSLMSQHKVDLYIVPSADAHSSEYIAPTDARREYISGFSGSAGTAIITQTKAALATDGRYFNQASQQLDSNWDLLKQGLQDVPTWQEWTVEQADGGKTVGVDPTTITATESRKLAEKIKKKGGKELVAVAENLIDKVWAKEKPARPNEPVRVLGLEYAGKKYQEKIEELRKELDKKKAAGFVVSMLDEVAWLFNLRGNDIPYNPVFFSYAVVTPTTTTLYIDESKLPDDAKSHLSGVHVRPYEDIFSEIATLAAEQSTSVNGGEESAPKKKFLISTAASWALQRSLGGEENVDEVRSPIGDAKAIKNATELEGMRQCHIRDGAALIEYFAWLEHQLIAEKAEIDEVAAADKLEAIRSKHHKFVGLSFDTISSTGPNAAIIHYKPEPGSCSVIDPAKIYLCDSGAQYLDGTTDTTRTLHFGTPDPKEIEAYTLVLKGNIGLELVVFPKGTSGFALDMLARQFLWQNGLEYRHGTGHGIGSFLNVHEGPIGIGQRIQYSEVPLSVGNVISDEPGYYEDGNFGIRIENVIMVKEVETKYKFGDKPYFGFEHVTMVPLCRKLVDVSLLTQKELDYINAYHKEVWEKTSPFFENDEKTLKWLERECQPY |
| 75 | Unigene0008496 | 181 | 53347 | 13.7 | LTYLLRLVPTVLAQFGFTEESDSFIVNSGGNSSLVTTIRKSDCDVRSLVYRGTELQGPQDQGTHIGSGLGSAEVTAEAVGDKYVKVTCNAGNLTQYIVVVSGQSNLYMATHITAEPQVGELRYLARLDSSKLPNEYPFGNGSHTAGSTETIEGSDVFIVDGETRSKFYSSQRFIDDHVHCVAGPDTHACFVKPQYESSSGGPFFRDINSNNGGVYTSLSFYMNSNHAQTEKYRMGLHGPYALTFTRSGIPKLEDFDFSFFADLDIEGYVPESGRGYVAGAVSGIPDDDQAVLHWFNTEAQYWTYAEDGDFTSPAMKPGTYTMKLYQTELEVASQNVTVTAGSETSSDIASALKEPATRLWTIGKCDGRPTGFRNADKQLRMHPSDLRMESWGPLTYTVGSSSDNDMAMALFKAVGSPQTIAFDLEDAGAATLRIRTTLAFASGRPQVKVNEWSAPAPAAPARIDSRGVTRGAYRGLGEAYEFEIPQGTL |
| 75 | Unigene0008495 | 181 | 53347 | 13.7 | LTYLLRLVPTVLAQFGFTEESDSFIVNSGGNSSLVTTIRKSDCDVRSLVYRGTELQGPQDQGTHIGSGLGSAEVTAEAVGDKYVKVTCNAGNLTQYIVVVSGQSNLYMATHITAEPQVGELRYLARLDSSKLPNEYPFGNGSHTAGSTETIEGSDVFIVDGETRSKFYSSQRFIDDHVHCVAGPDTHACFVKPQYESSSGGPFFRDINSNNGGVYTSLSFYMNSNHAQTEKYRMGLHGPYALTFTRSGIPKLEDFDFSFFADLDIEGYVPESGRGYVAGAVSGIPDDDQAVLHWFNTEAQYWTYAEDGDFTSPAMKPGTYTMKLYQTELEVASQNVTVTAGSETSSDIASALKEPATRLWTIGKCDGRPTGFRNADKQLRMHPSDLRMESWGPLTYTVGSSSDNDMAMALFKAVGSPQTIAFDLEDAGAATLRIRTTLAFASGRPQVKVNEWSAPAPAAPARIDSRGVTRGAYRGLGEAYEFEIPQGTL |
| 76 | Unigene0007427 | 178 | 85626 | 10.8 | RSLALLATALSGLLSFSPAAQVAAAPAAQADSAATAASSYWLGTISREGGTVWGNNDTDYKIFRNVKDFGAKGDGVTDDTEAINTAISSGKRCGQDCESSTILPAIVYLPPGVYKVSKPIIMYYYTQLIGDAVEKPTIQSTPDFAGMAVLDSDPYDDTGKNWFINQNNFFRQVRNLKLDLTQTKAGTGIHWQVAQATSLQNIDFIMTESADSEQQGIFMDNGSGGWFSDLTFTGGKFGAFLGSQQFTSRNLVFKNCRTALYMNWNWGWTLSNVSISGSNVGLDMSANPQNLSTGSVLLSDSIIQNTEYGVVSAFSVSENVPFNGNTLVLDNVDMSGVEKAAVWSSKDNQTILAPGKISSWVQGTSHVNSATGNRTQSATSTLKKSALLVDSDGKIYGRSKPQYENVPASNFKSAKAAGCKGDGETDDTQCIRDLFESVASDPNAIAYFDHGAYLIKDTITVPTSIKITGEIWPLLVADGESFNDAENPKPVFQIGARNGDGKGEVEITELMFETKGAAPGAIMIEWNLNSDPLKSGMWDSHVRIGGSYGTQLSEAECPGWPTTEPTDKCQGVFLMFHARPESGGLYLENTWFWVADHDLEVKNQTQISIYSARGTLIQSQGPAWLWGSASEHSVIYNYQIDGAKAVFGGFMQTETPYFQPNPEATEPFSVNTEYHDPDFSNCNATGTPGVPCADAWGLIVKDTESLLVYAVGMYSFFNNYQQACVPLHNCQENMIKIQNSTVDMYTVTTKAAVNMILDDSIQGSVLGSDNRNVFGDTIAYYHSGSS |
| 76 | Unigene0007426 | 178 | 85626 | 10.8 | RSLALLATALSGLLSFSPAAQVAAAPAAQADSAATAASSYWLGTISREGGTVWGNNDTDYKIFRNVKDFGAKGDGVTDDTEAINTAISSGKRCGQDCESSTILPAIVYLPPGVYKVSKPIIMYYYTQLIGDAVEKPTIQSTPDFAGMAVLDSDPYDDTGKNWFINQNNFFRQVRNLKLDLTQTKAGTGIHWQVAQATSLQNIDFIMTESADSEQQGIFMDNGSGGWFSDLTFTGGKFGAFLGSQQFTSRNLVFKNCRTALYMNWNWGWTLSNVSISGSNVGLDMSANPQNLSTGSVLLSDSIIQNTEYGVVSAFSVSENVPFNGNTLVLDNVDMSGVEKAAVWSSKDNQTILAPGKISSWVQGTSHVNSATGNRTQSATSTLKKSALLVDSDGKIYGRSKPQYENVPASNFKSAKAAGCKGDGETDDTQCIRDLFESVASDPNAIAYFDHGAYLIKDTITVPTSIKITGEIWPLLVADGESFNDAENPKPVFQIGARNGDGKGEVEITELMFETKGAAPGAIMIEWNLNSDPLKSGMWDSHVRIGGSYGTQLSEAECPGWPTTEPTDKCQGVFLMFHARPESGGLYLENTWFWVADHDLEVKNQTQISIYSARGTLIQSQGPAWLWGSASEHSVIYNYQIDGAKAVFGGFMQTETPYFQPNPEATEPFSVNTEYHDPDFSNCNATGTPGVPCADAWGLIVKDTESLLVYAVGMYSFFNNYQQACVPLHNCQENMIKIQNSTVDMYTVTTKAAVNMILDDSIQGSVLGSDNRNVFGDTIAYYHSGSS |
| 77 | Unigene0002265 | 175 | 94844 | 7.6 | MVLPRAALLALLPAVCAQLQPFANNTGENLDFAESPPKYPSPWSEGLGDWREAYEKAVAFVSQLTLLEKVNLTTGVGWEGEKCVGNTGAIPRLGFRSLCLQDSPLGIRFADFASAYPAGVTIAATWSRELFYKRGHDMGSEHRDKGIDVQLGPAIGPLGRNAAGGRNWEGFSPDPSLTGVAVYETVKGIQDAGVIACTKHYIANEQEHFRQGGPNLTESISSNLDDVTMHELYLWPFADAVRAGTGSIMCSYNQVNNSYACQNSYLQNYLLKSELGFQGFIVSDWAAQHSGVSSALAGLDMTMPGDVAFDSTTSYWGPNLTIALLNGTVPQWRLDDAAVRIVAAWYYVDRPGNQVENAPSFSSWTQDTFGFQHFYAQQNYTQLNYHVDVRAEHAKDIREFAAKGTVLLKNKGALPLTGKEKLTAIFGSDAAESQYGPNGCPDRGCDNGTLAMGWGSGTAQFPYLITPLEAIKAEVRSNDGAVESVIDDYAYTQINALARRVPDNNGVCLVFANSDAGEGYIIVDGNEGDRNNLTLWHNGDDLIRNVTSFCNNTVVVLHTVGEVTVTPWFDNENITAIVWAGIPGQESGNAITDILYGKVNPGGKTPFSWARQREDYGTDVLYEPNNGEDAPQDSFEEGVFIDYRALDKYDRTPIYEFGFGLSYTTFSFSDLQVQAHQVAPYTPTTRNTPPAPTFGTIANNTEEYVLPANFSGVYAYIYPYINSTNLEESSTDPDYGTDYSWPEGSYDSSAQPYAPAGGAPGGNPQLYDVLFTVTATVTNTGSVIGDEVPQLYVALGGPNDPKVQLRDFDRLTIEAGASATFTGNITRRDLSNWDTASQNWVISDYTKTVYVGNSSRRLPLSAVLNL |
| 78 | Unigene0004020 | 174 | 55495 | 14.9 | GSDYTGLNPPQDAKIKGVLYPRAGTLGGCTAHNALITIYPNEVDWEHLVDVTGDSSWAPDNMRGYFTKLEHNKYLPTSIVGHGYSGWLPTSLTSLTLILQDVKIISLVVAAATAIGQSLLSSVLTTVTEVAHVLLTDINAPGKERDGSEGVYQVPLAMQVPGYKRAGPVDLLNQVISATNSDGSRTYKLDIALNTLVTKINLDESGSTPKATGVDFLAGQYLYKADPRRQSGSATDSGTPGTVKATREVIVSAGAFNTPQLLKLSGIGPRAELESYGIDVKVDLPGVGKNLQDRYEVAVAGRAPTPINLINDCTFLESSPDPCLVNWESRAIEKGVYSTNGVALAVLKKSSVAENDTSDLFIAGWPAHFVGYYPNYFQEAVAGRNAWSWVPLKAHTRNSNVGTVLLNSADPTDMPSINFRYFENGADEDLQALVEGQKYGRKVFEDLIPLDGKFTEDWPGEKVQTDEEWKQFIKDEAWGHHACCTAKIGSDDDSDAVLDSNFKVRGVDGLR |
| 79 | Unigene0006612 | 171 | 66043 | 6 | MKASYSILPLIGSVLSFPAADTLEARATGSLESWLAAESPVALQGILNNIGSQGNKVQGAYPGIVVASPSRNDPPYFYTWTRDAGLTIKCLVDQFLAGQTNLEGTIQDYINSQLSLQTVNNPSGGLCSGGLGEPKFEVDGTQFTGDWGRPQRDGPALRATAMIAYARHLLSKGQTSQVRDIIWPIVSNDLTYVASNWNATGFDLWEEVNSSSFFTTASQYRALVEGAALASQIGQSCAHCSSQAPQILCFLQSYWTGSYVRSNTGGGRTGKDVNSILTSIHLFDSAVGCNANTFQPCSDKALANHKQVTDSFRSVYSINSGIPPGKGIAVGRYSEDVYQGGNPWYLATFAAAEQLYYAVYQWRQQGSLTITDLSLPFFRDVYPSAATSTYASSTTTFTSVVNAVLAYADSYFANAQQYTPQNGALAEQYSRSNGSPLSAVDLTWSYAAFLTALNARKAVLPSSWGAGSAQVPNSCSGGSANGPCVAATNTFSRPGSTPTSTTGACSATPTRTNVLFKETVTTNFGESVFITGGIAELGNWDTNNAVALSAQNYTSTNNLWFATVSLPARGGFNYKYIRKQQDGSVRWESDPNRYYVVPANCAGSATQSDTWR |
| 79 | Unigene0006611 | 171 | 61784 | 6.5 | SPVGILNNIGSQGNKVQGAYPGIVVASPSRNDPPYFYTWTRDAGLTIKCLVDQFLAGQTNLEGTIQDYINSQLSLQTVNNPSGGLCSGGLGEPKFEVDGTQFTGDWGRPQRDGPALRATAMIAYARHLLSKGQTSQVRDIIWPIVSNDLTYVASNWNATGFDLWEEVNSSSFFTTASQYRALVEGAALASQIGQSCAHCSSQAPQILCFLQSYWTGSYVRSNTGGGRTGKDVNSILTSIHLFDSAVGCNANTFQPCSDKALANHKQVTDSFRSVYSINSGIPPGKGIAVGRYSEDVYQGGNPWYLATFAAAEQLYYAVYQWRQQGSLTITDLSLPFFRDVYPSAATSTYASSTTTFTSVVNAVLAYADSYFANAQQYTPQNGALAEQYSRSNGSPLSAVDLTWSYAAFLTALNARKAVLPSSWGAGSAQVPNSCSGGSANGPCVAATNTFSRPGSTPTSTTGACSATPTRTNVLFKETVTTNFGESVFITGGIAELGNWDTNNAVALSAQNYTSTNNLWFATVSLPARGGFNYKYIRKQQDGSVRWESDPNRYYVVPANCAGSATQSDTWR |
| 80 | Unigene0002750 | 171 | 57628 | 11.3 | MALKYILPSVSYLTAVLAAENLTCPDYSVYAKERHEPYSEGTYQLSYQRPVTRCRTFNSSVVEEAIERIQGEIADPDLKRLFENAFPNTLDAAVKWKGTAAGSDEELTFLITGDIDAMWLRDSANQVQSYLPLLKASSESNSLASLFRGVINLQARYLLFDPYCNSFQPPVESGLNASFNEYAAEDIVLPKYDNTTVFECKYELDSLAAFLQVSYDYYKATQDAEFFGKYQWLKAVEAVLKTAEEQMVGSYEPDGQVYNLTYQFQRPTTRSTETQANNGLANPFNNGTGLVRSAFRPADDATIYQGLIPSNMQFSRYLAATADIAAKISGQEALAQKMRDLSECIRNAISNYGIVEIPGYGKVYAFEVDGYWGQNIMDDANIPSLLSAPFLGYVDASDEVYQNTRKLILSSANPYFMRGPVINGIGGPHVGPGYAWPMASIVRILTSSDDAEITEQLREIVSSTDRLGLIHESINTFNASDWTRQWFSWANGLFGQAILDLSDRKADILKQSFQ |
| 81 | Unigene0007335 | 170 | 19513 | 24.6 | MLFKSVLAAAACFGFVTAEPGCNNCTNESPDPFVDVPAPWNLKGDIYTFFLLPGLGIPLDNQLPKKAFPPLERQFPSAIEGDYKGTLGMVQIVRYTESPVGPYDEMFIVPGFFEYDRDGGKKRGVRVSRIYVSQKYPVWTSRITWNQPKHLARFEWQDRSDGSTSVKIYPF |
| 82 | Unigene0009071 | 170 | 10179 | 44.8 | NQSFCASQFAGAAEVQPYEGSGKTTSVAPDVYFANLEKNCPAATPTPGDDGTKPSDKVCQAYVGLRNCQGQPSPNQSFCASQFAGAAEVQPYEGSG |
| 83 | Unigene0012678 | 169 | 42247 | 12.6 | MRIPSFLFALLSATSIVSAVSPKRPYFYKGHDLSSLKILEEGGAVYKDTARGNATRPAEDILGDGGMNTVRLRLWVDPTVPYDGGYYQSYDLEYVLSIAKRFHKKGYKIYLDYHFSDYWADPQKQWQPVAWPTTLKPLAKTLRGYVSESLQAFHKAGVDLALVSLGNEIRNGMVWPLGRVSVTVEPESARIANFTGLATLYKAARNGVDDAVKAGLRKPQVMIHIDNGYDLSLQQRWFGALVGTGIVKTSDWDIFGFSIYPFYGTSATLANLKTTVNWVANKYKKPIQVVETDWPAICTGEGVPTLSEPSIPASVDGQLEWVGKVIDVVKEIPRGLGQGIHYWEPAWLNNTGLGSACQDAILFDADWSTFPNVTAYSRKS |
| 84 | Unigene0013498 | 168 | 41237 | 11.8 | MRFEPLLALGATASATTLFVADYSGNITTLSLAESNGNYSLTKTSASDGCAPNPAWLTVDPGRGLLYCTNEGLSSPNGSLASFSINADGSLDSIVNLTTANGPVSAAIYGNPSAGERGLVLAHYSGSAVTTYKVSSTGNVTLNEEFFFTIPSPGPDPDRQDAPHPHEAFVDPTGQYILVPDLGADLVRIFSFDVETLKLTELDPLEAEPGSGPRHLAFWTPYNTPGGTTYLYLVGELTATVTGYAVQYSANGTGLSFEDIGYLKTTGLFNVPQRVAPAEIQVSPDNRFLIVSNRNDTTFTLPNAIGKSDSIATFPLGQDGNLTALHQLWPAGGNFPRHFETNAAGNLVAVGLQNDQAVAILRRDVTTGLIGEPIARIEIGGNVTTVVWDE |
| 85 | Unigene0009305 | 166 | 30324 | 27.1 | PNLKHEHAGDVFRKLHRKQCYLDGPASNGSSTGEYRTVEGVDLYIAHPPSRHHKKTKTTPPAIIYLTDIFGVQLLNNRLLADAFSKAGYLTIIPDLFRGDPVPANALSDPNSNFNISEWQTRHPASEIESIITSTLRTTTTEYGIPSSKIGAVGYCFGGKYVAKYLSSNSTRGNTTFAAGFTAHPSNTLPSEWEAIENPISIAFGELDASSTPENRTNIEGIFIQGNKTFQTNLYADAEHGFAVRTNLTDPKKRFAQESAYLQAVRWFDQWVK |
| 86 | Unigene0007804 | 166 | 5907 | 64.8 | VTNGGAVCDAAGGDGSSWYTDSKGNICCPFPCNPKDGDPMCKDANTNNQKKPDC |
| 87 | Unigene0000979 | 165 | 37637 | 13.8 | TDTDGKPIQAHGGNIIQAQNGDGSWYWFGEDKSSNNGRFNGVSCYKSSDLSTWTNEGHALSPIANTNISDSRVVERPKVLYNDKNAEYVMWFHGDSSNYGDAQVGVATSKTIDGEYEWKGNFKPFGNDSRDMTIWKDPDDGSAYLIFATSNNADFQIATLDDDYYNVKEAIYTFRGVFQEAPGVFKIDGKFYLIFSPQDGWTPTDNGYHVADSMSGPWSDATLLHPKGAYAYLTQNAYDITIKGSEQTFYLYLGDHWNANNLGASTYAFYPVLYDGSGLVLHPTGGWTLDIAKGTWADLPFTTITADDSTTPKDQLVQCEDSCAGGLAANMTSTENSFSF |
| 88 | Unigene0000262 | 165 | 15213 | 21.5 | IETGMFMYKDYNQETDIEWISDPESFANQQFSSNGSRVLLYTNQGPSGNTDALTVSGRAPADATSAVHEYRLDWTEDAVEFYLDGVKQQTLTDYVPTVGGSWLWNSWVNGDPYFTAGPPVNDAVFKILRIEMDYN |
| 89 | Unigene0010418 | 162 | 24373 | 17.2 | MFSRASSTTAFTKALPTFRLQPTSNRSFHASAAIMVQTYFTVEWTGRLVTIDKANNRVVVEPEEKQRKKTIKFNLFDKDVPKTAENFRQLCTGEKGENFGYKGSKFHRVIPQFMLQGGDFTRGNGTGGKSIYGEKFADENFKLSHTKPFLLSMANAGPNTNGSQFFITTVVTSWLDGRHVVFGEVVPGDEESIQAIASIEDRGSSSGALKGGLPVTIVDCG |
| 90 | Unigene0010023 | 160 | 40572 | 12.6 | KCRGVNLGGLFVLEPWITPSIFEATPENVVDEYTFCETLGKDEAKRRLEEHWSTFYTEEDFALMKQYGLNTVRVPIGYWAVAPVEGDPYVQGAYEHLPDIVQWAGNNGLTVMIDLHGAPLSQNGFDNSGKRGPVGWTQGDSVQQTRNALDRIRDDYANNPTVSSIELLNEPMGPQLDMNVVRQFYYDGWGSLRDSPVAVVFHDAFMGVTSWNEFGAGMQNLVLDTHHYEVFSSGELQMSPGQHISTACGFGGQMASTNKWTIAGEWSGAQTDCAKYLNGRGVGARYDGTFNKDGQGSSYIGSCDRKTSGSVGGLSGDEKNNIKQFIAAQIAAYEKAAGWIFWCWKNEAAPEWHFRDLVAGGLVPQ |
| 91 | Unigene0000999 | 160 | 20367 | 26.7 | MAQSARQMMGAPPSTASPSDSTLVIIDAQNEYAEGALKVTDAPGTRKIIASLLEKYRAAGGQIVHVVHNVPEGTPIFTPGTNLAKEFEELTPKDGEKVIEKIHPSSFADTTLHDYLRGAGAKKVVLTGYMAHVCVSTTARDAARLGYDVLVVEDGVGDRDIPGASGAEVTKMVMHELADFFATIVQSKDIQ |
| 91 | Unigene0001000 | 160 | 20367 | 26.7 | MAQSARQMMGAPPSTASPSDSTLVIIDAQNEYAEGALKVTDAPGTRKIIASLLEKYRAAGGQIVHVVHNVPEGTPIFTPGTNLAKEFEELTPKDGEKVIEKIHPSSFADTTLHDYLRGAGAKKVVLTGYMAHVCVSTTARDAARLGYDVLVVEDGVGDRDIPGASGAEVTKMVMHELADFFATIVQSKDIQ |
| 92 | Unigene0010237 | 156 | 18823 | 5.6 | MLLGLFISITLAYFSNAQSCGSVTCGAHEQTAQIGNLLAFDNLEGLPMQAGQHVVVGWPAQEGAGLTMAYGNPTNVPIAVTVNSITHDTGVSFVAEPGVGYGTVQLDANRNLYLGHGVEILLTYVPTTPPLRNHRVATKNGRLAKHVRPVGSSLRKVPTGPSCDGKCNSKEGHLPGY |
| 93 | Unigene0010037 | 154 | 18763 | 14.5 | MRSASATLLAVASTFFAGANACLYFNATLTDTKLSVYSWDDANNNNATNPEDIICQGQNLDQESDGYWSVPCARADNATSDTVFDVFLNKDNTRFLDVIYKYSNPEAAGLQQGSGDPNPDEFYFNFRVGSVDGKFFKSREFCAQYDLFRLLKPEALKNPCDSLDSC |
| 94 | Unigene0011783 | 154 | 13036 | 9.6 | PGAFEVSRFHYVCAIGEPRYCRWSFDLAVSGSAPNHPALPGPYYCEGSSNNTFFTPCSSVSETQELLAYIPENNVVQLQYTALNYNNGVTYAYFGEYQTQPNNPYQATNFTVAES |
| 95 | Unigene0002935 | 153 | 60034 | 11.5 | LAGLLALGSSQLASALSPTVSLDYATYAGSALSNGITQWLGVRYAAPPIGPLRFSEPQDPIDQTSNGTIPATEHGKICLPTGDLDDNDFTFPNGNYSEDCLFLDVFAPSNATKDSKLPVFFFISGGGFNQLSNAKYNGSGIIQAAEYDAVVVLFNYRVGPYGFLASKEIVEGGAGINNGLKDQRKALQWVQKYIPHFGGNPEHVIVGGASAGAQSVSLQVTAYGGRDDGLFVGTAAESQSFPPVRTIEESQFAYDNLVIRTNCTAQWNDTLSCLRNLSAEELQKVNYNTPYPGAQEAALYMYGPTLDFDFIADYTTRAYVEGKFVRVPAIYGDDTNEGTGFAPKNTTSYGQTSTFLQNNFPELTIQQLGKINELYPVEDTPSFNGSGRYWRQAADAYGELRYTCPGILVSASLERYNVPSWNYHWNVVDPVAEAAGEGVEHTVEVHAIFGPENTRGGAPDSYRVGGVNEGVTAVIQGYWTSFIRTLDPNKKRVTGTPKWEEWTGTALNGQDGKRLRFERNLNATEMESPPAIQQERCAYFSSIGLSVKQ |
| 96 | Unigene0000795 | 153 | 23238 | 22 | TEWICHCKSGQNNGVDQKTGFPADVSNCDSYPGFAGSVHFPHCWNGKMPKVNDNSHMAYPEGDVQSGPCPSSHPTRLPHIFLENFYDLAKVADKIKPNSFSLSQGDPTGYGFHADFFNGWDEGAIPKLFDDCPQPYYGNSDVGTCPSFQGFDTKNTDCKLTIEFKENVDTPGKYLPGCNPISSDNPAQKMKPAPLGVCSDECTPAGGAS |
| 97 | Unigene0002081 | 151 | 81932 | 7.8 | MSVVGLDFGTQNSVIAVARNKGVDVITNEVSNRATPTLVSFGSKARFLGEAAKTQEISNLKNTVGSLTRLAGRTINDPDVQIEQEFVSAPLVDVDGQVGAEVTYLGQKQKFTATQLIAMFLTKARETASKELRLPVNDMVIAVPAWYTDAQRRGILDAAEVAGLKVLRLINETTATALGYGITKLDLPGPEEKPRRVAIIDIGHSNYTCSICEFRKGELKVVSTAYDRHFGGRNFDKVLIEHFRNEFKEKNKIDIYENPKARVRVAAAVEKLKKVLSANAMAPINIESLMNDVDVRGMLKREELETLVQPLLDRAHIPLEQALADAKLTKDDIDYIELVGGCTRVPALKSIIQNFFGKNLNFTMNADEAIARGCAFSCAILSPVFRVRDFSVQDIVQYPIEFTWEKSPDIPDEDTNLTVFNKGNALPSTKILTFYRKQPFDLEAKYAKPDALPGKPNPWIGRFSVKGVKADGKDDFMICKLKARLNLHGVLNVEQGYYVEEQEIEEPIPEAKDGDAMDTDKANGEATPPVKTRKVKKQVRKGDLPLSAGTASLDQQQKDLFLEKEFQMIAEDKLVAETEDRKNELESQIYSMRGKMDEPYTSNGYADFASDEEKDKIRAKCDELEDWLYEDGEDATKAQYIAKYEELRSTAAVVISRFNEKRQEEEEARRKVEEEAAAKRRAEEEAKRAADAEKKKVEEEAKKAAAEAKPDEEMKDADAADGEKPADVEEA |
| 98 | Unigene0013769 | 151 | 40320 | 6.7 | FRYIVPALAVAGSAFAQCGEEGATTTIQSAADATGLSGCSTYSGSIAISTSTTDNIDLAGISRIDGSLIADNVTGIVQLGASDLEEIGERFDLNGLTILSTLSFPRLAKVDTIAWIALPALQGLTFSQTGVQEVNMLQIDNTELGSLDGINLQVADTIYIANNRFLNSVNMQLGNVTEAMTFTNNGEDLVAEFPNLEWANNITIRNLSSVSMPSLASVNGSLGFYGNTFENFSSPNLTSVGGSLAFVSNEQLNNITLPELTTIGGGFQIANNTNLESINGFPKLATVGGALDFTGSFSGVEIPALEDVRGAFTLLSNQDIQEVCSTFQGQAGSARAIKGTFTCKGNTDPKDGSATGSGSGSSSSDSAASPLLIPGATGVLGVVAAIFGLL |
| 99 | Unigene0011104 | 150 | 65776 | 9.7 | LFLSAQSTTASPLADSEAQNEHEKRQLTTFDYVIVGGGTAGLVMANRLSEDPAVSVAVIEAGSYYQVSNPLLSSTPAGDTFFVGADPSKAQPLVDWKIVTQPQAGANGRRILYARGKCLGGSSARNFMIYQRPTAGSLQQWADAVDDDSYTFSNWLPFFQKSVKFSPPGPARASNATTGFDASAFSANGGPLDVSYANYAGPFSSWIQGSLNEIGVSTNDQGFNSGHLFGAQYCSSTINPSDQKRESSQTSFLDAAARRPNLRVFTLTKAKKILFENKRATGVRVTSGGFIPYTIRARKEVILSAGAFQSPQLLMVSGIGPASTLRRFNLPVIQDLAGVGQNMWDHIFFGPSYRVKVETLTRVVNDPLYLAAKFAEYEGQKTGVLTNPVCDFLGWEKTPAALKNRFSATARAELSRFSADWPEIEYLSAPGYVGEFSDLPSTQPKDGFQYASILTALVAPLSRGTVTISSSDTDDLPVIDPAWLTSRTDQEVALAAYKRNRAAFASNFMKPVLADPVEYFPGPRVQSDADILATIRSTLLTVWHAACTCKMGVASDPMAVVDNRGRVFGVTGLRVVDASAAPLLPPGHPQSWIYALAEKISSHILNGD |
| 100 | Unigene0013119 | 149 | 52440 | 8.4 | MGEPAQKFKVADINLAAFGRREIELAEQEMPGLMQTREKYAAEQPLKGARIAGCLHMTIQTAVLIETLKALGAELTWTSCNIFSTQDHAAAAIAASGTPVFAWKGETEEEYQWCLEQQLKSFPSGQPLNLILDDGGDLTALVHTKYPEMLTGCYGISEETTTGVHHLYKMLKNKGKPSGLLAPSINVNDSVTKSKFDNLYGCRESLVDGIKRATDVMIAGKIAVVAGFGDVGKGCAQALHSMGARVLVTEIDPINALQAAMAGYQVVTMEEAAPISQIFVTTTGCRDIIVGKHFEVMREDAIVCNIGHFDIEIDVAWLKKTAKEVVSIKPQVDRFLMPNGRHIILLAEGRLVNLGCATGHSSFVMSCSFTNQVLAQIMLYKAGDRQFATKYVEFARAIEGEADGPIKVQEEGQVKSSVPRMPIGVYVLPKILDEQVALLHLEHVNVKLTKMTEAQAEYMSLPIEGPFKTEMYRY |
| 101 | Unigene0009857 | 148 | 67927 | 11 | LLLAVLHLAAGEQPSAPAPIAAPLRELPWGQLNFLHTTDTHGWHGGHLQEAQYSADWGDYISFAHHLRKRADEDGSDVLLIDTGDRVEGNGLYDASDPKGKYTLDVIKHQSIDVMSVGNHELYKQNTSINEFNNVVPAFNDAYLASNLDIISPKSGEQVPLARRFKKFTTKNQGIRIVAFGYLFNFKGNANNTIVKPVQDTIKEQWFKDAIADKEVDLFLVIGHVPVRDTPEFEAVYKAIRQENWDTPIVFFGGHTHIRDYRKFEKKAWGLESGRYMETLGFLSIDGLSTGKKDVPTASKSVEYKRLYIDNNLFSMHHHSGTNETTFGTELGRNVSQAITEARTQLKLDHTFGCAPQDYWLSRVPYPSNNSLLTLLETQVLPDALKGTKKPTIAITNSGALRFDIFKGPFTIDSTFLVSPFTSGFRVLKDVPFKAASRVLQTLNDQGPILLADMITMAGEDGAKLKELLPHHPVRADSLLSADESPSRLQLLLGAQSQTPFGSGDGDAPRVPGYTTKDDAGTDGDDTIHQRIDFYNVPNVIGSNFSFPTTATNPETVDLLYNEFIENFVLLALRYLGEKREPKDSSRAVDGKTLTDVISEWIQDHWQCE |
| 102 | Unigene0009770 | 143 | 43364 | 10.6 | MKGALMASALVAGAQAGVHKMKLQKVPLSEQLEGIDIETQVQRLGQKYMGIRPQSRLDEMFKETSVHAEKGHPVAVDNFLNAQYFSQIAVGTPPQEFKVVLDTGSSNLWVPSQECGSIACYLHSKYDHGDSSTYKQNGSSFAIQYGSGSLEGYISQDTVQIGDLKIKDQLFAEATSEPGLAFAFGRFDGILGLGYDTISVNKIPPPFYNMIDQGLLDEKVFAFYLSSTDKGDESEAIFGGVNKDHYTGDLTKIPLRRKAYWEVDLDAIAFGDQLAEIDSTGAILDTGTSLIALPSTLAELLNKEIGAKKSYNGQYTVDCSKRDSLPDLTFTLTGHNFTIDAFDYILEVQGSCISAFMGFDIPEPAGPLAILGDAFLRKWYSVYDLENNAVGLAKAK |
| 103 | Unigene0013198 | 142 | 43221 | 9.5 | FPASVLDQVAQDPALTARAAEIMENVKRQEGADGATAIFEANPTFSEEQLIDVGPGSGHEWQAPRQNDLRGPCPGLNAFANHGFLPRNGYATISQFIDATTTVVGMGPILAAFLAVLGASIDGDGTSWSIHGTPPLGVGGPLARFGNGISGSHNKYEADASPTRFDLYQEGNNFKINIDQFQELIDFSQSGFVDLNSLTAFRSKRFDDQIANNPYFFNGPFTGVLVQPAAYTFIYRFMANHSAEDPVGFLSHEVLQSWFGIEGESGSYSVNPGAERIPDNWYRRSLTTPYESTYFLGDAANAIRLHPKFANVGGNTNGPNTFTGVDITDLSGGVMNGDNLLEGNNFACLAYQFAAQAKPDLLGALGGLTSPLQNVLGPLSCPQLRALDEGLLEQFPGYQRS |
| 104 | Unigene0002737 | 141 | 52946 | 11.2 | YEQQPLLSSSGKKLPLVETEKLQEAISGDNLLARAQDLYKIAELSWHEYNHPTRVIGSAGHQATLEYIWATIAKLGDYYNLTEQNFPAYSGNVFESRLVLGDEVINNASAMSLTPPTKDNEPVHGLLVPVENSGCSASDYPASVKNSIAFIKRGQCAFGDKSLNAGKAGALAAVIYNNENGTLHGTMGTPDPQHVATFGISLEEATPTLDKLKSGKEVDAIAYIDAVVSTIHTQNIVAQTRDGDPDNCVMLGAHSDSVPEGPGINDDGSGTISLLEVARELTNYTVNNCVRFAWWAAEEEGLVGSNFYSSTLPEEENLKVRLFMDYDMMGSPNFAYQIYNATNTESPAGSEQLRDLYVDWYKKHGLNYTFIPFDGRSDYDGFIRAGIPAGGIATGAEGIKTKEEAKVFGGKAGDWYDPCYHQLCDDVGNVNVTAWVVNTKLIAHSVATYAVSLDDFPKRTDAAVMKESAYVQNVKYRGSRLFI |
| 105 | Unigene0012945 | 140 | 46935 | 9.8 | TLSVLLPILSLGLTGVDAAGPLKGHPIKHSHLHAAVDLPKRSGHEQAAAAHGAQQGFKTVGYYASWSIYARNWPPQNIQSEQLTHLLYAFANIDNKTGEATLGDPFADVQKKFDTDDAKDNSTSNLYGNLKQLYLLKKKNRNLKTLLSIGGWNIRSYFAPALANEQGRKNFARTSVQLLKDLGFDGLDIDWEYPNNTKQAQDLVETCKEFRKELDNYSRNLTGNPHFLLTMAVPAGPDHYPYFDMAAMEPYVDFINLMAYDYAGSFSDYSGHQANLYKSLENPRSTNFSAEGAMEYYLNETGWKPEKLTFGMPLYGRSFANTTGPGKNFTKATDGSWEAGVWDYKALAGNESFGEVYTDEKIVASWAYDERDCYMVSYDTPEIARRKAEYIKSLCLGGAMWWELSGDTPVNGTSSLIGAV |
| 106 | Unigene0013050 | 139 | 17795 | 19.4 | MKFLAAAAATSALFTTALAAPTACCNDTDGSVFQIKDFSLRKRNGKDISNVSFRILATNGGTLDFQCIPYDPATDAATKDFVPGQVYFCGKDSSLSFSYTPKHDDQKQNELWLWQTITQEQVYAGHVDFDDPICRAGGAGQQDFICNVPQGVDLFVTVEK |
| 107 | Unigene0014926 | 139 | 50754 | 12.1 | FQQCIASALDNNSALYAFPQDILEELTNNHPYNLDWPWTPAAIAYPRNAIDVSKLVVCASEYGIAVQGKSGGHSYLNFGYGGQNGALALDLQHMNDFSYNPADQTVTFGPGNKLADLDVQLAAIDRVMAYGVVGDIGTGGHIAIGGLGPLSRLLGLASDQVVSVQCVLADGSIVTASEYEHPDLFFAVRGAAWSFCIMTEIQMQTSPPPSAVTQFQYNITVGRIADLADTFKKWQVLIADPGLSRYFAATLTLLEGVMIFAGTFFGDDAAFNQLNLDLIPFGQEHLGITVTSRVVTGLIHSLSDLIYDIGGSIPAHFYSTDLKYTRQTLLSDAAVDQLFQYLESADKGTLIWFVVWDLNGGRIEEIPQSATAYWHRDAIHFQQGYVVNPLGPVSPVSRQFLIGLNQLCRQLRPGIDESAYPGYVDPFLQDPLTAYWGGNVPRLIQIKGVYDPRNTFRNPQSI |
| 107 | Unigene0014925 | 139 | 52766 | 11.7 | FQQCIASALDNNSALYAFPQDILEELTNNHPYNLDWPWTPAAIAYPRNAIDVSKLVVCASEYGIAVQGKSGGHSYLNFGERRLFGVSSNHELTSEIGYGGQNGALALDLQHMNDFSYNPADQTVTFGPGNKLADLDVQLAAIDRVMAYGVVGDIGTGGHIAIGGLGPLSRLLGLASDQVVSVQCVLADGSIVTASEYEHPDLFFAVRGAAWSFCIMTEIQMQTSPPPSAVTQFQYNITVGRIADLADTFKKWQVLIADPGLSRYFAATLTLLEGVMIFAGTFFGDDAAFNQLNLDLIPFGQEHLGITVTSRVVTGLIHSLSDLIYDIGGSIPAHFYSTDLKYTRQTLLSDAAVDQLFQYLESADKGTLIWFVVWDLNGGRIEEIPQSATAYWHRDAIHFQQGYVVNPLGPVSPVSRQFLIGLNQLCRQLRPGIDESAYPGYVDPFLQDPLTAYWGGNVPRLIQIKGVYDPRNTFRNPQSI |
| 108 | Unigene0008279 | 135 | 34404 | 14.5 | MATERESKTFLARLCEQAERYDEMVEYMKEVAKVHSFSISYRCSFCTRSCKDELPATTTNDHUHLTULGGELTVDERNLLSVAYKNVVGTRRASWRIISSIEQKEEAKGSDKHVGIIRDYRQKIETELEKVCQDVLDVLDDSLIPKAETGESKVFYHKMKGDYHRYLAEFASGEKRKTAATAAHEAYKNATDVAQTELTPTHPIRLGLALNFSVFYYEILNSPDRACHLAKQAFDDAIAELDSLSEESYRDSTLIMQLLRDNLTLWTSSDGGEPEAAAGETKAEEKAPEPAAEPPTEEAKPAA |
| 108 | Unigene0008278 | 135 | 30293 | 16.4 | MATERESKTFLARLCEQAERYDEMVEYMKEVAKLGGELTVDERNLLSVAYKNVVGTRRASWRIISSIEQKEEAKGSDKHVGIIRDYRQKIETELEKVCQDVLDVLDDSLIPKAETGESKVFYHKMKGDYHRYLAEFASGEKRKTAATAAHEAYKNATDVAQTELTPTHPIRLGLALNFSVFYYEILNSPDRACHLAKQAFDDAIAELDSLSEESYRDSTLIMQLLRDNLTLWTSSDGGEPEAAAGETKAEEKAPEPAAEPPTEEAKPAA |
| 109 | Unigene0013463 | 135 | 41839 | 10.9 | MEEEVAALVIDNGSGMCKAGFAGDDAPRAVFPSIVGRPRHHGIMIGMGQKDSYVGDEAQSKRGILTLRYPIEHGVVTNWDDMEKIWHHTFYNELRVAPEEHPVLLTEAPINPKSNREKMTQIVFETFNAPAFYVSIQAVLSLYASGRTTGIVLDSGDGVTHVVPIYEGFALPHAISRVDMAGRDLTDYLMKILAERGYVFSTTAEREIVRDIKEKLCYVALDFEQEIQTASQSSSLEKSYELPDGQVITIGNERFRAPEALFQPSVLGLESGGIHVTTFNSIMKCDVDVRKDLYGNIVMSGGTTMYPGISDRMQKEITALAPSSMKVKIIAPPERKYSVWIGGSILASLSTFQQMWISKQEYDESGPSIVHRKCF |
| 110 | Unigene0011520 | 134 | 25100 | 11.9 | SDLTTDYLGASHDRIFESNRKWASEKAKTDPEFFTKLSSGQKPDYLYIGCADSRIPSNEIMGLDAGEVFTHRNIANLVCNTDLNVMSVINYAVRHLEVKHIVVVGHYSCGGVKAAMTPADLGILNPWLRNIRDVYRLHEQELDAITDQDQKYNRLAELNVLEQARNVVKTAAVQQSYAKNKFPIVHAWIFDFKDGLLKDLKLDFEKQLRDIQKIYNLTG |
| 111 | Unigene0014414 | 134 | 49684 | 8.8 | FLRFFTMLSFGKKWLHVFALLALTFVLATGHPQKRAIIPAIDPFYKPPAGIESAAPGEILRTRRIIPATFGLIPTLGVEAHQLLYRTTAIDGSAIAAVTTVFRPLVAKADRFVSFHTAYDAAATICNPSYTYQFGAIPFNLITSVEYLLLQAFLASGYIVSSPDYEGPDAAFSAGRLSGTGVLDSMRAVKNFRTTLRLSTSTPKIVGYGYSGGSIASGWAAQLNPTYAPDVKVSGWAAGGIVANLTGTAVFIDNTLFSGFLPVAFAGLNKPSSYAARLDPVIDQIITPAGQAQLDSVNQNCATQLFTFAEQSVLSTSFQSLGDQVLYQADIASVLAQQTMGQSNTENPIAPVYVFHASNDEIIPYSNATTLVNSWCSKGVSVDYVTYAAGGHATTEIVGFVGAFQFLEKAFAGTLGSGCTTSTKLDETLNPLALGLNLEPILVRLIDALAALGRGDANLRQDLSVL |
| 112 | Unigene0011480 | 133 | 45620 | 16.9 | MKLSVLLTVLSAAFGQAQYDGDVVAYWVDSSSSLSNNSLIGGLQSPPSGWFTAIVHASVYVAALQSQDEELAFQQLAVSHAAHNALTWTFHGTRNFANIYDKMSNITARIGINHTSDDYRRAAEIGQAAALRVTTARQGDLLDNFVQYVYGPEDLGVYQRTPGGLTAPDTPQARYLRTFGGIGNITRFRAPPPPAINSEEYEGILNYVKAQGEQNSTERTEHNTETAYFWRESSPIIWSRFAYNVLANRYATDVLSSAKFAAQLNYALANAAIAGWDSKSFYNSWRPVTAIRRTDVWLPSGNNVSDPEWTPLLSPTPNHQEYTSTHACFGAAAAAVIKAWNNGSDTIDTLLSTNATGRGVIQRRYTSLQQAVKDNGDSRIFGGIHFQFASDVGSVVGNDVGEATLKAFDDRWWEF |
| 113 | Unigene0002359 | 132 | 47821 | 10.1 | MRLAGSVVALLPAIGIAAFVPADTSGTDQLACKALDNVRLRTSRQLPSTLASLSLSITHRRLPRTESTKPPADGNTQCTFENAKVRREWDTLPAEEKRAYIDAVLCLMSKPSISGDLVPGARSRYDDFLGTHINQTLSIHGTGNFLSWHRYFTWTYEKALREECGYGGTFPYINWGRYAEDLYNAPIFDGSETSMSGNGDPDSRNNGTWVPNAALPQIQLPGGEGGGCVTSGPFANLTVNLGSLAPALSYIPVNPLPNGLGYNPRCLRRDLGAYAAETWLRDPNTTALIANNHEIGPFQSIFQGNFSEGFLGAHTAGHFVVGGDPGGDLFASPGDPYFWVQHSQVDRVWWIWQNYPENSWKYNGIEGDEKDRLYVIGNTTTTNNMTPSRDASLDDRVDLGVNDGFGVGKGADGQGYKIRELVDTMSGPFCYLYE |
| 114 | Unigene0013096 | 132 | 16735 | 19 | MAASGVSVAPECITAFNELKLGKSTKWIIFKISDDWKEIVVEETSSDPDYSKFREKLVSAQSKSKRGADAPLGIGGRYAVFDVQYEAPGGAGTRNKITFIAWVPDDAPQYPRMMYSSSKDALKRALNGIAADVQANDADDIEYDTIVEKVSKG |
| 115 | Unigene0012985 | 130 | 31307 | 6.4 | LSKFIPALAIAGAASARIVGLAAPKVIAVNETFTVTLLTENYIQSVKDLVAAFALSPRADADGFIGTEYLGSFYLGPDKSNVLTNITFEVTAPATNVGQYLNGVVTSLYGASNGATTIQWSVPITYGDEVSTEQVRSIEGNNVCSSSTPTDPGSNTTTTAPPSTDPTSPPATGNCFPTSTQTLIQSSLVYANALIDSIVQSNNQTGRQNLGQLNGFLGDVSAAVGVGRGGESCNNPPSPSWPPLSPGDSQVRAISILRNVQDTLQAEQGAILQCNDAQAKSIQCQVLRLVDNLDSYYS |
| 116 | Unigene0001074 | 129 | 56005 | 11.4 | VACPNTQLVRPANGLNSDESKYIRSRKPKADASLSAWLAKRGKFLTSSQPLVALTSSGGGYRALLETAGVVQALDGRDGNFSTSGIFQGLTYEAGLSGGAWFLSSFSGNNWPTVSSLRDGLWEPQFQASLLLPANIQGTDKYAEVTTDIVAKQLAGFDNTIIDAWGRLLSYQFLYGEDGGDDIRLSTLTSFSNFTSFNVPYPIMTALGVDDSSEGQCVPTINATQYEFAPYEYGSWDQGVSAFASTKYMGSDLTNGRPTNAGKCIEHYDNIGYIFGTSSDIFVAACSVLTPSNSSNADLANFLQDIISVSKRPAFQDLFGIYPNPFFNYSRSTTVKNDKLITMADGGLAGQNVPIWPFIQPARKIDVLIANDNSADTTDNFPNGTEIRQTYENARAVGLTRMPFIPDVAEFVAKGLNKRAVFFGCDEPNTVFIIYLPNVNYTYPSNQPTSKIQYSVEETNGMIQNGNAIATQNGDASWGLCFACGIKARDGRGKLPKGCDACFQKYCYRR |
| 117 | Unigene0012557 | 127 | 81448 | 6.1 | MRSALGFALSALSGVAWAQTGLKVDGTNFTLTGDGVSYLFHVDPKSGEVVGDHFGGTVTEFVPPAPISTQGWHDGLALVKREFPDVGRSDVRLPAIHIRHADGNTVTAFAYQSHEVIEGKPALPGLPATFGNASDVSTVKVTLYDNYSDVTAVLSYSIFPQYNAIARSFQIHNNGTEDIVIERAASFSVDLPNLDLNMVELQGDWSHEMNKVIRKVQYGETGFRSTEGYSSHLHNPFFALVDPTTTESTGEAWGFNLVYTGSFAATAERFSHGYVRVLLGLNELHSTLSVAPGETFTSPEAVAVYSSEGLGGMSRSFHSLYRNHLSRSHFTHETRPILLNSWEGLGFGYNDTSMVQLAGEAEALGVKLFVNDDGWFGTDFPRNNDTLGLGDWTPNPAKFPNGLGPYVAAVNNITTEGTDASPMKFGLWVEPEMINPNSTLYREHPEWVMKSGQHKQTLTRSQLVLNVALPEVQDFIINFVSELIDNANVEYIKWDSNRAIHEMPSPSTDHKYILGLYHIIDTLTTKYPQVLWEGCASGGGRFDAGLLHYWPQSWTSDNTDGSDRLTIQMGTSLAYPPSAMGCHVSKVPNGLNGRNISIEYRAHVALMCGSFGFELNPHELTPEETSAIPEIMEIANRIGPIVISGDFYRLRLPEESNWPAVQFVSANESTAVVFAFQQTNAIKPAPPSLRLQGLDPEARYWNDYDNSTYSGQTYMNGGLNLDFPQRDYQSMLVWLYK |
| 117 | Unigene0012558 | 127 | 81448 | 6.1 | MRSALGFALSALSGVAWAQTGLKVDGTNFTLTGDGVSYLFHVDPKSGEVVGDHFGGTVTEFVPPAPISTQGWHDGLALVKREFPDVGRSDVRLPAIHIRHADGNTVTAFAYQSHEVIEGKPALPGLPATFGNASDVSTVKVTLYDNYSDVTAVLSYSIFPQYNAIARSFQIHNNGTEDIVIERAASFSVDLPNLDLNMVELQGDWSHEMNKVIRKVQYGETGFRSTEGYSSHLHNPFFALVDPTTTESTGEAWGFNLVYTGSFAATAERFSHGYVRVLLGLNELHSTLSVAPGETFTSPEAVAVYSSEGLGGMSRSFHSLYRNHLSRSHFTHETRPILLNSWEGLGFGYNDTSMVQLAGEAEALGVKLFVNDDGWFGTDFPRNNDTLGLGDWTPNPAKFPNGLGPYVAAVNNITTEGTDASPMKFGLWVEPEMINPNSTLYREHPEWVMKSGQHKQTLTRSQLVLNVALPEVQDFIINFVSELIDNANVEYIKWDSNRAIHEMPSPSTDHKYILGLYHIIDTLTTKYPQVLWEGCASGGGRFDAGLLHYWPQSWTSDNTDGSDRLTIQMGTSLAYPPSAMGCHVSKVPNGLNGRNISIEYRAHVALMCGSFGFELNPHELTPEETSAIPEIMEIANRIGPIVISGDFYRLRLPEESNWPAVQFVSANESTAVVFAFQQTNAIKPAPPSLRLQGLDPEARYWNDYDNSTYSGQTYMNGGLNLDFPQRDYQSMLVWLYK |
| 118 | Unigene0001730 | 125 | 33479 | 8.6 | MSEITHPTIKDGWFREINKMWPGQAMTLKVNQVVHHEKSKYQDVLIFESSDYGMVLVLDNVIQCTERDEFSYQEMIAHLALFSHPNPKKVLVIGGGDGGVLREVVKHESVEEATLCDIDEAVIRLSKKYLPGMSVGFNHPKCKTHVGDGFKFLDDYKNEFDVIITDSSDPDGPAEALFQKPYFQLLHDALREGGVISTQAENPWLHLNIIQQLKKDCKSVFPVAEYGWTTIPTYPSGQIGFMVCTKDASRDVTKPLRTISDEKEDELFRYYSKKVHEAAFVLPKFAEKALRE |
| 119 | Unigene0004807 | 122 | 64790 | 8.1 | MFARVPLILSLATATLASNLDSPAAYSPPPQQPAAPNGYGQQSTLTITRTVYNNPAATKAPPVPPIPSKNTTSPTVNSNSTGSCKNVQSSKIRADETRAAAVKEAFLHGWNAYVKYANGFDELAPLALNGTNSRYGWGLTIVDSIDTAIIMGLTDIVNDMLDFISGVDFTKTEFVEADEGVNLFETNIRYIGGLLGAYDLIKSEQFGTYDQQKVDMLLTQATILADKVAYGFNTPSGLPATFANFTSNEPVYDTYTDPVTDTTYNSTNAASIGTFLLEWGRLSDLTGNSSYREIVEKADSYLINPSPAPALTNLIGTQFDVETGNMLTKDGGWQAGVDSFLEYLIKYYQYSPSNVAQQYKDFWVKATQTTIDNLVIHPRGFNQLSFVSRLSENGTVDNLADDFSCFAGGNWLLGGALLDDPKITQLGVDWTAGCYHLYNTSTTGLGPLTWSWFDQQGNTVNKNFQNDAAALRSAAARGWFIPNNLENWFSRPEPLESVFYAYRITGDQKWADANWRIFQAINTTARTAEAFAAVNNVDMPYGGSMSNNLDSFFFAEALKYLYLTFVDGSVVDLSAWVFNTEAHPLQAQCA |
| 120 | Unigene0005812 | 122 | 94779 | 4.7 | CLLTTCTLAQNQQNMSTRLTSYVDPLIGTVGSTPGSAIAGGNSFPGATLPWGLAKPGIDTSYLGVPNGTAVDCNAGYSPLGNVTGISMTHVSGTGGVPTYGLISQMPLYGDLSGVNLADNTTYWQNRSLEDEKATVGHFRTILLNGIEVDIASSRRAGLIKYTFPASIASNASMSGSTPEVVQTATIEDAHVLVDLTHVLPGYGTQAYSQRYTHGTLHTRTGKNGQPSYSGSATYTGGWSQPQSHTLYFCGNFSATNLEPTDAYVTQNSRDRVPGAGTINWPYGPSEPPAFKNRPIPRSFTELESFAGNGMGIGALFSWTPSANNASAGREILARIGISYVSHEQACVNSANELPANLSFTEVVERSREEWEKEILGAIEVVDDGSASSSNTTLKRMLYTALYQTGLMPTDKTGESPAGYDGIPYFDDHYTLWDTYRSLTPLYHLLYTETYSRVIKGLISIFTVEGWLPAGRIANWNGRVQGGTHADMVLADAYVKNSGELADDIDWQEAYRAVLNDALNNPVHNVDTATFDGATKEGRGALDDYLSLGFITRNHTRSISRGVEYSQNDFAIYSMSKSLSNSTTQTSSFLNKSSWWQNQWSPSANTSLYIPTLSNSTSNTTFTGFPAPRNSNGEWNFTAYDPLTCGNCGWADDIYEAKPWETAFGAAPHDMSTIINLMGGSQKFVQRLDASFIPGLGSSVGANNDAGTALFNPGNEPSFLTPYLYNYVSGYQWKTVQRTREIVDQYYSDERNGYPGNIDGGALPSWLIFNLIGLFPVAAQPVYLIGAPRFSSLRIKLFTGTKRETTLNITAPGLSDTNLYPQSITFNGKNHTRSWLSHEELSQGGNLVFNVGPEPSSWDIGERPPSLSGWS |
| 121 | Unigene0008792 | 117 | 29533 | 14.4 | MGYEDSVYLAKLAEQAERYEEMVENMKAVASADQELSVEERNLLSVAYKNVIGARRASWRIVTSIEQKEESKGNEQQVTLIKEYRQKIEAELAKICEDILEVLDKHLIPSAQSGESKVFYHKMKGDYHRYLAEFAMGDKRKTSADKSLEAYQAATEVASTDLAPTHPIRLGLALNFSVFYYEILNSPDKACQMAKTSFDEAIAELDTLSEESYKDSTLIMQLLRDNLTLWTSSEAEPAQDAPAAGEGESKDTSDAPAGEEAKPA |
| 122 | Unigene0002508 | 116 | 52347 | 14.4 | MKFSSVLPLAAATSAFVLPPEEVLSELKIEDHHLEAKVDHWLEEASSTKDEFLSSVKKYFGEVSETTKDAWKEISETGKSVLDQALEKAHDTAESLGSTLGDAADDFQSYVEDNDFFTGFGGDHHGKPKHPPHHHEPNETVYQLIAGSKYTTKLAKLISEYDDLVDALNSTKANFTVFAPTDSAFEKIPEHAPKPSKEQLKAILSYHVVPDVYTAGRVLVTHTAPTLLKGEHLSSQPEPQRIAFKIGLRGLTVNFYSRIVAINIFGTNGVVHGVDSIILPPPNAITLVDLFPGEFSTLELGLGKTGLLDTLNTTHHAGGTLFAPSNWAFQKLGPKINAFLFSSYGLKYLKALLEYHVVPGNTLYSDAYYKAKDDKDAATNVDIPKGLFHVDLPTLLKDRNLSVDIARYGGFISIKINGFATVAVQDGIAEDGVIQVTRDVLIPPKQLAGAELEQWDGSELTIEDLKERLEPFVAKTDL |
| 123 | Unigene0011045 | 113 | 56047 | 14.8 | PITEGQLAYKGPGLLKSIIPSKKEFWLSDEAVAPQNLDLYLRGEKPEISHPVVAWASHTGKGLLFFNKKGETNRTHPSDVLALYDASDLKKSAQHEFSFKLNGDNHTFKASNDAERDGWYLSIERAIEHAKASKEEIRASEGYKAEIEKLNKPNVVGAATTRSLSKSKKSTDVPPTERIGSSDGEADEDKKKSQKSRSTSRGILNRFKTNKEESAEKKVDSEDKKEEVKASEETPVAASTEAAPIVASTTAAEIPAAAEVAAEEKAVETPVEEKAKGGKRGSIFGRVSSGWSGLRSPAKEKDLKDAELKNETTTAAPVSENPPVLPETTATTDSTAVTAIPAVEEPVAETKTEEKKDEVATPNKEKNNFLSGIGGFIKRNRSVSPNTHKKEAAKAEETAPAPAETAVTEPVVKAEEPATESAATAAEPSTEAPAEKVEEPKTEATTPNKRQSVLGSLGRRASKALNRIQAPKKENAAPAATDAKAEETAAVKPAEETPAVNGETTKDEPEAPKTIGDVVPDAVNVG |
| 124 | Unigene0000605 | 112 | 14634 | 28.1 | MKLATVIAALALGVAAVDKPLDIEKTHTIECTTRTKVGDKVEMHYRGTLASDNSEFDASYNRNRPLSFTLGKGQVIKGWDEGLQDMCVGDKRKLTIQPEYGYGDRGMGPIPGGATLVFETELVTINGKGAEEAPA |
| 125 | Unigene0012555 | 111 | 49158 | 7.6 | TFTSVTALALAGNVAAFWRMPCRSHTGIARIDPLVAPADVADHSHLVFGSGNFGNNATFKDLTQCPDLSHDCSSCAVTQDKSAYWTPPMYFKHKNGTVEMVENVGGMLAYYLFYLDDVQPFPEGFQMIAGDPTFRNFSGVFPDEPLSSWPTDPSDQFFLEQRAIGYNCLNYAKDPEPSLYRHVFPERSVMDSQCKDGLRLEMAFPSCGTGELDSRNHRSHVAYPSLVKEGNCPKGYDVHYPFLFYETIFATQKFAGMDGEFVLSTGDPIGASYHADFMMGWDSADFLGKAINTCTSSSGRIEDCPLFNIQSDAEAAKCKFPVPDYLDGDDCTGPRDDLPVGVPIQYGPEPATKYPVQGAKGGPATHAPKPSSAPKNEPKPYKPAEAPKPSEVKPNPPAYAPAEPSVTAAPSAPPASKGNKITTKYVTKGNEVYEYIIEEVDVTVT |
| 126 | Unigene0006466 | 110 | 49359 | 11.3 | MAGQEQQPRFETLQLHAGQEPDPTTKSRAVPIYATTSYVFNDSAHGARLFGLKEFGNIYSRIMNPTVDVLEKRIAALEGGVAALATASGQAAQFMTIAALAHAGDNIISTSNLYGGTYNQLKVFFPRLGITTKWVNGDKAEDFKALIDDKTKAIYLETIGNPRYNVPDFEAIVKVAHEAGIPVVVDNTFGAGGYFARPIDHGADIVVHSATKWIGGHGTTIGGLVVDAGKFDWGTPAARKRFPQMSEPSDGYHGLKFWDTFGPITFIIRLRVEILRDLGAALNPFAAQQLLLGIETLSLRCERHASNAQKLATWLENNPNVAWVSYPGLESHPSHQLARKYLPRGFGGVLSFGVTGGGAAGSQVVDGFKLISNLANVGDSKTLAIHPWTTTHEQLSDEEKINSGVTEDLIRISVGTEHIDDIIADFEQSFAQAATKPGQGQDNSDVTGNPNAPGGKPVV |
| 127 | Unigene0010371 | 110 | 42554 | 11.7 | QVRGFNYGALYADNRARQLADWEADFRAAQDLPGVGGFNSARLFTMIQGYSQTDIISALPAAVSTNTSLLLGLWCSGGDEAFNNELTALRNAIEQYGDQLRDSIIGISIGSEDLYRISPTGIENESGPGAQADQLVGYIQRTRDVLQGTAWADVQIGHVDTWTAYVNGSNNAVIEALDWVGVDAYPYFQTVNENSIQNAQQLFFDALQATEGAAQGKPVWVTETGWPVTGPQSGLASSGTENAEQYWKAVACRLLGNVNTWWYQLNDGADQEVSFKVINPDRGAPLYDLSCPANSDDDEESTSSVTAVSGSGSLIPVPTGTPSPRPAGGNNSTSTATGSTGSPNDNNQNAGPVAGGSGSGSGSGSGNGTAPTGSSPSATSSGNTASYTGAAVANAPAAAGLLLVGAIMAAL |
| 128 | Unigene0003811 | 110 | 21626 | 25.1 | ELMQVVEEAPSPDIDPFGIEATGEALGTFPVQITASNGRIYIGGNQTQPTNCSSAQSVHDFATFIWRSDRTLWLYGQDPAHSQQIWADVGADGLTGYSTAKEATPGQASLSPFEIDPETRIVTFNGSTVKGCPKDSTKPTTAPFGIWFSTEVKPGNLANCFDVTLQAYKTVYADACTYSHLASCDEAPETCGSMKDPVG |
| 129 | Unigene0012361 | 109 | 25920 | 4.4 | MKSYIFAAAALVSAVANGAMIRREEDCDEPKAPTYSSSSHHGTNGTSGQGSNGNLPCGATSSTTYTVVSGDTLTTISTATGAGICDIASASKVTNVNLITPGQVLTIPQGCTLPIDNTTCITTPDIPATETCVAGLPSTYTIVSGDTLTAIAKDFNITLDSLIRANTQIANPDAIAVSQVINVPVCPNSQCASVGPYVIVSGDLFVDLAATYKTTVGQIKALNPTVNATAIAVGQQIILPKNCANVTAAPA |
| 130 | Unigene0005976 | 108 | 32864 | 15.2 | SMPQWGIRKVGAPHTLEHRIYIEKDGVPVSPFHDIPLYANEQQTVLNMIVEIPRWTNAKQEISKDEELNPIKQDTKKGKLRFVRNCFPHKGYLWNYGAFPQTWEDPNVTHPETKQKGDNDPLDVCEIGELVAKPGEVKQVKVLGVMALLDEGETDWKIIVIDVNDPLAPKLNDIEDVERHLPGLLRATNEWFRIYKIPDGKPENQFAFSGECKNRKYATEVIRECAEAWDKLITGKTQPGDVNLTNVSVPQSANRVDPKSLNIPAGEEKAPAPIDPSIDKWFYISGASA |
| 131 | Unigene0013227 | 103 | 37581 | 12.7 | SGDYSHELNVALLAVQRASLLTKAVFHQNAKGTLNKSDASPVTIGDFGAQALIISALQHNFPNDEIVAEEEARDLRENETLRNTVWELVKNTSLSDAAAEKQLGGPLSSPEAMLDIIDKGDSKGGNKGRIWAIDPIDGTKGFLRGGQYAVCLGLMVDGDVKVGVLGCPNLPVSDSEPLKENIGADASDEEGKGVLFSAVLGQGAASRPLGAGSLASSSKIQMKPISNIADATFCESVEAGHSNQSDSVQIANKLGITKPSVRMDSQAKYGSIARGAGDLYLRLPVKKDYVEKIWDHAAGDLIVREAGGQVTDVDGNRLDFSHGRTLLQNKGVVAAPKDVHKNVIEAVQSVLGSKQ |
| 132 | Unigene0000021 | 102 | 44370 | 6.2 | MKAGLLVAALAATATAQLHRRGHRHARRENPVAYHEEVKVVTETAKHVVYVDSNGNPIAEPAKTPAPAPPAYQAPPAYQAPPAYQAPPAYQAPPAYQAPPAYQAPPAYQAPPAYQAPAAPAAPAPAPAAPKQEAPKEAPKEAPKPAAPKTEAPKDYKPSSGGGQGIVYSPYNNDKSCKNQDQVNADFEKLGDYKLVRIYGCDCDQVNTVHKAATAKGMKVFIGIFDINQVQSDVEKIVAVANGDWSWVDTVSVGNELVNNGAASVDAVVGAVNAARSQLRGAGYQGPVVIVDTFVAMIANPAICEASDYAAANCHAFFDGGKTADQAADFVAEQAERVKQACGGKKVVIAESGWPWNGATNGVAVPSKENQAKVIDGLKSKFSENIYLFTAFDDLWKDNFAGSHGAECHWGFIEHSA |
| 133 | Unigene0008731 | 102 | 67153 | 4.2 | FTVLLRFSLASASMKSFIACSLLGGLSLACAAPATQEYDYVIVGGGVSGLVVANRLSENEQVTVLVIESGESVDTDATKIPYKANDLTSSAGLIWENITSLPEPGLGNSTFSVLVAKVLGGGSVINGMVYDRGSAADYDAWEALGNEGWGWQGLLPYFKKGTTFQPPSAAVAESFNITWDPEAYGDGPLPVSITDSQYPDTKDYWAAWRATGVHVPIDGNNGEAYGPSWYPNTMDKTTGRRAHARYAYIDPISQRKNLKVLTGTTAEKIVFGDANSPYEATGVEVSTAGTGETAIINARKEVILAAGAIQTPKLLQLSGVGPRSVLEAAGIEVKVELDAVGSNFQDHPYATVIFNTSSTTFPSANSLVNNATFNASAWEEYEVNKTGPYTYARGNALAFISLPDMTSNFSSVSYQLDNENAIDYLPSLYQNNTGLLAGFTAQRDIISSIFGRKDAAVAEFPIPVDAAYSIVAVQKPVSRGTVHLNPANPTGAPNVLHNAFVNPVDRLILSIGMRYLRDIFSYHPLQEKFSISEIVPGTQYTSDTEIYDALVEQQTLSPTFAHPSCSCPMMLEELGGCVSDTLLVYGTQRLSIVDASIIPIIPSQHLQSTIYMIGEKAADLIKAR |
| 134 | Unigene0013716 | 102 | 81636 | 5 | MGKAFKKPPQAPPVFTHTPETVIERTKRLLDANKSLQDKIVKDITPETATFENVELPFAQDGNNMALEAHIIGFYQAVSTDAKLRDASTEAEKLMDDFSIESSMREDIFKLVDAAFKKQKDDQKLDAESRRLLEKDHKGYIRMGLGIPAGPDRDRFKEIKKRLSELSITFQKNLNEEKGGLWLTPEELDGVPEDVTSGLQKGKEGTPSEGKLWLTFKYPDLFPTLKYAKNEATRRKVFVANENKCPENIELFKEAIVLRDESARLLGYPNHATFRIEDKMAKTPKTVDDFLGDLRKRLAPGGLKEIEKLKELKKKQGADGDNYYLWDHRFYDTLMLERDYQLDQNRISEYFPIQTSISGMLNIFEEIFGLHFVEVQGEHRDQISETGKGDDIVWHPEVQVFSVWDDESEGGSFVGYLYLDLFPREGKYGHAANFNIQPGFITENGTRRYPATALVCNFSKPTPKKPSLLKHDEVVTLFHELGHGIHDLVSKTTYSRFHGTSTVRDFVEAPSQMLENWCWTPSQIKSLSKHWSHLSPEYEAAWKETGKEKPAQSEMPDDLIQSIISTRHVNDGLFNLRQLHFGIFDMTVHEAPSHDWVKGLDASETYNKLRSEISRLDGPETIGEGWLWGHGQATFGHLIGGYDAGYYGYLSSQVYSADMFYTIFKADPMNGKEGRRYRHMVLEKGGSQDEMLTLEQFLGRPPSTEAFYKELGLS |
| 135 | Unigene0010495 | 101 | 14343 | 9.8 | MSWQAYVDTSLVATGNVDRAAIFNSEGNSVWATSAGFTVKPEEMQAVVAAYKDKGDEQGVKKVQSEGLHIAGERFVVIKADDRSIYGKKGREGVVLVKTTQAILVTHYPETVQPGSAANTVEQLGDYLIKVGY |
| 135 | Unigene0010494 | 101 | 13436 | 10.3 | SSSLVATGNVDRAAIFNSEGNSVWATSAGFTVKPEEMQAVVAAYKDKGDEQGVKKVQSEGLHIAGERFVVIKADDRSIYGKKGREGVVLVKTTQAILVTHYPETVQPGSAANTVEQLGDYLIKVGY |
| 136 | Unigene0009736 | 100 | 56343 | 5.6 | MASKASVQRPADFVDFLNASPTPFHAVRSAKERLENAGFKPIRERDSWNATLQPGGKYYLTRNTSTIVAFAIGTEWKPGNPIAMVGAHTDSPCLRIKPVSKRSAEGFLQVGVETYGGGMWHSWFDRDLGVAGRVMVKGKDGVVEQKLVRIHKPIARIPNLAVHFGGYEPFTFNKETQLFPVLGLISAELNRTGKSKDELKKEADEEEKAAGFEPLKTANQRHHPYLISLIAQDAGVDTQDVLDFELVLYDMQPATIGGINDEFVFSARLDNLGMTYCAVEGLIQSVASASALKGDPTIRLIACFDHEEIGSQSAQGADSNMLPSVIRRLSVLPASASDSEKSYDKVSNGDSADTSTAYEQTLSTSFLVSADMAHSVNPNYGGNYEPEHRPHMNEGTVIKINANVRYATNSPGIVLLQECAKRAKPTSWQLPEAKTKDGGVPLQLFVVRNDSRCGSTIGPMLSAALGARTIDVGNPQLSMHSIRETGGAYDAEHGVNLFDSFFEHYGELESKILVD |
| 137 | Unigene0014300 | 100 | 41963 | 8 | MSDTAGKTITCKAAVAWEAGKDLVVEDIEVLPPRAHEVRIEVYYTGVCHTDAYMLSGKDPEGAFPVIAGHEGAGIVESVGEGVTNVKVGDTVIALYTPECKECKFCKSGKTNLCGKIRATQGKGVMPDGTSRFKCKGKDLLHFMGCSTFSQYTVLADISVVAVTPDAPMDRTCLLGCGITTGYGAAVITAGRGGVEKGSNVAIFGAGCVGLSVIQGAVKQGSSKIIVVDVNDAKKEWAKKFGATDFVNPAKDLKEGENIQSRLIDMTDGGCDYTFDCTGNVHVMRSALEACHKGWGESIIIGVAAAGQEISTRPFQLVTGRVWKGCAFGGVKGRSQMGQLVQDYMDNKLKVDEFITHRQPLGGINDAFHDMHAGDCIRCVVDMRKV |
| 138 | Unigene0014668 | 99 | 37318 | 6 | MGSIEETHGIFRFVDPDLSVSAYDRAIFANPRPKRLEQLKLPLHNFWTSDEVAKGNAGLDTQGFTYIKHASALTAENVFESNNVEDVLVPETVDMMLKLTGAKRGVVHNVTFRRKSAAKQENLDFIPMRGNELDQILAKMPKNQLLVNGKDVQSSSEPARQCHVDLTLKGLRTTLRSATTEIAEAARDVIEAEDLRASGADVSVPRCASYSIWRPLQTVKRDPISICDYRTLDMSELIPTENRIPSGINPEGDYVVEAYTAQPPKRPELQKWYWVPEQKPDEVCIIKFADTAAESDPSIAACGLHVSPVLPGTENEEIRQSSETRVYLFWE |
| 139 | Unigene0001071 | 98 | 36556 | 14.4 | MPIPDHFVLNTGAKIPAVGFGTWQAAPHEVENAVEEALKQGYRHIDGAAIYQNENEVGAGIKKSGVPREQIFLTGKLWNSKHRPEDVEGALDKSLKDYGTDYLDLYLMHWPVAFKAKDKFFPIDEQGVFEIDEEYQTDDKLAETYNAMVKLLSTGKVKAVGVSNFNKRRLEHLLARTDVVPAVNQIEAHPYIQQPELFEYAKSKGIIIEAYSPLGNNQTGEPRTVDDPKVHEIAKALEMDPGAVLAAWAVQRGQVVLPKSVTPSRIAANLKIKELPQEHFEALNALERGKRFNYPKHWGVDIFEEGKGEDFKKFALDAAEANKKKF |
| 140 | Unigene0014312 | 97 | 52743 | 6.7 | NANTSQFVVDGTALPDVDFDIGQAFAGQLPISQEANETRKLYFFFSPSSDPAASDEILIWLNGGPGCSSLAGFLQENGPLSWKYGTYKPVPNKFSWNKLTNMVWLDQPLGTGFSNSEPTDQSEADVARNLMGWLRNFIDTFGFHGRKIYLTGESYAGFYISYTANAMHEANDTTYFNLQGALLYSPAVVWSSVSQLIPSVPFVDHWASVFNLNETFMTSIRNASEACGHREWMETHLTFPPPQGPFGTPPDGLEYRDGCDTWSAILDAVMLVNPCFNSYHVTDTCPILYDPLGFPYTFQYVAPGAQVYFNRSDVQRAINAPEISWSTCRTDVLTSDASEWPSLADAGVDRVPLAAMIEKNNRTIIVAGDSDFQFSVNGTLIAIQNMTWNGAQGFQSRPSKPFVVPYFDQDICDSSLLGSEQCGDANFAGAGILGTTHSERGLTWVEQSGAGHAVPQYNGAAAYRHLEFLLGRIPSL |
| 141 | Unigene0005110 | 96 | 22321 | 8.6 | AGNRYUHIATTAFSRDFKKKGMIALSTYLKQYRVGDIVDVVANGAVQKGMVGGUMDTHFLSLVVSVLWLSRHTLQAPPCGERLTPAFQUPYKVYHGKTGVVYNVTKSAVGVILYRQVGNRYIEKRINVRVEHVRHSRSRDEFLARVKTNAAKQRQAKETGEHVHLKRQPAQPREARTVSAKDNKPENVAPIAYETTI |
| 142 | Unigene0013343 | 96 | 6180 | 28.3 | IIFALSAILGLAMAFCSKDDCCFKDNVARDKHNGDAFYCDDAVRKDCNADCCS |
| 142 | Unigene0013341 | 96 | 6289 | 28.8 | LISYSILTCIQMAFCSKDDCCFKDNVARDKHNGDAFYCDDAVRKDCNADCCS |
| 143 | Unigene0002708 | 95 | 38171 | 5.4 | MAPQVPEMQWAQVIEKTGGPVEYKQIPVQKPGPDEILVNIKYSGVCHTDLHAVNGDWPLPTKLPLVGGHEGAGEVVAMGELVHDWQIGDYAGVKWINGSCLSCDFCQTSDEPLCGKALLSGYTVDGSFQQYAIAKAAHAARIPKECDLAAISPVLCAGITVYKGLKESGAKPGQTVAIVGAGGGLGSLAQQYARAMGLRTIAIDSGDEKKKMCLEQLGSYAFVDFAKSQNVVKDVQAATEDGLGPHAVILVAVNEKPFQQAAEYVRPRGTVICIGLPAGAYLRAPVFESVIKMVTIKGSYVGNRKDSSEAIEFFRRGVIKAPFKIVGMSELQKVYDLMHAGQIAGRYVLDTSK |
| 144 | Unigene0004716 | 95 | 74640 | 3 | MNVLKLQKKYPQFPQAEIYGLSDAFRKLDVDDKGYVDEGTAIKSAQNSERQPYDVVRQALKEVELDSSRRVELDDYVDLIARLRQSSPAQQRTRGFSNAAQKTGDGAAAPSPSHASKGSIGGGAQARAGRITVGGSTASSQHTINEDERTAFTSHINAVLAGDPDIGHLLPFPLDTFEMFDSCKDGLVLAKLINDSVPDTIDERVLNRPGKKIKSLNNFHFTENNNIVIESAKGIGCSVVNIGAGDIIEVREHLILGLIWQVIRRGLLGKIDIKLHPELYRLLEDGETLEQFLRLPPEQILLRWFNYHLKNAGWHRRVQNFSNDVKDGENYTVLLNQLAPQVCSKAPLQTSNLEQRAEQVLQNADVLDPPCRKFLTPKSLCAGNPKLNLAFVANLFNNHPGLDPITEEEKAEIEDFDAEGEREARVFTLWLNSLDVKPSVVSFFEDLKDGIVLLQAFDKVIPGSVNWRHTNKPPANAVAPVSQDEDEAYLTIKSGMSRFKAVENTNYAVEIGKQNKFSLVGIQGADITDGQKTLTLGLVWQLMRRDITNTLQGLAQRLGKREITDSDMVKWANDTVKKGGRSSAIRSFKDPQLASGVFLLDVLNGIKSEYVDYDLVFSGRTDEEAYANAKLSISIARKLGATIYLVPEDITSLRSRLIMTFIGSLMATAER |
| 145 | Unigene0006795 | 94 | 70869 | 5.2 | MAPAIGIDLGTTYSCVGIFRDDRIEIIANDQGNRTTPSFVAFTDTERLIGDSAKNQVAMNPVNTVFDAKRLIGRKFNDPEVQADMKHFPFKVIDKGGKPIIQVEFKGEEKTFSPEEISSMVLTKMRETAESYLGGTVNNAVVTVPAYFNDSQRQATKDAGLIAGLNVLRIINEPTAAAIAYGLDKKTEGERNVLIFDLGGGTFDVSLLTIEEGIFEVKSTAGDTHLGGEDFDNRLVNHMVNEFKRKNKKDLTVNARALRRLRTACERAKRTLSSSAQTSIEIDSLYEGIDFYTSITRARFEELCQDLFRSTMEPVERTLRDAKIDKSSVHEIVLVGGSTRIPKIQKMVSDFFNGKEPNRSINPDEAVAYGAAVQAAILSGDTSSKSTNEILLLDVAPLSLGIETAGGVMTPLIKRNTTIPTKKSEVFSTFSDNQPGVLIQVFEGERARTKDNNLLGKFELTGIPPAPRGVPQIEVTFDVDANGIMNVSALEKGTGKTNKIVITNDKGRLSKEEIERMLAEAEKYKEEDEAEAARIQAKNALESYAYSLRTTLSDPKTEEKLEASDKETLKAKIDETVSWLDDNQTATKDEYESSQKDLESVANPIMMKLYGAGGEGGMPGGMPGGAGFPGAGGPGGPGGAGGDDGPTVEEVD |
| 146 | Unigene0006597 | 93 | 11193 | 19.6 | SQAVNAGGLIFVSGQIPAKADGTLVTGSIADQTTACIEGLKNILEAAGSSIAKVTKVTVFLTSMDNFAEMNSVYEKYFGEHKPARSCVAVKELPKGVPVEVEAIALA |
| 146 | Unigene0006596 | 93 | 11193 | 19.6 | SQAVNAGGLIFVSGQIPAKADGTLVTGSIADQTTACIEGLKNILEAAGSSIAKVTKVTVFLTSMDNFAEMNSVYEKYFGEHKPARSCVAVKELPKGVPVEVEAIALA |
| 147 | Unigene0012914 | 93 | 68807 | 3.4 | LTQCSPILNTRQDAQYEGYAFAYFTGNSIDGEKIFLAASNGNNALDWRELNGGQPVLSSNQGTKGLRDPFVIRSNDGTKFFLIATDLSIGSGTSWGDSVRTGSRYLEVWESEDLVNWSEQRHVLVSPPTAGNTWAPEAYYDEDVGSYVVYWASSLYAEDDTNHTGNTYHRMMYATTQDFVTFSEAKIWQDAGTSRIDSTVLKSGDTFYRFTKDEGGVTGCTDIIQESSSTLLSQLDGWTIAASCIGRDAGTQAIEGPTAFRANPGDVNGDKFYLFVDEYGGRGYIPLETADVANPDWKVSPSYKLPTSPRHGTVIPVTAAELQRLTSALAKRSENKLQKRSTLIPGLYADPNIAIVGCEYYIYATTDGFPGWGGQQFFVWKSSDLISWTRSDTAFLTLNGTNGNVPWATGNAWAPGFIERNKKFYFYFSGQNPTYDRKTIGVAVSSSPEGPFTAEPSAMILNNEAITSGQAIDPQAFHDPITGKFYLYWGNGNPVMAELASDMKSIIPSTLQSISGLTDFREASFVVYRAPYYHFTYSIDDTGSPNYRVGYATGPSATGPWTYHGVILEKDESQGILGTAHNSIVNVPGTDEWFIAYHRFSIPDGDGTHRETTIDKLEFGEDGL |
| 147 | Unigene0012913 | 93 | 38201 | 6 | MRSFVVQLLTAATSLATLTQCSPILNTRQDAQYEGYAFAYFTGNSIDGEKIFLAASNGNNALDWRELNGGQPVLSSNQGTKGLRDPFVIRSNDGTKFFLIATDLSIGSGTSWGDSVRTGSRYLEVWESEDLVNWSEQRHVLVSPPTAGNTWAPEAYYDEDVGSYVVYWASSLYAEDDTNHTGNTYHRMMYATTQDFVTFSEAKIWQDAGTSRIDSTVLKSGDTFYRFTKDEGGVTGCTDIIQESSSTLLSQLDGWTIAASCIGRDAGTQAIEGPTAFRANPGDVNGDKFYLFVDEYGGRGYIPLETADVANPDWKVSPSYKLPTSPRHGTVIPVTAAELQRLTSALAK |
| 148 | Unigene0002746 | 92 | 16140 | 18.9 | MSAVQSVQCFGKKKTATAVAHTKQGKGLIKVNGKPLSLVEPQILRFKVYEPLLILGLDKFADVDIRVRVRGGGHTSQVYAIRQAIAKSIIAYYQKYVDEHSKNQLKQALVAYDRTLLVADNRRCEPKKFGGPGARARYQKSYR |
| 149 | Unigene0013250 | 92 | 15201 | 5 | XSFEIFSLIYKTPIQKMHFSVIVCSLTAFSLGVLAVPTQDPKTKDLIKEKSTACFKDLDAINAGTSDDTATAAACDSAMAECGAGLESIGPSYTNSGSLTCAYDPKICKSAQKHIENICRKDYDMVGYTSHGLSEPTEG |
| 150 | Unigene0012587 | 92 | 52943 | 7.7 | NSSSHCCRALEDAGLQSALIYPDDVTTFEASVGKYFSQVAQLSPQCFLQPGNTAEVAQAVTALVAANQTRHCQFAVRSGGHISWPGAAGIEDGVTIDLGRLKEISYSSKDNTVSVGPGAVWGEVYQALDKLNVMTTGGRSSSVGVSGLTIGGGNSYFAAQYGFVCDNVASFEVVLGNGTTVTASKDDNPDLFRVLKGGNLNFGIVTKIVLNTFPGGPFFGGNVIYPADTADQQFKALVNFGNGINDDRHASTIVIGYHDSEKNSTVFLNAYEYTKPVQRPDAKIFKEFFSIPGNISDTTGLRNMTSLAHEFEAPKDRRVQFATLTFKSDIRVLQRAHESFLKVIEELNGRMNGGYFQAWTLYQPIPTIFAKHGLEKGGNVMGLDRYDDTLIMYETYFRWEETENDQVIEEQSQWLRDDIQSFAASVGADADWLYLNYADKDQKALEGYGAENVAKIRAAAQLYDPQGVFQNMVPGGFKISRV |
| 150 | Unigene0012588 | 92 | 52943 | 7.7 | NSSSHCCRALEDAGLQSALIYPDDVTTFEASVGKYFSQVAQLSPQCFLQPGNTAEVAQAVTALVAANQTRHCQFAVRSGGHISWPGAAGIEDGVTIDLGRLKEISYSSKDNTVSVGPGAVWGEVYQALDKLNVMTTGGRSSSVGVSGLTIGGGNSYFAAQYGFVCDNVASFEVVLGNGTTVTASKDDNPDLFRVLKGGNLNFGIVTKIVLNTFPGGPFFGGNVIYPADTADQQFKALVNFGNGINDDRHASTIVIGYHDSEKNSTVFLNAYEYTKPVQRPDAKIFKEFFSIPGNISDTTGLRNMTSLAHEFEAPKDRRVQFATLTFKSDIRVLQRAHESFLKVIEELNGRMNGGYFQAWTLYQPIPTIFAKHGLEKGGNVMGLDRYDDTLIMYETYFRWEETENDQVIEEQSQWLRDDIQSFAASVGADADWLYLNYADKDQKALEGYGAENVAKIRAAAQLYDPQGVFQNMVPGGFKISRV |
| 151 | Unigene0000385 | 92 | 22573 | 11.9 | LLSDSADIVCPTVQSASAGYKGLEEYQVIGRHLPSDANPTPKLYRMRIFAPNDVVAKSRFWYFLAKLRKIKKANGEIVSLNKIHEKHPLKVKNFGIWIRYDSRSGTHNMYKEYREMSRVDAVKSLYQDMASRHRARFRTIHILKVVEVEKTDDIRRPYIKQLTTKNLKFPLPHRAPPKRGKKVFAATRPNTFY |
| 152 | Unigene0001594 | 91 | 12551 | 9.9 | MGKDEFKDGDAKKGANLFKTRCAQCHNLKAGEPNKIGPNLHGLFGRQTGQVEGFSYSDANKQKGITWEEGTLFEYLENPKKYIPGTKMAFGGLKKGKDRNDLITYLREETK |
| 153 | Unigene0001883 | 91 | 12090 | 40.7 | MRGNDPQTKVHYKGDNDDFIVLVESAKAVQDWKKDSSVPLAQVVAGFKIFLTHNQGNTGVLDTASKGALEGEFGTSNEDDVIKQILEKGNIVESGNSGRDGVKNVTQGPMVGH |
| 154 | Unigene0013759 | 91 | 56502 | 3.1 | MRYSSSILAISAATLTTALPQYEPYPGEPKDESQQRANAVKQAFEFSWQGYSEYAFPNDELLPVTNGFSNSRNGWGASAADALSTALVMGEKEIVNQIIDYVPLIDWSVSADDEVVSLFETTIRYLGGLLSGYDLLSGPLADLADNKDNVASLLVQAQNLANNLSFAFDTPTGIPYNQIFVGNRTNDGSDTNGLATIGTLILEWQRLSDLTGDPTYGNLASRAESYLLNPSPPSAEPFPGLVGSEIGINNGSFTNARGAWTGGTDSFYEYLIKMYVYDTSRYAEYRDRWVLAADSSIQYLASHPSSRPDLTFLADYNGQRLINGSQHLACFDGGNFILGGLVLNEQKYTDFGLELVEACEETYSQTLTGIGPEVFSWNTTTLPANQTEFYERAGFWIVAGSYVLRPEVIESFYYAYRVTGDRKYQDYAWNAFVAISETCRVGSGFSSISDVNAPNGGSFDNFQESFWFAEVLKYSYLIHAPDAEYQVNHEGKNEYVFNTEAHPVKVAGKPI |
| 155 | Unigene0010115 | 90 | 16784 | 12.6 | MGRLHSNGKGISSSAIPYSRAPPAWLKTTPDQVVDQICKLAKKGATPSQIGVVLRDSHGVAQVKIVTGNKILRILKGNGLAPEIPEDLYMLIKKAVAVRKHLERNRKDKDSKFRLILIESRIHRLSRYYKTVGALPPTWRYESATASTLVA |
| 156 | Unigene0010297 | 90 | 28993 | 13.6 | TLFGGDNGWHSDDWIASDDRVRGGKSISYLDCTKTTGTFRGTLDIKTLGGAGFASQRTTGDDREWDLSDYAGIELYIAKGDKKRYTFNLKDELLPQDPDTGREQSSISYECDFELPPQTVPGDAHDKTIFIPWSSFNATYRGRPKKDAKPIDLKKIKRFSIMMRSFFGTQEGDFSLSIRSISALQEVPKDTNTDAEGESWLDASLEKGYNSASQRHVPRGSVSSNTSLWQRRKGTCLLLVAVSVLAVYHLAGKYLHNC |
| 157 | Unigene0001554 | 89 | 79572 | 5.2 | MAMMKSFTRISAAAGLVSVVGAQTFQRFGTCPTLGCVIPPDQQDFLAGQFFDIRVEVHAPVNGSEATNGIPDEDFTLTIRKDGGEARPVTEYFELEEPEIEKWDFSWYEDLFALDAETPSAVNVASKAWRRVALYEPGEYTVVLTYNNGTNTTANWLVRDIEQVRKTKNILLFIGDGMTTNMVTAARLIAHKTINGRYQSTLALDKFPTIGHQMTHSIDTFITDSANSATALYTGHKSVVNALGVYGDTSPDPFDDPKVESIAEIFHRLVGGHVGIVSTAFVADATPAALTAHTRDRGEYGAVIDSFVNGIVNYTWTEWDGPDVLFGGGAENFCSPDNGGETYQDLDYYKVFADAGYNVIYNKTGLDATSSEERTLGIFSQSNMAKWLDRNVYTDNLNDTESPLCNGETAADQPGLKEMTIKAIDILNNRAGDKGWFIMSEAASVDKMMHVLDYDRALGELLELDDTIKHSIEHLKEIDAYKDTLIVVTADHGHGFDVFGNVDTKYLNEKTDDREKRNAVGIYERSGLSGYINTGDLRYGDSNFPSNWEPRYTLAQGFGAFPDHRETFQVHKDGPRSPASNITGFATDNYFADPKNDEGGFVVNGTLPESANQGVHSLTDVTVWAQGPCSEIFGGVYNSIDVFFKMAECLGLSEKRGGNGTAPGYGGHQKEKKDNWEKCEGKKGYQRYDEKHPKYAGEEHGHNTGVKPRSFKPGHAYC |
| 158 | Unigene0003851 | 89 | 17519 | 10.5 | MADADYNAEEAAELKRKRAFRKFSYRGIDLDELLDLSSEQLRDVVHARARRRFNRGLKRKPMGLIKKLRKAKQEAKPNEKPDVVKTHLRDMIIVPEMIGSVVGVYSGKEFNQVEIKPEMVGHYLGEFSISYKPVKHGRPGIGATHSSRFIPLK |
| 159 | Unigene0012760 | 89 | 71952 | 5.3 | SALLFTLTSARVPRKFTRQATNETQPYQLQTPPLDTDYTSSVGTNPWPQHPRPKLKRNQWQNLNGIWQYTNASSLDAVNNPPFGQNLAREVLVPFCLESGLSGIQGKDLFYSWYRTTFDVPTDWTGDRVLLNFDAVDYEATVFVNGQNVTFHRGGYSAFSVDITDALQAGDNGTNELVVFVHDPTDSDPYVIPIGKQTLRPSHFFYTPCSGIWQTVWLEAVPTNYISSLDINGDADGNLDITVISADNSTSDVEVTVYEKNTTNSVATGGGNSGSAFSIKVDSVRPWTPESPTLYDVVVKLGDDEIQSYSGFRTISRGEVNGVQRILLNGASYFPFGTLDQGYWPDGLYTPPTYDAMVYDLEVLKDIGYNMLRKHIKVEPPLFYEATDRLGLLVMQDMPSLRPSQSKTLEDCTSVTILPDAAQQQEFQRQLEVLVTQFRSYTSIFSWVIYNEGWGQIRTQPYPEFGLTDVVRRLDPTRLVNANSGWYDHGAGDFSDNHHYANPQCGTPWYSIDSSPHDPSRIGFQGEFGGTGHNVSAENLWKVEQAINRINQTYEIDETLDIWNYRGHFLLNELLSQVELYSCAGGVWTQTTDVEGEVNGMLTYDRRIIRPDLEQWNRDIQALYNANAARSNATAPTF |
| 160 | Unigene0003202 | 88 | 14935 | 18.7 | MAPKAATKTPTTAGKAPAGKAPAEKKEAGKKTAAPSGEKKKRTKTRKETYSSYIYKVLKQVHPDTGISNRAMSILNSFVNDIFERVATEASKLAAYNKKSTISSREIQTSVRLILPGELAKHAVSEGTKAVTKYSSSTK |
| 160 | Unigene0003201 | 88 | 14265 | 19.7 | QTPTTAGKAPAGKAPAEKKEAGKKTAAPSGEKKKRTKTRKETYSSYIYKVLKQVHPDTGISNRAMSILNSFVNDIFERVATEASKLAAYNKKSTISSREIQTSVRLILPGELAKHAVSEGTKAVTKYSSSTK |
| 161 | Unigene0010012 | 88 | 63431 | 4.9 | AVMKCFIAAAGLVCLTSAAPRVQTPHQRPDSYGIPSVTVKNGTIAGVHKSNYKQDYFLGVPFAQPPVGPLRFTNAQSLNSSFDGTLQATEYAPMCYGYGGDQIGYPQSEDCLYLNVVRPSGYENASLPVGVWIHGGGLYMGGTQDRRYNLTWIVQNSVEIGKPIIGVSIAYRLGPWGFLASQEVTGSGNTNIGLRDQRLALHWINENIDAFGGDKDKVAIWGESAGAGSVGFHLTAYNGRDDGLFRAGIMESGNPVNYNSYATNARYQPKYDALVNATNCSTETDTLGCLRGIPEQTLQVLFNTTSSLSSGWNPVVDGDFIQRWASIQLKEGDFVKVPIIDGANTDEGTSFGPDGIDTDEEFAAYITNSSQLTSLPAALAPRALEAYPNEPAYFIPPVEEVPLNYTYPPENGAQYRRANAYAGDVAFHANRRGAMEAWAANNVSAYSYRFNTLPTGVPWYAGVVHFQEVAFVFDNTQGLGYDAEHGTVNPFQNKSRSYYDLADYMSKSWVSFIHDLDPNYEAKPASAPAWPQYSLDQPENIVWDANRTELAWLEPDTYRQEGIRFILDNALAYKR |
| 162 | Unigene0006813 | 88 | 83305 | 6.5 | MKGVVYAAGALLSLASTARSIGYTNVSEVPLYGQSPPVYPSPKGNGGSDPKWQAAYVKARSIVNQLTLEEKVNMTRGHTGLCVGNTPNITRLGIPALCFADAPDGIRGQEFVSAFPAQLTVGATFDKRLMYEYGKALGEEYRGKGIHVALLPVAGPLGRVARGGRNWEGFGADPYLSSVGMREVTTGLQDQGVISQMKHWLLNEQEWRRNPGSMGESTSSNADDRTIHELYAFPFMDAVHAGAASAMCSYQRINNSYGCQNSKLLNGILKGELNFQGFVTSDWAAQHAGVASANAGLDLVMPDGGYWGDNLTQAVTNGSVTETRLNDMVTRILAAWYHLGQDAGYPEIGVYPYDQQHPIIDVRYDHASLIRAIGAAGTVLVKNKDNALPLKNPRFLNIYGYDAKAPDSPWTTPSRFGGGYDVNFGWEIFNGTLVTGGGSGGSSPSYFISPFDAIQRRVIEDRGTVRWDFASVNPTVYANADACLVFINAYSSEAFDRTTLKDEFSDNLVKNVARNCTNTIVVLHSVGIRVIDEWVNNENVTAIIYALLPGEEAGNSIADVLYGDVSPSGRLPFTVAKNESDYGNLLNSTIGSGPFPQDNFTEGLYIDYRYFDQKNIEPELEFGFGLSYSAFEYSGLSITPVSNTTQEWPTPDTPIPQGGHPEIWETLFRITVQITNTGDVAAAEVPQLYISIPTAPKWQLRGFDRLYLEPDQTKTATFKVTRKDLSVWNVFHQQWQLQQGPYDLFVGSSSRKPLLQGTL |
| 163 | Unigene0013528 | 88 | 32307 | 12.2 | LRSILLAAAILNITTALPGLLCQTQSQPNPIAKDRPTEVTGTINGTSAIVPIPYDVARSVVPAQYGILREAYEKLIPGFPKDMYPAEFEGLLDHDVQSFGIKIPDFQRMALRFPFVDRLNDGYSCFRYTAPQLVSVSNPVAIAGSTAYGKTIPATFDPPCDGYANDKTGSTYLSGYAVPESKLLGSEPTFDSRFRSVVSIPYNQKLLVNITNQPSFGSGLPVCDNYITLYNTSVTQGRFAPVPVQGELKVEPPYYPQKTTLKAWGYRMDNAFIEKNNVPCESLKGYSGTGPGDS |
| 164 | Unigene0001382 | 88 | 60659 | 4.7 | MSVKTVQTKPFGDQKPGTSGLRKKVKVFQQQNYSENFVASIILSIPEGAQDAFLVVGGDGRYWNPEVTQVIAKIGAAYGVKKLLIGQNGILSTPAASHLIRVKKATGGILLTASHNPGGPEEDFGIKYNLTNGAPAPESVTNKIYETSKTLSEYKIADIPDIDLSTIGTKTYGPLEVEIVHSTEDYVKMLKDIFDFDLIKKFLKNNPDFKILFDGLSGVTGNYGVDVFEKELGQKGSTQNCVPKPDFGGHHPDPNLVYAKSLVDRVDKEGIHFGAASDGDGDRNMIYGANSFVSPGDSLAIIAHYADLIPYFKKQGVYGLARSMPTSGAVDLVAKKKGLKSYEVPTGWKFFCGLFDSDKMNICGEESFGTGSNHIREKDGLWAIVAWLNIIAGVGEQTGSTPSINSIQHDFWNIYGRTFFTRYDYEGVESEGANKVVAHVKELITTKKDSFIGSSISGRKVVEADDFSYTDLDGSVSKNQGIYVKFDDGSRIVVRLSGTGSSGATIRLYIEKHENDKSKYGLDAQEYLKDNVKLATDLLKLQEYVGRTEPDVKT |
| 165 | Unigene0013773 | 87 | 124261 | 3.5 | MGGETHSTNTMTSSTAQYPQLAARPKGKLIHDIYRDRLNQFTSGGQYKEQGLLGKLYNGRLGEGHVHLSVWSAPDLTRPTFAEATDSKNTYKKTRKGESFGPSWSTHWFRVDFTVPTEWKDEERVELHWDSSSEGLVWTHDGKPLQGLTGNGERIEWIVPDSYRTGKEETIYIEMACNGMFGNPTGGDTIQPPDPNRYFRLQLADLVSVNLDARALFYDFWILGDAAREFPQDSWEEHRALTIANEVIDAFIAGNGSKEAIKECRKIAKKYVGDKVDGPEVYELTEKDPLREVLVSGIGHCHIDTCWLWPWAETKRKVVRSWSNQCDLLDRYPEHRFCASQAQQYKWLEELYPYAFDRVKNHVKKGNFQPIGGSWVEHDTNMPSGESLVRQFLYGQRYFESRFGQRCRTFWLPDTFGYSSQIPQLCRLAGMNRFFTQKLSWNNINNFPHTTFNWVSLDGSQVICHMAPSETYTAEAHFGDVKRSVTQHKSMDQDPTSLLVFGKGDGGGGPTWGHIEKLRRCRGIADNVGLLPRVHMGRSVEDFFDRLEKNAATGKTEFVTWYGELYFELHRGTYTTQANNKKGNRKSEVLLHNIEYLATMASLHDKDYKYPKMEIDSMWESVLLCQFHDCLPGSSIEMCYDDSDKEYAKVFKTGQKVLHEALKSLGFVDDKTAQPVAINTLGWPRTDQELVESRYPEVVGQPAGTQTLIKRATEEASVEELDDGAFSLQNSKLALTVADGVITSLYDKAADREVLPKGGKANQLVLFDDKPLYWQAWDVEVFHLESRQELKATSKTEITENSTQRVAVTTKTQISDKSWIKTSIALDATSDGAETSYVQVEAEVEWHETMKFLKVEFPTTITNTEASYETQFGINRRPTHYNTSWDMAKFEVCCHKWADLSEHGYGVSILNDSKYGFATTGKLMRLSLLRAPKAPDAHADMGHHKIRWAILPHAGPLDHRTVRAAAEFNNPMIAHRHPEVQSVKELFSAFKLTANSSPSLVVDTVKRGEDDADVSVDDLRVKDGRHVVLRIYDSLGGTSRGTIALGPIKVHKAWKSNILEDEVEEVKVGKNGIDIELRAFEVATYKLLV |
| 166 | Unigene0014825 | 86 | 132633 | 3.2 | MSDSNARDESAIEDGDYGTQDPHGETHHQKNTVHQRLRANSSIMQLNKILVANRGEIPIRIFRTAHELSLQTVAVYSHEDRLSMHRQKADEAYVIGKRGEFTPVGAYLAGDAIIKIAKEHGVNMIHPGYGFLSENYEFAKAVEAAGIIWVGPEPKTINDLGDKVSARNLAAKADVPTVPGTEGPVAKFEAAKEFTDEYGFPIIIKAAFGGGGRGMRVVWKQEDLKDSFERATSEAKSAFGNGTVFIERFLYRPKHIEVQLLGDNYGNVVHLYERDCSVQRRHQKVVELAPAKDLPQETRDAILNDAVRLAKSANYRNAGTAEFLVDQENRHYFIEINPRIQVEHTITEEITGIDIVAAQIQIAAGASLAQLGLTQDRISTRGFAIQCRITTEDPSEGFKPDTGKIEVYRSAGGNGVRLDGGNGFSGAVITPYYDSMLVKCTCHGSTYEIARRKVLRALVEFRIRGLKTNIPFLASLLTHPTFIDGTCWTTFIDDTPELFNLIGSQNRAQKLLGYLGELIVNGPQVIGQIGESKFKGEAIIPTLAGDDGKPLELDGPCQKGWRNIITEKGPEAFAKAVRQNKGCLIMDTTWRDAHQSLLATRMRTIDLLNIAKETSYALSNAWALECWGGATFDVAMRFLYEDPWDRLRKMRKAVPNIPFQMLLRGANGVAYSSLPDNAIYHFCEQAKKNGMDIFRVFDALNDMNQLEVGVKAVLKAGGVAEGTVCYSGDFLNPKKKYNLEYYMTCVDKIVEMGAHILGIKDMAGVLKPRAATLLIGTIRKKYPDLPIHVHTHDSAGTGVASMVACAQAGADAVDAATDSMSGTTSQPSIGAIVASLEGSDFESGLNPVHLRAIDSYWAQIRMLYSPFEAWLTGPDPEVYEHEIPGGQLTNLIFQAAQQGLGEQWAQTKKAYEQANDILGDIVKVTPTSKVVGDLAQFMVANSLSYDDVHEKAEELDFPSSVLEFFEGLMGQPYGGFPEPLRSKALRDRRKMDKRPGLYLDPINFDKVRETLKEKYGGCSETDVASYVMYSKVYEDYKKWTSQFGDLSVLPTRYFLNKPEIGEEFHVELEKGKVLILKLLATGPLSDQTGQREVFFEVNGEMRQVTVQDKHAAVENTSRPKADSGDSSQVGAPMSGMVVEIRVKDGGEIKKGDPIAILSAMKMEMVISSPHSGKIEDLSVKEGDSVDSSDLICKIAKS |
| 166 | Unigene0014824 | 86 | 132633 | 3.2 | MSDSNARDESAIEDGDYGTQDPHGETHHQKNTVHQRLRANSSIMQLNKILVANRGEIPIRIFRTAHELSLQTVAVYSHEDRLSMHRQKADEAYVIGKRGEFTPVGAYLAGDAIIKIAKEHGVNMIHPGYGFLSENYEFAKAVEAAGIIWVGPEPKTINDLGDKVSARNLAAKADVPTVPGTEGPVAKFEAAKEFTDEYGFPIIIKAAFGGGGRGMRVVWKQEDLKDSFERATSEAKSAFGNGTVFIERFLYRPKHIEVQLLGDNYGNVVHLYERDCSVQRRHQKVVELAPAKDLPQETRDAILNDAVRLAKSANYRNAGTAEFLVDQENRHYFIEINPRIQVEHTITEEITGIDIVAAQIQIAAGASLAQLGLTQDRISTRGFAIQCRITTEDPSEGFKPDTGKIEVYRSAGGNGVRLDGGNGFSGAVITPYYDSMLVKCTCHGSTYEIARRKVLRALVEFRIRGLKTNIPFLASLLTHPTFIDGTCWTTFIDDTPELFNLIGSQNRAQKLLGYLGELIVNGPQVIGQIGESKFKGEAIIPTLAGDDGKPLELDGPCQKGWRNIITEKGPEAFAKAVRQNKGCLIMDTTWRDAHQSLLATRMRTIDLLNIAKETSYALSNAWALECWGGATFDVAMRFLYEDPWDRLRKMRKAVPNIPFQMLLRGANGVAYSSLPDNAIYHFCEQAKKNGMDIFRVFDALNDMNQLEVGVKAVLKAGGVAEGTVCYSGDFLNPKKKYNLEYYMTCVDKIVEMGAHILGIKDMAGVLKPRAATLLIGTIRKKYPDLPIHVHTHDSAGTGVASMVACAQAGADAVDAATDSMSGTTSQPSIGAIVASLEGSDFESGLNPVHLRAIDSYWAQIRMLYSPFEAWLTGPDPEVYEHEIPGGQLTNLIFQAAQQGLGEQWAQTKKAYEQANDILGDIVKVTPTSKVVGDLAQFMVANSLSYDDVHEKAEELDFPSSVLEFFEGLMGQPYGGFPEPLRSKALRDRRKMDKRPGLYLDPINFDKVRETLKEKYGGCSETDVASYVMYSKVYEDYKKWTSQFGDLSVLPTRYFLNKPEIGEEFHVELEKGKVLILKLLATGPLSDQTGQREVFFEVNGEMRQVTVQDKHAAVENTSRPKADSGDSSQVGAPMSGMVVEIRVKDGGEIKKGDPIAILSAMKMEMVISSPHSGKIEDLSVKEGDSVDSSDLICKIAKS |
| 167 | Unigene0012611 | 85 | 32462 | 11.6 | RTTYRNRTTMSFETTQTINSFGGKLLKLQHKSQVLTTEAKLNLYLPPQASSSKPVPVLFYLAGLTCTGDNGAEKGFFQHKAAQKGIAIVYPDTSPRGLGHAGEDDAYDFGSGAGFYVDATKEPYSKGYKMYSYITKELPEQLFSSFKELDGSRVSIFGHSMGGHGALTLFLKNPGQYKSVSAFAPISNPSNCDWGKKAFGGYFGDDQKQKWAEHDATELIRNYSGDTDILIDVGTGDNFYKQGQLLPENLEKAAKEAGKKVTVNYREGYDHSYFFISTFADDHVEHAAKYLLA |
| 167 | Unigene0012610 | 85 | 32462 | 11.6 | RTTYRNRTTMSFETTQTINSFGGKLLKLQHKSQVLTTEAKLNLYLPPQASSSKPVPVLFYLAGLTCTGDNGAEKGFFQHKAAQKGIAIVYPDTSPRGLGHAGEDDAYDFGSGAGFYVDATKEPYSKGYKMYSYITKELPEQLFSSFKELDGSRVSIFGHSMGGHGALTLFLKNPGQYKSVSAFAPISNPSNCDWGKKAFGGYFGDDQKQKWAEHDATELIRNYSGDTDILIDVGTGDNFYKQGQLLPENLEKAAKEAGKKVTVNYREGYDHSYFFISTFADDHVEHAAKYLLA |
| 168 | Unigene0009075 | 84 | 28495 | 4.3 | IRDVLRMPLVGQPGEAWEYGTGIDWAGIVLERATGVRLNDWIQENIMAPLDLHSVNMIPTADMKKNLAFMHQKWPGSPEKVEERDHIYREPIIAESAEERAKLFHSGGAGLYAKPAEYVQVLAALLNDGVSPKTGQRILQKSTIDEMWKNQIPQWPDFARQGIPDVKKEHTNPIPQLYPQDGNPPQGWGLSMMLTQEPGATGRGRNTGWWAGIANLFWWCDREKGVAGMIASQCMPFCDPNVLGAWVNCESAV |
| 169 | Unigene0002483 | 84 | 33115 | 7.5 | SFHQDSGAPYMLPNDAAEHARLERQSRMLSAIMNGKIPHAPLDPSKTQRLLDIGCGTGYVTKTLADKFPNAQVYGVDLTPVPQIRQYPPNVKFLQGNIVTQSPTEWKCLDGDAKLNEHENVFDLCFERLCVNSIPDAPGFIKREYQLLKPGGWIEMHELAKPFVFPDGSSVSGIDVMVEMEKIYQEKSGAVLRPGELIPGWMRDAGFINVQTSEYRWPIDMSGASPAIRAEMSDIADGVGAKDQLDVITSIITRMHMRGLLNDEEASRMIESAKVFYATDVHLKYMTFTVTIGQK |
| 170 | Unigene0013560 | 84 | 55349 | 6.5 | MFTAQTVLSTIMKFYTQVPSILTSACAAIGTCSAQSAHSGAQCSTAASNVSTAWHPPAQYFVNNLTLTLNSTGTYGFIYNTSQSPHGTVYNWCNMPHVNPTTYKKPSSDYKLEYVEVVHRHHKRTPYASNTFPAESYAWDCSDEGLFYGGKPLGPAGNDSASTYWSVYTSDSNPFTPQGFNGTCQFPQITRQGLDDSWEHGADLKAVYADLLNFIPVEYDPGATTFRVTNNVITSQVASMLIAGMYPAQSQRDTPLLIQPNSIDSLEPRYSCPASAALFSNYGVGSSATNWTEHLTVSSALFSQLDNVSGVDPNDSGWHQSLDHYFDNLSARLCHQKALPCSVNDTSCIEQADAESVFRLGQYEYSFIYRDAPQSLRASVASYGVWVAELAAHLRRAAIGDGQVRYRHNVAHDGSISRLLSILQIERMVWPGMGSEVIFELYKKEECHFLRVLWAGQALQSSHPSFRSMDMVPLNTFLAYIDELVGQGAKKVPQLC |
| 171 | Unigene0011416 | 83 | 62266 | 5.4 | MAARVNKTALHPTGVEPKHEHTEIEEELHDRAHIDYDRVAIIANPSVAALYEDALVYETGSAITSTGALSAYSGAKTGRSPSDKRIVKEEGSESDVWWGPVNKPMTPEVWRINRERAVDYLNTRNRIYVIDGYAGWDERYRINVRVVCARAYHALFMYNMLIRPSRKELEHFHPDYVIYNAGAFPANRYTTSMTSNTSVSINFQEKEMVILGTEYAGEMKKGIFTVLFYEMPVKHNVLTLHSSANEGKDGDVTVFFGLSGTGKTTLSADPKRALIGDDEHCWSDRGVFNIEGGCYAKCIGLSAEKEPDIFNAIKFGSILENVVFDPETRVVDYDDTTLTENTRCAYPIEYIENTKIPCISDHHPSNIILLTCDARGVLPPISKLSSAQTMYHFISGYTSKMAGTEQGVTEPQATFSSCFAQPFLALHPMRYAKMLADKISQHNTNAWLLNTGWTGAGATTGGKRCPLKYTRAILDSIHNGELAKAEYETYETFNLQVPKTCTGVPDELLNPAKGWTGTASLKDEVTKLAELFQENFKKYSDEATEEVQKAGPTI |
| 172 | Unigene0011119 | 83 | 78669 | 6.6 | MDEKDLVQYLTDDPPTVCPLAIKPHFDALSPQEKKYAHYISRAAFSGTRVNLRQVSDESEPIYDLIVDLHKRSNGDWKALQSEAGISDDDLRHFLSFSAQFLGNTGNYKSFGDSKFIPRISKEKFAALAKTSENASRLFQQVEGVLYDSSSVPQLHLGYPEQGHISTYYPNSPGIKTAEIAAISDFFKEKQLMPENTRLRKTSAGDFELLVASAVTEPAQRDLKETEWTLDGKKVRLVFGDHAVEMGKIARNLAEAKKFALNNDEDKMHGEYVKAFHDGSMLAHLESQRHWIRDKGPIVETNIGFIETYRDPHGIRGEWEGFVAMVNKERTAAFGRLVESAPAQIPKLPWPAAFEKDRFLSPDFTSLEVLTFAGSGIPAGINIPNYDVVRQTEGFKNVSLGNVISAAAPKEPTPFIRMEDQDLWDKYKDAAFEVQVGLHELLGHGCGKLLQETEPGVYNFDVKNPPLNPWTGKPITTWYKPGETWGTVFGGEGPSYEECRAESVAMSLCPDYSILSIFGFGDGKEDINSVAGDVLYICYLQMARAGVAALQFWEPSTQKHGQAHMKARFALLNVFLEAGKEFCELRYTKDDLSDVEIYLDRTKIESHGRPAVNRFLQKLNVYKSTADQKGGLEFYKKYTTVDEWFATKLRPEVVRQAKPRKVFVQANTFLEGDNVVLKEYDATPEGLIQSFADRDYI |
| 172 | Unigene0011118 | 83 | 78669 | 6.6 | MDEKDLVQYLTDDPPTVCPLAIKPHFDALSPQEKKYAHYISRAAFSGTRVNLRQVSDESEPIYDLIVDLHKRSNGDWKALQSEAGISDDDLRHFLSFSAQFLGNTGNYKSFGDSKFIPRISKEKFAALAKTSENASRLFQQVEGVLYDSSSVPQLHLGYPEQGHISTYYPNSPGIKTAEIAAISDFFKEKQLMPENTRLRKTSAGDFELLVASAVTEPAQRDLKETEWTLDGKKVRLVFGDHAVEMGKIARNLAEAKKFALNNDEDKMHGEYVKAFHDGSMLAHLESQRHWIRDKGPIVETNIGFIETYRDPHGIRGEWEGFVAMVNKERTAAFGRLVESAPAQIPKLPWPAAFEKDRFLSPDFTSLEVLTFAGSGIPAGINIPNYDVVRQTEGFKNVSLGNVISAAAPKEPTPFIRMEDQDLWDKYKDAAFEVQVGLHELLGHGCGKLLQETEPGVYNFDVKNPPLNPWTGKPITTWYKPGETWGTVFGGEGPSYEECRAESVAMSLCPDYSILSIFGFGDGKEDINSVAGDVLYICYLQMARAGVAALQFWEPSTQKHGQAHMKARFALLNVFLEAGKEFCELRYTKDDLSDVEIYLDRTKIESHGRPAVNRFLQKLNVYKSTADQKGGLEFYKKYTTVDEWFATKLRPEVVRQAKPRKVFVQANTFLEGDNVVLKEYDATPEGLIQSFADRDYI |
| 173 | Unigene0014365 | 83 | 52577 | 4.3 | KCSDLASIDLKPYRESFLNATYYAEGSRNATGGVNGALNQVGFCEVNAVISYGNNQSLNFATWLPDTYEDRFIAVGNGGMAGTIDVSGMLTQLNNGLGMAVAAGDAGHLAADNQNITGYLPYLHDKEQVQAWIHDAISLFTPAAQALVEKYYAKQPNKSYYYGCSTGGAQGFALAQFHPELFDGIYAGCPGNWYSHLALSFLWNAQHADTPETALTQEELDFVTGAVLDACDEIDGVADRLIENPLACKFDVNSLACQSSSNSTCLTEAKIETVKAIYAGPKRADTGAEVYPGFDFGSEIGWAVQQRNLSRDFSVPILKNLVFDDQSYDNATFNWASDVDIVGARAGRLIDSITPDLSAFKSRGGRLLTSQGWADQYNAGTWPIEHLQQVESAMGGSVDDFYRLVMIPGGGHCGANPGYPNVPATYSFIAPLVKWVESGGAEPPTQLLSTSPPSGGNETRKLCVWPAVAKYGGGDVDDWAAYEC |
| 173 | Unigene0014366 | 83 | 30526 | 7.5 | SLTRSQTPETALTQEELDFVTGAVLDACDEIDGVADRLIENPLACKFDVNSLACQSSSNSTCLTEAKIETVKAIYAGPKRADTGAEVYPGFDFGSEIGWAVQQRNLSRDFSVPILKNLVFDDQSYDNATFNWASDVDIVGARAGRLIDSITPDLSAFKSRGGRLLTSQGWADQYNAGTWPIEHLQQVESAMGGSVDDFYRLVMIPGGGHCGANPGYPNVPATYSFIAPLVKWVESGGAEPPTQLLSTSPPSGGNETRKLCVWPAVAKYGGGDVDDWAAYEC |
| 174 | Unigene0012380 | 83 | 52168 | 8.2 | MALSSRTATRALRASKLSQTVPLAYNASRSYATAEPDLKETLRNAIPEKRELLKKVKANASKTIGEVKIENTIGGMRGLKAMVWEGSVLDANEGIRFHGKTIKDCQQELPKGTSGTEMLPEAMFWLLLTGQVPSTNQVRAFSKELAEKAQLPAFVNKMLDDFPKDLHPMTQFACAVSAMQYESVFAKAYERGINKADYWEPTFDDCINLLAKLPTVAAKIHQNAYCGGGALPAEVDLNQDWSYNFAAMLGKGGSENENFQDLLRLYLALHGDHEGGNVSAHATHLVGSALSDPYLAYSAGLQGLAGPLHGLAAQEVLRWILQMKDHIGTKFTDQDVKDYLWTTLKSGRVVPGYGHAVLRKPDPRFEALMDFASSRKEIAEDPVFQLVKKNSEIAPGILTEHGKTKNPFPNVDSSSGVLFHHYGFHNTLYYTATFGVSRGLGPLAQLIWDRALGLPIERPKSIDLKGLLKLAEA |
| 175 | Unigene0014285 | 83 | 41084 | 4.6 | DVAQYQRQLEQAIQEKNLQRFYGPGTLGAQKLPQIAARAAQCVDRLCQAWRVQKEIANDIVRLALYDVVVYVDDSGSMSFEENGERIKDLQLILQRACFAATLFDDDGIDLRFMNEDLPMQNVSHIRSEQQVEQILSQKRYKGLTPFGTELRKKVIDPILIQRLNSGQMQKPLLIISITDGQPAGENQNALTETVQYAVQAAQRSQYGPGAVAFQFAQVGNDQKATEFLAKLDNDPMVGREVDCTSNYENESAEMARAVPPVDLTPDLWMIKLILGAIDPSYDTKDEKSHMAPGAGVPPPGQYGQQQYGAPPPQQYGAPPPNQYGAPPPNQYGAPPPGQQQYGQQQQRPPQGYPGQQQYGAPPPGPPRY |
| 176 | Unigene0009205 | 82 | 14657 | 10.6 | MRTSILASAATLLVATQARIVGFYAPNTAAPGSKVRIEIQTENFSQSIEDVAVSFGLSTGDGFTLGSLGQLISTKILGADLSNTLENITHIIQIPADATPGPAVIQGAHFSLTGAVYSPLVEVYYANITIGQGNNFPLVQS |
| 176 | Unigene0009204 | 82 | 8458 | 18.1 | MRTSILASAATLLVATQARIVGFYAPNTAAPGSKVRIEIQTENFSQSIEDVAVSFGLSTGDGFTLGSLGQLISTKILGAGTSS |
| 177 | Unigene0001577 | 82 | 17669 | 15.3 | LALIAASMAASSMAQYIPRSCNEIFGDSGYICSRDGSEVQYCDFNSSPVQVTSEYCGFNDCWQSQGQGVKNDPNELSCYYGRQYNQANRARGPTSCDQVGGFGYICSSDYRTLQFCQGGGGGRFNRFGPQTQAQDWIYCGGATCFASINGGQGECIQ |
| 178 | Unigene0001100 | 82 | 88729 | 2.6 | LLSGLGGASTLTPPVLPLIVRNPYLSTWLADAREEPWSKWPMFWTGQSMGFSVLASVPDTSTVYPLLGRPHDSLDRESNHYNLATPHYLGAKYDASTTNLTYVISNSSSSQNVEIVLSFLSPITPTSTLRQAIPASYVTVFVKGNVHVNVYIDVNGQFVSGDRGSHIQWALEEKSRKGSAKLKTFRVKRSNEELFTEKWDRAEWGTLHFTGPSHTAHEAGTSGILRQRFARTGTLQNSVDDAFRSIMDEEPVFAFVKSFNLSSASQQSGTQQDSVVFTLAHTQDPVTQFASSRGLTWMRPLWMSYFSSDAELIDFHYTDFDHARALALDYSQQVARDAYASGSDSYKDIVELSARQVMGATSFSGTPENPILFLKEISSNGNCQTVDVIFPAFPFFLYTNPRWLAYLLEPLLEHMLSGQYPNDYTMHDLGTHFPNMTGHPDGRDEYMPVEECGDMLIMGLALVNSLTYDSAETAQSPWSALGEPKSQEQLQMQSDDATPFSLPSKFETREGILGLDDQFGGVTSSHQAKKWLSRTYRLWKQWTGYLVDYSLYPHNQLCTDDFAGWLPLMTNLALKGIIGIKAMSELAELSGETSDAKYYRNISEAYIKEWEKQGMSRDGGRAKLAYDWHGSWTTLYSLYADALLCFHPSTEAAESSVASSRRGGVQAPLHPGAKGKANFIPDRIYQTQSDWYGVVMQKYGLPLDSRHLYTKSDWEFQAAAVASKKTRSEILDKVATWLNETASDRPFTDLYKTEGDGGFPGPNFFARPVVGSHFAFLTLERACQGKGAAAFDY |
| 179 | Unigene0012603 | 81 | 61226 | 2.6 | MRASTLLTGLLGSSVLATEPVVKVLNGSYSGIYLPQYQQDIFLGIPYAQDTGGQNRFRIPQTLNETWQDVRPATSYSHACPSYDPNDAIYGMSENCLSINIVRPHDIPEDQKLPVMLWIHGGSYQVGTTGRPDYNLTYIVEKSIRIDKPIIGASINYRKGGWGNQYSIEIQGSGNANLALRDMRKGLSWISENIAAFGGNKDSVTIWGESSGSFAVGQLLLSYGGKTDGLFHRSIQESGSATTAWYNGTDWYQPIYNDLVDNTNCTDAPDTLACLRTLEYDDIFPFLNNSNVAGPGWYPTVDGDVIPNFSTILLEEERFAQIPHLYGSTSDEGTDNVIPGIDTDEELRYHLLYNTGFQFPNSTVSRILELYPDDPTQGVPINTGTERFADYGQQYKRAAAIIGDVFYHAPRLSDARYYAQHSPTYIYRFNTRPWLKSTGTLALPHKGVQHFSEVAFVFNNPDFVGPYPEYQALSEQISAQWINFAWCGNPNGEGLPHWPLYNESQAGQNLVLQTESQGGNYVEEDTYRLEGREYLTKWARRRHV |
| 179 | Unigene0012604 | 81 | 57038 | 2.8 | MRASTLLTGLLGSSVLATEPVVKVLNGSYSGIYLPQYQQDIFLGIPYAQDTGGQNRFRIPQTLNETWQDVRPATSYSHACPSYDPNDAIYGMSENCLSINIVRPHDIPEDQKLPVMLWIHGGSYQVGTTGRPDYNLTYIVEKSIRIDKPIIGASINYRKGGWGNQYSIEIQGSGNANLALRDMRKGLSWISENIAAFGGNKDSVTIWGESSGSFAPIYNDLVDNTNCTDAPDTLACLRTLEYDDIFPFLNNSNVAGPGWYPTVDGDVIPNFSTILLEEERFAQIPHLYGSTSDEGTDNVIPGIDTDEELRYHLLYNTGFQFPNSTVSRILELYPDDPTQGVPINTGTERFADYGQQYKRAAAIIGDVFYHAPRLSDARYYAQHSPTYIYRFNTRPWLKSTGTLALPHKGVQHFSEVAFVFNNPDFVGPYPEYQALSEQISAQWINFAWCGNPNGEGLPHWPLYNESQAGQNLVLQTESQGGNYVEEDTYRLEGREYLTKWARRRHV |
| 180 | Unigene0002867 | 80 | 59906 | 6.5 | MFRTALRSSARAAGAISASSRFAAQRTTPAVATAFARGYAAESKAQPTEVSSILEQRIRGVQEESGLAETGRVLTVGDGIARVYGLNNVQAEELVEFASGVKGMAMNLEAGQVGVVLFGTDRLVKEGETVKRTGEIVDVPVGVELLGRVVDALGNPIDGKGPIKTKERRRAQMKAPGILPRKSVNQPVQTGLKSVDAMVPIGRGQRELIIGDRQTGKTAVALDAMLNQKRWNQGNDETKKLYCIYVAVGQKRSTVAQLVQTLEEQDAMKYCIIVAATASEAAPLQFIAPFSACSMGEYFRDSGKHAVIIYDDLSKQAVAYRQMSLLLRRPPGREAYPGDVFYLHSRLLERAAKMNKSFGEGSLTALPIIETQGGDVSAYIPTNVISITDGQIFLESELFYKGIRPAINVGLSVSRVGSAAQLKAMKQVAGSLKLFLAQYREVAAFAQFGSDLDAATKQTLQRGERLTELLKQKQYTPMAVNEMVPLIFAGVNGYLDNVPVNKILKWESDFLNHLKTNEAGLLETIDKEGALSKDLEAKLKDVVTSFLKSFS |
| 181 | Unigene0013639 | 80 | 50306 | 12.6 | MGKEKTHINVVVIGHVDSGKSTTTGHLIYKCGGIDKRTIEKFEKEAAELGKGSFKYAWVLDKLKAERERGITIDIALWKFETPKYYVTVIDAPGHRDFIKNMITGTSQADCAILIIAAGTGEFEAGISKDGQTREHALLAYTLGVKQLIVAINKMDTTKWSEDRYNEIIKETSSFIKKVGYNPKTVPFVPISGFNGDNMIDNSTNCPWYKGWEKETKTKSTGKTLLEAIDAIDTPQRPTEKPLRLPLQDVYKIGGIGTVPVGRVETGVIKAGMVVTFAPAGVTTEVKSVEMHHEQLTEGLPGDNVGFNVKNVSVKEIRRGNVAGDSKNDPPKGCDSFNAQVIVLNHPGQVGAGYAPVLDCHTAHIACKFSELLEKIDRRSGKSIENSPKFIKSGDAAIVKMVPSKPMCVEAFTDYPPLGRFAVRDMRQTVAVGVIKSVTKSDKGGAGKVTKAAQKAAKK |
| 182 | Unigene0000504 | 80 | 69804 | 6.8 | SIAGALPSQKRSTTYVLKERHLVPRSWRKLGPASKSDTLNLKIGLVQQNPGAIEQHLMQISDPTHERYGQHLSQEEIDEIVAPPKESMDLVKSWLEEHGITNYVPNKSKTMIHCAIPIGKAETLLNTTYSTFKHEDGTEINRAPEWSLPEFLLDSIEIVQPTNSFFHPKQHLVSDNNAWHDAAWWKSEGEQTYPQAFAGSADGQYGQAYESNINGQPPPVQGAQVPQFGQSQPQQNGQPSQGGFQPPQGGNVGGTIDVSQVCQDKFVTPQCRRTLYGTIDYIPQAPNQQTVATTNYLNETVIRSDISLFMQTFRPDASNIENEINIVSIANGDVDQNISPQKIAASTDQEANLDAGNIESIAYPIPLTAYHTGGRPPFQPSAATPNNSNEPYLEWLDYMLALDQVPQVISSSYGDEEMTVPRSYAERVCSGFAQLSARGVSLIVSTGDDGVGKDGQCIANDGSGDKFVAVFPASCPWVTGVGATAGFNPEVAAYRFASGGGFSYYFDAPDYQKSTTQAYIQALGSQYGGAYNPNGRGYPDAAAHGDHDAVVFNGQVSTMGGTSASAPTFAGVIALVNDALIAAGKPPLGFLNPWLYSVGYQGLTDIVSGSSSGCNTTGFPAQQGWDAVTGFGTPNFRQLVQLALN |
| 183 | Unigene0002378 | 79 | 58981 | 6.7 | MASTVIHRDEEQNAGTMELKENTVIIVLGASGDLAKKKTFPALFGLFRNGFLPRDVKIVGYARTKMDHTEYLKRVKSHIKTPTKEMEQQLDEFSKFTTYVSGQYDKDESFQELEKHLQELEKGQKETNRVFYMALPPSVFIPVSEHLKRNNYPKSGIARIIIEKPFGKDLASSRELDQALRPNWKEEEIFRIDHYLGKEMVKNILILRFGNEFFGATWNRNHIDNVQITFKEPFGTEGRGGYFDEFGIIRDVMQNHLLQILTLLAMERPISFSAEDIRNEKVRVLRGMPAIEPKNVIIGQYEKSLDGSKPGYKEDDTVPKESRCPTFASMVAYIKNERWDGVPFILKAGKALNEQKTEVRIQFKDVTSGIFKDIPRNELVIRVQPNESIYIKMNSKLPGLSMQTVVTELDLTYRRRFSDLKIPEAYESLILDSLKGDHSNFVRDDELDASWRIFTPLLHYLDDNKEIIPMGYPYGSRGPAVLDDFTSSYGYKFADAAGYQWPQHQIEPNKL |
| 184 | Unigene0011200 | 79 | 56436 | 7.7 | MSIAGIVALLVLTLCQVAFAANGDAWRGRSIYQVFTDRFARTDGSTTASCDTGAADYCGGTWAGLINRLDYIQDLGFDAVWISPVTHQVEGQSIDGSAYHGYWQDDINEVNEHFGTADDLRALSAELHSRNMFLMVDIVVNHLAWIGAPDTIDYSQFPQFNNEDYFHPYCVNNYSQDNMTNTEQCWMGSTNVPLPDLRTEDSDVQNIWNAWISRLISDYSIDGLRIDSVMEVNTGFWPSFLDAAGVYALGEVYMNDPDFVARYQGYMDGVFNYAYYFPLRGAFVQGGDISSLAEMIDKVKAGAYRDTSLLGSFSENHDQPRIGALNPDMALAKNVLVGTMLVDGIPVIYQGQELHYQAYGGQSTPFNREAIWLSGFPTDGELYQATKACNAARKNAQADDSSYLTYQNYHFYTDGDTIAMRKGKMVTILSQLSSTGSNYTLNLDSGYDAGTQVTELLTCSDLTVSSNGTLAVPMSAGQPRVYYPTDSIGSQCGGSSKRMVRRSAKFR |
| 185 | Unigene0010033 | 79 | 74937 | 4.4 | MPYNKTDELAINTIRTLAVDATFAANSGHPGAPMGMAPVAHVLFNKFMTFNPKNPKWLNRDRFVLSNGHGCMLQYALLHLFGYDVTIDDLKAFRQVGSKTPGHPESTDTPGIEVTTGPLGQGFTNAVGLAIAQAHTAAVFNKPGYELVNNHTYTFFGDGCAMEGIASEAASTAGHLQLGNLIAIYDDNHISIDGDTKCAFTEDVTKRFESYGWHVQWVKDGDNDLEGIEAAIKKAKEVKDKPSMIRLTTTIGFGSVLQGTGGVHGNPLKEDDCKQVKEKFGFDPSKSFAVPQEVYDAYHKHAAEGAAAEQEWNQLLEKYSNEHKELAADLKRRLTGKLPDGWQNKLPVYKPSDKAVASRKLSESVLEAIYEAVPELLSGSADLTGSNNTRWKSAVDFQPPSLGIGDYSGRYLRYGVREHAMAGIMNGLSAYGTIIPAGGTFLNFVSYAAGSVRLSALSHHRVIYIATHDSIGLGEDGPTHQPIETLAHFRALPNMMVWRPADGNETSAAYYVALTSEKTPSILALTRQNLPQLEGSTIENGIKGGYVAVEAEGAQITLVSTGSEVSLCLEAAKYLKDNKGVTARVVSMPCMEVFDAQPKDYKLKVIPDGIPALSVEVMSTLGWEKYSHEQFGLNRFGASGPYKEVYAKFEFTPEGIAKRAVATIDFYKEVKPLRSPLNRAFQQLI |
| 186 | Unigene0003211 | 78 | 17203 | 17.1 | LVARQSSSSDELENGPCRQVTFIFARGSTEAGNMGTVVGPQTCAALKTLLGSENVACQGVGGPYLGTLIVNTFPRGTSDAAIQEAVRLFTLANTKCPDTIVTSGGYSQGTAVIAAALSDLTTQAPAVVDQVVGAVLFGYTHNKQNGGRIPNYPTERTEVYCAPG |
| 187 | Unigene0010434 | 78 | 22867 | 6.2 | MTEQLPEIPNIERLSPRVIRILGGNPSKFTLQGTNTHLIGTGKRRILIDTAEGLPIWKENLAKVLHDEGGVELEAVILTHWHPDHVGGVADVKELLGEKGTGVKIWKKDPDAGQEGFENGQVFEIEGAKLRALHSPGHTTDHMCFVLEEEKALFTGDNVLGHGTAVFEDLTAYMNSLNVMLEAEGFEGRAYPSHGVVIEDGKGKIKEY |
| 188 | Unigene0003277 | 78 | 56825 | 6.6 | AQQALNDPKSACSNLASTFTYPNVTINFSEHVAAGTNLTFDQSTPELRSCSRASQVVPVDLCRVAFYVATSNRSGITAEAWLPSNWTGRYMSTGNGGLNGCISYEDIAYGTEFGFAAVGNNNGHNGTGGTSFYQNADVVQDFAWRSIYTETKVGKELTKAFYGEDLSKSYYLGCSTGGRQGFKMAQDHPDLFDGILAGAPALAFNNLSSWSGHFLPIIGSNTSETYLSPAKWNLVHQDILKQCDSLDGAEDGVIEDPNLCHYKPVALQCASNATNTTNCLTAPQVKTVTGVFAPYYGENGDLVYPRMQPGSESVASRLYYNGQPFPYTTDWYRYAILQDPNWDPSTLNASVVAYAASLNPFNIETWSGDLSAFQKRGGKLLTYHGLMDAIISSENSPRYYEHVATTMGLSPSELDSFYRFFRISGLGHCSGGDGAWHIGQKTEGESGAERNVLQRIIDWVEKGDAPVTVTGVRYVNDTKSEGIDYERKHCKYPLRNKYVGPGNWKEADAWTCV |
| 189 | Unigene0005833 | 78 | 65277 | 6.8 | NVTYPYQNGSLCIDERLDDLISRMTVAEKAGQLFHTQMMMGPNGTLDQGDERRNSTTTMISNMFLTHFNLASDVVNVTQTAEWHNTVQELALNTRLGIPITFSTDPRHAFTENLGTGFAANAFSQWPESLGLAALRSAETVQSFAEVAREEYKAIGLRSALHPQIDLSTEYRWARIANTMGEDAELTSELVVAYLKGFQGEEFGTHSVTTVTKHFPGGGPMENGEDSHFTYGKNQTYPGNNFEHHLIPFKAAIAAGARQMMPYYSRPIGTEYEEVGFSFNKGIITDLLRNELGFDGIVCSDWGLITDTVILGQDMPARAWGVEYLDELERAARILDAGVDQYGGEQRPELIVELVENGRISEERIDLSVRRLLREKFALGLFDNPYVDVEKAAQIVGNDEYVKLGAQAQRDAYTLLTNKDDILPLKDFSGKVYAEGFNSTYLTRRNIEVVDTPEEADIAFLRLQAPYEPRPGGFEARYTAGSLEYNATEKARQAEIYAAVPTIVDVYLGRPAAIPEVAEQAAAFLGSFGSGPDAFLDVIFGVDGAEPKGKLPFDLPRSNQAVEDSYEDVPFDTTDPPFRFGDGLKYKK |
| 189 | Unigene0005834 | 78 | 65277 | 6.8 | NVTYPYQNGSLCIDERLDDLISRMTVAEKAGQLFHTQMMMGPNGTLDQGDERRNSTTTMISNMFLTHFNLASDVVNVTQTAEWHNTVQELALNTRLGIPITFSTDPRHAFTENLGTGFAANAFSQWPESLGLAALRSAETVQSFAEVAREEYKAIGLRSALHPQIDLSTEYRWARIANTMGEDAELTSELVVAYLKGFQGEEFGTHSVTTVTKHFPGGGPMENGEDSHFTYGKNQTYPGNNFEHHLIPFKAAIAAGARQMMPYYSRPIGTEYEEVGFSFNKGIITDLLRNELGFDGIVCSDWGLITDTVILGQDMPARAWGVEYLDELERAARILDAGVDQYGGEQRPELIVELVENGRISEERIDLSVRRLLREKFALGLFDNPYVDVEKAAQIVGNDEYVKLGAQAQRDAYTLLTNKDDILPLKDFSGKVYAEGFNSTYLTRRNIEVVDTPEEADIAFLRLQAPYEPRPGGFEARYTAGSLEYNATEKARQAEIYAAVPTIVDVYLGRPAAIPEVAEQAAAFLGSFGSGPDAFLDVIFGVDGAEPKGKLPFDLPRSNQAVEDSYEDVPFDTTDPPFRFGDGLKYKK |
| 190 | Unigene0012299 | 77 | 71235 | 3.4 | MKQSAAWLAAWAALSPASATPVDLEALALAELVRRAAPANAPNGYTPTFAQCPANRPSVRSAGQLSPNETQWLEVRRNATMEPMKALLSRLNITGLDINQYIDNNRNNASALPNIGIAASGGGYRAMLNGAGVIQAFDARTPGSTTGKQLGGLLQSATYFSGLSGGSWLVSSIYSNNYTSVSDILAQDTADDDDSSSGRVWQLDNSIFTGPESGILSSVDYYAGLLGQVGGKRDAGFNTTLTDYWGRALSYQIVNATDGGPDFTWSSIATQDWFTRGDAPLPLVVTDSRNPGETVISTNSTVFTITPWELGSDDPTLYAYAPLQYVGTNFTNGEAVEGQQCVTGFDNVGFVFGTSSSLFNAILNTVDNANSTGALSSALQDALTGVLEALGQDEEDIADWVNPFQGYRNGTFPQATDQTLTLVDGGLDGQNIPLNPLIQPNRNVDVIFAIDSSADTNGTEGGGQWPTPQSGANWPDGTSILTTYERSLSDIGNGTNFPAVPSRNTLLNLGLATKPTFFGCDTSNFTSGNIPALLVYMPNGPYVYYSNTSTFGKLSYNDTERNGMVLNAYNMATQGNGTVPGFEDWPTCVGCAILSRSLERTGTTVPDVCEQCFTKYCWNGTTDDRTPSTYDPALKLPDSEFNVASGSARLVSEISMVLIAAVAVGMAL |
| 191 | Unigene0001868 | 77 | 15204 | 18.3 | TDKMQIFVKTLTGKTITLEVESSDTIDNVKSKIQDKEGIPPDQQRLIFAGKQLEDGRTLSDYNIQKESTLHLVLRLRGGIIEPSLKALASKYNCDKMICRKCYARLPPRATNCRKKKCGHTNQLRPKKKLK |
| 191 | Unigene0001888 | 77 | 18033 | 15.5 | MQIFVKTLTGKTITLEVESSDTIDNVKSKIQDKEGIPPDQQRLIFAGKQLEDGRTLSDYNIQKESTLHLVLRLRGGMAKKRKKKVYTTPKKIKHKRKKTKLAVLKYYKVDGDGKIERLRRECPAETCGAGIFMAAMHNRQYCGRCHLTYVFDDPK |
| 192 | Unigene0013451 | 77 | 52118 | 5.8 | GSWGPSIQFPLVTVSGAIAPDTGNLIVWSSRDRDWSGDGNVGQTFSAEYNWRSGAVSERLVSQTNHDMFCSGVSMGHQGDILVTGGSSATRSSFHDWRTPDVWKPGPELKIPRGYQTQTTLSDGRMFLIGGSWSSMTAGWWNGVNGGKHSEIWDPSTNAWELLPGIPVDPILTDDWAGLYRSDNHAWLHTWKDGSVFHAGPSKRMNWFYTNGEGSHAPAGERDGVDAMCGFSVMYDAVAGKILSGGGAPSYEESAAITNSHITTINEASGIAQVKQLKGLNRGRIFGDGILLPTGHVLTTGGMSWGKVYTDATAVTVPEIWDPVTEEWTELADEVTPRTYHSIGVLLQDGTVFSGGGGLCGVVCVNGINHFDGKIFTPPYLSGGEGTRPTIESTSTQDVPVGGILIATLGANPDDATFSLVRLSTVTHVVNTDQRRVPLTPVAREGNSYTLQLPGDAGVLLPGYWYLFAISSAGVPSVAQIIKVHI |
| 193 | Unigene0012396 | 76 | 36926 | 10.4 | MAEEAAYVQKLKSIPKATFLFGPSPIQHLPRLTESLGSKVQIYAKRDDCNSGLAYGGNKVRKLEYLVADALSKNSTHLVSVGGVQSNHTRAVTAVATASGLKAVTVQEKWVPIDPPLYDKTGNILLSRLMGGDVRLNNEGFHIGHKTATQEAFAEIEKDGGKPYYIPAGASDHELGGLGFTNFVVELAEQEKTLGLPFFDTLVVCSVTGSSHAGLIVGAVAEGRGRKVIGIDASGKPAETKAQVARIARNTAKLLDEKLEIPDEAVVLDERFHEGIYGIPGPSTIEAMKVAARTDALITDPVYEGKSMAGMIQLIKEGTIKEGSNVIYVHLGGQPALNAYSSYFD |
| 194 | Unigene0014906 | 75 | 87034 | 7.1 | MVQSSVLGFPRMGRLRDLKKANEAYWADKLSRDELLAEGKRLRLEHWKIQKDAGVDVIPSNDFAYYDHVLDHIQMFGAVPERYSSAKLHKLDEYFAMGRGHQKDNVDVPSLEMVKWFDSNYHYVKPTLQDNQTFTLAENPKPVAEFLEAKEAGIQTRPVLLGPVSFLALGKADRGSSVDPITLLDKLLPVYEQLLTQLKEAGAETVQIDEPVLVFDLPAKTKQAFKPAYEKLTALQGGKGPQLVLATYFGDVVHNFDVIDSALTGTHAFHVDLVRHPEQLAQVIEHLGPKQVLSAGVVDGRNIWKTNFKKAIEVVETAIQKLGKERVIVATSSSLLHTPHTLASEKNLDEEVRAWFSFASEKAVEVAIIAKAVTNGPDSVRAELEANAKDQQSRATSKRTNNPEVKKRIEAVKKEDYERKSGFDTRYAAQKKHLDLPIFPTTTIGSFPQTKDIRIQRNKFTKGEITAEEYEKFIEKEIQECVQIQDELGLDVFVHGEPERNDMVQYFGERLDGYVFTTHAWVQSYGSRCVRPPILVGDVSRPAPMTVKESKYAASISKKPMKGMLTGPITCLRWSFPRDDVHQSVQAQQLALALRDEVVDLEAAGVYVIQVDEPALREGLPLRSGVEREKYLDWAVNSFKLSTAGVEDSTQIHSHFCYSEFQDFFHAIAALDADVLSIENSKSDAKLLKVFIDEAYPRHIGPGVYDIHSPRVPSEQEIKDRIAEMLQYLKPEQLWINPDCGLKTRQWKETKAALVNMVNAAKHFRAQQSK |
| 195 | Unigene0001201 | 75 | 27947 | 13 | MSGSAGFDRHITIFSDQGRLYQVEYAFKAITAANITSVGVRGKDCAVVISQKKVPDKLIDPSSVSYVYKLSPSVGCVMTGSIADARASVNRARGEAAEFRYKNGYEMPCDVLAKRIANISQVYTQRAYMRPLGVATTLISVDEEFGPQLYKCDPAGYFVGYKATASGPKAQEALNFLEKKLKNKEHADGSWDEVVELAITTLSTVLSVDFKKGELEIGIVGGPRKDGKEGTDKAFRPLTEDEIDERLQAIAEKD |
| 196 | Unigene0009691 | 75 | 75896 | 3.8 | ICAQSSFTPARPPAIPLAVRTPYLSTWQAAGSNGGNGGYLAGQWPTFWAGQINGWAGLIRVDGETYTWMGKPDPLPTIVTQTAFEYTSTRSTFTMDVDGKVSMNVTFLSPVSPTNKERQGLPVSYMSVNVQSSDGAEHDVSIYTDISAEWTSGDRGKVAEWSRGTARGMGAAAGNANIAYHKVWRQVQQEFSEDSDQAAWGNWYYTTEDAEQLTYQSGADVDVRKKFTDDGTLANTDDTNFRPINQAFPVFAFAKDLGSVGNQNVETLFTINLLQQNSVQFATGVNQIQSMPAYWRAVYGDDELSAVATMYYDYQDASASSSGLDAQVDRDARAVGGADYATITSLAVRQAFASVQLAGTQDENYLFLKEISSNGNFQTVDVVFPFHPILMYLNPDWMKLILDPLFINMEAPGLWPQDYAIHDLGDHYPNATGHPDGIAALQPLEECGNMLIMTLAYAQRTNNIDYLNTHWDALHKWGQWLITNNSVIPFNQISTDDFAGPLANQTNLALKGIIGLKAASIIANLTGRSTEAQEYDEQSRDWIQQWQDLGNLPNANPPRTSLSYGDEESWGLLYNLYGDALLEANLVPKEIYEQQSDFYPTVQARFGVPLDTRARRSKNDWEMFVAAIASDETRDMFLSDLVKFINETPTTGPVTDLFDVDSGDYPSGTMFRARPVVGGWFALLAL |
| 197 | Unigene0000558 | 73 | 14065 | 20.8 | MSANKKVILTENAPPPLPGVLSQAIVANGTVYCSGSVAVDPATGKLIEGTVADRTHQCIKNLTAVLEAAGTNIENVVKVNVFLDDMANFSAMNEVYKQYWGENKPCRTCVAAKQLPLGTDVEIECIAVLP |
| 198 | Unigene0010659 | 73 | 16890 | 10.6 | MSPSSTLLRQTLLTTRLSSRTSTTTAFRATYQPIQQTFRNFSSTMPAEAVHNIKAKADFDSLKADKDKLSVIDCYATWCGPCKVIAPQVSKFADQYNNATFYKLDVDELPDVAQELGVRAMPTFYLFKNGEKVQEVVGANPNALEAAIKAH |
| 199 | Unigene0013980 | 73 | 28803 | 9 | MPQRIPEAESLKELLSLKGKVVVVTGASGPKGMGIEAARGAAEMGADLAITYASRPDGGEKNAKELSEKYGVKVKAYKCQVDKYESVEKLVKDVIADFGKIDAFVANAGATANSGILDGSVEDWNHVVQVDLTGTFHCAKAVGHHFKERGTGSFVITSSMSGHIANFPQEQTSYNVAKAGCIHMARSLANEWRDFARVNSISPGYIDTGLSDFVPKETQKLWHSMIPMGRDGLAKELKAAYVYFISDASTYTTGADLIIDGGYTCR |
| 200 | Unigene0012828 | 73 | 89737 | 3.8 | MASRHAKLAALASAFFAAHGQAQVDILSFVDPLIGTTNGGHVFPGATLPFGMAKAGADVNVENQGGFSTGDVTGSITGFSHMHDSGTGGSPSLGNFPLFPQAGCPDDDINQCVFPYDLRSVPRINDSVEAHPGYFAVSMQTNIHTEMTATNHTALYRFNFPEQPVERNATLSPLILVDLIDLPRSRINGSISVDSSSGRISGSGVFSPSFGIGNYELHFCADFSGADIRETGVFMNNRAGTEPKNLSVVIDGINNSPDVLPAGAWTRFEAPKENRQIIARVGVSFISISQACHNAEMEIPNFDFNSTYAAAQEAWRDTLGVVEVKPGGVNGSLQTVLWSGLYRASISPQDYTNENPLWNSPEPYYDSYYCIWDSFRSIHTLITILDPYSQIQMIRSLIEIYRQEGWLPDCRMSLCKGFTQGGSNADVVIAESYLKLGQLAADHGVDWKTAYEAVVKDAEEEPQNWAVEGRGGLLSWKTLKYIPTDDYDPYGVGPFTRSISRTVEYAYDDYCIAEMARGMGNTADYEKYLASSEYWRNMWKADQNSSINGTDTGFEGFLMPKFLNGTWGSQDPIFCSLILNFTSCYLNPDGHETYEGSSWLYSFFVPHDQASLIATMGGAQTFVDRLDYLHEFPGLLYIGDEQGFLPVYQYHYAGRPGKSAERAHFYIPSQFNDTINGIPGNDDSGAMGSFVALSMMGMFPNPGQDVYFITPPFFEEVSVTNKLTGKKATIRNINFDPEYQNIYIQRATLNGEPYTKNWYSHSFFLDGGVLELTLGRNESTWGTRPEDLPPSVSTSAGYERNEFNYFM |
| 201 | Unigene0014402 | 73 | 35445 | 11.1 | MAEQLVLRGTLEGHSGWVTSLATSLENPNMLLSGSRDKTLIVWNLTRDDTSYGYPKRSLHGHSHIVSDCVISSDGAYALSSSWDKTLRLWELSTGQTTRRFVGHNNDVLSVSFSADNRQIVSGSRDRTIKLWNTLGDCKYTITDKGHTEWVSCVRFSPNPQNPVIVSAGWDKFVKVWELSTCRLQTDHIGHTGYINTVTISPDGSLCASGGKDGTTMLWDLNESKHLYSLSAGDEIHALVFSPNRYWLCAATAKEIVIFDLEKKSKVDELRPEFVESGGKARDPECISLAWSADGQTLFAGYTDNKIRAWGVMSRA |
| 202 | Unigene0010565 | 73 | 25214 | 7.9 | MAGRPEPLRLGSIAPNFQAETTNGPIDFHEFIGDNWVVLFSHPEDYTPVCTTELGAFAKLEPEFTKRGVKLIGLSANTIESHGGWIKDINEISGSNLRFPIIGDKQRQVALAYDMIDHQDATNVDEKGIAFTIRSVFIIDPKKTIRLILSYPASTGRNTAEVLRVVDSLQTGDKHKVTTPINWVPGDDVIVHPSIKTPEAEKLFPKINIVKPYLRFTPLPKEETSAA |
| 203 | Unigene0010582 | 73 | 24041 | 14.6 | MSDGEVEVEQSPNYNVLPKDVVENIGQTKLFNKWSYEDVEIRDISLTDYIQIRQPVYISHSAGRYAVKRFRKAQCPIIERLTNSLMMNGRNNGKKLMAVRIVAHAFEIIHIMTDQNPIQIAVDAIVNCGPREDSTRIGSAGTVRRQAVDVSPLRRVNQAIALLTIGAREASFRNIKSIAECLAEELINAAKGSSNSYAIKKKDELERVAKSNR |
| 204 | Unigene0007076 | 73 | 43150 | 15.7 | MKSFVAISLISGASAFPWVANVPGVDSSALSAVQKRQQNPQPGGPGSPETCPFNAQHVNAVPVREGWYNNARGGRKGNERGGYQVPRPDDPDHQFIAPRAGRDIRGPCPGLNTAANHGFLARDGITTFNELVDAQQNVYNVGHDLSLLLAFLGLQADGDLVTTKLSIGCDATTRTSIAPILTGSQPGLAGHNKFEADTSLTRNDFFTGGGDNFSFNRTLYDMMAATTGGLHNRENLAVYRGERFDQSLVENPNFFFGPLSLLLFGAASFLYELFPSGPNYIPDQATMESFFVREQLPANWINRIEPYTNRLVTQEILAMYLLNPRPFGGNTADGSYNAINWRAIQNGELPIGIGAPETLCLLYQLGTQSIPSTLNGIVTPTVEAISFFLTRVQPAF |
| 205 | Unigene0006408 | 72 | 65703 | 5.3 | VSASLYSAAGDALDISARSARGYAPDLAISGGVLTSKWVEGTKYTQIVEFVVTNNHPANSLTLADTLNITLKSNNLELVQSATLTRLAPKQAAVVQLGVRNLRNTPSGSTCSGTIVATYGQGYGPVVTRDQTITGICGIPDYTADTGSLGHHWNPDWYNNVKFGIFIHWGLYSAPAFGNATPVQDYSEWYWCRQHDPNYRTKTYQYHEKTYGRNFNYDQFMANFTDTGYDPVAWINLFAEAGARYIVPVTKHHDGFALFNTSPNVSRRSSMYYGPKKDLIGPLLAAAKQYQPQIRRGTYFSMPEWYNPQYAQYKNPDPNWAGGCFGAENINPYTKAPLDYTGHVQVNDYVTGLQLPQMNELAYNYETELMWCDIGGANNATIFASKWLNWARDQGRQVTFNNRCGIGGDFQTPEYATNVDTVVAKWESNRGMDPFSFGYNYRTPDSEYLNGNDIVQSLVDIVSKNGNFLLDIGPKHDGSIPDIMQKGLRDAGSWIIPHGESIYDTRYWSVTPGKGNFRYTTTKDAFYIHYLTKPGSTITITDPVPWLPGDTVTVLGGSQNGKVVQATKLGNNLVLQLNDAIINGDKYV |
| 206 | Unigene0008033 | 71 | 28913 | 13 | MSSAFPWGIETSRLDGKVALVTGSGRGIGAAIAQELARRGAKVVVNYANSSDAADKVVGAIKEHGGDAIALQADVGDVSQTIKLMDTAVEHFGQLDIVCSNSGVVSFGHLKDVTEEEYDRVMRINTRGQFFVAREAYKHLSVGGRIIMMGSITGQAKGVPKHAVYSGSKGAIETFVRCMAIDCGDKKITVNCVAPGGIKTDMYHAVCREYIPDGEKLSDDEVDEYAKTWSPMARVGQPVDIARVVGFLASQDGEWVNGKVIGIDGAACM |
| 207 | Unigene0002800 | 71 | 61648 | 4.7 | MKLHSLLALGAVAATAHAADDESTESLEANLPSFTPTTIKADFVEQFTDGWDARWKASHAKKEGTEEEWQYVGNWEVEEPSVFPGIKGDKGLVLKDKAAHHAISAKFDTPIDNKGKTLVVQYEVKLQNFLECGGAYLKLLQDGAVSSQDEFSNASPYVIMFGPDKCGATNKVHFIFKHKNPKTGEYEEKHMNGAPSAIVNKLSNLYTLVVRPDQSFEVLINGESKKNGTLLDDFTPSVNPPKEIDDAADSKPEDWVDEAKIKDPEAKKPEDWDEDAPYEIVDESAEKPEDWLDSEPLTIPDPEAQKPEDWDDEEDGDWIAPQVSNPKCDDVSGCGPWEKPMIKNPAYKGKWTAPLIDNPAYKGVWKPRKIANPDFFEDKKPADFEPIGAIGFELWSMNENILFDNIYIGHDEAQAKKFKQETFDVKKPNEEALDKASKPKVDDKKSPSDLVFLDDPILYIKEKTALFIELAQKNPVDAIKFVPEVAGTIGVVLVTIIALVFGLGGAAAPSKEEIKAKAKQAKDAAQKTKNDVADAVASGADKAQAEVNKRTTRSGAQ |
| 208 | Unigene0005433 | 71 | 20798 | 6 | HLSSDSTTRPDPRALCFGDIHTSTSQRPRSPHTIMADSLTEEQVSEFKEAFSLFDKDGDGQITTKELGTVMRSLGQNPSESELQDMINEVDADNNGTIDFPEFLTMMARKMKDTDSEEEIREAFKVFDRDNNGFISAAELRHVMTSIGEKLTDDEVDEMIREADQDGDGRIDYNEFVQLMMQK |
| 208 | Unigene0005434 | 71 | 21597 | 5.8 | GTRNSDTPLVVCVSGTLSSARRHALHTUYFGSRTLLTERSRUADSLTEEQVSEFKEAFSLFDKDGDGQITTKELGTVMRSLGQNPSESELQDMINEVDADNNGTIDFPEFLTMMARKMKDTDSEEEIREAFKVFDRDNNGFISAAELRHVMTSIGEKLTDDEVDEMIREADQDGDGRIDYNEFVQLMMQK |
| 209 | Unigene0008346 | 71 | 41094 | 7.3 | LSLLLASAANAVPINEQSNCACSSPRIRKEWRTLSSFEQEAYIDAIKCLKNSPSKGKEIFNTLESRYDDFVAIHINATRGGQDAPPLADLHLSENSTQRPPTIYGIHGVGVFLPWHRYALWIFESVLRSECNFTGTQPYWDWTLDNPASNGSLLGSPVITSFGGDGIGSSGCIADGPFNGSTFVNIGPLDSLARNPRCLTRFLSEDLFTQSGTWERIYPPTMSRTGYVQLQQFIDDLSFVPKDDTVTSVLFGNPHELGHSGIGGDMLDVYASPNDPLFWLHHAQLDYMWALWQKDDKARLVDIGGARTVQGFGPDADHAEPTTLDTPVWMGFMNEDVTVKAVMDTVNKDGKGVLCYEYDDSPALEGRDL |
| 210 | Unigene0007022 | 69 | 33164 | 5.2 | MKYIAETAALVAALSATGAQAAPSQNDKLFNSPRVLTLPYDGNPAFYADPKSFQGGRFASKRDAIIDNMPKVIPHPGGIEGAIADARRLYPEIYQRDAQGFGPEDFLGLPDTSINPNFGNTGTFGKRSAEAFIDNQPKVISGPNDWAEARRLYPEIFQRDEPKSSTPFAGFRRSREPARLPLLSGPAGPDLPNLGARDRRSPFQLQQRDAVADPFLGDIMKLVGMVIDKTTKRDAVADPFFADIVKLIGMGIDKATKRDAAADPFFADIVKLIGMGIDKATKRDAAADPFLGDIMKLVGMVIDKTT |
| 210 | Unigene0007023 | 69 | 33406 | 5.2 | MKYIAETAALVAALSATGAQAAPSQNDKLFNSPRVLTLPYDGNPAFYADPKSFQGGRFASKRDAIIDNMPKVIPHPGGIEGAIADARRLYPEIYQRDAQGFGPEDFLGLPDTSINPNFGNTGTFGKRSAEAFIDNQPKVISGPNDWAEARRLYPEIFQRDEPKSSTPFAGFRRSREPARLPLLSGPAGPDLPNLGARDRRSPFQLQQRDAVADPFLGDIMKLVGMVIDKTTKRDAVADPFFADIVKLIGMGIDKATKRDAAADPFFADIVKLIGMGIDKATKRDAAADPFFADIVKLIGMGIDKATKR |
| 211 | Unigene0006023 | 68 | 9973 | 21.1 | MSGLKAGEQFPEGVKFKYIPWTTADTKVCGFPVDYNTDKEFANKKVVLVSVPGAFTPTCSANHVPRFIEHIADLKQKGVDQLIIIAPNDA |
| 212 | Unigene0005171 | 68 | 29382 | 12.7 | MGRGPKKHQKRLSAPSHWLLDKLSGSYAPKASPGPHKLRDCMPLIVFIRNRLKYALNGRETKAIVMQRLIKVDGKVRTDPTYPAGFMDVISIEKTGENFRLVYDTKGRFTVHRITDEEASYKLGKVKRVQLGKGGIPFLVTHDARTIRYPDPAIRVNDTVKINLETGKIDDFIRFDTGVIAMATGGRNMGRVGVITHRERHDGGFNIVHIKDAIDNEFATRESNVFVIGREKPWISLPKGKGVKLTIAEERDRRRAYALA |
| 212 | Unigene0005172 | 68 | 32590 | 11.5 | MGRGPKKHQKRLSAPSHWLLDKLSGSYAPKASPGPHKLRDCMPLIVFIRNRLKYALNGRETKAIVMQRLIKVDGKVRTDPTYPAGFMDVISIEKTGENFRLVYDTKGRFTVHRITDEEASYKLGKVKRVQLGKGGIPFLVTHDARTUVSTLRLDRHSVVPKLWUIHPDADGINSIRYPDPAIRVNDTVKINLETGKIDDFIRFDTGVIAMATGGRNMGRVGVITHRERHDGGFNIVHIKDAIDNEFATRESNVFVIGREKPWISLPKGKGVKLTIAEERDRRRAYALA |
| 213 | Unigene0012761 | 68 | 85275 | 3.9 | MLAQRLAARQLPRLGRRTFATVNDSPLTKKVEMTNWEKGHYINYAGMNENLNIVRKRLNKPLTYAEKILYSHLDDPHGQEIERGKSYLKLRPDRVACQDATAQMAILQFMSSGMDAVATPTTVHCDHLIEAQVGGEKDLARAYDINREVYDFLSSSCAKYNIGFWKPGSGIIHQIVLENYAFPGGLMIGTDSHTPNAGGLGMAAIGVGGADAVDVMASLPWELKAPKVIGVRLTGKMSGWTAPKDIICKVAGILTVKGGTGAIVEYHGPGTENLSCTGMATICNMGAEIGATTSLFPFNDRMYDYLAATKRQHIGDFARGYAAELREDEGAEYDELIEINLSELEPQINGPFTPDLATPISKFADAVKENKWPQELKVGLIGSCTNSSYEDMSRAAAIAQDALDHGIKAKALFTITPGSEQIRATCERDGQLKTLEEFGGMVLANACGPCIGQWDRRDVKKGEANSIICSYNRNFTGRNDANPATHSFVASPDMVVAMTLAGDLTFNPLTDTLKDKDGKDFKLKEPTGDGLPSRGYDPGMDTYQHPPEDRSSVKVQVSPTSDRLQLLEPFEPWNGQDAKNVPILIKAQGKTTTDHISMAGPWLKYRGHLDNISNNLLIGAINEANGEANKVKNQDTDNWDAVPAVARQYKKEGVKWVVIGDWNYGEGSSREHAALEPRHLGGLAIITRSFARIHETNLKKQGMLPLTFQTPEDYDKIKPDDRVDLKLTDIGVGKPVTMTVHPAAGGQAFDITLVHTFNEGQLEWFRNGSALNTM |
| 214 | Unigene0010653 | 68 | 16623 | 5.2 | MKAAFTVLAIGATLAAAQDLASLPPCGQTCINNMLALAPSLGCPDAQADCLCRNADFGFGVRDCANQACGDASAAQQVIAYGTQYCGGALAGGDDSNGGAGGAGGAGGAGGSASGTESGATPSATGNGDDSEEGGAAGAAAITTSALTALVSASDSTFVTTTGFTTIFDTASG |
| 215 | Unigene0008323 | 67 | 36770 | 3.8 | MAPPAMSFEEQHAPVVDGYPSKLATLPHGAAIPTTNKLTSLESMAEDNWSNFNFAPIRESQVSRAMTRRYFADLDHYAESDIVIIGAGSCGLSAAYCLAKARPDLKIAIIEAGVAPGGGAWLGGQLFSAMIMRKPADAFLREVGVPYEEEGENYVVVKHAALFTSTVLSKVLQFDNVKLFNATTVEDLITRKGEDGQLHIAGVVTNWTLVSMHHDDQSCMDPNTINAPVIISTTGHDGPFGAFSAKRLVSMKHENLPALGGMRGLDMNSAEDAIVKGTREIVPGLIIGGMELSEVDGANRMGPTFGAMALSGVKAAEEALKVFDVRREERADKSFNKLNGK |
| 215 | Unigene0008321 | 67 | 36770 | 3.8 | MAPPAMSFEEQHAPVVDGYPSKLATLPHGAAIPTTNKLTSLESMAEDNWSNFNFAPIRESQVSRAMTRRYFADLDHYAESDIVIIGAGSCGLSAAYCLAKARPDLKIAIIEAGVAPGGGAWLGGQLFSAMIMRKPADAFLREVGVPYEEEGENYVVVKHAALFTSTVLSKVLQFDNVKLFNATTVEDLITRKGEDGQLHIAGVVTNWTLVSMHHDDQSCMDPNTINAPVIISTTGHDGPFGAFSAKRLVSMKHENLPALGGMRGLDMNSAEDAIVKGTREIVPGLIIGGMELSEVDGANRMGPTFGAMALSGVKAAEEALKVFDVRREERADKSFNKLNGK |
| 215 | Unigene0008322 | 67 | 36770 | 3.8 | MAPPAMSFEEQHAPVVDGYPSKLATLPHGAAIPTTNKLTSLESMAEDNWSNFNFAPIRESQVSRAMTRRYFADLDHYAESDIVIIGAGSCGLSAAYCLAKARPDLKIAIIEAGVAPGGGAWLGGQLFSAMIMRKPADAFLREVGVPYEEEGENYVVVKHAALFTSTVLSKVLQFDNVKLFNATTVEDLITRKGEDGQLHIAGVVTNWTLVSMHHDDQSCMDPNTINAPVIISTTGHDGPFGAFSAKRLVSMKHENLPALGGMRGLDMNSAEDAIVKGTREIVPGLIIGGMELSEVDGANRMGPTFGAMALSGVKAAEEALKVFDVRREERADKSFNKLNGK |
| 216 | Unigene0014233 | 67 | 30282 | 7.5 | MADRYSFSLTTFSPSGKLVQIEYALNAVNQGVTSLGIKAANGVVLATEKKSSTPLIDASSSSKVSLITPNIGMVYSGMGPDYRVLVDKARKVSHTGYKRIYNEYPPTRILVQDVARVMQEATQSGGVRPYGVSLLIAGWDDGILPEQEQAEAAEEMDLDDKKKKSGKTGGILKGGPSLYQVDPSGSYFPWKATAIGKSATSAKTFLEKRYTEGLELEDAVHIALLTLKETIEGEMNGDTVEIGIIGDPADHLLGYEGVEGATGPRFRTLSPQEIEDYLTNL |
| 217 | Unigene0013115 | 66 | 17451 | 18.5 | NPPSNPSLAETYSQVSIVPLSSTKRLISFAGQTGTGKDQAANAKLSFAEQIKIALEKVDKLLAATGAKKGDIVMIRPUVGDSLRLDWRIMGMNVECUYLGSYVVKLTSRSEEEIKSKDEIFLKWWRSTEGEKALPPPDSLLGVDSLWSKETLFEIEV |
| 217 | Unigene0013116 | 66 | 14597 | 21.8 | NPPSNPSLAETYSQVSIVPLSSTKRLISFAGQTGTGKDQAANAKLSFAEQIKIALEKVDKLLAATGAKKGDIVMIRPYVVKLTSRSEEEIKSKDEIFLKWWRSTEGEKALPPPDSLLGVDSLWSKETLFEIEV |
| 218 | Unigene0000002 | 66 | 39068 | 10.9 | MKASIQLLAASAAVTTARTISPNITTSPIDIPLKHWPEPQASQLRKMIAGNANSSNYACFDMDNTSYRFDLEESLLPFLENRGILTRDTMDQTLKLVPFADNAASNYTESLYSYYLRLCEIDDMICYPWAAQIFSGFTLRQLKTWVDELMSLNTTIPVQYLSTSENGTTTLESDQVSPPKLFRAQVELYNTLQANGIEVYVLTAAHEELVRMVASDPKYGYNVPPQNVIGVTTLLRNTTSGEVTTARKQIEAGTYNATANLDLVITPYLWTPATWFAGKWAAILTYIDEWKRPVLVGGDTPGSDGYMQFHGVDVAKGGVHLWVNKSASNWEEIQGMIKENTESQIKNGR |
| 219 | Unigene0014115 | 65 | 84936 | 3.1 | MPGVVKGPSTLYDKTFEQHIVDEKPDGTVLLYIDRHLVHEVTSPQAFEGLKNAGRKVRRPDNTLATTDHNVPTTSRKGFKNISDFVKEDDSRLQCETLEQNVKDFNLTYFGLGDKRQGIVHVIGPEQGFTLPGTTVVCGDSHTSTHGAFGALAFGIGTSEVEHVLATQTLMTKRSKNMKIEVNGELAPGVSSKDIVLHVIGVIGTAGGTGHVMEFCGSAIRSLSMEARMSMCNMSIEGGARAGLVAPDEITYEYIKGKPLAPKYNSPEWKKAIKYWENLKSDPDAKWDAVVTIDAKDIAPTVSWGTSPQDVVPITGKVPGPDDFDDKDKKLACERALKYMGLTAGTPMTDVVIDKVFIGSCTNSRIEDLRIAAHIVKGKKVASNLKRAMIVPGSGLVKQRAESEGLDKIFTDAGFEWREAGCSMCLGMNPDILSPGERCASTSNRNFEGRQGAGGRTHLMSPAMAAAAALAGKLADVRHLEGYEQALPQAKIAPTNVEAHSEDPESDVDLDKILDLPQNQDITASPSDVQKSASGSPGMPPFKQLKGLAAPLDRSNVDTDAIIPKQFLKTIKRSGLSAGLFYALRFNEDGSKNKDFVLNQEPWTNAKTLVVSGPNFGCGSSREHAPWALLDFGIKCVIASSFGDIFFNNCFKNGMLPIRITDQKVLQKLFDEASAGKELEVDLEGNVIKAADGSEITTFEVEEFPRHCLINGLDDIGLTMQDNDKIAKFEARRSEETPWLDGSGYLAKHRTGPVKVEAAPVPKTNRGEEKTEPLEW |
| 220 | Unigene0011945 | 65 | 21674 | 6.5 | MANPRVEELPDEEKKVQEVEEESSDSDEEGEANIPAGASVAVHSRNEKKARKAIAKLGLKHVEGITRVTLRRPKNILFVISNPDVYKSPNSNTWIIFGEAKIEDLNSQAQASAAQQLASQAANEAGHDHSADGDKGKAVEGGSDKKAEEEEDDGEEVDDTGLEAKDIELVMAQASVSRKKAVKALKENDNDIVNSIMALSI |
| 221 | Unigene0000858 | 65 | 30939 | 4.3 | VGTVTTSDGVKLAYIQSGPAAGQKLLFIPGWRQTAAEWRKQVEYFSGAGYQVTAYDHRGHGDSEKTNFGYRLSRLGADLNDVINTLALSNVSIIGHSMGSSVTWALWDQYPEQHSRIHKFVLVDQSSVLVQDPTWTQAQRDTWSAALFTPAQTYTFSANMSNELIPFVTSMFTKDISVEDLDWAIAQNLKMSDEHAATLLINHAFMDWRDVLLRIDVPTLVLSGDASLNNATGIDWAATQIPGARSYTFTKEERGSHFVFWENPERFNQVVREFVT |
| 222 | Unigene0012517 | 64 | 30003 | 7.1 | MSSLAKFIIAGLGATAGVTAYPQEQAPSNTVPVISQPPTPSPVASSAAASASAANVALAKEALQAVTNVGRFNALLTVDGAGRQLLPDDTLRSRLVFDYGARANPLGQGGRFILANQNNFPILVNQGISTAVGFLNPCSMNSPHTHPRATEFFTVVQGKFQTGFMLENGFLANATEGRQTTQVSVQELGPFQATVFPLGSIHYQFNHQCEPATFIATFNSADPGTSQVAQNYFFLDSGVVNVTLGEINQIDGTNIEQFRTSLPANLVQAVDSCLARCGIEKK |
| 223 | Unigene0002301 | 64 | 104489 | 1.4 | SVTTRRTPTSRYPNPVNAARSSKFAARLHPFPPVLANRSCSSHASSTLVKMASDRDILSDHVKPINYAISLTDLTQGEPWTYQGKVDIEVEVKKSTKSITLNTHELKVHSAELVADSGKHSSAVKASDISYDTKHQRCTFTFDQELAQSPKAVLSIAFEGIMNNHMAGFYRSKYKPTVPASQGVARDSEWHYMFSTQFESSDARRAFPCFDEPNLKATFDFEIEIPEDLVALSNMPEKESRKSKDGHKIVSFDRTPIMSTYLLAWAMGDFEFIEDFTRRKYNGKSLPVRVYTTKGLEQQGQLALESAHQVVDYFSEIFEIDYPLPKVDLLAVHEFSHGAMENWGLITYRTTAVLFDEKNSDQKYRNRVVYVVAHELAHQWFGNLVTMDWWSELWLNEGFATFVGWYAVDHLHPDWNVDAQFVTEGMQMAFQLDSLRTSHPIEVLVRNALEVDQIFDHISYLKGSSVIRMLASHLGHKTFFKGVADYLKANKYSNATTNDLWSALSKASGQDVTGFMDPWVRKIGFPVVTVAEEPGQISVQQSRFLSGGDVKPEEDTTTWWIPLGLKTGPQATDAKREALTVREDTYRDIDTSFYKVNGDQAGFYRTNLPPPRLVELSKHLDKLSIQDRIGLVGDAGALAVSGHGTTSAVLSFVEGFHVETNYLVWSEVLTTLGKIRRIFASDDEVSHGLRAYTLKLVSAATEKVGWTFAPNDDYLTGQLRALLISSAGLAGHESVVAEATKQFDAFVGGDAQAIHPSLRAAVFKIAIKNQGEKAYRAVQKEFLSTKSIDGREITLASMGSVPTKELANDYLKFAFAGNVAIQDLHTVGASLANNSKVRSTVWEYIKSEWPAIREKLGGNMVVLERFLRMSLQRFADAKVEQDIAQFFGDKDNTGYDRGLAVVSDTIKGSAQYKERDLENTREWLKAHNYIK |
| 224 | Unigene0005766 | 63 | 12139 | 28.8 | MYGLFHLAVLSATALSASALPQQQETPGVNKDFICPPEDMKRTHCMGPFDCLYALPGNCLGYIHCQPKDTTYETGIAYERPCRAGQWWNDEKKYCDTVEPPICK |
| 224 | Unigene0005767 | 63 | 12139 | 28.8 | MYGLFHLAVLSATALSASALPQQQETPGVNKDFICPPEDMKRTHCMGPFDCLYALPGNCLGYIHCQPKDTTYETGIAYERPCRAGQWWNDEKKYCDTVEPPICK |
| 225 | Unigene0014281 | 63 | 74609 | 2.8 | MARFSRSQSGPSRSTVSPWMTAFYICAILLAPMLFMGMIPSAHAQDAEKADTSVTGPVIGIDLGTTYSCVGIMKGGNVEILVNDQGNRITPSWVAWNDDERLVGDAAKNQFASNPHRTVFDIKRLIGRKYEEKDVQKDIKHFPYKVVNKGGQPRVKVDVKGDEKTFTPEEISSMILGKMKEVAESYLGETVTNAVVTVPAYFNDAQRAATKDAGTIAGLNVLRVVNEPTAAALAYGLDKTDKERQIIVYDLGGGTFDVSILTVDQGVFEVQATAGDTHLGGEDFDQRIVDYFTKLYNKKHSTDITKNAKTMGKLKREVEKAKRSLSSQMSTKIEIEAFHEGTDFSETLTRAKFEELNNDLFKKTLKPVEQVLKDAKLKKSDIDDIVLVGGSTRIPKVQAMLEEFFGKKASKGINPDEAVAFGAAVQGGVLSGEAAASEVVLMDVNPLTLGIETTGGVMTHLIKRGTTIPTKKSQIFSTAADNQPTVLIQVFEGERSMTKDNNQLGKFELSGIPPAPRGQPQIEVTFELDANGILRVSAGDKGSGKSESITITNDKGRLSAEEIERMVEEAEKYAEEDKATRARIEARNGLENYAFSLKNQVNDEDGLGGKIDEDDKEALLEAVKEATDWLEENAQTAEAEDFDEQKQKLSDVAYPITSKLYGGGGAGGMPDYGEDDEPSGHDEL |
| 226 | Unigene0014159 | 63 | 83553 | 2.9 | MAPSFENLDPETEYEVDDEEIDFSDLREKFETVMEEGLDTFIVIDGLPKVPEASKDKLIKFLLRSLTKVGKTKEENVFMPLSGETGQTEGFAFVEYETPAQAAAAVKALHAQPLDKKHTMAVNKLTDIERFGREGRIDETYKAPEIAPFEEKEHLRWWLGDADGRDQFVMYRGDNVGVFWNEREAAPEQIVDRQHWTESFVQWSPKGTYLTSMHAQGVQLWGGPQWSRQKRFMHPGVNLVDFSPDEKYVTTWSHRPMVVDEGNPILSLEEDGKNYIIWDVATGKPLRSFVTLDLPSPGLDPEGNPIKQKIQWPAFKWSADSKYVARMTPNQSISVYELPSMRLMDKVSVKIEGVQDFEWCPANPKRNEHKEYEQLFAYWTPEMGSNPAKVGVMSIPSKEIVRTRNLFNVSDAKLHWQSDSKFLCCKVDRHSKSKKSLATNLEIFRVQEKGVPVEVVDSLKDTVINFAWEPKGDRFVLITAGEVPAGAAIPPKTSVSFFAPEKAKGNAVGNFKLIRTVDKKNNNAIHWSPNGRFCIVATVLNQQSFDLDFWDFDFEGEKDEKEKDLTANLQLMNTADHYGITDIEWDPSGRFVATSASVWKHRMENGYHLYSFAGQLLREEPVEGFKQFTWRPRPERLLSKEEMKEIRKNLREYSKTFEEQDQAKKSSADKEVIENRRRQLEEWLAWRERTKEELLDERQDLGLPEISPEQQALVVEDSEGDSKVV |
| 227 | Unigene0001105 | 63 | 28116 | 3.9 | MFRNNYDNDSVTFSPQGRIFQVEYAQEAVKQGSVVVGIVSKTHAVLAALKRNAEELSSYQRKLIEIDNHYGIALAGLASDARVLSNFMKQQSLASRLTYGRPIALSEITSRIGDRAQTNTQHYGKRPYGVGLLIAGVDAKGPHLFEFQPSGVTQEMIACGIGARSQMARTYLERNLDEFEGSSREELIKHALRALKDSLSQDKELTVDNTSLGVTGMGENFKLYEGQEISGWLETTFENKPEEGESTEGMEVDS |
| 228 | Unigene0011838 | 63 | 22563 | 4.6 | MVNLRTQKRLAASVTGAGKRKIWLDPNEVNEISNANSRQTIRKLLADGLIIRKPVTMHSRARARELTAARRIGRHRGFGKRKGTADARMPTQVMWMRRLRVLRRLLVKYRAAGKIDKHLYHELYHLSKGNTFKHKRALVEHIHKAKAEKQREEKLKAEMDAKRAKTKAARERRQERITAKRNALAGEDDEEPAAQ |
| 229 | Unigene0003899 | 62 | 19429 | 13.9 | APKSNNPYKNLLHKLYSFLDRRTESKFNATVLRRLRMSRINSPPISLSKIVAVTANKHSGPAHEGKIRAVVGTVTDDNRLLEVPKLSVCALRFTAPARARIEKAGGECLTFDQLALRAPTGSNVILLRGPKNAREAVKHFGFGPHTDKKPYVESKGRKFERARGRRRSRGFKV |
| 229 | Unigene0003898 | 62 | 21254 | 12.7 | MGVDLDKHHVKSGHRKAPKSNNPYKNLLHKLYSFLDRRTESKFNATVLRRLRMSRINSPPISLSKIVAVTANKHSGPAHEGKIRAVVGTVTDDNRLLEVPKLSVCALRFTAPARARIEKAGGECLTFDQLALRAPTGSNVILLRGPKNAREAVKHFGFGPHTDKKPYVESKGRKFERARGRRRSRGFKV |
| 230 | Unigene0011173 | 62 | 57019 | 8.1 | MCEEKHNTTMHWHGLTQRLAPFSDGTPQASAWPIPPCHYFDYEVFPLRSECGTYFYHSHVGFQAVSSTGALIIDCPDPQPYQYDDERIIELSDYFNHTDERIEKGLIAKPFVWSNETNAILINGVGVAQEEAHRAGTEGCSLPVIEVEPCKTYRFRFIGSLAISLVQLGIDQHENFTIIEADGSYTKPHTEKFMQITTGQRFDTIFKAKDEAELNGKRDYLIQFVTKDRPKSYTGYGILRYKGGAPEITKGPDTPPLTFSNKTYEWAEFALQPLHDNNFPRADEVTRRIELDNRQMSTDSIVWRTNGLEWNETTPPQPGDVPYLVDIYQRGEAAMPNYTAALANNGWDPTTYTFPAKLGEVLEIIWYNTGSLVKNNGGQDFHPLHAHGAHYYDCGSGNGTYDPIEHEKKFLNYTPVLRDTTTLYRYIDKTIAGERLGYRVWRLRVTDAGVWMIHCHILQHMIMGMQTAWIMGDLEEIRRVPLFDARAYLDFGGGAF |
| 231 | Unigene0005895 | 62 | 24008 | 10.8 | MASSIHVSSHPCVRAKLSQLRSASTNARDTKTLVHEIATIVGVEALAKGLEVENVGTDKSPLGYTYNTEDISPSNISLVPILRSGLSMVEAVQNLLPNPVSVHHLGMYREKSTLQPVEYYNNLPYHKAGETSSVPPLAIIVDPIIATGSTVCAAIETLKDWGVQRIISISILASQTGLQRAAEAWPEGVELWVGGVDAETDERGMIKPGLGDIGDRLYLTLGK |
| 232 | Unigene0001119 | 61 | 54675 | 5.6 | MRGLFARLLAWSQAPLSPQFTSPEAPDKPHTTGIRSIETEIAFVADSDNKCNSDRYDPYVCECLGLNGCWFPSEVQEGPPYSHDEPWPQRSKTCNVPAGGDPKGDDAPAIIQAFKECKEDGHIIFDNTTYYIGSIMNTTGLKDVTIELRGTMLWDTNIPYWLANSMPIGFQNQTSAWHLGGENLHFFGNGYGTIDGNGQAWYDFNQGVSNRHGRPHSLLITNTKNAVIEGLRFIKPQMWTMTVARSEKMLLQDIFINATSDTRDFRANVNTDGVDTVYANNITFLRWTVDNGDDSISMKQNSTNIYIEDCTFYNGQSLAMGSIGQYPGQIEVIENVTAHNIKCFNTGYGGRVKTWTGKNKGFPPNGGGGGLGWARNITFTNFELNDVNLAWAISQCTSYNGQRGDCNSSDFKVSDMHWGNARGTLKGDRVATLQCSAEAPCSNINLFDNELWALDQDILSYSYLCEAVENTVGFNCTGACNGQCPR |
| 232 | Unigene0001118 | 61 | 54675 | 5.6 | MRGLFARLLAWSQAPLSPQFTSPEAPDKPHTTGIRSIETEIAFVADSDNKCNSDRYDPYVCECLGLNGCWFPSEVQEGPPYSHDEPWPQRSKTCNVPAGGDPKGDDAPAIIQAFKECKEDGHIIFDNTTYYIGSIMNTTGLKDVTIELRGTMLWDTNIPYWLANSMPIGFQNQTSAWHLGGENLHFFGNGYGTIDGNGQAWYDFNQGVSNRHGRPHSLLITNTKNAVIEGLRFIKPQMWTMTVARSEKMLLQDIFINATSDTRDFRANVNTDGVDTVYANNITFLRWTVDNGDDSISMKQNSTNIYIEDCTFYNGQSLAMGSIGQYPGQIEVIENVTAHNIKCFNTGYGGRVKTWTGKNKGFPPNGGGGGLGWARNITFTNFELNDVNLAWAISQCTSYNGQRGDCNSSDFKVSDMHWGNARGTLKGDRVATLQCSAEAPCSNINLFDNELWALDQDILSYSYLCEAVENTVGFNCTGACNGQCPR |
| 233 | Unigene0001493 | 61 | 59454 | 2.1 | MQRVLSSTPRAAARQARIRPGSLTQQRFAHKELKFGVEGRAALLTGVETLAKAVATTLGPKGRNVLIESSYGSPKITKDGVTVAKAIQLKDKFENLGAKLLQDVASKTNEVAGDGTTTATVLANAIFSETVKNVAAGCNPMDLRRGTQAAVEAVIEYLRANKRDITTSEEIKQVATISANGDTHIGGLLATAMEKVGKEGVITVKEGKTIEDELEVTEGMKFDRGFISPYFITDTKSQKVEFEKPLILLSEKKISAVQDIVPALEASQQQRRPLVIIAEDIDGEALAVCILNKLRGQLQVAAVKAPGFGDNRKSILGDIAVLTNGTVFTDELDIKLEKATPDLLGSTGSITITKEDTVILNGEGSKDMVTNRCEQIRGVMADPTTSDYEKEKLQERLAKLSGGVAVIKVGGASEVEVGEKKDRMVDALNATRAAVEEGILPGGGTALLKAAANALSGVKTANFDQQLGVSIVKQAITRPARNIVENAGLEGSVVVGKLMDEFGKDFNKGFDSAKGEYTDMIAAGILDPFKVVRTGLSDASGVASLLGTTEVAIVEAPEEK |
| 234 | Unigene0010001 | 61 | 59061 | 4.6 | SAAVQTTFWNGYRGEEPQWRYTGPDNSPEFWFKETQDALATMQATFWNGTYWPSTIQWIGALIDTIVASTERTFVDGLQVYGDDVKVQLEGDIRNYYSQIQAYYDTEDTIQIFDAAYDDAQWVVMEWLEVIRFINQYEAYSQSGLGIDDISAYAHRAHIFYNIVQDKFNTSQCEGGLTWNPALETYKNAITNELFVSSSIMMYFFFPGDSDTDPYPHPNYTAQTNKTLPPLQPMAAHDPIFLKNAQDEWAWFKTHNFTNAQGLIVDGFHITPNQTTCDERNEMVYTYNQGVMLTGLRGLWEATGLISYLDDGYSLIETVINATGWDANVSGQASEWAGLGRNGILEDYCDAPANCSQDNLIFKGAYFQHFDYFCAPLPTDVPLVDGISVLASTELAQHHNDRCASYSPWVQHNAHAALSTRDGTKVMGEWWGAPYLNRSQGPAPKYAESKPVGSIDICNQPQLLDTYPWKCSSSDCAHYSASKRRHRHYPRAAADNRTVETQAQGLAVLRAATDLTLIN |
| 235 | Unigene0004697 | 61 | 75339 | 3 | GLHQCRYGYRDFYGVAEVVANYSKANIPLETMWTDIDYMYARYIMTTDPDRFPIERVRDIVDYLHENHQHYIVMVDPAVAYQEQKYDNLTYTTFTTAAENGYFVYKNGSIYKGVVWPGVTAFPDWFHPEVQQWWNEEFSAFFDRDTGVDIDGLWIDMNEAANFNYYGDNPEETQEERGFPPTRPALRSQPRPIPGFPAEFQPGAQDYPPDDLAYAPPWLAPDSNPNDAKKRSVSTNAQVAQPVKRQASSAPSGAELIGYPNRNFLAPPYQIDNANTVEAVGGLSNFTLDTDIVHYDGHVELDVHNLYGTQMSEASRTAMLARRPERRPLVITRSTFAGAGRSVGKWLGDNLSNWDQYRNSIQGMLNFASIYQVPMVGSDICGFGANTTETLCARWVTLGAFYTFMRNHNGDTSIPQEFYLWDTVAEAARNALDIRYRLLDYIYTGFHKQSKDGTPVLNPLWFLYPEDENTFGNQLQFFYGDSLLVSPVTEENATSVSIYLPDDRFYTWGSWEVVEGAGAAVNLTDIGFTEIPLHVKGGSILPVRQKAGYTTTETRKSPFELIVAPGRDGKASGSLYLDDGDSLVQAATSEITFEYENGELKIGGTFGYTTDALVTTVTVLGGGSGGGNAPLYYKGSTKTKTCKDDEWQYDAGKGVKTAQVNEALDGEMVVRV |
| 236 | Unigene0006617 | 60 | 41987 | 2.8 | MAANDETYRASTFAPVNIAIIKYWGKRDTKLNLPTNGSISVTLSQDDLRTHTTASCSAALTDDSLTLNNAPQDISGARTQACLRELRSLRKEVEDADSSLPKLSELKLKIVSENNFPTAAGLASSAAGFAALVRAIANLYELPTSPTDLSRIARQGSGSACRSLMGGYVAWQKGEQPDGSDSVAYEVASASHWPDMRALILVASAEKKGVSSTAGMQQTVASSALFQHRAEEVVPKRMKAMEKAIHNRDFESFAKLTTKDSNNFHATCLDTDPPIFYMNDTSRAAVRMCEYINASHPEEKQYCAYTFDAGPNPVVYYLAEHEDIVAGTFKSVVGDKTGWEGARGQKIKATPLPEGAEVAAEKLNSGISRVILTSVGDGPRKTDKHL |
| 236 | Unigene0006616 | 60 | 39766 | 3 | RYWGKRDTKLNLPTNGSISVTLSQDDLRTHTTASCSAALTDDSLTLNNAPQDISGARTQACLRELRSLRKEVEDADSSLPKLSELKLKIVSENNFPTAAGLASSAAGFAALVRAIANLYELPTSPTDLSRIARQGSGSACRSLMGGYVAWQKGEQPDGSDSVAYEVASASHWPDMRALILVASAEKKGVSSTAGMQQTVASSALFQHRAEEVVPKRMKAMEKAIHNRDFESFAKLTTKDSNNFHATCLDTDPPIFYMNDTSRAAVRMCEYINASHPEEKQYCAYTFDAGPNPVVYYLAEHEDIVAGTFKSVVGDKTGWEGARGQKIKATPLPEGAEVAAEKLNSGISRVILTSVGDGPRKTDKHL |
| 237 | Unigene0006472 | 60 | 19388 | 14.4 | PAPQAGSSPQEPLKFAGMSLRSASPIHFGQINANGSEFLVGAEPATYKPDVIEGDFNNQTVFTYVNGAGTIGLLTSVPGGQQIYVTEGNETIGQVAGQLKFTQAHTARTDGPAIYTGFENVYDATLKFENSSWVACPSPRIEGAYTVYAASRFTNATDECLGFSWRIVQLADNTTSAWQY |
| 238 | Unigene0009143 | 59 | 26981 | 3.3 | FDMNDPTDSGHVQQLKDGQTDAVQLASYLEQSWSNAVDNGIYGHYFGDADKDAVQKVYKSVCSRCSPSUPLYUPATLTDIWGSDDQEGSIDLGYIFWTNKDIKGICTTPGTEKVGDSITLAYIGNLVRNNQQIAIAHMCDQGYDVPKRSDVDCDSLGSTVSTLMDILGAIILHEYTHFDRIGQAATGNHITDKAYGPLDVKGLSDADKLINADSYRWLALEYYWTTICGKPFSGPRDDRDN |
| 239 | Unigene0013964 | 59 | 33870 | 4 | ANMTTAIFKHIDTTTYTGKPWGKVDADATSFKLEDVERNVHNIRGREHDFTTDNSGFAVYNSPAKEKLFTDDNAVREGYYAEVEDLLRKNIKGIKKVHIFDHTIRRKDKASPRQPVQQVHVDQTAGATDVRVRRHLSSEADELLKGRYQIINVWRPIGHPASDFPLAVVDWRSTQPKDFIPVDLLYPVRPDSAMDDDDRGKERLADPATLQSTEGYEARGETLGVAANENHKFYYMKDMTPDEVMLLKCYDSFGVGEKFEHEGIATRTPHTAFFDPNTPADAPGRQSIEVRCLVFYE |
| 240 | Unigene0005174 | 59 | 16060 | 8.7 | MAPKKAAPRVQENVQLGPQVREGELVFGVARIFASFNDTFVHVTDLSGRETISRVTGGMKVKADRDESSPYAAMLAAQDVATRCKELGITALHVKIRATGGNGTKTPGPGAQSALRALARSGMKIGRIEDVTPTPSDSTRRKGGRRGRRL |
| 240 | Unigene0005175 | 59 | 21421 | 6.6 | MAPKKAAPRVQENVQLGPQVREGELVFGVARIFASFNDTFVHVTDLSYVUSSQNQRLATILETQKRQKRPLEGLAGLHDTKLFLHGSUADLGDSSGRETISRVTGGMKVKADRDESSPYAAMLAAQDVATRCKELGITALHVKIRATGGNGTKTPGPGAQSALRALARSGMKIGRIEDVTPTPSDSTRRKGGRRGRRL |
| 241 | Unigene0002275 | 59 | 76956 | 0.9 | VMELRSARPYPYKLRAESTALIIIDMQRDFVDFNGFGQIQCGNDDIFKKVRNIVPRTQKALEAARSLGLYVVHTREGHKPDLSDLPPSKQLRQISAPSGHHTMGIGDQGPMGRLLVRGEYGHDVIDELRPIPGEPVIDKPGKGSMWDTNLHRTLLARGITHLLFAGVTTECCVNTTARECADRGFETCILSDCTDGFDQGFYTSTLDMLCSYDGLFGFVGSSTELLKYAPTLTQTPPTTPPGFSGDVSIAGLRKQYTSGQLRPTDVAKEISARLLSYKEKDSAVWIQVQDPAELLKAAQAVEDRFAGNPLPELYGTFFAVKDNIDVANITTTAACEAYAYVPKENAEVVRAMIDAGAIFVGKSNLDQLATGLSGCRSPYGTPHSVFSSQHISGGSSSGSAIAVGAGLVSFALGTDTAGSGRVPAGYNGIVGHKPTKGTLSAQGMVPACKSLDTITVFATTVEEARKVWLVADQGPDENDPYAKSQQSLALWHADFRGVKTGGFTFGIPPSTALQQCDEWSRKLFAKSVARLERAGGTSREVEWESFEGGSNILYDASLVQERIACIGPEFIERNLETLHPTTKELLSAATNSAIKPWEVFRDQHLQAQYTRDAAKVFKEIDVLLVPTTPCHPTIAEMEEDPIGLNAKLGYFTHFANVLDLCGIAVPAGTYQDRSGITLPFGVTLLGASGKDGRIFDIAREFERT |
| 242 | Unigene0014539 | 59 | 41695 | 4.7 | MASTFPPPPVNTIDWDDIGFKVREVNGHIESTYSVKTGEWTAPKFVQDPFLRLHGMAPGLNYGMQCYEGLKASRGPDDKTIGIFRPQANAERMQHSASFVSMPDVPVELFKKSVHLAVSLNAAYVPPHRTGASMYIRPLLFGSSAQLGLDPPEEYTFLVFVMPTGVYHGVHPVACLILEDFDRAAPEGTGSAKIGGNYAPVLRHSGKAKKEGFGITLHLDSRTRTEVDEFSTSGFIGIHGSPEEGCTIVVPDSKNVIKSVTSNTICEIAKSWGWKVEQRPIKYNEIGTFTEIAAAGTAAALVPIKSITMRSKNDTFKYQGGGDEPGPAVVKLLAQLKGIQQGKIKDEFGWVEQVREYKPDEYAHDGAGQSNGVNGATPNELP |
| 243 | Unigene0014351 | 58 | 33365 | 12.7 | NTVHVQGISGQTSEKEVRDFFSFCGKIQSISVTPESNEASSSQSATVTFEKETAAKTALLLDNTQLGPSQVHVTPGKSLDQAAGGKTAGASDDDLAQEDKPRARVAAEMLAHGYNLSDQVIQKALALDQQHGISQRFTSALTNFDAKYKVTEKAQATDNQYGITAKANEGWRGLNSYFEKALGTPTGQKVRAFYDQGSKQVLDVHNEAKHLASLKTGKAPNHPVEGKEGRTECNCGSAAVKCSCKPGECACSSCSKNPDDSKPAVSAEEAELEKVPGTEKTKCNCGGADEKCACEPGKCACASCPRSS |
| 244 | Unigene0006782 | 58 | 22145 | 18.3 | MAPRSYSKTYKVPRRPFEAARLDSELKIVGEYGLRNKREVWRVQLTLSKIRRAARQLLTLDEKDPKRLFEGNALIRRLVRVGVLDESRMKLDYVLALKVEDFLERRLQTCVYKLGLAKSIHHARVLIRQRHIRVGKQIVNVPSFIVRLDSQKHIDFSLTSPFGGGRPGRVRRKKAKAAESKGDEDAGEEEE |
| 244 | Unigene0006781 | 58 | 19540 | 20.7 | DSELKIVGEYGLRNKREVWRVQLTLSKIRRAARQLLTLDEKDPKRLFEGNALIRRLVRVGVLDESRMKLDYVLALKVEDFLERRLQTCVYKLGLAKSIHHARVLIRQRHIRVGKQIVNVPSFIVRLDSQKHIDFSLTSPFGGGRPGRVRRKKAKAAESKGDEDAGEEEE |
| 245 | Unigene0000635 | 58 | 8720 | 17.2 | MSAPNADKPNEGVIGQIGNSLNNAANYVSETVQGKSAEASKEANKEKAKGNTGGTIGDRISGAAGAVSDGLEQKKHEGSAKANKEGI |
| 246 | Unigene0009554 | 57 | 57703 | 3.6 | MKYSLGLLGLAALAAASDVHDLTKDTFDDFVKENDLVLAEFFAPWCGHCKALAPEYEEAATTLTDKAIKLAKVDCTEQQELCQQYGVEGYPTLKVFRGPENIAPYQGPRKAQAIVSYMVKQSLPAVSILSTAEALEEFKTADKVVLVGYFGADDKSSNATFTELAEELRDNYLFAATSDAALAKAEGVEQPAVVLYKSFDEGKTTYTEGFSKDALVTFAKAAATPLIGEVGPETYSDYMSSGLPLAYIFSETAEERESLAKDLKKLAEKFKGKINFATIDAEAFGQHAGNLNLEVGKWPAFAIQDTAKNQKFPYAEAGDVKKLTEKLLSKFVEDYSEGKLDPSIKSEPIPEKQDGPVTIVVAKNYEEIVLDDKKDVLIEFYAPWCGHCKSLAPKYDELGTLFKSHSDKVVIAKVDATLNDVPDEIQGFPTIKLYKAGSKDAPVDYSGARTVEDLVNFVRDNGSHGVDVSDAIGADAEDVDTEGMPQQAAAATEAAEGVAEKVKEKVKEAGEAVKQAVLDDDEVADHDEL |
| 247 | Unigene0001300 | 57 | 51673 | 5.5 | RTFASTTRMGRIESDAFGELEVPDDKYWGAQTQRSLGNFNINQPQDRMPPPIVRAFGVLKGAAATVNMKFGLDPKLGKAIQQAASEVASLKLVDHFPLVVWQTGSGTQSNMNANEVISNRAIEILGGKMGSKKPVHPNDHVNMSASSNDTFPTVMHIAAVLDFEESLLPALKNLKEAMKHKAASFENIIKIGRTHLQDATPLTLGQEFSGYVTQLEYGIERVESALPRLRLLAQGGTAVGTGLNTFEGFAEDIATEVTNLTGHQFYTAPNKFEALAAHDAIVEAHGHLNTLATSLYKIAQDIRFLGSGPRCGLGELNLPENEPGSSIMPGKVNPTQCESLTMICCQVFGNQAATTFAGSQGNFELNVFKPVMIRNLLHSSRLLADGMNSFRENLVLGLEANEERIANIMKESLMLVTCLNPVIGYDMASKVAKNAHKKGISLKESAMELKALSEEKFDEVVRPELMIAPKAKK |
| 248 | Unigene0000647 | 57 | 58041 | 3.5 | MVQVKQKVALICIDGWGIPSESSNPEGNAILNAETPWMDEFAKDGSKEAQGYTELEASSLAVGLPEGLMGNSEVGHLNIGAGRVVWQDVVRIDQTIKKGEMNKVENIVKSFTRAKEGNGRLHLCGLVSDGGVHSHINHLKSLLKVAKEIGVPKVYIHFFGDGRDTDPKSGKGHLEDLLAFLKEEQIGKLATIVGRYYIMDRDKRWDRVEIGLKGMVEGEGEETDDPVATFQKRYDAGENDEFLKPIIINGKEGRIQDDDTVFVFNYRSDRVREFVQLLGDQDRSPKPDFPYPKNINITTMTRYKTDFDLPIAFAPQVMDDVLADWLSKKGCKQTHIAETEKFAHVTFFFNGGQEVQYENEDRELIDSPKSVATYDQAPKMSAMGVAERMAEKIGDGKYEFVMNNFAPPDMVGHTGVYDAAVIGCAETDKAIGVVYEACKKHGYTLFVTSDHGNAEEMLTEEKTPKTSHTTNKVPFVMANAPEGYSLKKEDGVLGDVAPTVLDIMGLDQPEAMTGHSLLQRK |
| 249 | Unigene0003021 | 56 | 64415 | 4.7 | YSKLPVDENETSGLQDEQPYRPSWHRKVVALVLVATFCLTNPWAPTLPYFAGKHHKCHHGTQYKGEKISWQKAGEIEGRHLETSSIVVPMDQFNLTNSGDKTFNISLVRLRGKDGSPNLLLNPGGPGGSGAKFVYRRGKQLSEIVGDGYHLLSFDPRGINGSGPLASCYPDKKAAQQLSDVRDTEIVHDSPEVYAWTQNFVKACEATTGEHAGYINTPQTAADMNSILDAVGQEDLAYWGFSYGTILGQTYASLFPNRSTRVIIDGVANNFVWYGDVFDGEQFTNTEDVLEGFFDECIKAGKNCSLSSHAKTKDELHEKVFGYLADLKAQPLSVFLNASDYGLLTYENILFDGIFPSLYKPASWYNLADNLAQLLSGNATAAWVAYGKNGGFGIEGEGNQFVTSNDGLSGPASGWPQDRETLLKQILSHVKESIFVPTENSGYYLRQQWTIRRTHNFTQKVGVETAHPLLILSTSYDPICPLVSAVSAFDAFEDSALVEVKGYGHCSVAVTSNCLAKHVREFLYNGTLPDGHVTCDVDGPYFIKPEEDGKVVAQKHFDDAQDQKIHLAQLEMARDWEW |
| 250 | Unigene0013245 | 56 | 13403 | 10.3 | MSFQDPKKTSQLDAPKIHKIRITLTSRKVQSLEKVCSELIERAKSKELRVKGPVRMPTKNLKISTRKTPCGEGSKTWDMYEMRIHKRLIDLNAPTEVVKQIIINIEAGVEVEVTIAA |
| 251 | Unigene0007971 | 56 | 75988 | 4.9 | SHACVREREFHSHGLTKRSVTPRAELSPDEQLIVDSFDANEISDWSYYYTHGNHIAGKNESQAQWTADRWAEAGFTTRLDAYNVYLNYPVSRSLALTWPNGTQYTPSLEEAVLEEDDTSSYPDRIPIFHGFSANGDVTAEYVYVGRGQQVDFERLKALGVPLEGKIALARYGGPFRGLKVKNAQDNGMIGAVLFTDPGDDGNITVAKGYAAYPDGPARNPTAVQRGSVQFLSTYPGDPTTPGYPSRKDSPRADKGAVTPRIPSLPISYAEAQPLLAALDGYGTNGSAVNRTNWVGALNATYSTGPAPGVTLHLTNQMEDAYTDIWNAIGIINGTSSDEAIVIGNHRDAWIVGGAADPNSGTAVIVELGRVFGKLLEQGWKPKRTIVLASWDAEEYGLIGSTEWMEEFLPWIKKSVVANLNIDVAVSGPHPDISATPELHRIAQDIMKKVLWPALEGNSTMYDVWSHDTNRTVGVLGSGSDYTSFVHRGIAAIDMGANPGPTDPIYHYHSNFDSYNWMSKFADPGFLAHKSMGQYLALLAYHLASDDLLPLEPINYADQMQRYYQTLQYTIGNSSRVDFDVSSLQDAISTFRTQAQEAADLAQLAIDSNNSELLQVVNAKYRDFQRGFTSQGGLPTRDFYQHLIFAPGLDTGYAPVTFPGVTEAVERDQWELAQDYLERTVRAIEVAGNILKS |
| 252 | Unigene0010758 | 56 | 64559 | 1.6 | KNGFAPQANIKQAAVHHHHPERRMVPAPADYDTTHSKETRKYLRTYGLTPPAVESFDNQAKRCLAVLESRKTPMEKYQYLSVLRNTNVNLFYRLFAHNVKELTPLIYTPTVGEACQRWSELYTQPEGLYLSWEDKGHLHSVIQNWPHDVEITVVTDGSRILGLGDLGINGMGIPVGKLSLYTACAGIHPLRTLPLCLDLGTSNEQFRNDPLYLGSRRDKVTAEEEKEFLDEMMAALTERWPSIVIQFEDFKNPFPSLERYQDTYTMFNDDIQGTGAVITGGFINAVKQSGVHPAEQRAVFLGAGSAGVGVAKQLTEFFVKEGLSEEEAKKRFWLVDSQGLVTLDRPGRPLAEHKVYFARDDNDGQQFQTLEEVIEYVKPTILIGLSTIGGAFTPEILTRMAELNDRPVIFPLSNPSSKSECTFEDAIKYTKGRCLFASGSPFPTLTYEGKELVPGQGNNMYVFPGIGLGAILSKAVNVTQNMIYASAESLSTSLTESEVADNWLYPDIRRIREVSVVVTRGVIRAAQKDGMDRELALRNLSDEDLDDYIRARMYDPFKEHEKIGDEIKELVNG |
| 252 | Unigene0010759 | 56 | 65170 | 1.6 | KNGFAPQANIKQAAVHHHHPERRMVPAPADYDTTHSKETRKYLRTYGLTPPAVESFDNQAKRCLAVLESRKTPMEKYQYLSVLRNTNVNLFYRLFAHNVKELTPLIYTPTVGEACQRWSELYTQPEGLYLSWEDKGHLHSVIQNWPHDVEITVVTDGSRILGLGDLGINGMGIPVGKLSLYTACAGIHPLRTLPLCLDLGTSNEQFRNDPLYLGSRRDKVTAEEEKEFLDEMMAALTERWPSIVIQFEDFKNPFPSLERYQDTYTMFNDDIQGTGAVITGGFINAVKQSGVHPAEQRAVFLGAGSAGVGVAKQLTEFFVKEGLSEEEAKKRFWLVDSQGLVTLDRPGRPLAEHKVYFARDDNDGQQFQTLEEVIEYVKPTILIGLSTIGGAFTPEILTRMAELNDRPVIFPLSNPSSKSECTFEDAIKYTKGRCLFASGSPFPTLTYEGKELVPGQGNNMYVFPGIGLGAILSKAVNVTQNMIYASAESLSTSLTESEVADNWLYPDIRRIREVSVVVTRGVIRAAQKDGMDRELALRNLSDEDLDDYIRARMYDPFKEHEKIGDEIKELVNGDRARL |
| 253 | Unigene0014481 | 55 | 36366 | 3.6 | MVVKAGINGFGRIGRIVFRNAIQHGDVEVIAVNDPFIEPHYAAYMLKYDSTHGIFDGKIEVDGNQGLIVNGKKIRFYMEKDPAAIPWGEAGAEYIVESTGVFTTTEKAQAHIKGGAKKVVISAPSADAPMFVMGVNNTEYKSDIPVISNASCTTNCLAPLAKVIHNEFTMIEGLMTTIHSYTATQKTVDGPSGKDWRGGRTAAQNIIPSSTGAAKAVGKVIPDLNGKLTGMSMRVPTANVSVVDLTCRIEKGASYDEIIAALRKASEGELKGVLAVTDDDVVSSDLNGNINSSIVDVKAGISLNKNFVKLVSWYDNEWGYSRRVIDLLAYIAKVDGNA |
| 254 | Unigene0002277 | 55 | 61609 | 5.4 | MGVTTSQPSAAPAATAAANITCASLCSALSFTDSEYPVHPVSCQSYAAGDDITITGGQAQAGCGTSFSPQLDVCRVVLTVETSDASETYMEVWLPENGQWNGRTMNTDNGGVNGCVHYVDMDYVSSRGFAAIGDNAGHNGSSFDGSWFADDNEVIIDWVWRARHSAVVAGKEVVNQFYDQAPEYSYYIGCSAGGAQGMKSAQMFPDDFDGIIAGSAAADFNHLQAWSGRFVQLTGTSASDPKFLTQNDWITVQAAIFDQCDEKIDGVNDGILEDPTLCQFDSSVLACSNNATLSGCLTDTQVNTVREVFTELYNTEGELLYPALLYGSQVDAFRLGQLSGSVQAISRDWYRYAVNDDPAWDPLDMDQADYARADALDAYHGNASQFSGDLSGFRAAGGKLLMYHGTADPLVSSSNSQRYYLKVASELGIDNVDMDDFYRYFRISGMAHCGVGGISGAGAWMFGQNAAAAVASHNIIDYLQEWVEDANPPETLIGTKYWYDTVSMGVQFERAHCRFPYRNTFITGQDPNDVDSWRCELIDEWRECGPGALPRLCNVDGSFN |
| 255 | Unigene0013546 | 55 | 30445 | 8.7 | LAVRIPDGTWDTHMHVVDPVTFPLAPGAQYVPEAHTMEQATTFLKQLGIEKAVVVQPSIYGNDNGATLSGLHDLGTANGRAVVQFDPAVTSKEQLQAWHEAGVRGVRLNFKSVGADPSGAELEERLRTYADAVRPFGWALDLYLGLEKVPLLEQYINELGVKVILAHYGHPSNETLSTAVSAEQVPGFDALVRVLKAGNVWVKLSGNYRLSRDPHNALVESLGRAVIRTRPDRCLFATDWPHTRFEDLDVAPFLERVLDWIEAEGASVTQVLVDNAQ |
| 256 | Unigene0010572 | 54 | 10334 | 9.8 | PTISQHDHAKSPPKTSPPDHTTAKMDSAKVPVKLVKVIRVLGRTGSRGGVTQVRVEFMDDTTRSIIRNVKGPVREDDILCLLESEREARRLR |
| 256 | Unigene0010571 | 54 | 5655 | 18.4 | AGSRGGVTQVRVEFMDDTTRSIIRNVKGPVREDDILCLLESEREARRLR |
| 257 | Unigene0001867 | 54 | 29745 | 13.2 | STTAPAQAMSGVNISKRRKFVADGVFYAELNEFFQRELAEEGYSGVEVRVTPTVTDIIIRATHTQEVLGEQGRRIRELTSLITHRFRFPPDSVSLYAAKVQSRGLSAVAQCESLRYKLLNGLAVRRACYGVLRFIMESGAKGCEVVVSGKLRAARAKSMKFTDGFMIHSGQPAKDFIDSATRHVLLRQGVLGIKVKIMRASSSPNDPSGEKAGGKGLPDSVTIIEPKEEQPILAPSSQDYGAKAAQAQAYAQQQQAEQEGAPAEDGAAQQGEY |
| 258 | Unigene0001750 | 54 | 34484 | 4 | MALRLATRRFAPQLVARRGMASVVDTAKNAANAPGIVSEATSRSTAPKPASETSTIQDPTADKNAKIKTFQIYRWNPDTPEEKPKMQSYTLDLNKTGPMMLDALIRIKNEVDPTLTFRRSCREGICGSCAMNIDGVNTLACLCRIPTDTAKESRIYPLPHTYVVKDLVPDLTQFYKQYKSIKPYLQRDAPPEDGKEVRQSKEDRRKLDGLYECILCACCSTSCPSYWWNSEEYLGPAVLMQSYRWIADSRDQRAEQRKDALNNSMSLYRCHTILNCSRTCPKGLNPALAIAEIKKAMAFS |
| 259 | Unigene0004672 | 54 | 17754 | 7.3 | MAPKFDPNEEKIIHLRATGGEVGASSALAPKIGPLGLSPKKVGEDIAKATGDWKGLRVTVRLTIKNRQAAVSVVPSASSLVIKALKEPPRDRKKEKNIKHTKSIPLDEIINIAKTMRHKSMAKDLKGTVLEILGTAFSTGCQVDGRSPKAISDDIKAGEIEIPEE |
| 259 | Unigene0004673 | 54 | 17623 | 7.3 | APKFDPNEEKIIHLRATGGEVGASSALAPKIGPLGLSPKKVGEDIAKATGDWKGLRVTVRLTIKNRQAAVSVVPSASSLVIKALKEPPRDRKKEKNIKHTKSIPLDEIINIAKTMRHKSMAKDLKGTVLEILGTAFSTGCQVDGRSPKAISDDIKAGEIEIPEE |
| 260 | Unigene0013799 | 54 | 35900 | 9 | MSSSLDQLKASGTTVVCDSGDFATIGKYKPQDATTNPSLILAASKKPEYAKLIDEAVQYGKKHGKNIDEQVDATLDNLLVQFGKEILKIVPGKVSTEVDAKFSFDKQASIDKALHIIELYKEEGIDKSRVLIKLASTWEGIQAAHELQSKHGINCNLTLMFSLVQAIAAAEAGAFLISPFVGRILDWYKANTKQEYNAQNDPGVKSVQQIFNYYKKFGYNTIVMGASFRNIGEITELAGCDYLTIAPNLLEQLYNSTDSVPKKLASEEAGKLDIEKKSYLKDEPAFRFDFNEDTMAVHKLSEGISKFAADAVTLKDILKEKINA |
| 261 | Unigene0013492 | 53 | 36439 | 11 | CTEENIIVRKEWSDLTPDERREYIDACKCLHDKPSRTPKGVAPGAKTRWDDFVVAHILSSDDVHRSPKLLAYHRLYLHELEKALRDECGYTGGQPYWLWSRFYGQPIESYPIFDGSDTSLSGNGKEDTPKKGCYCITEGPLKDWRVHLGPAAGNDACTPNPQDDGLGLNDRCLERNFNKKYLGNLTYDNVTFTINNFKDIDGFGVYLESWPVGVHQLPHVFMGGTNADVPSAASEPWFPCHHAALDFIWLLWQSIDVDNRTLALGKAESYADLRKRSQAPPATGIGLDDDISISPYFDKVKVRDIMSPTGGHLCYRYE |
| 262 | Unigene0000069 | 53 | 38291 | 3.5 | YSWKTNLYGLTVDTIKSFTLVCPNGTIATVDQSRPDLFFALRGGLNRFGVVADAELYTHSQVPLIYGGTRLYDSTQVDKIVNATYEFERTNTDPKATVITSILGTTATTQFFYDGPGKPASFAVFDGIRTLLLDTVKTQSFSDFVASIPAEIRDVSNFRGTWETFSTTKTTLRFLKEVQAEAKVRLSVIFVNLVLIWCAETUUTTSCPDGHEPRSRCVYSRNVRPACSSGHFLPTLRIPASGESAGGKLUARLTPTUVLLDVAWAFSGDDEFWRRQVRQSRDALVKIAQEEGILHPISYSNYAQGTTPAQELYGSTNAARLARIRNEVDPQRIMDLTGGYVI |
| 263 | Unigene0012960 | 52 | 71806 | 3.6 | MSARPAPESVAKEAAKAIHAPRDPNTLSNYNAWRTKHTTADFNVDFDNKRLTGVVHLTLEKLADEKQIILDTSFLDIQGVDVEGDQVQWKLNKDRIEPYGSPLTITLEDKHNKTTGSEIKLSIGVNTTKDCTALQWLTPAQTSNKKHPYMFSQCQAIHARSLLPCQDTPDVKSTYTFNIRSPLPVLASGLSTGTKDYVPGKDGKSGTLLYTFHQQIPMPSYLFALASGDLASASVGPRSQVWTGPEELTAAQWEFEADTEKYIEIAEKIVYPYAWTTYNVLVLPPSFPYGGMENPVVTFATPTIVSGDRQNVDVIAHELSHSWSGNLVSNASWEHFWLNEGWTTYLERRIQADYHGGDQHRDFSAIIGWKALSDSIEQFGEDHEFTKLVIDLKGKDPDDAFSSIPYEKGFTFLYTLEKLIGKEKWDKFIPHYFTTYKQRSVDSYEFKATLLDFFASDAEASKKLEDLDWDTWFYKPGYPPKPDFDTTLADQALALASKWEALNTGSSSDFKPSKSDISNFSSLQSVVFLEKVQTFDKPLSHALVEEMGQAYQFSTSKNVEVVSRYLVVGLQAHAKSVFEPTAKLLGEVGRMKFVRPLYKQLIKNDLQLATKTFEKNRDFYHPICRGMVEKLFEK |
| 264 | Unigene0009341 | 52 | 35304 | 3.1 | MPGEYSKSWLQWEEASGGRAILHGSPAEIKGMYDALIQALLPLLPAPSENVAVQEGDVDGIKYRVYTPKSSKGGLPIGVNTHGGGFMTGDLNSDALLCMAISEYTNTAIVDVDYRLTPDFKWPVQLEDSLKVYKWAHDNASSFGGDATKMFTMGGSAGGALALQIANQIVKDSKYKSSLKGVAAQVPCTTHWDNVPEKYKSKYNSYTENAKGTPIIDKESMDTFYTHVNADPKDPDVFTILATDNHANYPPVYFTACEFDPLRDDAYIMKDALDAAGVKTKLDYYEGMPHYFWIFPPVPESQTYLQNLVAGVEWLKGQM |
| 265 | Unigene0010240 | 52 | 25246 | 5.6 | RMHSLNSIALLSVYAAKFALGQQQPIAESHLTINGIPFSTRAHWMRRANKALADLVSPCPFGAFGTAIVNHTAGDGLGELVCIGANSIGSTGNPTLHGEIVAINNCTTVLTNPEGPYRLSASEAQRAFASLSLYTNAESCPMCASAIRWAGFKEYIYGTSIDSLIKKGWGQIRIGSMEVFEQSFDLGSSTRLIGEVLTNETDPYFSWQYDPNSACPAGCTRSEGQCARTDQ |
| 266 | Unigene0012918 | 52 | 10282 | 9.5 | VSRAAGEVTCSASYKFFFEDFSIRGKNVPDSKLGDNGSNLRQQLTGCGALTKWKFEWTPNDGNYAWHATGRLPIGTKACVGRALNTALGAGSGGC |
| 267 | Unigene0010498 | 52 | 26392 | 3.7 | HANFAQYPSLRNKTVLITGGAEGIGASATSQFSHQGSQVLILDISESSAIKLIETLKSQGASPLPAFYQCDVSDLKALKSTCDEILKKYGTVDILVNNAASAGGAARAGTFEVTEESWQFGVDVNLRHQFFLTQYLVPAMKSSGRGGSIINMGSITWRIPATGLPVYTACKAAILGLTRTHAKEFGKDGIRVNSIMPGSIATERQRREVLTEEYERLTLEAQSLKRVLEPDEVGRCILWLGS |
| 268 | Unigene0001705 | 51 | 110211 | 1.7 | RGDFQGGQGRGGFQGDRGRGDFRGGRGGPPGRGQSSQVPFRGGRGGGGGSGPREFPGIYLESAPVAAPDASITAAENALALQTKGKIIDGFPGRPGFGTKGKPIVLRTNYFKITTAYEAKQPEVPLYRYAVNIRETSVSKPKLRRLFNEIFKHPAFSQVKWATDYATILVTTDKLDLEKMQKGEEIKVTLPSENGASPQASQGEQVPDFVKQARDRNTFHFKIRYMDSFSLRQMIDFLESTSSGALYAGRVDLIQLLNIIVAKAPNEMANVRGVGGNKYYPYNGHPGLEYKDLGEHLEALRGYFSSVRPAISRLLVNINVTSGAFFKAQHLVQVFFNTRSSAEQLEAFLRMLKVEARYVKDGQQKPFMTKTKTIVGFAKSTTTIRVKRFGNANEVKFSYVDRSIPNARAREVTVAEYFKQQHGITLRNANLPVLNVGTRADPQYLPVELCWITPGQAYRRLLSGNQTSEMLRFAARAPNLNAMSIAGVAGNPGNGLRLLRFANPNGNPQADSVEPFGFRVGTEMISVPGRILPSPTVKYGGKDASTSNGAWNLRGAKFDKPGKFNRWQVLVINRQGNNGHAMRGPPEATIKALEQALKTYNIQMGARGPTLQITLDALNMTNRPANDKMLKSAFEKAEGSKVDMLFIILPEADRWLYARIKYFGDVEHGIGTICSVGSKLENDKGQQMYFGNLALKFNLKGGGVSHSVVNTVSPPIDNNTMLVGIDVTHPSPGSVEGAPSIACVVASVDSRMFQWPGSIRTQTGRQEMVGGADANSVDHLEEMFNERLNLWVKRNQKLPTQIVVYRDGVSEGQYHQVLDIELPSFEKVFEKRYGDKKKWPKIAIIIVGKRHHTRFYPTRKEDADQRSWNPQPGTVVDRGIVGKIVREFYLQAHQGLQGTARPAHYVVIKDDISFSADALEQFTHHLCYLFNRATKAVSICPPAYYADLLCERGRSYLFSKLAENNASDASVSDGGDDEWTGGVHARLQETTWYV |
| 269 | Unigene0010859 | 51 | 142275 | 1.6 | IAMARATVWRTLATAVLLWTPSAVAASASEPPWTVRHEAGRCALRGQCGKQSLFGKELPCPDNGPAEDPSDEVRDKLVSLCGDGWRHGKVCCQEDQLDVLKSNLDTANSIISSCGACKKNFFDLFCTFTCSPDQSLFVNVTAIAPKGDKFLVTELDQLVSEDFGSGLYNSCSDVKFGATGGRAMDFIGGGAKNYTQMLKFLGDKKPFVGSPFQINFPRPNEQFEKMHPILDDSTPCNTTDERYRCACVDCAAACPALPEVTETKQCHVGLLPCLSFAVVLIYSVFVALLVLAVFGHAAAAKRRRSKNERLQLLQDASPSDDEDEGDMVHGAASLDRPTKQYFVNTYCDRIFSNLGRACAKFPGITIGTCVIVVGLLCIGWVRFDVETDPVKLWVAPDSDAAVEKRFFDENFGPFFRAEQAFLVNDTLPSGPGPVLSYETLKWWEDVERRVRAQKSLQGGHTLKDVCYRPTGDACVLQSVTGWFSGSSIERDSWQDQILKCADSPGDVDCLPEFQLPLPPERLLGGYNRSSEPATSASAIVTTWVVKNYNSGDPGLAKAEDWEQSTNRLFQDIQSEAKERGLRLSFNTEISLEQELNKNTNTDAKIVIISYIVMFIYASLALGSTTVTAGMILRNPVAALVQSKFMLGIVGIAIVLMSVAASVGLFAAAGVKATLIIAEVIPFLVLAVGVDNIFLIVHEFERVNISHADEPVSERIARALGRMGPSILLSASTETVAFALGSAVGMPAVRNFAAYAAGAVFINALLQVTMFVSVLALNQQRVEEGRLDCVPCVKIRESHLDHIPGGFGGAPFSGADEEGWLSRFIRKQYAPALMGNKTRVAIITVFLGLFAAGIALLPEVPLGLDQRIALPSDSYLIDYFNDLDSYFNQGAPVYFVVKDLNVTARPHQQELCARYTTCNEFSLANVLEQERKRPDVSYIADATASWIDDFIQWLNPDAGECCVDGSKACFADRQPPWNNALRGMPEGKEFVQYAKRWLNSPTGQECPYAGQAAYGDAVVIDEKRMTVPASHFRTAHTPLKSQEDFINAYASARRIAKDISEHNGIEVFPYSKFYIFFDQYATIVHHSTALVGTALAFILVITTVLLGSVATAVVVTLTVVMIVVDIVGTMALAGVSLNAVSLVNIIICVGIGVEFCAHIARAFTIPSASIMERAHSRFRGKEARAWAALVNVGGSVFSGITITKLLGVFVLAFTRSKIFEIYYFRVWLSLVIWAALHALVFLPVALSLFGGKGYIDPDAFGGLEQDLASRRYRALLPDEEYDSDDY |
| 270 | Unigene0004895 | 51 | 14183 | 21.5 | MDFLKNAAGSLGGGNNNNNNQQQQGQQGQQGGNPEQQQQGSSSGGGGGFLDGIMNKAHGAAGGGPESEKNEDYLDKGVDYVQEKFLGQGAQNNESAVEQAKDEQISDFIRGQYKSKTGGDMPIKDKPTALDRNVD |
| 271 | Unigene0011190 | 51 | 41966 | 4 | MTPKVVATINSTGRQAASFIRAAAAVGWHVRAQIRSCDGLVAEELAELPNVELIEGDLTGPARTSILNRLFTGAKIAFINTTHWGDEVAIGKACADAAKKAGIAHYVYSSMPDHSAYGEDWRALPMWATKFAIENYVRQIGIPATFVYTGIYNNNFTSLPYPLFQMELQDDGSFVWQAPFHPDDPLPWLDAEHDVGPALLQIFKMGANHWKGQRVTLAFEKLTPLQACARFSRGVGRPVNYVHADIKIAVNIPSGYREQLEILQETLGYKRAPYFGPDLEYPKEGRSIWEGYRGIEEYAREVFPIEEYANGLRWMEEDGMSVTGVSTPGESNGDPIDFPRSRPMTPANAVTPLHVSGAYTPKSHPEGLHRGFFVGSC |
| 272 | Unigene0012619 | 51 | 38949 | 6.2 | MVDAVKSALGMGKTVPDFDDLPKVEGMPQGCAWGVWDKFRKDGKKDIYGTLNLLTPAVIKAAYAEAKEGVHVSLNWPIGAIKTPGFGRKGLVHKTFSFVDTPLAAHGYDDEIEFNTQCSSQWDSLMHFHHQPSQSGYNGIKTTPEELVQTHGNEDKEKNIATLNHWHDRGGMVARGVFIDFKKYADETGRAFNPFTDDKITIEDIETIAKKQGVEFKEGDVIIIRSGFTEGLTDKSGEEQEKLMGSHRVCGVVGTPEAAKWFWNKHFSAVAGDMIAFEHIPSVVDGQEVPVSGLVLHQYFLSLFGMSIGELWDLKALSETCAKLNRYSFLLTSVPLNVPGAIGSPPNALAIF |
| 273 | Unigene0000451 | 50 | 58498 | 3.2 | MGDSLVHQEYGRTRVQWLASLNTEYHPPKQFRRTSIICTIGPKTNSPEKINMLRTAGLQVVRMNFSHGSYEYHQTVIDNARKAEATQEGRPVAIALDTKGPEIRTGNTPNDEDIPISAGSEINITTDDKYATASDNKNMYVDYKNITKVIEKGRTIFVDDGVLAFEVLEVTDDKNLKCRAVNNGKISSKKGVNLPKTDVDLPALSDKDKADLKFGVKNNVDMVFASFIRRAEDIAAIREVLGEEGKDIQIIAKIENQQGVNNFDEILKVTDGVMVARGDLGIEIPPAQVFIAQKMMITKCNIAGKPVICATQMLESMTYNPRPTRAEVSDVGNAVLDGADCVMLSGETAKGNYPREAVTMMHETCLLAEIAIPYLNAFDELKQLAARPVPTTENCAMAAVSASLEQNAGAILVLTTSGNTARLISKYRPVCPIIMVTRNARASRYSHLYRGVYPFHYDVEKPDFKTTPWQEDVDARLKWGINNAIKLGVLNKGDAVICVQGWRGGMGHTNTLRVVPAQEDLGLDQNA |
| 274 | Unigene0013780 | 50 | 9610 | 12 | ISSLLLALLAIFAAAAVEPKHQVIISYPKDTPASIMEDAKKAIIEAGGFITHEYHLITAIAATVPAKILDTIQAAGAGHNVLIEEDGEVSIN |
| 275 | Unigene0007369 | 50 | 35577 | 4 | ATPLLKRDYPPVGECTGQCEGRLHDPAVIYRENTSTYYRFATNDGINIATAPSISGPWEFQGAALPGGSSIDLPGNRDLWAPDVFNFGGTYYLYYSVSEIGSMNSDIGVATSQTLDAGDWTDRGSIGIPASASYNRIDANLYAYDANRLVLNFGSFWENIFQLPIQNLPLQVEAAGPLQHLARNTTTRPQGLATGAQEGPYMFFWQGWHYLFFSAGNCCDAAGSLPPAGEEYHILVCRSRDQSGGFVDQDGRDCLTQSGGTLVLGSHGDVYAPGGQGVMWDARVGSVVMYYHYVRPSVSYDYDNFFFGWSKLDFGSGWPVVVA |
| 276 | Unigene0002925 | 50 | 46471 | 2.1 | MIAKSALQRAAAAPARAAFQQQSIRAASAWSQVPQGPPDAILGITEAFKKDSNKQKINLGVGAYRDDKGKPYVLPSVKTAEQKVVQQNLDKEYAGITGVPDFTKAAALLAYGPDSAAIKEGRVVITQSISGTGALRIGGEFLARHYPGAKAIYIPTPSWANHKAVFLDSGLEVKQYKYYNKDTIGLDFDGMVADIKAMPKGSIVLLHACAHNPTGVDPTEEQWKAISDAVKEGEHFPFFDMAYQGFASGDTDKDAFALRYFIQQGHQPCLAQSFAKNMGLYGERVGAFSIVTSSPEEKTRVDSQVKILVRPLYSNPPVHGARIASTILNDPALNKQWLGEVKEMADRIIKMRALLKGNLEKLGSKHNWDHITSQIGMFAYTGLKPEQMTKLAEEHSVYATKDGRISVAGITSDNVGRLAEAIYKIT |
| 277 | Unigene0002278 | 50 | 127836 | 1.5 | MEKLKKLLIANRGEIAVRIIRTAKQLGIKTVSVYTSADAASLHVSDADEAVLLPTLQSYTDGDELLRIAKEKGVDAIIPGYGFLSENAAFAKAVSDAGLAWAGPREDAIKAFGIKHTARDLAKQSGVPIVPGTEGLVESEEDAVKQSDTLGYPVMLKITAGGGGSGLITCQNAEQVKDGFKKAQSRGQALFKDSGVFIEKYYPEAHHIEVQVFGNGLGHAIHFGERECSIQRRHQKVIEECPSPFVQKHSGLREKICSAAVSLAQSVKYGSAGTVEFLVDDKTADFFFLEMNTRLQVEHPITEACYDVDLVELMLKQADAQLSGQGGLSADYLDSLQKPGPFGAAVEARLYAENVLRDYAPSPGLLTEVYWAERQNARIDTWVRTGLRISPNYDPLIAKVINHAKTRDAALKGMSMLLEQSVVSGPPNNLDFLGAIIKDETFQSGRTITSFLQNFDYKPNVIDVVTAGAYTLVQDLEGRPSVGKGIPRSGAMDPIALAVANTLVGNERGQEGLEITLSGPELKFHGSAVIAVTGAPIEAALDGDSIPMWTRKHIKPGQTLRIGKTTAGGCRSYLAVYGGFTSVADYFGSKSTSPIVAIGGYQGRQLAPGDQLSLVKEIPSDLHSHPSVPESLRPDYSSPWTINALPGPHEEGYLTDEDIEMLYNTEWKVSHNASRSAIRLIGPVPKWARADGGEGGSHPSNLVEYGYSCGSLNWTGDEGCIFALDCPNFGGFASSATAIRADYWKLGQLKAGDSLKYQRVSLEEALSLRTNVEDYIEGISKGVQTNDFNNVKPLASDFKASGNYSKAVVWERPASGAQPQVRYRQAGDDYLLIEYGNEQFDLNYRCRVTALEKAINDSSSAPSWLTKDLIITVGCCTSLNIHYDGSEMDRAQLIAHLQTLEDQIGDLSKTKVPNRRFKLPLSFESKEQTEATERYMSNQRPHAPYLPDNLDFVAKNNAFTADQLKHNMLTGELMAVVVGFYCGNTVSLPVDPRMRMSAPKANPSRVFTPEGTFGWGGSCASIYPVDSPGGYQMLGRTIPCFDYHGFKSGFTPTRPWLFQDFDILTFHLVTESELNAQLELFRSGQYVFTYEDSEFDMAEHNKLLEATRSEVEEIRAKQRKVQEEMITAENESLKRWREEKEKRKPDLSTIESLLGQEGV |
| 278 | Unigene0002310 | 50 | 50670 | 3.9 | MPPKDRLAQLSRHFSHSPAHDIPLNTPFSIQSKIVDDFASTGSGETFNKPEQRRDLSQSAVSQEEEGKRSKPAAATMSNQPPHPTLLIPGPIEFDDAVLQSMSHYSESHVGQPFVNCFGDVLSMLRKLFISTDPNAQPFVISGSGTLGWDQVAANLTEPGEDALVLHTGYFADSFADCFETYGVKATQLKAPIGDRPQLDEIEKALKEKKYKVITVTHTDTSTGVLSEIEPLSKLVKKVSPETLLIVDGVCSVGCEEIRFDEWGVDAVITASQKAIGCPAGLSIVFASGRAIQAFKNRKTPPNSYFGSWKNWLPIMQNYEAKKPSYFATPSPQLIHALHTSLTQILSIPLEQRFQRHKEVSQKIKKAVADLGLKQLASKPENQANGMTAIYLPNDLKPPEILPNLLKKGVIFAGGLHKEIATKYIRFGHMGVSIMDPQRDDVDRALQSLKDALGEVGYK |
| 279 | Unigene0003531 | 49 | 27424 | 8.4 | MVRYAATEIESAKSARARGSYLRVSFKNTRETAQAINGWKLQRALTYLDNVQNKAEAVPMRRYAGSTGRTAQGTUAHLMVMWLPCARMHRWNTFIQTNTRTHIERCCCGRNFGTSYUETISCSGKQFGVSKARWPVKSAEFLSSLLKNAEANADTKGLDTSNLIVKHIQVNQAPKQRRRTYRAHGRINPYMSNPCHIELILTEGEEVVQKSPETVERKSLHLNSRQRAQRQRKAITAA |
| 279 | Unigene0003530 | 49 | 21136 | 10.7 | MVRYAATEIESAKSARARGSYLRVSFKNTRETAQAINGWKLQRALTYLDNVQNKAEAVPMRRYAGSTGRTAQGKQFGVSKARWPVKSAEFLSSLLKNAEANADTKGLDTSNLIVKHIQVNQAPKQRRRTYRAHGRINPYMSNPCHIELILTEGEEVVQKSPETVERKSLHLNSRQRAQRQRKAITAA |
| 280 | Unigene0006177 | 49 | 32750 | 7.7 | ALTALASACAPELLKRDGWRGAHIAKRAEPGARAWSFELAEAWNQLNPEWSTCYSGRRQSPINLRSKWGYYTQEPPTFHYPKNLTGKIDNWDFGPQFSIEVDDDNKTAAYIEFDHDGDPEIAYFRQWHVHTAAEHTIDGYRPKGEIHFVHYDDAGVPRSVIGFFFDRKSKYDDYNFNFTGPAIDATGSAFYKQLDRLELPDIFSNNTLEAVELDLNLLVEEAKGYKKFWTYKGSLTTPPCSEGIRWFISPEIIWLSDNQTESLLRASTFGDRPVNSVWDHDINV |
| 280 | Unigene0000806 | 49 | 32750 | 7.7 | ALTALASACAPELLKRDGWRGAHIAKRAEPGARAWSFELAEAWNQLNPEWSTCYSGRRQSPINLRSKWGYYTQEPPTFHYPKNLTGKIDNWDFGPQFSIEVDDDNKTAAYIEFDHDGDPEIAYFRQWHVHTAAEHTIDGYRPKGEIHFVHYDDAGVPRSVIGFFFDRKSKYDDYNFNFTGPAIDATGSAFYKQLDRLELPDIFSNNTLEAVELDLNLLVEEAKGYKKFWTYKGSLTTPPCSEGIRWFISPEIIWLSDNQTESLLRASTFGDRPVNSVWDHDINV |
| 281 | Unigene0004949 | 49 | 29973 | 3.5 | DCYSKYRECQGGPDANQSFCASQFAGCANYNPFTGEEQAPYFANLQASCKAATTSAPGGYPTAPPAYGASSSPPAPTYPAGKPDAKTCECYAKLGQCQTAPSANQASCAAEFASCSGNNPYTGDDRATAYFDNLKKNCPTNSAPAPSSTPTAPGKPDAKTCECYAKLGQCQTAPSANQASCAAEFASCSGNNPYTGDDRATAYFDNLKKNCPAGGPTTPSSAPQPSGTPTGPTGKPDAKVCACYDQANKCRVGPGANQSFCSSQFASCAGYNPYTGDTAATT |
| 282 | Unigene0001280 | 49 | 18536 | 20.5 | MATELTVQSERAFQKQPHIFQNSKSKAKSSRVGKGGRRWYKDVGLGFRTPKTAIEGQYIDKKCPFTGLVSIRGRILTGTVVSAKMHRTIIIRREYLHFIPKYSRYEKRHKNLAAHVSPAFRVSEGDQVTVGQCRPLSKTVRFNVLRVLPRTGKQVKAFNKF |
| 283 | Unigene0008689 | 48 | 27666 | 10.2 | MLTPAFTFAVLAAARLAQAQYASTTTFAFAGNALPTGLANYYSRVNDTKTVGAPYDHEFEPSMSYVEGGFLNLLVPGGQQNDQVIWSAEVQTTFTVSAARVDTWAILTETHGVCNGIFSYDGDTSISGGIHESDIEWLSDPNSLAFTRPGIARALYYTNQNTKAVGSPGTSGPGVPPADATSKVHKYSIEWRNGLTSFFVDDVPQYTFDTNVPTTTKAVWVWNNWANGDPNWSVGPPATDAVFKIQKIVMAYNPGS |
| 284 | Unigene0012189 | 48 | 40301 | 5.9 | MAAENPHFEPNPKYDHYDFPTTAPENKPGHPGHTTKEQDAQVEQLRMMLESEGYTERLDTLTLLRFLRARKFDVQLTKEMFVKSEEWRKTFGGGVDNLVRTFEYKEKEQLLPYYPQYYHKTDKDGRPVYIEKYGNIDLEAMRKITTDERMLENLVVEYEKVADPRLPACSRKSGQLLETCCTIMDFKGVGLMKMNQVYGYVQRASAISQDYYPERLGKLYLINTPWGFSSVFGVVKRFLDPVTVAKIHVLGGGYQKELLAQVPAENLPKEFGGTCECPGGCQLSDAGPWHDSQFAKPAKWETEKTETKPAANTIPATETHTGEGAPAAAPPAGTTAAPAPAGDRVPGTEAHTGAPAPQ |
| 285 | Unigene0014442 | 48 | 9595 | 11.6 | MENERGELVDLYVPRKCSATNRIIKAKDHASVQISVAKVDENGRATGENQTYALCGFVRAMGESDDAINRLAQRDGFLKGVWSASR |
| 286 | Unigene0009861 | 48 | 60910 | 5.2 | MKLTGLLLPPFLLSQLCSAVPTAQSEPCDDADGNKLGAVASESNECSEVGAQILKKGGNAADSMVATVLCVGVIGMYHSGIGGGGFMLVRSKNGSYEFIDFRETAPAAAFQDMYNNNTDASLYGGLASGVPGELRGLQHLHENYGKLPWKDLVLPSVKIARDGFVVGKDLVKYMDSAVASAKDVVGNFLVDDPNWAIDFAPNGTRLGLGDTVTRKRYAATLETIAEHGPDAFYSGPIAQTWINHTRATGGNMTLEDLKNYTVAIRKPASITYRDFNIHSCSAPSSGTIAMSIMKIIEGYNNIGWASAVNESTHIFNEALRFGYGQRANLGDPLFVEGLDQYQEEILSEAVVSEVQAKISPLQTFNTSYYDPSGLESLETPGTAAVTTADADGLAIALTSTINLLFGSKVLIPETGIIANNEMNDFSVPGQSNAFGFIASPANYVAPGKRPLSSITPTIVEHSNGTLYFVTAAAGGSRILTATVQSLWNVLDKNATSAESLAAPRLHDQLVPNQVSFEYAYDNSTVAYLASLGSNVTWVAPGGSSAQCLRRLSNGTFEAAGEPRQFDSGGVVV |
| 286 | Unigene0009862 | 48 | 52768 | 6.1 | SGIGGGGFMLVRSKNGSYEFIDFRETAPAAAFQDMYNNNTDASLYGGLASGVPGELRGLQHLHENYGKLPWKDLVLPSVKIARDGFVVGKDLVKYMDSAVASAKDVVGNFLVDDPNWAIDFAPNGTRLGLGDTVTRKRYAATLETIAEHGPDAFYSGPIAQTWINHTRATGGNMTLEDLKNYTVAIRKPASITYRDFNIHSCSAPSSGTIAMSIMKIIEGYNNIGWASAVNESTHIFNEALRFGYGQRANLGDPLFVEGLDQYQEEILSEAVVSEVQAKISPLQTFNTSYYDPSGLESLETPGTAAVTTADADGLAIALTSTINLLFGSKVLIPETGIIANNEMNDFSVPGQSNAFGFIASPANYVAPGKRPLSSITPTIVEHSNGTLYFVTAAAGGSRILTATVQSLWNVLDKNATSAESLAAPRLHDQLVPNQVSFEYAYDNSTVAYLASLGSNVTWVAPGGSSAQCLRRLSNGTFEAAGEPRQFDSGGVVV |
| 287 | Unigene0001700 | 48 | 8605 | 23.7 | MLASIFLLSLATIASADSCVERGYGPPGCPSHTERSYYWRCLPSARDPANKDGPPGNQCGFVSRDTDGTKYYCCQN |
| 288 | Unigene0014571 | 48 | 10647 | 8.2 | MKVFLCIFTALWALISTVSAAPQPIPDPFLGFNAMGWSAKILAYCNRGWGGDGGCEAKGFYTFCCKQDYGGEFKHSKFKAINAGLGGCADGGTILCA |
| 289 | Unigene0014340 | 48 | 40157 | 3.2 | MASRPTVTIIGADGAPSQDTHPLPNVFKAPIRPDIVQTVHTGMAKNKRQPYAVSEKAGHQTSAESWGTGRAVARIPRVSGGGTHRAGQAAFGNMCRSGRMFAPTKVWRKWHQKINLNQKRFATASAIAASSSAALLLARGHRVNEVPEVPLVVSSTAFSNAAVKKTSAAVALLKSVGAGPDLEHVKNSRKIRAGKGKLRNRRHTQRRGPLVIYEPEKDGKELVRAFRNIPGVETCTVYALNLLQLAPGGHLGRFVIWTSAAFAALDSVYGSTTEPSALKRDFLLPQNTVAQADIAKIINSSEIQSVLRPVKGGAITKRTVVQKKNPLKNKQVLLRLNPYAKAYSSEGLGHVKSTDKPIVKEKAFEATLHEN |
| 290 | Unigene0009461 | 47 | 56807 | 1.6 | MVGLGPRRQPSRRGSMADMPKDLLAEIKRLEEIFTVDTAKLKAITDHFVSELEKGLSKEGGSIPMNPTWVMGFPTGHETGTFLALDMGGTNLRVCEINLPEEKGEFDIIQSKYRMPEELKTGNADELWGYIADCLQQFIEYHHEGEKLDRIPLGFTFSYPATQDYIDHGVLQRWTKGFDIDGVEGKDVVPPFEKALEERGVPIKLTALINDTTGTLIASAYTDSEMRIGCIFGTGCNAAYMEHAGEIPKLEHMKLDPKQEIAINCEWGAFDNEHKVLPRTPYDVIIDKDSPRPGQQAFEKMIAGLYLGELFRLVLVDLHEQPHVQLFQGQDISSLKKPYSLDASFLSDIENDPYENLQETYDIFYDKMKINCQKPELELIRRLAELIGTRSARLSACGVAAISKKKGYKECHVGADGSVFNKYPHFKARGAQALKEILDWDKGRNGKSLGKGHDPIEILPAEDGSGVGAALIAALTLKRVQEGNHAGIRDPKSMLAGTAASSSKTNQV |
| 290 | Unigene0009460 | 47 | 56807 | 1.6 | MVGLGPRRQPSRRGSMADMPKDLLAEIKRLEEIFTVDTAKLKAITDHFVSELEKGLSKEGGSIPMNPTWVMGFPTGHETGTFLALDMGGTNLRVCEINLPEEKGEFDIIQSKYRMPEELKTGNADELWGYIADCLQQFIEYHHEGEKLDRIPLGFTFSYPATQDYIDHGVLQRWTKGFDIDGVEGKDVVPPFEKALEERGVPIKLTALINDTTGTLIASAYTDSEMRIGCIFGTGCNAAYMEHAGEIPKLEHMKLDPKQEIAINCEWGAFDNEHKVLPRTPYDVIIDKDSPRPGQQAFEKMIAGLYLGELFRLVLVDLHEQPHVQLFQGQDISSLKKPYSLDASFLSDIENDPYENLQETYDIFYDKMKINCQKPELELIRRLAELIGTRSARLSACGVAAISKKKGYKECHVGADGSVFNKYPHFKARGAQALKEILDWDKGRNGKSLGKGHDPIEILPAEDGSGVGAALIAALTLKRVQEGNHAGIRDPKSMLAGTAASSSKTNQV |
| 291 | Unigene0013494 | 47 | 75842 | 1.3 | ASSKAAPAEPVDPFKTGWLKGVYNEKPVPPSEVQTRFPPEPNGYLHIGHAKAITVNFGFAKSYGGKCNLRFDDTNPAKEEEQFFTSIKDMVQWLGFEPARITHSSDDFDRLYELAEELIRRGKAYICFCSAAAIQAGRGGKEHGPRSVCEHWSKGVEQNLEEFRGMRDGKYEAGQALLRMKQYLGTKPEEYEAAEGDSAEVKKEKDKLRKLANNPALWDVAAYRIKKENYHHRTGNKWRIYPTYEFTHCLCDSFEGITHSLCTVEFETLRPAYDWLCEALDFKLPGSDEKGPMQREYGRLNVEGTILSKRRIAMLVNGTTVGGDAPDPVDEAADAVDEIKLEEEGDEEPETIGEASKAVTAGTGRKIPPVVRDWNDPRLFTLVALRRRGVPPGALKKFVFDLGVTKSNANTMTHALDATMRTYLERTVPRLMLVLDPIKVTFTNLPDDYVEERVVPFDPKDKEKGDHKVPFTKTIYIDRDDFREVDDPDFFRLSPGQSVGLLNAEFPIKIVSFSKNAEGKVDEIKAEYGKDVPAGKARIHWVGESKAHDSPIKAECRIFNALFKTTKPNELDWKNGGYYDDVNPESEVIYKNALVEVGFREIQQRAPWPKTDGETSVNADKSSVRFQGLRTAFFAEDQDSTPEHVVLNRIVSLKEDTSKKELGAGAKKEG |
| 292 | Unigene0002372 | 47 | 14815 | 10.8 | MVKTSVLNDALNAINNAEKTGKRQVMIRPSSKVIVKFLQVMQKHGYIGEFEEVDDHRNGKIVIQLNGRLNKTGVISPRYNVQLTDLEKWVVRLLPSRQFGYIVLTTSAGIMDHEEARRKHVAGKIIGFFY |
| 293 | Unigene0014272 | 47 | 15144 | 13 | ILILFAGLAAADVLRIPPKPRPKPHGNQAEIFPDADTRPGLDWFPDCQCNIDGAYSTWLTNAVCLRDSYNYHVSGRCWNNPGIWMKAPAWQNDCVEAARAGFTDYRLVGFFHKHWEEYVHKVESGVTGVCF |
| 294 | Unigene0006575 | 47 | 20045 | 8.4 | ENRAFEKGRQPANTRIGAKRVHLVRTRGGNRKFRGLRLDSGNFSWGSEGISRKVRVIAVAYHPSNNELVRTNTLTKAAIVQVDAAPFRQWYEAHYGQPLGRRRQAKAETEEVKKSKSVEDKQAARHKAAGKVEAPIERQFEAGRLYAVVASRPGQSGRVDGYILEGEELAFYQRAIRK |
| 294 | Unigene0006576 | 47 | 22592 | 7.5 | MGISRDSRHKRSASGAKRAYYRKKRAFEKGRQPANTRIGAKRVHLVRTRGGNRKFRGLRLDSGNFSWGSEGISRKVRVIAVAYHPSNNELVRTNTLTKAAIVQVDAAPFRQWYEAHYGQPLGRRRQAKAETEEVKKSKSVEDKQAARHKAAGKVEAPIERQFEAGRLYAVVASRPGQSGRVDGYILEGEELAFYQRAIRK |
| 295 | Unigene0001963 | 47 | 15279 | 19.3 | MTKINSNLHSSRSKSRKAHFQAPSSVRRVIMSAPLSKELREKHNVRAIPITKGDEVIIKRGSNKGREGKVTSVYRLKYVIHIERVQREKSNGQSVPLGIAPSKVEITKLKLDKDREKIIERIAAGRVANEKKRKA |
| 296 | Unigene0003586 | 47 | 10969 | 7.1 | MAENNGTPPADQPDAQQPASEHLNIKVTDGNNEVFFKIKRSTQLKKLMDAFCDRQGKSPQSVRFLFDGQRVNATDTPDVLDMQDGDSLEVHQEQIGGC |
| 297 | Unigene0002253 | 46 | 58357 | 2.2 | MRTFALASAAAAIVGSAAAADLPSIVIKGSKFFYENNGTQFFMRGVAYQQDYSGGGSTGNTSDTQTYKDPLADADACKRDIPYLQELYTNTIRVYAINPDSNHDECMSMLADAGIYVVADLSAPVDGSSINRNSPAWNDALYERYTSVVDAMAGYSNVLGFFAGNEVSNQPNNTDASAFVKAAVRDTKAYIKSQNYRTIGVGYATNDDADIREDMEKYFNCGDQEAAIDFWGYNIYSWCGESSYSKSGYDVRTKEFSSYSVPVFFAEYGCNEVRPRPFTEVGALYGEKMTPVWSGGIVYMYFQEANDYGLVTVDGDNVKTNDDFDNLKSELAKIDPTGVNSASYSPTNSPAACPTESSDWNAESSPLPPSPNKELCTCMYNSLGCVVTGQTDVDDYGDLFGTVCGYGDDVCAGITANASTGSYGAYSMCNSTEQLAFAFNQYYLSQNSAADACDFNGAAVTKSATKASGACQTLISEAGTAGTGTVTSSPTANSNQQAQQSGKGSSSSSGAASPLGVPTAGASLLPVFGIMILGMISGAGMILL |
| 298 | Unigene0001295 | 46 | 54893 | 2.5 | MVPRPIANPDLPKPPSGKYPAKAHARRVAKYIAEHGGPSSGIIYLEGQSTRMTEDDDQAGHFRQRRHFYYLTGCDLPDCFFAYDVASDTSTLWIPPVDPEYVMWAGMPLLPKEALERYDIDHVSTTDDLKSGKSLVEMLSKQKPVILAIEDRADLAIFDTPAIKNFQPEINLASLREAIEECRVVKDEHEIAMIRHANIVSSYAHEQVLSSVPRASNERELNAVFVMHCHANGCKEMAYGCICASGTAGSTLHYVHNDQPLEGKDNILLDAGAEYNCYCADITRTFPITKDGRFTKESKEIYDLVLLMQSEAFKLIRPGAMWEDCHMKAHTTGALGLQKLGIFNKDLTLEQIMSSHIMTRFFPHGLGHYLGMDTHDTGGHANYDDPDPYFAYLRKRGPLPVGAVITNEPGIYFREFPLRQELKDGKWDGIVVQEVLERYWRVGGVRIEDDVVITEDGYENLTTVSSDWQTVEAMVQRGLANGAL |
| 299 | Unigene0001458 | 46 | 49791 | 2.1 | MASSQQQPLYSEYSFNPLEHLAGIAPYFEPNDPAREPSPPQGCEVEKVAYLVRHAAINANDFDYETYLEPFTDKLKNTTVDWSKIPELSFLSTWTPPKLEEQELVTRSGKLEASQLGVQMSFRYPKLRLPKRVWASTAERTVISAKGLIRGLETDDNQINLVQIYEGKESGADSLTPYKACPAYSSSRGSEQSQTYAKKYTAPILARLKAYAPNFNWTVNDVIGMQEWCGYDTVVRGSSPFCSTTLFRPDEWLQFEYTQDLMYHHNTGYGNPISGSLGYGWVNATQQLLSSDDDDDQDIYVSFTHRELPPTVLVTLGLFNNSDSTGANDINATMPLDKVNYHRQWVSSYILPFLTNIAVERMNCTGSYGYQNQTDPTYYRVLVNRSPQTIPDCHDGPQESCSAAGFKSFVEERGQLFGDFAGACGVEYDNSTSVMSFYS |
| 300 | Unigene0009254 | 46 | 15468 | 6.7 | MSESQVTLRTRKFIRNPLLGRKQMVVDVLHPSRPNVSKDELREKLAGLYKMNKDQVSVFGFRTQFGGGKSTGFALLYDSAEAMKKFEPHYRLVRYGQATKIEKASRQQRKQRKNRSKEFRGTAKTKGPAKDKKK |
| 300 | Unigene0009255 | 46 | 12572 | 8.3 | MSESQVTLRTRKFIRNPLLGRKQMVVDVLHPSRPNVSKDELREKLAGLYKMNKDQVSVFGFRTQFGGGKSTGFALLYDSAEAMKKFEPHYRLVRYGQATKIEKASRQQR |
| 301 | Unigene0004679 | 46 | 105364 | 0.9 | MRLFRYALAVAHLLGLPTSAQRVISLAGDDWTLSNPLLNISLPAKLPSQATLDLYDNQVIGDPLFGRNSYALRFIVWQNWTYISAPISGLSSNASSTWLLFNGLDTFTSISFCGQHVASTNNQFRQYWFDVSKFLTNCTEENRILDINFGSAANIADEIANQPGQETWADGIQQLYQFPNRWFVRKEQNDFGWDWSAGFVPAGPWQPAWIVQLEDREVHIRNSLVDIYRQGQLNNLPPDQNQPWVVNASLDYFGNVPDSASLKYKLTSIDNATTIASGDLTGVNVTDTAVTGSTIVPQEDVELWWPVGMGPQTLYYITIELVSKTGQTLASVSKRVGFRTIVLNGNPVSEAEMAKGIAPGNNWHFEINAQPFFAKGSNFIPPDEFWPRVTPQRISDLFDTAIAGNQNMLRIWSSGAYSPDFMYDLADEKGLLLWSEFEFGCALYPADPEFLENVRAEAEYQVRRVNHHPSLTFWAGGNELENLELPNANRSSPEEYPRLVGEYEALFLHTLLPAVYENSKSISYQPSSTSNGWLSLNHSAPAKEIMIQRYNNKTKGAVYGETDYYNYNSSFLGNTTSYPVGRFSNEFGYHSMPSLQSWRQQISEKDLQFNSTTILLRNQHNPNSDLDIYNLTNSRVGQGQMTTAVENWYPIPEKTDPIANFSAWCHATQIFQAEFYRSQIEFYRRGSGLPNRCLGSLYWQLEDIWVAPTWAGVEYDGRWKVLHYVAKDAYSHVIIAPYFDRATGNLSVWATSDLWEDVSGSATFAWYNWQGEKLDVQMDESKDVTIGALNSTQVLQTNTTEILSSSDHDNAILRLEIQVEGRLPNSNTTETFTHASFFTPGALKDAQLVDPGLQLSYDDSSKKFSVTATQGVAAWVWLDYPSGTVVHFESNGFWLGKNETREIGFTVQKDETYGTWKDGVTVESMWNQTLST |
| 302 | Unigene0002915 | 45 | 18665 | 5.7 | MKLLSVLSSALLATAVTARSTLVKRDDNLSVPGENPLEFCQDPADNILAIKKVDLDPNPPKAGTKLQVAATGILAEDVEKGATVAYSVKYGIITILHSTADLCETVANVDLECPLKKGDLTLTKDVDLPAQIPPGTYTVEATVLSKDEKVITCLKAKVQFKAGGSALEGLFKEGL |
| 303 | Unigene0008749 | 45 | 8528 | 16.4 | KGQDLDQNDIPQECRSTCQPVVDTENRCDQQFDDDDNNNNLQPGIPANQQYRDCVCQDQNAAQALASCVECVS |
| 304 | Unigene0010922 | 45 | 20557 | 4.6 | LRQLNVIHRASRPSRPDKARRLGYKAKQGYVIYRVRVRRGGRKRPAPKGATYGKPTNQGINQLKYQRSLKSTAEERVGRRAANLRVLNSYWINQDSTYKYYEVILVDPQHKAIRRDPRINWIVNPVHKHRESRGLTATGKKSRGLNKGHRYNNTTSGRRHTWKRQNTLSLWRYR |
| 304 | Unigene0010923 | 45 | 24217 | 3.9 | MGAHKYLEELQKKKQSDVMRFLLRVRCWELRQLNVIHRASRPSRPDKARRLGYKAKQGYVIYRVRVRRGGRKRPAPKGATYGKPTNQGINQLKYQRSLKSTAEERVGRRAANLRVLNSYWINQDSTYKYYEVILVDPQHKAIRRDPRINWIVNPVHKHRESRGLTATGKKSRGLNKGHRYNNTTSGRRHTWKRQNTLSLWRYR |
| 305 | Unigene0006606 | 45 | 22654 | 15.5 | MPGFADTQLEPVKLISLEEMQGCMAAVYEWADSYDSKDWERLRKCIAPTLRIDYRSFLNKLWEEMPAEEFVQMASDPHVLGDPTLKTQHFIGGTKWEKVSDDEIIGYHQLRVPHQKYTDETLTKVAVKGHAHSFNLHWYKKVNGVWKFAGLNPDIRWFEYDFDKVFETGRNQMGDEQEQKNLAAQAEDSILPPS |
| 306 | Unigene0013210 | 45 | 25806 | 6.6 | MPPSKWSAINGASQPKEDPTTESFTITAPPKTDIWRRSDTDDVFNAPTLYQKLPAKTFKSISVTVYAPWHTQYDQGGLILAFPSSATPKATENEIKKAEDEEPQNPVRPTKWVKAGIEFFELSSVLGIVGTDRFSDWSLAPMSQEYHQKARFKIERKEKTLWVYARQEGEEKLKPMREIKWAFMEGRDEEDIWVGVYAAKPTPEEGEDDEKGIEVSFSDLELELED |
| 307 | Unigene0012278 | 45 | 57282 | 1.7 | MVNSLPFALAAASLLLPATNAFYDKSSPVIQADGKSFNNIVNKSNHTSIVEFYAPWCGHCQNLKPAYEKAAKSLSGLAKVVAVNCDEEPNKQLCSQMGVQGFPTLKIAKPGKKPGRPIIQDYQGARSAKAIVDAVIEQIPNHVKRLKDDDYAAWVAEGTGPKAILFSNKGPVSPMLKAVAVDFLGGLDVAQIRDEETKAVGTFEVEKFPTLVLLPGDGKDPVTYNGEMKKDKIVKFLSQAATPNPDPPKSEKKTKSASTDKKKDASSSSKFAKSSASQKSSESEAGETATPETKPTPKAAAPKPEATPIASLADPLALQHKCLNDKAGTCVLALLPEHTSSESTTLAIKSLNEIQQKYEGRKLFPFYQIPHANAQNPALRSKLDLASDKVAIIAINGKRDWVRHYTVADFHQSKVEDWIDAIRMGESPKKKLPEGLVVDASELPAESVVIEDPDQVRMKETAPDPDNVEPESDAVKKMKEQLKAQMPEGMQFEMEEVSDEEYEKIMKQGEAAGKKSDAADDHDEL |
| 308 | Unigene0005536 | 44 | 55095 | 6.8 | LLARLRLVTSTPSGNDEALTVRASTGFITGRTNANYTNVREFLSVPFGQDTAGQNRFRPPLAVPLSSERIDATEYPPACPQYVTSQKTIWNQKIPQYLQEWRASNLTAGISAPFATEDCLKLAIWTPANATHASRLPVAMFWTGGGFQTNGILVPGQLPGRWVSRSQSHIVVTINYRMNIMGFPNAGGISTQNLGLLDQRLALEWVRDNIAGFGGDPSKITIWGQSAGAASVDIHNYAFYNDPIARGFFAQSGSIIAFAPSVPDATHSNFTFVAKNVGCDFPSNFTAELECMQKVDHNDIINFLGRYQDNGTQPGISFSTVADEKLVFSNYTERYLQQRVTRAPMIYSSTANEGGSLSTYPADDPQRGVNQTEADQTTLQILCGAARSGALRNGLGLPTYRYQYAGNWTNQDPLPWMGAYHSSDLVMFFGTYEDGVGPATPLEIETSETMEDLLLDFVRDPWNGLSNATWPAYDTTASNGGTLLRFGADGKAVQEVNADEVE |
| 309 | Unigene0012746 | 44 | 48064 | 3.3 | MMSVQQIQSKGIFHGLPVYPPELVGLTAIVTGANGISGHYMLKVLSEDPKRWKRIICLSRRPPLIPGGFPDNAEHIPVDFLQEPKNIADVLQAHKVEADHVFFFSYIQPTPKEGAGLWTNAQELVEVNSKLLSNFLEALKLAGVTPKRFMLQTGAKNYGGHLGPTRLPQEETDPRVELEPNFYYPQEDLVFQYAKDTGCGWSVHMPGPIVGAVPDAAMNIAFPLAVYATVCQKLGQPLEFPGDAASWQMHSSMSAAQMNAYQEEWAVLLSPPNQKYNTCDNSAFTWEKAWPRIAGWYGIDSKGPQDGDQYVEYPSRFNPRGYGPKGITRRKFRMVDWAKKPEVQQAWRETVKEHGLTQELKEVDRVFGFLDGSLCRPAPLLFSMDKSRKLGWHGFVDSSEAILEVFKDFAKLNMIPPVPLVKVSFN |
| 310 | Unigene0012448 | 44 | 58760 | 4.7 | MTSSPSTAKTSGLLSSATTTTTSQLRVDSISRHLHPTNSANMSGQTEAVGDFGLIGLAVMGQNLILNAADHGFTVVAFNRTVAKVDRFLDNEAKGKSVVGAHSMEEFVAKLKKPRRMMLLVMAGQPVDDFIENLLKSGIEEGDIIIDGGNSHFPDTNRRTKYLASKGIRFVGSGVSGGEEGARYGPSIMPGGNEEAWPYIKDVLQSISAKSDGEACCQWVGDEGAGHYVKMVHNGIEYGDMQLICEAYDIMKRGLGMSGKEMADVFAEWNKGVLDSFLIEITRDILYFNDDDGTTLVEKILDAAGQKGTGKWTAINALDMGQPVTLIGEAVFARCLSSLKGERTRASEKLTGPTPNFTGDRAQFLKNLEQALYGSKIISYAQGFMLMQSAAKEYGWKLQKPEIALMWRGGCIIRSVFLKDITKAYRSNPDLENLLFDDFFNKAIHEAQEGWRDIVSKGALWGIPTPAFSTALSFFDGFRTKDLPANLLQAQRDYFGAHTFRIKPEHASEKYPEGKDIHVNWTGRGGNVSASTYNA |
| 311 | Unigene0012910 | 44 | 38803 | 3.6 | MQFKNLLFAAAAALVSAQDAAAPPALADALSTTDDLSSLNTVLQRFPDLVATLAGASNITIFAPSNSALEAAAQALESLQGTPDVIAALLTYHVVNASVLSANISETPTFAPTLLTNETYTSLPEGQVVGVRREGENVVVISGAGARSNVTTADVQIANGSVVHIIDSVLTLPIDVYQTAVAANLTALAGALNATNLTEPLAASEQITVFAPTTEAFDDISSALANLSVADATAILGYHVINGTVAYSSNLTNTSVETTGGSVNITIIDGAVFVNAARVIAADVLIANGVVHVIDSVLNPNGTAQPDPASDDAVVQFEGATDEGDVPYISGVPTATGAPLPTNDDVAAGYTPPASENAVSSSEGAAPRATGAVAAAVLFGAAGFAANM |
| 312 | Unigene0007373 | 44 | 59235 | 1.9 | TAAQTNASKILASIQDQLRGPGGAVAIVKDGETLARHTYGYADLDRRIPLTPQMHMPVCSISKQMVCLVLVSLKHEPTPLMAKYPGDIWEQLAGELQKVLPHLVERGLTVPDLYNMQSGIRDYWAMTTLWGAQPEGPFSIGLDAPRAIARTKSFHFEPGTEYSYSNVNFHILGSMIEAVAGQSLGQLLDERVFKPAGMRSAALCPNTAGHPLPVVGYDGDEKHGYKAGVNRIEWSGDAGIVATLDDMIAYEKWLGKTYAEEGSLYHTVAKSTTYRDGSAAKYGYGLARGETAGKAWLGHGGALRGFRLWRMHVPEEHLSIVVMFNHEGDSAAPADAILKRLLNWQEPGKTLSTADSKLFGAYLDVETQLLVRVEKSDDEGYVNVTYHIAPEKTKLTSGTSVESKGMIANLEADTLHVQRLRDNRVLQAKRLNVPKGEETLAKGDLYTGSYHCADSDNIFHCFGAGSMLYGSFDGFLGKGPAYLMRYVGEDIWLLSNPRGMDAPAPGDWTVVFRRDGSGVVTNATIGCWLARKNEYARV |
| 312 | Unigene0007374 | 44 | 59235 | 1.9 | TAAQTNASKILASIQDQLRGPGGAVAIVKDGETLARHTYGYADLDRRIPLTPQMHMPVCSISKQMVCLVLVSLKHEPTPLMAKYPGDIWEQLAGELQKVLPHLVERGLTVPDLYNMQSGIRDYWAMTTLWGAQPEGPFSIGLDAPRAIARTKSFHFEPGTEYSYSNVNFHILGSMIEAVAGQSLGQLLDERVFKPAGMRSAALCPNTAGHPLPVVGYDGDEKHGYKAGVNRIEWSGDAGIVATLDDMIAYEKWLGKTYAEEGSLYHTVAKSTTYRDGSAAKYGYGLARGETAGKAWLGHGGALRGFRLWRMHVPEEHLSIVVMFNHEGDSAAPADAILKRLLNWQEPGKTLSTADSKLFGAYLDVETQLLVRVEKSDDEGYVNVTYHIAPEKTKLTSGTSVESKGMIANLEADTLHVQRLRDNRVLQAKRLNVPKGEETLAKGDLYTGSYHCADSDNIFHCFGAGSMLYGSFDGFLGKGPAYLMRYVGEDIWLLSNPRGMDAPAPGDWTVVFRRDGSGVVTNATIGCWLARKNEYARV |
| 313 | Unigene0013690 | 44 | 20761 | 7 | DYGALEPAISGQIMELHHSKHHNTYVTSYNTQIEKLQEAQHKGDIQAQIAIQPLINFHGGGHTNHSLFWENLAPKSQGGGEPPSGALSKSIDSHFGSLDNLKSQFNTALAGIQGSGWAWLVQDTQTGAIQIKTYANQDPVVGQFRPLLGIDAWEHAYYLQYQNRKAEYFSAIWDVVNWKAAEKRFK |
| 313 | Unigene0013691 | 44 | 25463 | 5.6 | MASTLIRQSALRTALRSSAPSTAKKAALASTTFTRGKATLPDLPYDYGALEPAISGQIMELHHSKHHNTYVTSYNTQIEKLQEAQHKGDIQAQIAIQPLINFHGGGHTNHSLFWENLAPKSQGGGEPPSGALSKSIDSHFGSLDNLKSQFNTALAGIQGSGWAWLVQDTQTGAIQIKTYANQDPVVGQFRPLLGIDAWEHAYYLQYQNRKAEYFSAIWDVVNWKAAEKRFK |
| 314 | Unigene0004139 | 44 | 13532 | 10.2 | AQKKGQKVTKKFIINATQPTQDRIFDPSAFATFLQQRIKVEGRTGNLGDNVTVNNLGDGRIEVVAHQEFSGRYLKYLTKKFLKKQQLRDWLRVVSTQKGEYSLKFFNVVGDEAEDDDE |
| 314 | Unigene0004140 | 44 | 13532 | 10.2 | AQKKGQKVTKKFIINATQPTQDRIFDPSAFATFLQQRIKVEGRTGNLGDNVTVNNLGDGRIEVVAHQEFSGRYLKYLTKKFLKKQQLRDWLRVVSTQKGEYSLKFFNVVGDEAEDDDE |
| 315 | Unigene0013950 | 44 | 44887 | 2.9 | MATERVKSILGQLLPGQTPLDKITQKNPDDIVITLAIRTPLCKGKKGGLKDTPLDGIVFKLLEQVVRKSNIDPALVEDICLGNVSDSKAAYYNRAAMLAAGFPNTTAGSSVNRFCSSGLKAVQDIANQIEHGDIEIGVALGAESMTAGGDRLERPFFEDILKANQEARDCMMPMGQTSENVGKDFNITREQQDRYAAESYRRAEVAQKAGWFDDEIVPIKAMVDGKEVTLTRDEGPRYGTTFEALNKIRPAFPDFGDRSTGGNSSQVTDGAAAVMLMKRSKALELNQPIVAKFVGATVAGLAPRIMGIGPSIAVPKLLSKYNLTIDDIDIVELNEAFASMAVYCKDVLKIPYEKMNVRGGAIALGHPLGCTGARQIVTGLSEARRQKKKILLTTMCIGTGQGMAGLFVNEQNV |
| 316 | Unigene0014249 | 44 | 32883 | 6.6 | KHDQLSVSTTSGRVHGKIDAAFPNVRQFLGIPFAKPPTGSRRWLAPQALSHSKGHVEATELPPSCPQFLTPSGSDVYTNDVLEFNLAGLNETGPTSEDCLTLSIWTPTVESGTKTSYQDPNCTENQEADSSNLLPVLIYIYGGSFKTGGQNVPYQIPTQFVSRTSSHLVVSFNYRVNLFGFPFASSLPDQNLGLLDQRLAIQWVQQNIRAFGGDPERMVLWGQSAGAVSVDFYGFTYAEDPIVKGLIMDSGTSFLSVQAGRNAPLPGDNTGANFTTVANGVGCAGLAEGEEMVECMRGVDWR |
| 317 | Unigene0008959 | 44 | 12206 | 13.2 | LRTQRSLLHQASLATFASIAUMIADSECSLSIWLLPVLGSDUFALRMYGGCYNFCNEPQKTFEVPECNGQKKTEICEYMSNDCGRVFGGCYPPEGPIPGWAEPPCP |
| 318 | Unigene0012675 | 44 | 71116 | 2 | MFSAGLRQFSGRGAVRAASRNFSSTPRANRIITSAPLRAKEASGAIGHKYPVIDHEYDAIVVGAGGSGLRAAFGLAEAGFNTACISKLFPTRSHTVAAQGGINAALGNMHEDDWRWHMYDTVKGSDWLGDQDAIHYMTREAPASVYELENYGCPFSRTDDGKIYQRAFGGQSREFGKGGQAYRCCAAADRTGHALLHTLYGQSLRHNTNYFIEFFAIDLIMEDGECKGVIAYNQEDGTLHRFRAHNTVLATGGYGRAYFSCTSAHTCTGDGMAMVARAGLPNQDLEFVQFHPTGIYGAGCLITEGSRGEGGYLLNSEGERFMERYAPTAKDLASRDVVSRSMTMEIREGRGVGPEKDHIYLQLSHLPPEILHERLPGISETASIFAGVDVTKQPIPVLPTVHYNMGGIPTKYTGEVITVDEKGQDQVVPGLFACGEAACVSVHGANRLGANSLLDLVVFGRAVSHTIRDNFSPGKPHKEISGDAGAEAISVVDQIRTADGPKSTNEIRSDMQKVMQSDVSVFRTQESLDEGVKRINEVDKQFHQVGIKDRSMIWNSDLVETLELRNLLTCAVQTAEAAAVRKESRGAHAREDYPDRDDENWMKHSLTWQKKPHEKVDIGYRAVQANTLDENECKAVPPFARKY |
| 318 | Unigene0001004 | 44 | 73448 | 2 | MAHRIGRTAFDATASTLRSSTSPSIRSSTPAWRKHQRIAPLPPQQKRGILGHTQRRRATTASGSYLDPNKHPLIDHHYDAIVVGAGGAGLRAAVGLAESGLETACITKLFPTRSHTVAAQGGINAALGNMTEDDWRWHMYDTVKGSDWLGDQDAIHYMCREAPKAVYELENYGMAFSRTEDGRIYQRALGGQSLKYGKGGQGVRCCSAADRTGHAMLHTLYGQSVKHNTNFFIEYFAIDLLMVDGECVGILCMSMEDGTLHRVFARNTVLATGGYGRTYFSATSAHTSTGDGNAMAARAGLANQDFEFIQFHPTGIYGAGVLITEGSRGEGGYLLNSEGERFMERYAPTAKDLASRDVVSRSMHMEIREGRGVGPDKDHVYLQLSHLPADLIMERLPGIAETASVFSGVDVTKEPIPVLPTVHYCMGGVPTNWKGEVLNIDQTTGQEKVVKGLYAAGEVACVSVHGANRLGANSLLDIVVFGRACALDIAANNDKGAPHTKVPEDIGLDHIASMERIRQADGDKLSAEIRTDMQKVMQADISVFRTKEALDAGNERMQKVQEDFNTRLAVKDKSMIWNSDLIETLELKNLLTCAAQTSKSAQLREESRGSHAREDFPSRNDDKWMKHTLSWQKDIGQETKIGYRGVVMDTLDKSEIDTIPPVARTY |
| 319 | Unigene0010349 | 43 | 18250 | 4.8 | MSTQYENLSSDLVWEVTRNSNSFLVKRRLGSGKQVVFSRDPLNLVNKHSRKYVGYANEQAIGINADGNTVNLTTKLASRGNKPAKQYQTSSFSASTPSRKLYTSIVNSTAKKGYRSDLRAEAVSRASAVKQSQRENKKDRVSKPRGKKASGAQGSQFLKLTPEEK |
| 320 | Unigene0001472 | 43 | 29506 | 4.2 | MPPKAGGKKAAPAPFPASKAGVSKKQAKNPLIERRPRNYGIGQDIQPRRNLSRMVKWPEYVRLQRQRKILNMRLKVPPAIAQFSNTLDRNTAAQAFKFLNKYRPETKAEKKERLHKEATAVAEGKKKEDVSKKPYAVKYGLNHVVALIEAKKTALVLIPNDVDPIELVVFLPALCRKMGVPYAIVKGKARLGTVVHKKTAAALAITEVRAEDKNELSKLVQAVKEGYLEKNEEAKRHWGGGIMGAKSQAATRKAKQRADREIKI |
| 320 | Unigene0001471 | 43 | 29375 | 4.2 | PPKAGGKKAAPAPFPASKAGVSKKQAKNPLIERRPRNYGIGQDIQPRRNLSRMVKWPEYVRLQRQRKILNMRLKVPPAIAQFSNTLDRNTAAQAFKFLNKYRPETKAEKKERLHKEATAVAEGKKKEDVSKKPYAVKYGLNHVVALIEAKKTALVLIPNDVDPIELVVFLPALCRKMGVPYAIVKGKARLGTVVHKKTAAALAITEVRAEDKNELSKLVQAVKEGYLEKNEEAKRHWGGGIMGAKSQAATRKAKQRADREIKI |
| 321 | Unigene0001198 | 43 | 14358 | 5.1 | MTGGKSGGKASGAKSNAQSRSSKAGLAFPVGRVHRLLRKGNYAQRVGAGAPVYLAAVLEYLAAEILELAGNAARDNKKTRIIPRHLQLAIRNDEELNKLLGHVTIAQGGVLPNIHQNLLPKKTTGTKPGKGGASQEL |
| 321 | Unigene0009869 | 43 | 14912 | 5 | MAGGKGKTGGKTGGKAGGDNSGKSQKSHSAKAGLQFPCGRVKRFLKSQTQNKMRVGAKAAVYVTAVLEYLTAEVLELAGNAAKDLKVKRITPRHLQLAIRGDEELDTLIRATIAFGGVLPHINRALLLKVEQKKSKKAEA |
| 322 | Unigene0002608 | 43 | 59427 | 7 | MAQTHKRQISKANGKEIDIDVLVIGAGPTGLGAAKRLQQIDGPSWLIVDSNETAGGLASTDVTPEGFLYDVGGHVIFSHYKYFDDCINEALPKEEDWYTHQRISYVRYKGVWVAYPFQNNLSALPKEDQVKAMEGLIDATVESRVAREKPKNFDEWILRTMGTGIADIFMRPYNYKVWAVPTTDMQCSWLGERVAAPNLKLITTNVILNKTAGNWGPNATFRFPAHGGTGNIWIKVADTIPKEKKLFGKHGEVQLVDAENKRVLLGDGTTVNYQKLVSTMSVDYLAQAMKNEELVNLSKGLFYSSTHVIGVGVRGVRPERIGDKCWLYFPEDNCPFYRATIFSNYSPNNQPEASKKLPTLQLGDGSKPSDTSAREGPYWSIMLEVSESSKKPVNQETMLADCIQGLVNTEMLKPEDEIVSTYHRRFDHGYPTPSLEREGVLTQLLPKLEKLDIWSRGRFGSWRYEVGNQDHSFMLGVEAVDNIVNGAVELTLNYPDFVNTRQNTERRLVDGAQGLKAKNAQQNIVSSR |
| 323 | Unigene0003292 | 43 | 16746 | 6.5 | MSSAIPTSSTVVESAVIKAPLGQVWHHIKLEQFGSWWSAVKQSDFVKGASPDTDIVKWTFKDGTVLEVKQEEHSSINHYITYSIITAQPELSYSSVVSTVRAYAITSGDLEGSTFVEWTGNFSNDADAGVIQDAKFKRREALADLAKVATSKK |
| 324 | Unigene0010490 | 43 | 45486 | 5.2 | AGRLDEWKAIADKMPSIRTASRVALSGVAAAQSVLGLMQSPVERQDAFAASATCNNPQLSCQNTTAQNDLCCFNSPGGALLLTQFWDTDPVVGPADSWTIHGLWPDNCDGTYEANCDDRRAYTNITQILQAAGKRDLVDYMHTYWQSNSGSAETFWEHEWGKHGTCISTLDPDCYTDYKPTEEVPEFFQKVVDIFKTLPTYQWLGDAGVTPSTSATYSLSQIQQALAKNHGGKTPYIGCRSGAVNEVWYFYNIRGSAQTGEFVPIDTLTKSNCPSSGIKYKLKGGSGGSPTSTTTSGGSQPTSSPGTPFAGSGFLNVVTGGSQKGCIISAGTWYTSGTCATITATPLGNGFTLKSSKGSCGIASGVFSCGSGVPSTVFTADGNTLQASGSGDFSTDSVPSGSTQVKVYTGSDHSVDVTIQWQGK |
| 325 | Unigene0003397 | 43 | 22780 | 4.4 | MEVLLGITGKDFTIIAASKAAMRGATILKASDDKTRSLNKHTLMAFVGEAGDTIQFAEYVQANVALYSMRNGTDLTPNETASFVRSELAKSLRSRSPYTVNLLLGGFDHHTNKPTLHWIDYLASCAPVPYAAHGYAQYYCLSTLDKHHHPDISFEQGMKILRMCTDELKRRLPIDFKGVLVKVVDKDGVREIEYDDSQQVLAP |
| 326 | Unigene0000847 | 43 | 34536 | 2.7 | TKLVKNSAYFSRYQVKYKRRRSGKTDYYARKRLITQAKNKYNAPKYRLVIRFTNKDIIAQIVTSELTGDKVFVAAYGHELKRYGITHGLTNWAAAYCVGLLIARRALNKLELEDTFTGVEEADGEFTETQPEEVDGETRRPFKVFLDVGLTRTSTGARVFGAMKGASDGGLYVPHSEKRFPGYDIETKELDAETLRKYIFAGHVAEYMETLADDDEERYKSQFSGYIDDEIEADGLEELYQEAHKAIREDPWKKDDEAGEKKSKDEWKAESLKFKQNKLTHEERRQRVQEKIKALASEL |
| 327 | Unigene0003186 | 43 | 30726 | 4.7 | ASFAALAAAAPVVQRQAAPPPAGIDDAVILQYALSLEHLENTFYREALTKFTAADFKAAGVPSAEAFYNNLQQIASDERTHVDFLETGLRAAGATPVAECSYNFGYTDAQSFLATANILEGVGVSAYLGAAKYIASPDYLTAAGAILTVESRHSSYLRNVQSPPQSPFPAPFDIPLEFNSVYSLAALFITGCPDTPGTTGLGLKAFPGITAAPAGISKVGDVLTFTVASEVEATNAYFITFPGGAIPAEITGSGANYQVTVPAAAQPGQAYVVLTKNGDAPTDDNIVAGPAVLQIAD |
| 328 | Unigene0014501 | 43 | 11746 | 8.7 | MSARYVSSSLLKNVKSKSGKQVALEVVDKTTSWSRPSGFPSEGVKQEFVKIAENNTEKFKPEVVKIAMKESEHQSDKDKRMHYTAYGLDKDGNVVDVQHLTQHK |
| 329 | Unigene0009332 | 42 | 38434 | 3.1 | DDVIIKVTTTCICGSDLHMYEGRTAAEQGLVFGHENMGVITEIGSGVTLLKKGDRIVLPFNVADGRCRNCEEGKTAFCTGVNPGFAGGAYGYVAMGPYQGGQAQYLRVPFADFNALVLPQGTEHEADFALLADIFPTGWHGLVLAGFQSGESVAVFGAGPVGLMAAYSGVIRGASRVFVVDQVKERLDQAEKIGCIPINFRDGDPVQQIIEKNGGMVDRAVDAVGYQAVDKDGSKEQPNIVLDQLIMVTRPTGGLGIPGLYVPSDPGAPDEKSGKGQILLSFGKLFEKGLKLGTGQCNVKAYNRYLRDLIISGRAKPSFVVSHELSLEDAPMAYDKFDKRIEGYSKVLLHPNGPLQ |
| 330 | Unigene0014547 | 42 | 17275 | 6.7 | ATKLATLLLIAGIQGFTFTAPDTSAPLNLSAPSIEIRWDRGDQPYNEVDLRFKAATTGSSLFTYYLADNVSIADGSFTWQPANVSEALQSTQTVLPNEKAYSFEALLHNANSSSGAGIESEKYSVTGYPYTGSEEGGEPRTISAATSVQARAGVVLAVLASIVV |
| 331 | Unigene0009498 | 42 | 51689 | 1.7 | MPSALKLAAVTLAVGLAAAAPAPAKRQDNGSWNPGKYEGAGTPYDDGKWSPGKYPGGGSNGGGGSQQPVAPQPYRGHAKGQPEKSLNVQLGPRPWYLVDNMDDGPLKEKLESCSEGPFKTSSFSISHRGAAMQFPEHSREGYEAAARMGAGIIECDVSFTSDRELVCRHSNCDLHYTTNILAIPELAAKCSEPFVPFNPETGDLATAKCCTSDITLSEYKTLCAEMESGTFNALNTSQYNGRLGQTPDWRTNLYAADCAEPVSLKEQIALVDSYGLNFTAEAKTPEIDMPFEGNYTQENFIQQIVDTFHSASIDPSRVWLQSFLPADIFYWIDNTPAYAAQAVYLDERADLNATGYDEAVASLPGLAERGVKIIAPSIWQLVTADNETQSIVPSTYATAAKDAGLEIITWSFERSGPLEEGGGYYYSSVSNLINNDGDQFEVIDVIAQQVGATKIFSDWPGTSTYYANCFGLE |
| 332 | Unigene0007212 | 42 | 68278 | 2 | MSKKGDKKQQQQQDGEEQGGSIFSISGPVIVAQNMIGVAMYELVKVGFDQLVGEVIRIDADKATIQVYEETAGVTVGDPVLRTGKPLSVELGPGLMETIYDGIQRPLKAISDKSNSIYIPRGIDVPALDRTRKWEFTPNDKFKVGDHITGGDVFGSVKENTLLSDHKIMLPPRARGKITKYPKKGEYTVDEKILEVEFEGQKFEYSMMHPWPVRVPRPSNDKLSSGDPLIVGQRVLDALFPSVQGGTVCIPGAFGCGKTVISQSLSKFSNSDLIVYVGCGERGNEMAEVLMDFPELTIDFDGRKEPIMKRTCLIANTSNMPVAAREASIYTGITISEYFRDQGKNVAMMADSSSRWAEALREISGRLGEMPADQGFPAYLGAKLASFYERAGKVVSLGSPDRQGSISIVGAVSPPGGDFSDPVTTSTLGIVQVFWGLDKKLAQRKHFPSVNTALSYSKYTKALEKYYQENAPEFPRYRERIRELLSQSEELDQVVQLVGKSALGDSDKITLDVATLIKEDFLQQNGYSEYDQFCPLWKTAWMMKAMMTFHDEAQKAISQGHNWSKVRESTSDVQSELRSMKFEIPSEGEEAITKKYEELVQRMTEKFAAVIDE |
| 332 | Unigene0007211 | 42 | 36598 | 3.6 | MSKKGDKKQQQQQDGEEQGGSIFSISGPVIVAQNMIGVAMYELVKVGFDQLVGEVIRIDADKATIQVYEETAGVTVGDPVLRTGKPLSVELGPGLMETIYDGIQRPLKAISDKSNSIYIPRGIDVPALDRTRKWEFTPNDKFKVGDHITGGDVFGSVKENTLLSDHKIMLPPRARGKITKYPKKGEYTVDEKILEVEFEGQKFEYSMMHPWPVRVPRPSNDKLSSGDPLIVGQRVLDALFPSVQGGTVCIPGAFGCGKTVISQSLSKFSNSDLIVYVGCGERGNEMAEVLMDFPELTIDFDGRKEPIMKRTCLIANTSNMPVAAREASIYTG |
| 333 | Unigene0012406 | 42 | 24314 | 7.4 | MSKITVAGVRTNVQELLKYSNEEKKRNFLETVELQIGLKNYDPQRDKRFSGTIKLPKIPRPNMSICILGDQHDIDRAKHGGVDAMSVEDLKKLNKNKKLIKKLARKYDAFIASDSLIKQIPRLLGPGLSKAGKFPTPVSHADDLTGKINEVKSTIKFQLKKVLCMGVAVGNVDMTEDELIGNIMLAINYLVSLLKKGWQNVGSLVIKASMSPPKRLY |
| 334 | Unigene0014114 | 42 | 47175 | 4.1 | MAFGKIYSYERNPRTTAILAVAKANGLDLEFVETKPGADITNDYRKLNKLGKVPTFEGADGYVLTEAMAIAIYLTSQNEKTTLLGKTKQDYASILRWMSFANTELLPSVGAWFRPLVGRDPYNKKSVDDAQKATQANVKVLEEHLTVNTYLVSERLTLADLFVAGIASRGFEHFFDKEWRAQHPAFTRWFETVVHQDIFTAVWGEYKFIDKAIPNTPPKVEKAPKAEKPKAAPKAAAKPAADEEEEEAAPAPKPKHPLEALGRPTFVLDDWKRQYSNEETREVALPWFWKNVNFEEYSLWQVDYKYNDELTMTFMTSNLIGGFFARLEASRKYIFGAASVYGKTNDSIIKGAFLVRGQEALPAFEVAPDYESYSFTKLDPKKAEDQEFVNDQWSWDKPITVDGKEYEWADGKVFK |
| 335 | Unigene0008766 | 42 | 13185 | 10.9 | RLKPQPTDSSQWTDQDGKNRVWEAAARKTRSKGGVDAVAIAPILRHPNRPPQTMVILQYRPPVEAYCVEFPAGLIDEGETPEQAAVRELKEETGYEGKVCDVSDVLSSQPGMTNANMSV |
| 336 | Unigene0012880 | 42 | 62263 | 2.8 | MPTPVNTDQILKDVNILGKLDDQRRRVLSKEATIFLAILHRTFNSTRKALLERRQVRQQELDNGHQLDFLPETKHIREDKTWRGAPPAPGMADRRLEITGPVDRKMVVNALNSDVWTYMADFEDASAPTWDNMINGQVNLYDAIRKQIDFKQGEKDYKLRTDRTLPTLIARARGWHLEEKHFTVDGEPISGSLFDFGLYFFNNAHELVKRGAGPYFYLPKMESHLEARLWNDVFNLAQDYIGMPRGTIRGTVLIETITAAFEMEEIIYELRDHSSGLNCGRWDYIFSVIKRFRQNPQFVLPDRSSVTMTVPFMDAYVQLLIKTCHTRGVHAMGGMAAQIPIKNDEKANAAVMDQVRTDKLREVRAGHDGTWVAHPGLAPIAGAVFNEHMPTPNQIHRRREDVNINANDLLNMNVPGKITEDGIRKNLNIGLGYMEGWLRGIGCVPINYLMEDAATAEVSRSQLWQWCRHGVPTAEGKKVDKQYALKLLYEQADELEKNGPKGNKYKLAAKYFATQVTGEEYAEFLTSLLYNEITNQGSPLPASQL |
| 336 | Unigene0012881 | 42 | 62306 | 2.8 | MPTPVNTDQILKDVNILGKLDDQRRRVLSKEATIFLAILHRTFNSTRKALLERRQVRQQELDNGHQLDFLPETKHIREDKTWRGAPPAPGMADRRLEITGPVDRKMVVNALNSDVWTYMADFEDASAPTWDNMINGQVNLYDAIRKQIDFKQGEKDYKLRTDRTLPTLIARARGWHLEEKHFTVDGEPISGSLFDFGLYFFNNAHELVKRGAGPYFYLPKMESHLEARLWNDVFNLAQDYIGMPRGTIRGTVLIETITAAFEMEEIIYELRDHSSGLNCGRWDYIFSVIKRFRQNPQFVLPDRSSVTMTVPFMDAYVQLLIKTCHTRGVHAMGGMAAQIPIKNDEKANAAVMDQVRTDKLREVRAGHDGTWVAHPGLAPIAGAVFNEHMPTPNQIHRRREDVNINANDLLNMNVPGKITEDGIRKNLNIGLGYMEGWLRGIGCVPINYLMEDAATAEVSRSQLWQWCRHGVPTAEGKKVDKQYALKLLYEQADELEKNGPKGNKYKLAAKYFATQVTGEEYAEFLTSLLYNEITNQGEKVPAAKL |
| 337 | Unigene0000001 | 41 | 22002 | 6.9 | VTWAQRSSASEAEKNHIFLAVNVPDVDPKKVKLDIQPSSLTFSGYSESKKAEYGVTLEFFAEIDPSASKIHHSPRAIELVLQKKELKAEYWDRLLKDSKKVHFLKTDFDKWVDEDEQDEVADDDDYMSRMGGMGGGMGGMGADGGFGGIDFSKLGGAGLGGMGGEDDEDADDDDEEMPELEGEDDAKEGAAGADKGKAKIEEV |
| 337 | Unigene0006380 | 41 | 24161 | 6.2 | MSAQPTDTTDAKVGSKTLTPEVTWAQRSSASEAEKNHIFLAVNVPDVDPKKVKLDIQPSSLTFSGYSESKKAEYGVTLEFFAEIDPSASKIHHSPRAIELVLQKKELKAEYWDRLLKDSKKVHFLKTDFDKWVDEDEQDEVADDDDYMSRMGGMGGGMGGMGADGGFGGIDFSKLGGAGLGGMGGEDDEDADDDDEEMPELEGEDDAKEGAAGADKGKAKIEEV |
| 338 | Unigene0010009 | 41 | 69747 | 0.8 | AGFFEQGGRLTIDDVHYVASPDGQLVPAAQTPFARDATLGYTQSNLRDUIREKSKGAIASERLHSISLHDSRVGGAEAVEAKLLSVGRRSVIIVNAVVTADLDIFVSGLLRARATGRTFIYRTGAACVSSRLGIQQIPPLKPRELSMDMSPSAPGGLIIAGSYVPKTTEQLESLIQGRGTKLITIELDAAALLHESESASAAVLAAANKAGDHISRGKDVFLMTSRKLITGTDELSSLKIGNVVAAALISFLRFLIPRPRYIIAKGGITSSDAATAGLMFKRAKICGQAAPGVPLWQCAEQTCKFPDLPYVVFPGNVGERDTLRNLVANWAKPERGEDLPPPMQYQRLGSSGLKVSRLILGCMTFGNPDUEGSPWVLPENEALPLLKQAYDSGIITWETANTYSNGWSEIIIGKALRKYNIPRSKVVIMTKLYYPVLENEPNRRPQPALNDGPLVNQMGLSRKHIFDAVEGSLRRLGTSYIDVLQLHRLDVDTSPEEIMRALHDLVQMGKIHYLGASSMYCWQFARLQYTAKLNNWTTFTSMSGLYNLLYREEEREMIRFCEAEGIGLIPWSPLARGLLSRPHSEQTDRSANDAKTKKWFQGDQNSSIVNRVEAMAEDKGCTMSDLALAWL |
| 339 | Unigene0006956 | 41 | 32672 | 10.2 | GFLDEIYPKPAKAIWDFEGINCSSRHIPGVRFAGLIHPGILGCAPSQEVLELWNKREGELISACSHMDRDVAKPPEPVNVHVGTGDEKIKEKVGKEGARTIPGRPEHGGNCDIKNLSRGSKVYLPVHVKGAKFSVGDLHFSQGDGEISFCGAIEMAGVITIKFSVMKNGLSRLGTKSPIYIPGPVEPQFGPGRYIYFEGFSVDENGKQHYLDATVAYRQTCLRVIEYLRRYGYDDYQIYLLLSCAPVQGHIAGIVDIPNACTTLGLPIDIFDFDIRPEAEVVKRDLGACAKASS |
| 340 | Unigene0007605 | 41 | 50649 | 4.4 | SAWAVARAAGEYKSERYNGFEKSKKGDRIRCINGKAEAVAGDANQTYACNGIDMYDFKTHAELGSNGGEGSGSWGVTLQGRDFIAIGQTDGAAFAEVTKQGKLVYLGRLPAQSTPIIWREIKANGPYMIIGSEAVAHGVQIFDMRKLLKVDPKKPKTFSTLTDVTVFNDPIGLPTGRSHNVVVNEERNYAVAVGAAPRNTTCNSGLVFINMDNPTKPYMSGCAPQDGYVHDAQCIVYRGPHKKYYGKDICYGYNEDTLTIYNVENKKGLTAAEIISVTPYKGASYSHQGWVLDANWQTHLVLDDELDEGLVAPDEVNPESPALDGFPVTYIFDITNLEKPVNTGFYKSKVKSVDHNQFVHNGLSFQSNYQAGLRVLDVSSIPRDPSGKGVREIAFFDTYPQDDSLPGGGLPDWTYGTWSHYSFPSGWIVINTIDRGPFVVKLNNFRKRGFGSEYVKR |
| 341 | Unigene0006607 | 41 | 29830 | 5.2 | TILVPVSFLVSPALAWWKPQPNTTWQIILSETLNPSSLPAVPVIDSDLFDNSKSTWTAVKARNIKTICYFSSQYEDWRPDASNFTSQPQNLGDSLDGWPGERWVNTRATGIRDIMRARLAEAKARGCDGIDPDNVDAYLHSGGGFGLTQSDAIDYVRFLAAEAHKQDLAVGLKNGDDIVNQLVDYVDFAVVEECQANSDLTTGSGYYDECKKYQPFIQKGKAVFSIEYVEGTPSKTTTQRICKNAIAKGFGVLIKRYDLGAWVVDCS |
| 342 | Unigene0006692 | 41 | 65696 | 3.5 | PHYGAAKISPYGIRTSPYYSSVTHSNGPCMIVTLAGQIGARPDGTVPSDPVEQYKLAISNLGRCLEAAGARVQDILKLNYYIVNYDSKNPRHRPILMEFLGDHRPASTLIPVEQLAVPDFLFEIEATAAIPQHATERVDVVVVGAGLSGLQAAVDLHKAGKTVKVLEARDRVGGKTWSRSAQGSVCDVGAAWINDTNQSKMYALARKYGLELITQNTDGSIVCDEGIGNHKSHPYGQLLASATDKAQIEDIIRVRDIFEATCQQIDISNPVASGQKLRKDLDDITFEQWLQTLNPSEHAINALRVGTRAMLGVEPSELSALYFLDYCKSGGGYMQMRSDQKNGGQYLRIYSGTQSFSKSLASELPGDALILMSPVRRIEQIEGGGVRVTSARGVFEASRVIVSVPTPLYREITFSPPLPVEKLELSRSSRLGDYCKSIVFYQSPWWRKHGLTGMSQSAKGPCVVTRDSSVDADKHYSLTCFIVGQPARDWMVLSDEGREKAVLEHIGKLFGKFAHVEEPIHIEEQIWRNEQWSQGCPCPVLGPGGLTKFEKVLREPAGKVHFVGTETAYEWKGYMEGAVRSGERGAAEVLRGL |
| 342 | Unigene0006691 | 41 | 43012 | 5.5 | LELITQNTDGSIVCDEGIGNHKSHPYGQLLASATDKAQIEDIIRVRDIFEATCQQIDISNPVASGQKLRKDLDDITFEQWLQTLNPSEHAINALRVGTRAMLGVEPSELSALYFLDYCKSGGGYMQMRSDQKNGGQYLRIYSGTQSFSKSLASELPGDALILMSPVRRIEQIEGGGVRVTSARGVFEASRVIVSVPTPLYREITFSPPLPVEKLELSRSSRLGDYCKSIVFYQSPWWRKHGLTGMSQSAKGPCVVTRDSSVDADKHYSLTCFIVGQPARDWMVLSDEGREKAVLEHIGKLFGKFAHVEEPIHIEEQIWRNEQWSQGCPCPVLGPGGLTKFEKVLREPAGKVHFVGTETAYEWKGYMEGAVRSGERGAAEVLRGL |
| 343 | Unigene0012169 | 41 | 31721 | 5 | DFAQRNLNTVREIYGLTVYPNNVPILKQGCDKALRPGLFSPDATGRVSPVGDFTGFNDSCEYFFALAPNPEDQEFQGLAIYQADVVEFTSGCPNIAASVVYLRTAKYDQNTRQIDPNVPVSTLAQVAFWQFDAYGQVERYHAWITNLEAWIKAGTGVDFSALLYQKFVPLVLCPGIQQRCTGPYQQYLDVTTCILELELKPFGSFDEVWGDNVACRLIHLILTQVRPDIHCPHVGPTGGGKCVDINYSVDYFSDLELFGLPEGSVFTCGGPLTDPMNRAAVG |
| 343 | Unigene0012168 | 41 | 11044 | 14 | DFAQRNLNTVREIYGLTVYPNNVPILKQGCDKALRPGLFSPDATGRVSPVGDFTGFNDSCEYFFALAPNPEDQEFQGLAIYQADVVEFTSGCPNIAASVV |
| 344 | Unigene0000406 | 40 | 63205 | 4.3 | MASRSLTFAASLRNASTTTTGLRAGQTSGLATLANFKPPTIHNEPNKHYEKDSVDRTKLAAAIQALRSQAPLQVPIVVAGKESRTQTISEQPIPYSHKDSIASFSNATAQDAQNAIESALAAKDSWESLPFADRAAIFLKAADLVAGKYRYDIMAATIVGQGKNAWQAEIDSAAELADFLRFNVKYAQELYSQQPEHNSPGVWNRVEYRPLEGFVYAVTPFNFTAIAGNLPGAPALMGNVVVWKPSPAAIASNWLVYNILIEAGLPRDVIQFVPGDAVEITKAVLAHKEFAALHYTGSTSVFRSLYGQIGQGTAEGRYRGYPRIVGETGGKNFHLVHRSADVENAVLNTVRGAFEYQGQKCSATSRAYFPKSLWDQAKPLLIKETQKLKIGSPEGFGNFIGPVIHQQSFNKLSKAIDESNKDSALELVVGGKHDGSKGFFVHPTIYEAKTPDHKNFTTEFFGPILTVYVYDDTTDPVKAYQDICKTIDTTSEYGLTGAVFAQDREALVYAENALRNAAGNFYLNCKSTGAVVGQQPFGGARASGTNDKAGSANLLSRFVSMRALKEEFNAIPSVEYPSNIV |
| 344 | Unigene0012114 | 40 | 58452 | 4.7 | KHYEKDSVDRTKLAAAIQALRSQAPLQVPIVVAGKESRTQTISEQPIPYSHKDSIASFSNATAQDAQNAIESALAAKDSWESLPFADRAAIFLKAADLVAGKYRYDIMAATIVGQGKNAWQAEIDSAAELADFLRFNVKYAQELYSQQPEHNSPGVWNRVEYRPLEGFVYAVTPFNFTAIAGNLPGAPALMGNVVVWKPSPAAIASNWLVYNILIEAGLPRDVIQFVPGDAVEITKAVLAHKEFAALHYTGSTSVFRSLYGQIGQGTAEGRYRGYPRIVGETGGKNFHLVHRSADVENAVLNTVRGAFEYQGQKCSATSRAYFPKSLWDQAKPLLIKETQKLKIGSPEGFGNFIGPVIHQQSFNKLSKAIDESNKDSALELVVGGKHDGSKGFFVHPTIYEAKTPDHKNFTTEFFGPILTVYVYDDTTDPVKAYQDICKTIDTTSEYGLTGAVFAQDREALVYAENALRNAAGNFYLNCKSTGAVVGQQPFGGARASGTNDKAGSANLLSRFVSMRALKEEFNAIPSVEYPSNIV |
| 345 | Unigene0007619 | 40 | 10530 | 8.3 | RDQSMLTFEGNPTQGGAAITTKLSELPFQRVEHQVATLDAQPSNEGGGILVIVTGALLVEEERRPMSYSQTFQLSPDGQGSYYIFNDIFRLVYPAA |
| 345 | Unigene0007618 | 40 | 14180 | 6.3 | MSADFQGVAKQFVEYYYKTFDTNRNDLAALYRDQSMLTFEGNPTQGGAAITTKLSELPFQRVEHQVATLDAQPSNEGGGILVIVTGALLVEEERRPMSYSQTFQLSPDGQGSYYIFNDIFRLVYPAA |
| 346 | Unigene0007191 | 40 | 29094 | 2.9 | GRLAGKNAIITGAAGGIGLETTILFLREGANVLMTDISEAALEKAIAKAKEHAPSASGKVEIAKVDVSKEADVEAAVAKLDAWGGLDIIFNNAGIMHADDADAVDTPEKIWDLTQNINVKGVWFGSKHAVLSLRKHNKTKGSIINTASVVALVGSATPQLAYTASKGAVAAMTRELAIVHAREGYRFNSLCPAPLNTPLLQDWLGDDQAKRHRREVHFPSGRFGEAIEQAQAVLFLASDEASFVNGHDMVVDGGMTKAYVTPEGPATQPPSNLAR |
| 347 | Unigene0005791 | 40 | 14481 | 8.1 | MSSGKVKASQLWGKNKEDLKKQLDDLKAELVQLRTQKIAGGASSKLNKIHDVRKSIARVLTIINANQRAQLRLFYQKKKYLPLDLRSKQTRAIRRRLSPEDAKRVTEKQKKKQRHFPQRQFAVK |
| 347 | Unigene0005792 | 40 | 14580 | 8 | MSSGKVKASQLWGKNKEDLKKQLDDLKAELVQLRTQKIAGGASSKLNKIHDVRKSIARVLTIINANQRAQLRLFYQKKKYLPLDLRSKQTRAIRRRLSPEDAKRVTEKQKKKQRHFPQRQFAVKV |
| 348 | Unigene0001602 | 40 | 55288 | 5.8 | MFKAVLPRAPMRAALRTATQSSALRVSPQSTSLLQSQLLQRRGYATPAEEKDLVIIGGGVAGYVAAIKAGQEGLKVACIEKRGALGGTCLNVGCIPSKSLLNNSHLYHQILHDTKNRGIEVGDVKLNLAQMMKAKETSVSGLTKGIEYLFKKNGVEYIKGTGAFADEHTVAVNLVDGGETSVRAKNIIIATGSESTPFPGLTIDEKRVITSTGAIALQEVPKKMVVIGGGIIGLEMASVWSRLGSEVTVVEFLGQIGGPGMDTEISKNIQKTLAKQGLKFKLNTKVVEGDDSSDIIKIKTEAAKGGKEETLDADVVLVAIGRRPYTSGLNLESIGLETDNRGRLVIDSEYRTKIPHIRVIGDCTFGPMLAHKAEEEAVAAIEFITKNYGHVNYNAIPSVMYTHPEVAWVGQNEAELKEAKVNYKVGTFPFSANSRAKTNLDTDGLVKFLADAETDRILGIHIVGPNAGEMIAEGTLALEYGASSEDVARTCHAHPTLAEAFKEAAMATHGKAVHY |
| 349 | Unigene0004695 | 40 | 30056 | 2.2 | VDAEFPDGKLSCDTFPSDYGAVPVEWVGLGGWTGVQCPGSGSSGTGIDNIRTVTHGGNCEEGAYCSYACPAGYQKSQWPTTQGATGQSVGGILCKDGKLHLTNPGMSNKLCMKGSDKVQILVKNKLDKNVAVCRTDYPGTESETVPVDVQPGQTSNLTCPEADNYYTWQGGKTSAQYYVNPAGVPVEDACQWGTPDNPWGNYAPMNLGVGYSNGAAWLSIFQNAPTTDAKLEFCVEIVGDGISGSCKYENGQYCSGTSCSATTGCTVSLSSGTATYVFS |
| 350 | Unigene0006565 | 40 | 42515 | 5.8 | LSALVAASPSVASILPAPEDKLLLAPDLVNFHRALVEIESISGNEKAVGDWLYESLVNQGYHAERQYVSKDPERFNVFAWPGHNRDPPVILSSHIDTVPPFLPYKSTKHKHNTTIFGRGSVDAKGSVATQIIAVNELLAASKISRDDVGVLYVVGEEVGGEGMKYANQLSLTPKTIIFGEPTELKLVSGHKGILSVHLTTKGKSGHSGYPWLGRSANEVLVSALAAIMQLGDKLPKSDKYGTTTFNLGRIEGGVAANVIAQDASANIAVRIAEGTPELIKKEITIAVHDAVASFLEDDKDLKFSDIIEIDFTGEGYGPIDIDDDVPGFDVITVNYGTDIPWLKKTVKDQKRYLYGPGSILVAHSANEELTVKDLEDAVEGYQKIVLFALGKGEK |
| 351 | Unigene0000074 | 40 | 25004 | 11.3 | MINQTGSLGEDGIHIDMNHLKSGEVNLGTSIMAINFKDGVILGADSRTTTGAYIANRVTDKLTQVHDTIWCCRSGSAADTQAVADIVQYHLSTYGITNDEAPTTQTAAALFQELCYENKDMLSAGIIIAGWDPRHGGQVYQIPLGGSLHKQAFAIAGSGSTYIYGYTDANWKEGMTEEEGIEFVKGSLKEAIKWDGSSGGVIRMVVLTAKGAVRHLYLPDRNYEGPGTDKP |
| 352 | Unigene0014526 | 40 | 36183 | 2.4 | AASPPPAGIYVPVPTFFAKEGSANYDEVSPPIDIETQSEHSLFLVRGGVKGLVILGSTGEAIFVRNSERHELIKSQRKTLDDAGFKDRPIIAGTATQNIDDTVEMIKDSQDAGAEYAMVLGPAYFAAATSQAGIQKWFEVVADRSPIPILIYHYPGVTNNLFIAPSTFEKLAAHPNIVGCKLSHGIIDDQTLIAASPNIDHDHFYVFTGLGQNLLPVLTIGGVAAIDGLAGCFPRVVVKLFKQFHESAAKGTSKEDLQLQRDLQFRICEGEKLIATWGPVGMKEAIARVWGIGSGKGNRLPLAGGFQDGDKEWTKWEKVFNGLRDLEKQFEA |
| 353 | Unigene0014889 | 40 | 17103 | 16.1 | PNQLATNLPGPTTYLTGHDQNTGKAIIHASNPVNWAKFEDDKLAMSVGFTTQFPADLNNDADVTAHQEKLSSGKLGLVSGGGTVLRYVDFAPDYECMMHRTQSVDYGIVIEGTIESVLDSGEVQVMKRGDVMVQRATMHAWRNPSKTEWARMIFV |
| 354 | Unigene0007779 | 39 | 24659 | 3.6 | TIGKDRKDYGADILYSETNDSVPQINFEEGQFIDYRAFDKHNSTPSYEFGFGLSFTTFNYSNLEISKLEAPAYKPFSGKTKPAPTIGNFSTKAEDYVFPANFSRVRGFHYPWLNSTNFTLATGDPHYGLNISLPAHSQDGSAQVVPKAGGAPGGNPRLYDVLFNITATITNTGKVAGYEVPQLYISRGGPYDPVRELRGFEKLWIEPGESKTFAVGLTRRDIS |
| 355 | Unigene0010045 | 39 | 16656 | 7.5 | MALKRINKELTDLGRDPPSSCSAGPIGDDLFHWQATIMGPGDSPYSGGVFFLAIHFPTDYPFKPPKVNFTTRIYHPNINSNGSICLDILRDQWSPALTISKVLLSICSMLTDPNPDDPLVPEIAHVYKTDRSRYEATAREWTRKYAI |
| 356 | Unigene0004206 | 39 | 9859 | 22.7 | KIPTDLREEIDQAFRNVDMAVKEAGGSGIEQVYKLNIYFAPLTEEAMESCGTALKEWFPKHAPLLTAIGVEKLALEGMRVEIEAWALD |
| 356 | Unigene0004207 | 39 | 14312 | 15.9 | SHLQYFSYAKNFHYSQSVRLPHNQILVSGQPGLNRETRKIPTDLREEIDQAFRNVDMAVKEAGGSGIEQVYKLNIYFAPLTEEAMESCGTALKEWFPKHAPLLTAIGVEKLALEGMRVEIEAWALD |
| 357 | Unigene0005770 | 39 | 57569 | 2.9 | MASIRTTARSSGSILCRACKRPAATQSFANAPAWRALSTEGQQKLLSANLEEADSTVFEIIRKEKRRQKHFINLIPSENFTSQAVLDALGSVMQNKYSEGYPGARYYGGNEFIDEAEILCQNRALETFRLENDKWGVNVQPLSGSPANLYAYSALLNTHDRIMGLDLPHGGHLSHGYQIPNKKISMISKYFETFPYRLDESTGLIDYDKLEEQALLYRPKIIIAGTSAYSRLIDYDRFRKIADKVGAYLLADMAHISGLVAAGVVPSPFDFADIVTTTTHKSLRGPRGAMIFFRKGVRSVDKKGKELLYDLENPINASVFPGHQGGPHNHTITALAVALHQAQQPEFKDYQRQVLENAQALANRLGSDKESGGLGYNIVSGGTDNHLVLVDLKDKAVDGARVERILELVGCAANKNTVPGDKSALKPGGLRMGTPAMTTRGFQAADFKRVADIVHRAVNITKSVDAKAKEAAEKSGRKNPGSVNAFKEYVKEGEEVVEILELRREVEDWVGTFSLPWTESS |
| 358 | Unigene0003273 | 39 | 47072 | 3.6 | MSKGKVCLAYSGGLDTSCILKYLLDQDYEVICFMADVGQEEDFEAAKAKALKIGATKCYIEDIRREFVEELCYPAIACNAIYENVYLLGTSLARPVIARAQVAVAQKEGCFAVSHGCTGKGNDQVRFELAFYALQPSIKVIAPWRDPAFYERFKGRQDLLDYAAEKGIPVTSTKSKPWSMDENLAHCSYEAGILEDPNTEAPADMWKLTVDPTKAPDTPEDFTLTFEKGLPVKLEYEGGKKTVTDPVELFLTANAIARKHGVGRIDIVENRFIGLKSRGCYETPGLTMLRAAHVDLEGLVMDREVRALRDQFVTFHYAKILYNGLYFSPEREFLEDSIKSSQKHVNGSVRCRVYKGMFSAVGRWSDTEKLYDASESSMDEIGDFAPSETTGFITVNAIRLKKYGSAKETAGESLVKK |
| 359 | Unigene0011048 | 39 | 85349 | 2.6 | MRVSQLFAASSYLLCAASTQSILFPSNDSKSTWELSSEFVLSREHDRLQISSPTLDIWSTATDKPFIRASAGNDSVTGSNGAFNITEIDIDTTDGQDIEDVQLVDWDGTATGKAARISGQLTFKGQLSGQYALYFWVPSDLSDRIAFYLDIISATEDPLKKLYFSFTSNAGEDFYGLGGQASFASLKNQSIPVITREQGVGRGDEPITSLENANNSIAGGSFYTTYTSVPSYISTDGKVFYLSEKSTGYSNFDFTQPDAVTIRYDSLSVDGGFTRTETLFDAIEALTAYTGRQPALPKWVDDGAILGIQGGQDKVNRIVEQGFELGAPIAGVWLQDWVGTHSQVGPYLNISRLWWNWENDDALYPTWTEFVQNLRDQYNVRTLSYINTFLTNVSTKDTGFRRNLYEEASANHYFVQNTTTNSTAIISSGPGLQAGVVDLTNPYLRDWYEDVLRQQVWNSNISGFMTDFGEYTPLSGDVKVYDAVSDAFFYHNAYPTQWAAYHRALVEDLGLQDEAVLFHRSAAQSSQKYMNLFWVGDQNVDWGVNDGIKSVVTVMVHMGFSGYSQQHSDVGGYTTVLTYNNYNITRSPELLGRWGELAAVSSAAFRSHEGNIPSVNAQFYSNTTTYEYYGYNARLFASLGKYRRHILETESEPKGWPLLRPVVLEHPQDLRARNISYQSFYLGQHLYVAPVLDPQTFEVTMYLPAGNGNRTYEHVWTGTKYNGGQDVTVAAPYGKPAVFLVNGARTPELQPFLDFVKAENGTKLSV |
| 360 | Unigene0004834 | 39 | 55459 | 1.5 | LSAFPSLLNANLEDLVTGLEKGLFTSVDLVQAYTARIEEVNDLLHAVTEVNPDALSIASSLDAQRHNGTILGPLHGIPILIKNNIATADQMNNTAGSYSLLGAKVPNDSTMASKLRKAGAVILGKTNLSQWANFRSDNTSNGWSAYGGQTTGAYYPNQDPSGSSSGSGVASSIGLAFASLGTETSGSILSPSDVNGLVGIKPTVGLTSRYLVIPISEHQDTVGPMARTVKDAAHVLEAIAGPDANDNYTSAWPFEKVPDYAGACRFDSLVGARIGVAWNVLDIWRDFIDQPVIDAFNAAIEEISAAGAIIVEANFTGFAEYNNTVSGEVLNADFISGLANYLSELSYNPNSVESLSDVRNWTHENGAIEAWPERDTDVWEEALFNQTWNNTDPRFWDAYQTNLYVAGEGGILGALKRSNSSAVLLPTQISPIIPALVGSPVVSVPMGYYPYDWNVTMNGFGNLVASGPNIPFGLSFMGDYWSEEELISLAYAYEQRTLHRAQVKPFVQPNSEIVHVV |
| 361 | Unigene0013765 | 39 | 45813 | 1.9 | MVNQKAQDAVQQSLDSVTGNKDTGVAGLVFVAIDKNGQQICANASGKKGVGENRAPMDLDTVFWIASCTKILATMACMQAVEQGILNLDDSAQVYKLCPELEKVQVLREDGTLEPKKSDITLRNLLSHTSGFAYEFFNPKLRDYGRPTGFDVFHADIRDILKMPLVHHPGENWEYGIGIDWAGIVLERASGVKLNDWIQKNIMQPLNLDAINMFPTADMKKNLAYMHQRWPGSSGSEERDHIYREPIIAETDEQKKNVFHSGGAGAFAKPVQYVQVLSVLLNDGKSPITGAQILKKETVDEMFKNQIEKFPDFARQGIPAAKPEQTNPAPELYPQEGNPPQGWGLSFMLTQETGATGRGSNTAWWAGIANLYWWADREKGVAGMIASQVMPFGDMNVMGQWGACEAAVYQATG |
| 362 | Unigene0009137 | 39 | 56469 | 2.7 | MKWVTFLLLLFISGSAFSRGVFRREAHKSEIAHRFKDLGEQHFKGLVLIAFSQYLQKCPYEEHIKLVQEVTDFAKTCVADENAENCDKSIHTLFGDKLCAIPKLRDNYGELADCCAKQEPERNECFLQHKDDNPNLPPFQRPEAEAMCTSFQENPTSFLGHYLHEVARRHPYFYAPELLYYAEKYNEVLTQCCTESDKAACLTPKLDAVKEKALVAAVRQRMKCSSMQRFGERAFKAWAVARMSQRFPNAEFAEITKLATDVTKINKECCHGDLLECADDRAELAKYMCENQATISSKLQACCDKPVLQKSQCLAETEHDNIPADLPSIAADFVEDKEVCKNYAEAKDVFLGTFLYEYSRRHPDYSVSLLLRLAKKYEATLEKCCAEGDPPACYGTVLAEFQPLVEEPKNLVKTNCELYEKLGEYGFQNAILVRYTQKAPQVSTPTLVEAARNLGRVGTKCCTLPEAQRLPCVEDYLSAILNRL |
| 363 | Unigene0002444 | 39 | 11305 | 6.9 | MAPATVTVVYPQGAKFNMDYYKSTHMPLVQEKWGKFGLKSWKVIQFSDDSPYCVQATLEWDSISDFQKAGGSPEAKDVMADIPNFSDKEPVIMAGEVQLTS |
| 363 | Unigene0002443 | 39 | 11305 | 6.9 | MAPATVTVVYPQGAKFNMDYYKSTHMPLVQEKWGKFGLKSWKVIQFSDDSPYCVQATLEWDSISDFQKAGGSPEAKDVMADIPNFSDKEPVIMAGEVQLTS |
| 364 | Unigene0014498 | 39 | 29457 | 2.8 | MPTIPRLENAISPEPSTLPIRKHKFDCPKLRLQLHDVNHEGSAIFLSNIKASEDFETQVQNVLNLLYDVPSAHRPGTRSITLILREMDGVAYTTGIDLDEDHKEIHFNLNYINRTRPDQRHELLGVVCHELVHCFQWNAEGTCNGGLIEGIADWVRLRAGLAAKHWKHEASGNWDGGYQHTGYFLDWLEHKFGPGTVRKINGCLRKGKYDENLFKECCEGHSVKKLWKEYGESLEQEKKKHDDESKPKEEIKS |
| 365 | Unigene0004774 | 39 | 44708 | 6.1 | MSHRKFEAPRHGSLAFLPRKRAARHRGKVKSFPKDDPKQKCHLTATMGYKAGMTTIVRDLDRPGAKLHKKEIVEACTVIETPPIIVVGLVGYIETPRGLRSLTTVWAEHLSDEVKRRFYKNWYKSKKKAFTKYAKKHAEDSGKSITRDLERIKKYCTVVRVLAHTQISKTPLKQKKAHLMEIQVNGGSIADKVEFGHGLFEKPVEIDTIFEQDEMIDCIAVTKGHGYQGVTSRWGTKKLPRKTHKGLRKVACIGAWHPSHVQWTVARAGQMGYHHRTSVNHKVYRIGKGSDEGNATTDFDHSKKTITPMGGFVRYGEVKNDFVLLKGSVPGVKKRVMTLRKSMFIHTSRRALEKVELKWIDTSSKFGHGAYQTPTEKKQFVGTLKKDLVTQA |
| 366 | Unigene0006212 | 39 | 17902 | 5.2 | MSLVTGEKSNFQFILRLLNTNVDGKEKVMYAMTKIKGVGRRYSNLVCKKADVDLNKRAGELTSEELERIVTILQNPTQYKIPTWFLNRQRDIVDGKDFQVLANGVDSKLRDDLERLKKIRAHRGLRHYWGLRVRGQHSKTTGRRGRTVGVSKKKG |
| 366 | Unigene0006211 | 39 | 21954 | 4.2 | MSLVTGEKSNFQFILRLLNTNVDGKEKVMYAMTKIKGVGRRYSNLVCKKADVDLNKRYDPHDTTMAHDAPNIHSDYRANRIMATYUHLFCSAGELTSEELERIVTILQNPTQYKIPTWFLNRQRDIVDGKDFQVLANGVDSKLRDDLERLKKIRAHRGLRHYWGLRVRGQHSKTTGRRGRTVGVSKKKG |
| 367 | Unigene0013361 | 38 | 18085 | 5.2 | HFASILFCATQCLASELQVAPRLKWKEFPLGESKESKSCGSKTCLPCERIKWGMIADHRTCSQIDQDDAIHGVNDVFWAVSGNCHPQVTFEGNKLNIITQTYDCHASIQMPRAHKKVDWAAVKGKTLPGRANYFHLVNWETDGSVWCTLEERRTC |
| 367 | Unigene0013362 | 38 | 18085 | 5.2 | HFASILFCATQCLASELQVAPRLKWKEFPLGESKESKSCGSKTCLPCERIKWGMIADHRTCSQIDQDDAIHGVNDVFWAVSGNCHPQVTFEGNKLNIITQTYDCHASIQMPRAHKKVDWAAVKGKTLPGRANYFHLVNWETDGSVWCTLEERRTC |
| 368 | Unigene0003859 | 38 | 131019 | 0.7 | MRTLLQQLLALGPELAAYILTTSLPVARAAFVQSAASSPNLDLSQLGRVAVGGDFDSISLYTYQGQNENTSTNGSQSLLTRYPDGAFQSLALADADTSIRAMCSFVVNGNLQGVVVGGNFTSLGGVRADSIALWNPDSNEVTALPGLSGGPVNALYCDDESGTVYVGGQFMAGNSSNAMAWTTGWVNLPFAGFNGPVNSITKDSGNNIVFAGQFTVLGNTTTTSTESIPQAVSIGSGDITAAGSTGRAGFDNPNNVICKNGEDQGSGNTWLLNDNTGGYWESALGFGVSPTALRIYNTDYEGRGTKSFYFEEMNTAGILELEYVDPTTGQNATCIRFCPLPEGNTTVQEFRFVRRVAMTTFRIWITDFYGAGAGLNGVELVSDEIYNFAVNDLNSPSCVDTAAASVSTATPADAWGRSRNPGLISSDYLSTVINSEANVGEDTNVVFQPNLQQSGNYSVTLYTPGCIIDESCSSRGRVEITASMTADSDPATTSSFQTNNYDKFEQIYYGYIDTDGFQPTVTLAPAAGQRVPLTVVASRVRFELISAAGGNSSTSTSSSSTTGGLNGLFEYNPNEAPTSTNFSTSVVNRAGTSLDGRATFASVIRYNDNLYAAGNFSGDGISNIMSIGSDGPASLPRGGLNGEVLNMMLNGSTLYIGGIFTNTAQDQTEGLNSVGSFSIDSNEWAPLGAGVNGGVFSIVPLTMNMTAEDRRDTLAISGNFTSVNGFGDNAAFDAAGFAIWVPSENNWLQNIPGTNSALNGQLTAYTTVPGMDPVYAGKIDSQGLDINDVVELVGSGTPELRSLPIRLSANTAASSSGSQKRDLTDRQTGSNYTGVYGGVFYNKNNLDITILGGSFSTTATNGSTIENLAFINNTDSDRESVSGVSGLTSDSIFIAMDTYQTALWAGGAVNGTVNGNPATGLVVYDLSADRFAAPHPPALGGENVVVHSIAAQPEGEDVYVGGDYATAGSLPCGPLCFYDTRALQWQSTGSGLEGVIYTMVWTSNTKLMIAGNLTVNGNETTMATYDAKEQTFTELGGASSLPGPVTAISAVNGDYSQFWVSGTSANNNSVFLTKYKDGEWTAASGLGDSTSIRKLQIMPLTSDHDSSDMVATDQVLMILGNINIPDQGNASAVLFNGTVYEPYILTNMEDGSQGSLSAIFVQNPNNFVNNKTSGLAVGIIVAIGLAIALAITGLIILLGLLLERRRRRIEGYVPITADKSANVNRLPPEQLFSKLEGGGHSPPKI |
| 369 | Unigene0007730 | 38 | 13987 | 13.6 | RRQLDRHPRNDEGGFNHRYPIYEHQMWLDGIFMADSFYARWTAKFDCENQTAWDDIVNQFDLIEKYTRNHTTGLLVHGYDESKSKVWADPITGAAPHVWSRAVGWYFIALLEVIPLLP |
| 370 | Unigene0008656 | 38 | 42126 | 5.5 | MLRTASNRLCLINKQIHSKTITRTIMASVSEAPKEGKFSVKIDKSGEFKRQTSSFRNWVSKDPNAEFPAEKGRYHLYVSYACPWAHRALIVRKLKGLEDTIPYTSVHWHLGEKGWRFATQDEKEPGEFTEPDPIHKEYTHLRDIYFENEPDYAGRFTVPTLYDVKQRKIVSNESSEIIRMFYHEFDHLIPEDKAKIDLLPSDLKDTIEATNEWTYNDVNNGVYKSGFATTQEAYESAVKTLFASLDKIEQHLSQSRDGPYYHGSRITEADVRLFTTAIRFDPVYVQHFKCNIRDIRSGYPNLHKWMRELYWKVPAFGETTEFTHIKKHYTKSHAQINPFGITPVGPVPDILPLEEEVAAVKG |
| 370 | Unigene0008655 | 38 | 39188 | 6 | MASSDKKSILNWVDPKDKSGEFKRQTSSFRNWVSKDPNAEFPAEKGRYHLYVSYACPWAHRALIVRKLKGLEDTIPYTSVHWHLGEKGWRFATQDEKEPGEFTEPDPIHKEYTHLRDIYFENEPDYAGRFTVPTLYDVKQRKIVSNESSEIIRMFYHEFDHLIPEDKAKIDLLPSDLKDTIEATNEWTYNDVNNGVYKSGFATTQEAYESAVKTLFASLDKIEQHLSQSRDGPYYHGSRITEADVRLFTTAIRFDPVYVQHFKCNIRDIRSGYPNLHKWMRELYWKVPAFGETTEFTHIKKHYTKSHAQINPFGITPVGPVPDILPLEEEVAAVKG |
| 371 | Unigene0006148 | 38 | 49954 | 2 | MPHKVLLLGAGFVVRPTLVELDKAGVQVTVACRTLDSAKKLVAGDLKHASAISLDVNDSKALEAEIGKNDLVISLIPYTFHALVIKAAIAAKKHVVTTSYVSPAMQELDQQAKDAGITVFNEIGLDPGIDHLYAVKTIDEVHKAGGKITGFWSYCGGLPAPENSDNPLGYKFSWSPRGVLLAARNTATFYQDNKKAEIEGKDLMRSAKPYFIYPGYAFEAYPNRDSTPYRQRYDIPEAETLIRGTLRYQGNPSIVQTFRDLGLLSEEPQDYLKQPIAWKEAFAKIVGSSSHDVKDLEWAVSSKTQFSSNDAKDRILAGLRWYGLFSDDKIEPRGTPLDVLCATLEKKQQYEQGERDFVMLQHKFEITWQDGRKEMRTSTLAEYGEPEGSGGYSAMAKLVGVPCAVAVLLVLNGKIQQKGVLAPVTPELVEPIRLELKEKYGIELQEKTLA |
| 372 | Unigene0004261 | 38 | 46614 | 2.5 | AWLHGFVGHLRRHQDRELGVLAAGHGQQPHYQWLRQREEKKSVGVGQSGMWLLIYUHLATRSPSRULDNSAAAIKQKRESSQASAINMAKKRVQCCISVDIDAVAGWLGSYGGEDSTSDISRGLFAGTIGVRRLLKLFEKYNITTTWFIPGHSLETFPEECRMIADAGHEIGLHGYSHENPIAMTLEQQTAVMDKCYKLITEFQGKPPRGIVAPWWESSQEGAELMLKYGLEYDHSFSHHDCQCYWLRTGDKWTPIDFKKHPEHWMKPLEGGPMTGLVEIPASWYLDDLPPMMFIKKAPNSHGWVNPRDVEELWMDQFDYFYREYDEFVFPVTVHPDVCGHPHGLLMLERIIEKINKHDGIEWVTMESICDDFKAKSTPPKGAFLPAEVGAIAKNPNLELKKQE |
| 372 | Unigene0004260 | 38 | 46614 | 2.5 | AWLHGFVGHLRRHQDRELGVLAAGHGQQPHYQWLRQREEKKSVGVGQSGMWLLIYUHLATRSPSRULDNSAAAIKQKRESSQASAINMAKKRVQCCISVDIDAVAGWLGSYGGEDSTSDISRGLFAGTIGVRRLLKLFEKYNITTTWFIPGHSLETFPEECRMIADAGHEIGLHGYSHENPIAMTLEQQTAVMDKCYKLITEFQGKPPRGIVAPWWESSQEGAELMLKYGLEYDHSFSHHDCQCYWLRTGDKWTPIDFKKHPEHWMKPLEGGPMTGLVEIPASWYLDDLPPMMFIKKAPNSHGWVNPRDVEELWMDQFDYFYREYDEFVFPVTVHPDVCGHPHGLLMLERIIEKINKHDGIEWVTMESICDDFKAKSTPPKGAFLPAEVGAIAKNPNLELKKQE |
| 373 | Unigene0011201 | 37 | 46289 | 2.2 | MATEVQQPPTLPDPQSAPVQQQQQPVDPSAAQGAQIPLTDFNIPSFPPEARGLQALTLTSDLKTDEYQEILQRPWTIPTTIPTSIESLTLELFSMGYPKGFLSTLADRLPNLKSLVIYSQLFAGITPETQQDAIEFFKKSRNLRALHLLDVFAKPHFFERAAKWLKYNDGSEDESHARRGLMFLEVNYTFRHEDEDFMGKIQATELPLLIGPGLISCSFNLAEPEHVSKEEDEQDPTVIAGASDKEGIMAFNKTLANELIEALTEGEQVPRGLRALNTTLYTLTPEQIRKVLDKQKGLMVMSLTAEIEPGEETKKDLLNAMELCKDLEQVEIVANPSLRFFMEVQNPRKQTLAKTFPDVFDMNRLSEKLPKLSSFKANVLRTQTLGEIEWEKAESKWKGGVKEGKGLPAGMV |
| 374 | Unigene0011405 | 37 | 58539 | 2.1 | DLSGSYSLGGQERHPDLPGGEVPLKPWYYNSTFEQLIDHKNPHLGTFDQVYFYDTTYWKGPESPVVFFTPGELNATKHARYLTPNYTTGHLAERIGAATIVLEHRYWGNSTPFTDLSTANLTYLTLENSIKDVTRFAREVQLPFAPQGGSNAQDVPWVMMGASYSGALTAAIARVDPGTFWAYAASSAPVQAVSDFWSYYVPIQKGMPRNCSKDVSLVIEHMDGVLMHGSTEEQRSLKTRFGMQDVEHNDDFMAALALGPWSWQDTQFYIDTGFWKWCDYIENAVNATARNISIPGAEGVGLEKALDGYAAWWKDVKLPDFCSSTYQYAEFNTTNNTRCYDTYNASSPLFTDISLANQVNRQWIWMTCNEPFGYWPNGSPPGRPTLVTRLITTGYALRQCGLWFPPGPNGETYGLAAGRTEADVNAYTGGWSNINTSRLVFINGEFDPWREASVSADADFRPGGPLRSTPQVPVEIVPGGFHGSDMRIRNGVVNERVKEVQERIVKQLEDWVKEWPKKR |
| 375 | Unigene0000439 | 37 | 82076 | 1.6 | MRNARRILLSLLASSLPASVLAADVLSTDGYSLCSTSDDIKVEALDASYDRNTRLITFNVAGSSAREQKVLLHLDVQAYGRQVYETDWDPCSDDKKVEQMCPVPAGNFSSKGSQTIPEEYASKIPAIAFNIPDLEGMVKLSVKSADGGQEIACVESSVGNGKTINIPSIGYAAAGVAAAALAMGAVGAMAAGGGGAGATTPSPTFTEVIGWFQGMAMNGMMSVQYPKVYQSFTTNFGFSCGLIPWGQMQTAIDNFRASTGGNTTHNSYEWLKNNATLVYTKPSNGSSVAKRALDSVLLWARDEISTSVNGTEVNIGGDGSSESNDAPTASEKEQHFVQGIQAYAEQLSIPSSSTFMNILLVWAIVVASIVVLILLFKAILEAWGQFGKLSPYFESWRKRYWWRMAKAITNLILMLYGVWTLWCVYQFTSGDSWAATVLAAVTFALFTALLGWFTWKIVSKARQAKKAQGDSAQLYENKETWIKYSLFYDNYKKGYWWIFIPTILYMFIKGCIIAGADGHGMIQVAGQLIVEALMLVLLLWSRPYERRSGKWINITIQVVRVLSVVCVLVFVQELGLSQTTQTVTGVVLIVVQSVMTGILAILIAVNAIIACVKENPHRRARKEREKMKNDFDALTPLDARNSLLMTDVKHPAYSEQDTSYKAAPIVSASPFGAPARGRYDPVEQQRRTESPAGHQFHDDDHLVSSAASMGRHEPAYRDHSCDSSISRPHDLDRQPRLPDLDFGR |
| 376 | Unigene0002462 | 37 | 22345 | 4.1 | IVIDGKGHLLGRLASTVAKQLLNGQKIVVVRCEALNISGEFFRAKLKYQAFMRKQTRYNATRGGPWHYRSPAKMFWRTVRGMIPHKTERGAKALERLKTFEGVPPPYDHKKRQVVPQALRVLRLKPGRKYCTVGRLGHEFGWKYQDVVERLEERRKVKGQAYYEKKKAARAKLADAQSKVSSDVQKQLASYGY |
| 377 | Unigene0008294 | 37 | 38297 | 2 | SAGKRTKTCYVKSHDDLKTDDSKYILDALKKCNHGGHVVFPQGTTYVIGQALDLTFLDQVDLDIQGYIQFTNDTDYWQAQSFKQVFQNATTFFELGGRDVFVYGGGMLDGNGQVWYDLYAADPLILRPIIFGTVGLNSGIISNLNLRYSPQWYNFVANSTDVVFDGLTISGYSQSSHEAKNTDGWDTYRSSNVVIQNSVINNGDDCVSFKPNSTEILVQNLYCNGSHGISVGSLGQYPGEVDIVEDIMVVNISMYNASDGARIKVWPGASAAMSVDLQGGGGLGRVNRVTYKDMYIENVDYAVEVTQCYGQKNLTLCNAFPSNLTISGIDISGIHGKTSKKYNPYS |
| 378 | Unigene0013410 | 37 | 29670 | 3.3 | MPGFDFSNHNRNLALHAAGVPLPKATSTGTTIVGALYNGGVVIAADTRATSGPIVADKNCEKLHYISPQIWCAGAGTAADTEFTTALMSSNLELHALSTGRKPRVVTVMTMLKQHLFRYQGHIGAYLVVAGCDPTGAHLFTVHAHGSTDKLPYVTMGSGSLAAMSVFETQWKPDLSKDEAVKLCADAIEAGIWNDLGSGSNVDVCVITKEKTTLMRNYITPNVRGKKERNYKFQRGTTAVLNEKVITKEEISKYVTVQDIGDGEAGVAEKMDVD |
| 379 | Unigene0011348 | 37 | 31058 | 3.8 | MASGYDRALSVFSPDGHVFQVEYALEAVKRGTCAVAVKGTAKDDQVVVMGCEKKSALKLQDTRITPSKISMVDTHVCLAFAGLNADARILVDKARLEAQSHRLTVEDPVTIEYITKYVAGVQQRYTQSGGVRPFGISTLIVGFDPGSKDARLYQTEPSGIYSAWKANAIGRSSKTVREFLERNHKDDMTRAETIELTIKSLLEVVQTGAKNIEIAIMAPGKTVEMLPSEEIEKIVEKINSDKDAAAEANAGRRGGARQGGAAAGDAASAQGPEQVLASRPAGEGSGAPPE |
| 380 | Unigene0011641 | 36 | 8487 | 8.9 | FLPLVLAAVTGASAMVLNCNAGTAGDGGCEMNGLNTYCCWSQGFSPQFPTVRDITVFSRNPEQGTSCESWGIPGTVACA |
| 381 | Unigene0003220 | 36 | 29643 | 6.1 | MSDAIVSKNLYELLGNDPELDPERATPPVPTKVVDKTLPRAGKRNGPAEGAAREGGARSGGRGGAVRGEGASRVDQQNRDDGLRGDRHSRPVRDYERRGGEGRGRGDRRGRGGYGGRGRGGAPRDDRHSHTGIGDQEKQAAHGWGAKDGQSEWADESAGDAIAKAEVNNEPGFTPDTSAADPAFTNGPDGELGEDAAGEPEEKVKSYDDYLKELAEKKLALSGESPAIRKANEGSKTKFPEGQAVNREQEDFFVGGGGKTKKVKEVKEKERINIDGQY |
| 381 | Unigene0003221 | 36 | 30937 | 5.9 | NLYELLGTPRRPUPSTRTSLVLTASPGNDPELDPERATPPVPTKVVDKTLPRAGKRNGPAEGAAREGGARSGGRGGAVRGEGASRVDQQNRDDGLRGDRHSRPVRDYERRGGEGRGRGDRRGRGGYGGRGRGGAPRDDRHSHTGIGDQEKQAAHGWGAKDGQSEWADESAGDAIAKAEVNNEPGFTPDTSAADPAFTNGPDGELGEDAAGEPEEKVKSYDDYLKELAEKKLALSGESPAIRKANEGSKTKFPEGQAVNREQEDFFVGGGGKTKKVKEVKEKERINIDGQY |
| 382 | Unigene0012420 | 36 | 18030 | 5 | PPHSHTNKLTPTSPQQDIQKSNTFYTSILGLKAEELKPAILKERMSVFPIGNTTVLLFALGQTNEDVHPIPSQPDLCVPKHGPGENAMKALLEGGEKLRQHYCLAVETKAEALEWEKYLKDQGVECHGKMEWPRGGYSVYFLDPDGHVGEIASRGIWAHW |
| 383 | Unigene0000832 | 36 | 33913 | 2.2 | MDAPTITPKFLSKVDELGLVAVGFSGGQCKPGTDAAPMALIESGLINDLRDELKYDVKYDNKVHAYGDLMPSEDPEHRRMKNPRAVSAVTKKLSSQVYEHAREGRFVLTLGGDHSIAIGTISGTAKAVRERLGRDMAVIWVDAHADINTPETSDSGNIHGMPVSFLTGLATDTAENPFGWITEDQRISPAKLVYIGLRDVDRGEKKILKDNNIKAFSMHDIDRHGIGKVMDMALGWIGRDTPIHLSFDVDALDPMWAPSTGTPVRGGLTLREGDFIAECVHETGSLVALDLVEVNPNLEAHGVSETVRAGVS |
| 384 | Unigene0008563 | 36 | 82173 | 0.8 | ENAAKSWEQAATECRAKVAAIVDECQILNQKYRDAIFDLEANQYCLQSLAGAYPKAVDNIDPPPWIKRIEDIFDEPQFYIDDATATDVHQGNSGDCWFLAALMAVSAKKELIERLCVARDEKVGVYGFVFYRDGEWIYEVIDDKLFIRVGDDDDLEVVRDWDRDAKEGLRIKYDDDKLKEALQKGGLALYFSHCKSNETWLPLIEKAYAKAHGDYFAIEGGFASEGVEDLTGGVAVILNPEDIMDKDRFWKEQLSRVNEKYLFGGGSKPTGTKGFIGGHAYAVLDKYEDPDSDLKLLKLRNPWGETEWDGDWSDGSKLWTAGMMTKLNHTFGDDGVFWISYKDFLKHFPSINRVRLFDNTWTVSQQWTCVNVPWTVDYLDTKFQITVPEAGPVVVVLAQPDDRYYYGLRGRMLYSLHFRIYKADEEERWIVRSLHNSGAETLFTRSVSAEIDNLEPGTYDVVFKVTATRSATGMTAEEAIMKYAVNRKEKLLNVGRRFDYAQSKGNLNAMEKQNRQRRRDQQREKDLSMLKKNRRLNQQDRERLKKRKARLTEALNAQMKEFHAKRSEKMKARRDRRKARKESVAALRKESVSNAETSEGKAEQLTPPEQSQSVPELPKEPETADNTEKGAETAQQPEEEASVAQQDDAKGDSGDIANKIAELEIRTNRKPSRDHRNSVSPFGPTVEESSESEWDSPIEPPDDLIDDDFDWDSEM |
| 385 | Unigene0014354 | 36 | 18438 | 6.2 | MDQIKKRMNALRIEADESAAKVEELSAKVKALEQENLQKEQEITSLQHKTSLLEQEVEKLEKLHGEAKSAADDSAQHGTQNEALTRKLQLLEEEAEANDKQLRETNEKLRQTDVKAGHYERKVQALEANNAQWETKYEEMAKKYADTKKELDDFVAEIGNI |
| 386 | Unigene0008013 | 36 | 40086 | 2.8 | QADELLRNDPDLNHEYLPIAGLPDFTSASQKLILGKTSPAIADKRVISLQTISGTGAVHLGALFLAKFYNPANAEAKAVYVSNPTWANHNQIFGNVRLPVKQYPYFSKQTKGLDFEGMIGAIKDAPEGSVILLHACAHNPTGVDPTQDQWKEIAKVMKSKKHFPFFDTAYQGFASGSLETDGWAINYFVEQGFELVIAQSYAKNFGLYGERAGCFHFVTSPGSGAAETTQRVGSQLAILQRSEISNPPAYGARIASLVLNDDKLFAQWEEDLRTMSGRIKEMRKAVRTRLEEAKTPGTWNHITDQIGMFSFTGLNEKQVEKLRSEYHIYMTKNGRISMAGLNTKNIDYFAKSVDAVVRETS |
| 386 | Unigene0008014 | 36 | 49117 | 2.3 | SSNINSNPSRHRLQQLTSQLNMGSVGDNVATAFTQDVVPLAPEDPLFGLMAAYRKDTDSKKIDLGIGAYRDNNAKPWVLPVVKQADELLRNDPDLNHEYLPIAGLPDFTSASQKLILGKTSPAIADKRVISLQTISGTGAVHLGALFLAKFYNPANAEAKAVYVSNPTWANHNQIFGNVRLPVKQYPYFSKQTKGLDFEGMIGAIKDAPEGSVILLHACAHNPTGVDPTQDQWKEIAKVMKSKKHFPFFDTAYQGFASGSLETDGWAINYFVEQGFELVIAQSYAKNFGLYGERAGCFHFVTSPGSGAAETTQRVGSQLAILQRSEISNPPAYGARIASLVLNDDKLFAQWEEDLRTMSGRIKEMRKAVRTRLEEAKTPGTWNHITDQIGMFSFTGLNEKQVEKLRSEYHIYMTKNGRISMAGLNTKNIDYFAKSVDAVVRETS |
| 387 | Unigene0010318 | 36 | 37550 | 3.8 | MSSDHAYELLALENPLLDIQGQGDDALLQQYGLKANDAILAEKQHLTLYEELIKNRDAKLLAGGAAQNTARGAQYLLKPDSVVFFGCVGKDKYADILQDANKQAGLAVRYRYDEKEPTGRCGVIITGHNRSMVTDLAAANAYKIEHLEENWAVAEKAKAYFVGGYHLTVCVPAVLKLAEEAAKTNKPFILSLSAPFIAQFFKDPLDQTAPYWDYVVGNETEAIAYADSHDLNTHDIPTIAKALANLPKKNTQRKRVAIITQGTDPTVVAVQDEGDAKSYPVHSIDKNEIVDTTGAGDAFAGGFVAGIVKGEKLETCVDMGQWLAALSLRELGPAYPFPKQAYGA |
| 388 | Unigene0002773 | 36 | 60997 | 4 | MSSKHFINDPTHLVSSALESITLTNPNTAFDAENKVIYLRPDAAPAQVSIISGGGSGHEPSFAAFVGPGLLSGAVAGTIFASPSAEQVRRCISQRVETSKGVLVVVMNYTGDVLNFGMAVEKAKAAGLEVEMVVMGDDVAVGRKKGGKVGRRGIAGTVLIHKIAGALAATGASLKEVHQVAQAVADNTVSMGSSLAHVHVPGREDHEEDLKRDEIELGMGIHNEAGSQRAAANLPENIKLMLKNMLDHSDEDRAFLKISSSDETVLLINNLGAVSVLELGGITTEVVKQLESDYGIKPVRIIAGTFMTSLNGLGFSISLLKTSDTGVGKSVLELLDAPSEAAGWSAAITSKTWTNRPTETRELARDKTAENQPSNIQIDASYANKVLGQALEKLIQAEPEITRYDTVVGDGDCGIGLKRGAEGIKKKLSEIGGQNDAALWLNEIIGVVETDMDGTSGALYAIFLNALAYGIRLQGNGGSKQADPKIWASALQEASQSLAKYTPAKPGDRTLVDALAPFVGELNSSGDVKKAAQAADEGSKKTKGMQASLGRTVYLGGSGYEEVPDPGAYGLSIFLSALAEAL |
| 388 | Unigene0002774 | 36 | 60997 | 4 | MSSKHFINDPTHLVSSALESITLTNPNTAFDAENKVIYLRPDAAPAQVSIISGGGSGHEPSFAAFVGPGLLSGAVAGTIFASPSAEQVRRCISQRVETSKGVLVVVMNYTGDVLNFGMAVEKAKAAGLEVEMVVMGDDVAVGRKKGGKVGRRGIAGTVLIHKIAGALAATGASLKEVHQVAQAVADNTVSMGSSLAHVHVPGREDHEEDLKRDEIELGMGIHNEAGSQRAAANLPENIKLMLKNMLDHSDEDRAFLKISSSDETVLLINNLGAVSVLELGGITTEVVKQLESDYGIKPVRIIAGTFMTSLNGLGFSISLLKTSDTGVGKSVLELLDAPSEAAGWSAAITSKTWTNRPTETRELARDKTAENQPSNIQIDASYANKVLGQALEKLIQAEPEITRYDTVVGDGDCGIGLKRGAEGIKKKLSEIGGQNDAALWLNEIIGVVETDMDGTSGALYAIFLNALAYGIRLQGNGGSKQADPKIWASALQEASQSLAKYTPAKPGDRTLVDALAPFVGELNSSGDVKKAAQAADEGSKKTKGMQASLGRTVYLGGSGYEEVPDPGAYGLSIFLSALAEAL |
| 389 | Unigene0011030 | 36 | 15898 | 6.3 | MSQYSASVPADGTVKPEIGSFFENFYAISDTPDGHERYADQFTNDGVLIMASNKVQGRDSIIKMRHGMWEKVAKRSHKPAQLYAFGSGSDDIMLYGTVDYTLKDGRGTTVDWSARAHFSRVGGELKMDFYQVYLDTAAMANAK |
| 390 | Unigene0010381 | 35 | 53285 | 1.8 | MGFPIPAEKIEYRHYINGEFVESSDNGKFELKSPYSHEKITDICEASVDDTNKAVAAAKEAFPAWSAKSPAERGALMKALAGKIRDAHNELAQLNAMDMGRPISTYFDGYSAASLFEHYAESAYTAKGETSLNSPGYINMTFRQPIGPVAAIIPWNIPTLSFAMKIAPAVAAGCTIVLKTSEKAPLTPTLCAKFAHEVGFPPGVINVLNGHGTPSGATLASHMDIRMVNFTGSTMTGKKIQQMAAGSNLKKVVLELGGKSPAIIFEDADIVKAATATSASMQMLMGQACVANSRIYVQESVADKFKEAFIAMFKAAKKGDPLLPETQQGPQADQLQHERIQTFLQAATEGKGKLESGGKPAQVDGKGYFIEPTVYSNVDENEKTQKEEVFGPFVNINTFKTESEALEKANATEYGLYAAVFTKDINRALRVAKAFESGTVGINCSSPTMAMDMPFGGYKGSGQGREGYGYSIEEHLEHKTVLIKIDDEAASPL |
| 391 | Unigene0002735 | 35 | 25344 | 5.4 | MSSSIKPLILHAHGTGPNPFKVAAVLESLNLPYEVKLWQFGDAANGVKGAQFTKINENGRVPALEDPNTGVTSWESGAVMNYIRRVYDKQNKLGPRSSSEQDIVDFEKWEYFLLSTLGPMMGQVNWFRHYHSSKNDDALKRYEEQAYRCFGVLDGQLKKHGGPFILPGNSVSAVDFHFYPWVYQHSFAGLSMDSYPNVKKWLEQVSALPEIKAAYEKIPKGKEV |
| 392 | Unigene0008402 | 35 | 14676 | 7.8 | FTKITPSLPVTSIPDAIEFYTTKLGFRISGRDRDDHTWLRLGGEDAEKASIPVNVYLRKRGFPEIPHDVEFGKVHIRVDGEEDELEKLYETFVANGVKVLEEVKVMPWGLKHFTVTDPDGNILNFDQAI |
| 392 | Unigene0008404 | 35 | 13162 | 8.7 | FTKITPSLPVTSIPDAIEFYTTKLGFRISGRDRDDHTWLRLGGEDAEKASIPVNVYLRKRGFPEIPHDVEFGKVHIRVDGEEDELEKLYETFVANGVKVLEEVKVMPWGLKHFTV |
| 393 | Unigene0004746 | 35 | 29974 | 6.7 | MAPTKQVKTSVATKDQILVPETLLKKRKSQEKERAEKAEQREARKKENKEKRAQIFKRAESYVKEYRDQEREKIRLTRVAKSEGSYYVPAEPKLVFVVRIKGINKIDPKKRKTLQLLRLLQINNGVFIRLTKATSEMLKIVEPFVAYGYPNLKSVRELIYKRGYAKTSQKHRIPLTDNSIIEEHLGKYGIVCMEDLIHEIYQVGPNFKQASNFLWPFKLSNPTGGFHKRKFRHFIEGGDLGNREEYINQLIRQMN |
| 393 | Unigene0004745 | 35 | 29077 | 6.9 | SSVATKDQILVPETLLKKRKSQEKERAEKAEQREARKKENKEKRAQIFKRAESYVKEYRDQEREKIRLTRVAKSEGSYYVPAEPKLVFVVRIKGINKIDPKKRKTLQLLRLLQINNGVFIRLTKATSEMLKIVEPFVAYGYPNLKSVRELIYKRGYAKTSQKHRIPLTDNSIIEEHLGKYGIVCMEDLIHEIYQVGPNFKQASNFLWPFKLSNPTGGFHKRKFRHFIEGGDLGNREEYINQLIRQMN |
| 394 | Unigene0014617 | 35 | 46179 | 4 | MLSFRLPSLPSWSSPFATGIKLTKGEVHEIEEQPEKRARTLKHLLKANHINHSIIYNELRFHNHTPHILGSAYILGGDTDHLTHIYEEEGKHLESWKDSPGEIDKSDWREYLGKREYQRAYIDFFEDQLVVNGYDWRALLDEFLLSGKEPLINNLIAGLAHPLIHLGYAHELSSRTVAIEALGLAACFYNEWHVYLDDLKYTKPAANPTDSLFSILERVRNDPQFDNLAEHQGSDNIEKILGNEELAAAALEYWNSWDLKDPKIQFAESQKLAVALVVAAQEPDNDKGYDFFGVHLLTSSHAVRTLLPFLPNKHHIPLVRQWWLFTLLVYIAQLRPKINIDTIKLVELEGRDWKFVTDKAVTGKYNTDAHYVKALRSMHECSKTWGDANQFYLKAAVKMAE |
| 395 | Unigene0014369 | 34 | 18601 | 9.5 | MRFNALTLGAFVLAATGSSALQLASAPGYNDAPLPSAPYCPPQDVSPATQKAILQRFLNKFFFEGNATGALLDHVSEGYIQHNPFINSTRQVAVDFFTAIGDLTANSNVTILNLGLDNNTGFAHTKWDTPGQEPNIIVDIWRFNGSCIEEHWDVMQRRPENATNPLAMF |
| 396 | Unigene0000125 | 34 | 27625 | 2.5 | TMAAAIQRQIDEGAVPNHVSNVCPKGTKFWLLEVGWLECDEGFVVRGGNTSLHSTKDQPFVNKRRQLPMYCILIDHPIEGVILWETGCGKDYPTVWGPQLSDVFTRVKYESHHELDAAIEATGHKLEDVKKIIIGHLHLDHAGGLDLFLDRKDIEIWVHDLELRSAFWSVATGADVGVYLKHYLRLDLNWKTFDERTLDFCQGITLHHLPGHTDGLIGMQINMPDSGTFLFISDHCHVIEN |
| 397 | Unigene0000242 | 34 | 34256 | 3.5 | IKIVFGGAGLNNGRPLAETATLKRAYDVLLAAGVKTIDTANLYGDSETILSGTGAPSRFTLDTKTRGGFNKENGASRENILKEAQNSKDKLGPVDIFYIHAPDPSTPIEDTLSAINEVHKTGFFKRFGLSNYKAEDVEKVYNHCKENEYPLPQVYQGNYSAVARKQEELLFPTLRKLGISFYAYSPLAGGFLSKSKEDIEKGSGRFNNTESGASMYKKLYGKPALLNALEQWASIAKDEGVTRADLAYRWVAYNSPLKNENGDALIIGASSVEQLQETLDSIGGGPLSDKAVKRIDAIWDEIKHEAPLDNYH |
| 398 | Unigene0010483 | 34 | 19011 | 6.1 | AEGEQNIKLVSSDNVELQTTRKVAERSMLIKNMIEDLGSPGDEPIPIMNVSEAVLRKVLEWCEHHKNDPAPSQDDDADSRKKTTDIDDWDQKFMQVDQEMLFEIILAANYMDIKALLDVGCKTVANMIKGKSPEEIRKTFNIQNDFTPEEEDQIRRENEWAEDR |
| 399 | Unigene0011587 | 34 | 29631 | 3.8 | MGRPPNLYSFGDVDKELAPNLRKYILEAQNEALSRQNGVFRIAVSGGSLPKTLAKALLAPSNGNSDDKLDFSKWEIFYADERVVPLDHEDSNHRLVKQDLLDQIPSELGQPKVFPIDEQYLDDAQECADQYEKTLVSIFAQRDSVKLPLFDLILLGCGPDGHTCSLFPDHPLLREKNAWILSITDSPKPPPKRITLSLPVVVHASKIGFVATGGGKKDIIEKIFETEEGASLPCGLVNAQAAERVSWFCDKPATEGVNFPRRGSVI |
| 400 | Unigene0009765 | 34 | 65300 | 1.5 | AVYQSLLHLSWIDNLLTTVKALFAKQYATELKKQHVSKIDTRDFDQTFDALVRKLDTGSTRRRPSELDSESQVELTPPSSSASQADEEDGPPAPPVPHFKKPAVPQVKDVVYDTTSADITPVATPDTSRPTTPLHAPSHLLAAKAGPGGLSRKQRKKANQFSNSAPVSSGDESDRRPKSSQGKAKAKRRWDASGVAQEDDGEVLDYSAAATPSNEEPNALDVDDVNATQMGSRTTKGQFVLKDLDDEVEAILAESKQQKQAASTEKEVSGLIGTASSKLSGLFRNVVGGKSLTKEDLKQPMQQIRNSLLEKNVAPEAADRLCQSVETDLLNQKTASFTSIEKTLRESMSKALTRILTPTSSLDLLRNINNTIEGRNPRPYVISIVGVNGVGKSTNLSKIAYFLLQNKFRVLVVACDTFRSGAVEQLGVHVRNLTELSRRENVGHVELFQKGYGKDAADVAKQAVTYATNAGGGNILFDVVLIDTAGRRHNDQRLMSSLTKFGQLANPDKIFMVGEALVGTDSVSQARHFAEQFVDQKGVLRNGGVGMDGFIISKCDTVGDMVGTLVSMVHATGIPVVFLGVGQHYGDLRGLNVDWAVSKLMK |
| 401 | Unigene0004966 | 33 | 21023 | 3.8 | MGLTFSKLFDRLWGKKEMRILMVGLDAAGKTTILYKLKLGEIVTTIPTIGFNVETVEYKNIQFTVWDVGGQDKIRPLWRHYFQNTQGIIFVVDSNDRDRVVEAREELQRMLNEDELRDALLLVFANKQDLPNAMNAAEITDKLGLHSLRQRAWYIQSTCATSGDGLYEGLEWLSNSLRKAGHN |
| 401 | Unigene0004967 | 33 | 21023 | 3.8 | MGLTFSKLFDRLWGKKEMRILMVGLDAAGKTTILYKLKLGEIVTTIPTIGFNVETVEYKNIQFTVWDVGGQDKIRPLWRHYFQNTQGIIFVVDSNDRDRVVEAREELQRMLNEDELRDALLLVFANKQDLPNAMNAAEITDKLGLHSLRQRAWYIQSTCATSGDGLYEGLEWLSNSLRKAGHN |
| 402 | Unigene0013889 | 33 | 16622 | 5.1 | VENADKVILHTTLGAITVKLFSQQTPRTCTNFATLAKTGKYDGVIFHRIISGFMIQGGDPTGTGRGGSSIYGNKFEDEIVPSLKHDAKGTLSMANAGPGTNGSQFFITLGPTAHLNGKHTVFGKVVEGMDVVDKLGAVRTGAGDRPVSEVKIERTEV |
| 403 | Unigene0013318 | 33 | 10712 | 10.3 | MSDDEERVTMPFKFVTAGFDARFPNQNQTKHCWQNYVDYHKCILAKGEDFKPCRQFYLAYRSLCPASWTERWDDQRENGNFPTRLDQ |
| 404 | Unigene0007362 | 33 | 8538 | 10.8 | SLADLVPHLVRRDFADSDILEVSLVDGSKRYISYGALKRNGIPCSRRGNSFTNCRTGQRANEWQRGCSAITRCR |
| 405 | Unigene0002345 | 33 | 59767 | 3.5 | MSGDAEQQAFEQEVKEVQQWWTDSRWRYTRRPYTAEQIVSKRGNLRIEYPSNAQAKKLWNLVEGRFKEKTASATYGCLDPVMVTQMAKYIDTVYVSGWQSSSTASSSDEPGPDLADYPYTTVPNKVNHLFMAQLFHDRKQREERLSAAPSERSKLANTDFLRPIVADADTGHGGLTAVMKLTKLFVEKGAAGIHIEDQMPGTKKCGHMAGKVLVPVSEHINRLVAIRAQADIMGVDLLAVARTDSEAATLITSTIDHRDHAYVLGATNPALQPLNDLMVAAEQAGKNGADLQAIEDAWTAQAGLKLFHQAVADTINAGVHVNKQALIDEFNKQAKGKSNSEARAIAKGLTGVDVQFDWDAPRTREGYYRYQGGCQCAVMRAVAYAPYADMIWMESKLPDFEQAKEFADGVHAVWPEQKLAYNLSPSFNWKAAMQPKDQETYIKRLATLGYCWQFITLAGLHQTALIADRFSKQYAARGMRAYGEIVQEPEMEEGCEVVKHQKWSGANYVDGLLQMVQGGISSTSAMGKGVTEDQFK |
| 406 | Unigene0003006 | 33 | 74420 | 1.6 | HHRSYSNPDRPSDAFRRSLSSRPPPPPSPPANDKNDLPFLVELRGKTPLLTRLRARAFEPTTLLTLLTIGVILLLQLALPKDLSLFQSRYHPQPPPSCPSPNPFVQELYCRAPAHVQGTLYGKSTSSMALAAAAERVAAEFEYSKEDVNKGVKEFIRQMDEGLGKNGATMSQIPTYVTAVPNGTEKGLYMAVDLGGTNFRVCSIQLHGNSTFSLTQSKVKIPHELMVAKTSHELFSFLAKQIELFLKTHHEDHYTSHKEGKGTSEHEVFSLGFTFSFPVQQFGINKGTLIRWTKGFDIQDTVGKDVCALLQAEIDALGLPVRVAALVNDTVGTLMARSYTSPGKTGTLLGAIFGTGTNGAYVEKLDKVTKLQKLSAQEAGEIDSSTGEMIINTEWGSFDNHLSVLPQTPYDADLDLESVNPGIQMFEKRVSGMFLGEILRRALLSLLLDFNVPLFADEHSNQNDVHSTTNVAPDSPLYKQWGLDSSFLSITTGDSSAGLKVTRQTLDQDYGVSAASAEDAEAVRLIASAVGKRAARLSAVAIAAIVISTGRLKNPADAATTNNATLEVNEDDIVDVGVDGSLVEFYPRFEEYIREALREIPEIGAQGEKKIRIGIAKDGSGVGAALIALVADKAAKGMSTQKIGSGGHADLVTGNYSSSSSASTSSSESGFFGRLIENINRSVRYRV |
| 406 | Unigene0003007 | 33 | 72780 | 1.6 | HHRSYSNPDRPSDAFRRSLSSRPPPPPSPPANDKNDLPFLVELRGKTPLLTRLRARAFEPTTLLTLLTIGVILLLQLALPKDLSLFQSRYHPQPPPSCPSPNPFVQELYCRAPAHVQGTLYGKSTSSMALAAAAERVAAEFEYSKEDVNKGVKEFIRQMDEGLGKNGATMSQIPTYVTAVPNGTEKGLYMAVDLGGTNFRVCSIQLHGNSTFSLTQSKVKIPHELMVAKTSHELFSFLAKQIELFLKTHHEDHYTSHKEGKGTSEHEVFSLGFTFSFPVQQFGINKGTLIRWTKGFDIQDTVGKDVCALLQAEIDALGLPVRVAALVNDTVGTLMARSYTSPGKTGTLLGAIFGTGTNGAYVEKLDKVTKLQKLSAQEAGEIDSSTGEMIINTEWGSFDNHLSVLPQTPYDADLDLESVNPGIQMFEKRVSGMFLGEILRRALLSLLLDFNVPLFADEHSNQNDVHSTTNVAPDSPLYKQWGLDSSFLSITTGDSSAGLKVTRQTLDQDYGVSAASAEDAEAVRLIASAVGKRAARLSAVAIAAIVISTGRLKNPADAATTNNATLEVNEDDIVDVGVDGSLVEFYPRFEEYIREALREIPEIGAQGEKKIRIGIAKDGSGVGAALIALVADKAAKGNYSSSSSASTSSSESGFFGRLIENINRSVRYRV |
| 407 | Unigene0012277 | 33 | 52212 | 5.1 | ASTSVSRLALRKMATQSSWARSPAYNAVRTYATPSKSASLKETFAAQLPEKIEQIKKLRKDYGNKVVGEVTLDQVYGGARGIKSLVWEGSVLDSEEGIRFRGKTIPECQEVLPKAPGGNEPLPEGLFWLLLTGEVPSEQQVRDLSAEWAARSDVPSFVTELIDRCPSDLHPMAQFSLAVTALEHESSFAKAYAKGIKKTEYWQHTFEDSMDLIAKLPTIAARIYRNVYKDGKVPAVQKDKDYGYNLANQLGFADNTDFVELMRLYLTIHSDHEGGNVSAHTTHLVGSALSSPYLSLAAGLNGLAGPLHGLANQEVLNWLQEMKKSVGSDLSDENISKYLWDTLKAGRVVPGYGHAVLRKTDPRYVSQREFALKHLPDDPMFKLVSQVYKIAPGVLTEHGKTKNPYPNVDAHSGVLLQYYGLTEQNFYTVLFGVSRAIGVLPQLIIDRAVGAPIERPKSFSTEHWAKLVGAKL |
| 408 | Unigene0008376 | 33 | 38940 | 3.2 | AVESKFDPKDMEFRHLGPTGLKVSVLSLGGWLTYGGTQKGNVVKDCMEAAWNAGINFFDTAEIYSNGQCEVEMGNALKELAWPRDEYVLSTKVFFGTGRKEPNTRGLSRKHVVEGLKSSLARLQQPYVDIVLAHRPDVGTPMKEIVEGFTQVIRNLNLAYYWGTSEWSAVQIMEATQIAEKYNLIAPIAEQPQYNAFHRQRFEVEFEPLYQQFQYGTTIWSPLASGLLTGKYNDGIPEDSRFATNKAFFEGTIKSLQSEEGKAKIAKVRKLTEIAEKLGGNVAQLSLAWCVKNPNVSTVILGATKVAQIEDNIGALKLLPKLTPEVLEEIEKILDNKPAKPSAFGRER |
| 408 | Unigene0008375 | 33 | 38940 | 3.2 | AVESKFDPKDMEFRHLGPTGLKVSVLSLGGWLTYGGTQKGNVVKDCMEAAWNAGINFFDTAEIYSNGQCEVEMGNALKELAWPRDEYVLSTKVFFGTGRKEPNTRGLSRKHVVEGLKSSLARLQQPYVDIVLAHRPDVGTPMKEIVEGFTQVIRNLNLAYYWGTSEWSAVQIMEATQIAEKYNLIAPIAEQPQYNAFHRQRFEVEFEPLYQQFQYGTTIWSPLASGLLTGKYNDGIPEDSRFATNKAFFEGTIKSLQSEEGKAKIAKVRKLTEIAEKLGGNVAQLSLAWCVKNPNVSTVILGATKVAQIEDNIGALKLLPKLTPEVLEEIEKILDNKPAKPSAFGRER |
| 409 | Unigene0011223 | 33 | 48562 | 1.5 | VTADFCSEGSVDDGGNYYCKCVNAITYTGVGGAGSYNKITNMDGSSGACSSSPNGYSGTLAPFDEEVSLHLRGPLILKQLAVYTPSSSSSSPTSYSRRAVHERRHIGGAHGHGHHAHLHKKRDAALGDVVNAIIDGKAVSWLNDYSGEPAASSGSSAASPQVNNAPAANRASINSAPATGASRAKTSSSSSSSSSSDDDDDEASTGEGWNRVAYYNAEKQSADGLVFLNHEGGTAGSGIFDYSFGNSLSYASADGCSGAGSPQILKDCTIPSKNEVVVMSDKKCSEGDDGCGYYRNGTVAYHGFDGPSKAFFFEFGMPDDGTTTADIYSPTNMPAIWMLNAQIPRTLQYGKPECSCWTSGCGEFDIFEVLAPGDKRCKSTLHGNIAGGSSDYFERPTSGTIKAALLLYKDNIHVKILDNNTDCFGKTMGNTFVDEMVQSTAAQDLQNLVSLFQLSG |
| 410 | Unigene0001104 | 33 | 42017 | 4.7 | MAEAAAVDYSLNNPDTLTKYKTAAQIGQKVLEAVSGWITEGAKVVELCQRGDKLLDEEIAKVYKGKKIAKGISHPVTVSPSDYVTPYTPLVSDAEEAETTLKAGEPVKIQLGAQIDGFGAIVCDTVFARAKGDSEKTLSGRQADLALATHYANELLLRLVAPPGLVATGTEEEQKKAQSQKPYSQAKITQLLEKVVKSYDCNLVENTTCWLFDRNEIEGKKKIILAPGEGVKGEGLAEIGEVWGLEIGVSTGNGKVKSLPKRTTLHRRTATTYGLKRPSSRATLSEIQKKFGTFPFSLRQLDSEKDAKVGVIECVRGGVVRAYEPAGSTDGEPVSRLFETVAITKNGVQKLSAPPAVDISQWQSDKKITDEEVLKILELPLNKNST |
| 411 | Unigene0008139 | 33 | 134742 | 0.7 | MDAYPPDYVQHNLPLVLLSGLGQLDHDSAKSPPARQESGARISTESPECRSDQAKRLLQAFRSLDGTDSVWNSSAQPGPTSSIRFKIKAVGRTYSLPPRKAAPLPQSPGAEGPHIARNTELHSPLSPLSPGSPVFPDGVFTPLWLHKHQEQVPCLVLAAFPISANEASQDERLRGDINAIRNSLTRSMFKTRFAVLLISDRSILEAPDLEDRLGMIRRATNLDSKSGLFFMPPMGSDAEIETFVQDMMKVLQPQCVEYYRDLTKHARRKKARGGPPPLSHTPVGGASQATTQSGWNVRYEFKQAVFAEYRQEMDVAERHFAAALEELFSTEGGVLETTTNWSPRWNEARLLSDVTALRILRCHLWLGQTTGAVQTWMNYKRRMKDLIDRRGKGSGTYSWAAWEGRWAVIMSQLIQRAEIPSLQRPPPESSSELEVFAEPERVALATERLRPWDLLHHSGYWLRLLSQGIRARWERALAIPDEDRLSPGQSPASSVANRWKTYDAYLVPDPHEEMSVSAEGGHDHVAELGTACMQASEEFAARQQLRMSEQIRLELAEDLISAGQRSDAIKILMEIWKNCTWREDEWHIPFARLLRMLLECARHEATEQYASIIPAVTWELLAIASDGPSDTPLDLMNCAPAPFENGIIALTNRDGDRLCPFTVSFAFATQSSYVGESQQCQLTLMLNTATMAKPIRLSSVHIRLGKKHVRVNHESGSTQSSTAPELTILPSFAEHDSGSSDVTADLLFSPQSTKTLQFEIMLREAQVCRIDEAVIYVKEPSFNLEHVLGDHCVLPLNRWQVRSDEKVDSLLLPHVDPRAITVLPKPPKLQVSLLEPRKQYFTDENIRLRVNVFNEETDCISGTITPALVGTTEDPATSLKWASDDASGAPAAISDLDASSSITKELFLQAPTEASSIALTLEVQYALKSDLDTLLSKVVTFDLSFVPAFEATFTFLPRWNPDPWPSCFSNTSTDIPTGIKQQWGFCAQLSSLTDQDVLLRTLEVVVHEAHGDVLCTIAEATRTSEVLLPAQARHMSEFELTSQKISLDDRRPSHIDLSLIVTWSVGTATETAETIIAVPRLTVPTSEPRVLCTIERGQTGASPILRYHLENPSTHYLTFALTMEANDDFGFSGPKYRALSLTPLSRSQVEYRLQLHNDTEGGSWISPNLQVVDSYYNKTLRVQPGDAHVKLAADKTVQAWTG |
| 411 | Unigene0000549 | 33 | 134742 | 0.7 | MDAYPPDYVQHNLPLVLLSGLGQLDHDSAKSPPARQESGARISTESPECRSDQAKRLLQAFRSLDGTDSVWNSSAQPGPTSSIRFKIKAVGRTYSLPPRKAAPLPQSPGAEGPHIARNTELHSPLSPLSPGSPVFPDGVFTPLWLHKHQEQVPCLVLAAFPISANEASQDERLRGDINAIRNSLTRSMFKTRFAVLLISDRSILEAPDLEDRLGMIRRATNLDSKSGLFFMPPMGSDAEIETFVQDMMKVLQPQCVEYYRDLTKHARRKKARGGPPPLSHTPVGGASQATTQSGWNVRYEFKQAVFAEYRQEMDVAERHFAAALEELFSTEGGVLETTTNWSPRWNEARLLSDVTALRILRCHLWLGQTTGAVQTWMNYKRRMKDLIDRRGKGSGTYSWAAWEGRWAVIMSQLIQRAEIPSLQRPPPESSSELEVFAEPERVALATERLRPWDLLHHSGYWLRLLSQGIRARWERALAIPDEDRLSPGQSPASSVANRWKTYDAYLVPDPHEEMSVSAEGGHDHVAELGTACMQASEEFAARQQLRMSEQIRLELAEDLISAGQRSDAIKILMEIWKNCTWREDEWHIPFARLLRMLLECARHEATEQYASIIPAVTWELLAIASDGPSDTPLDLMNCAPAPFENGIIALTNRDGDRLCPFTVSFAFATQSSYVGESQQCQLTLMLNTATMAKPIRLSSVHIRLGKKHVRVNHESGSTQSSTAPELTILPSFAEHDSGSSDVTADLLFSPQSTKTLQFEIMLREAQVCRIDEAVIYVKEPSFNLEHVLGDHCVLPLNRWQVRSDEKVDSLLLPHVDPRAITVLPKPPKLQVSLLEPRKQYFTDENIRLRVNVFNEETDCISGTITPALVGTTEDPATSLKWASDDASGAPAAISDLDASSSITKELFLQAPTEASSIALTLEVQYALKSDLDTLLSKVVTFDLSFVPAFEATFTFLPRWNPDPWPSCFSNTSTDIPTGIKQQWGFCAQLSSLTDQDVLLRTLEVVVHEAHGDVLCTIAEATRTSEVLLPAQARHMSEFELTSQKISLDDRRPSHIDLSLIVTWSVGTATETAETIIAVPRLTVPTSEPRVLCTIERGQTGASPILRYHLENPSTHYLTFALTMEANDDFGFSGPKYRALSLTPLSRSQVEYRLQLHNDTEGGSWISPNLQVVDSYYNKTLRVQPGDAHVKLAADKTVQAWTG |
| 412 | Unigene0012818 | 33 | 33133 | 4 | IAIVGASGHLGKEVAGALLSSTFRQSYGNVLLLTRDRGDSQPAAPPAGAEIRRFSNDRLADALQGVDVLISTVGPAGHSFKDSLVDAIAQSNVKLYIPSEFGVDHTIHDFSQPEWDHKKHHDGLVKARLQKTKVCRIYDGLFLEDSLGPWFGMDVKRGVFHSVGPADSPISFTTKADVGKVVAQLARMPYDEVPPHLRISGDALSMRQIAKVLEDAGSSPIAVKEIELNDYKRRAIAENVPDPMKYLRFLMGEGKINHTASGLGNDNELVNPGQSLWTWKTVSDYAKEVKGRPWADYDWPAE |
| 413 | Unigene0006378 | 32 | 23286 | 9.9 | MAIKHNQQIQYNHFRKDWQRRVRVHFDQPGRKQRRRNARQEKAAKVAPRPVDRLRPVVRCPTIKYNRRVRAGRGFSLAELKEAGIPRKLAPTIGIAVDPRRQNLSDESLAANVARLKEYKQRLVLFPRKLKAQKNGDASKDEVDAAKQIGETAHDGKVKHTKQAFPIDNKVVVKEGKISDYPQTENAYRVLRTARSDARLQG |
| 413 | Unigene0006376 | 32 | 24928 | 9.2 | FRKDWQRRVRVHFDQVIPHSSUMLARLQCTAUTUSSDLEFSIUPGRKQRRRNARQEKAAKVAPRPVDRLRPVVRCPTIKYNRRVRAGRGFSLAELKEAGIPRKLAPTIGIAVDPRRQNLSDESLAANVARLKEYKQRLVLFPRKLKAQKNGDASKDEVDAAKQIGETAHDGKVKHTKQAFPIDNKVVVKEGKISDYPQTENAYRVLRTARSDARLQG |
| 413 | Unigene0006377 | 32 | 21680 | 10.6 | FRKDWQRRVRVHFDQPGRKQRRRNARQEKAAKVAPRPVDRLRPVVRCPTIKYNRRVRAGRGFSLAELKEAGIPRKLAPTIGIAVDPRRQNLSDESLAANVARLKEYKQRLVLFPRKLKAQKNGDASKDEVDAAKQIGETAHDGKVKHTKQAFPIDNKVVVKEGKISDYPQTENAYRVLRTARSDARLQG |
| 414 | Unigene0002226 | 32 | 27208 | 4 | MARQFFVGGNFKMNGTISKIKEIVSHLNEAKVDPNTEVVISPPSLYLLLTREHLRKGIEVAAQNVFDKPNGAFTGEISVEQLKDSNITWTILGHSERRTIIQETSEFIASKTKSALDGGLGVILCCGETLEQREANKTMDVVLAQLKAVSEQVKDWSKIVVAYEPVWAIGTGKVATNEQAQEVHRGIREWIAKELGNEAAEKTRIIYGGSVSEKNCKDLAKEADIDGFLVGGASLKPAFVDIINAKQ |
| 415 | Unigene0000840 | 32 | 26696 | 4.4 | NQRKGRGSIFTANTRLNKAPAKFRTHDFAERQGYIRGVVKEIVHDAGRGAPLAKVVFRDPYRYKLHTETFIANEGMYTGQFIYAGKNASLTIGNVLPLGSVPEGTVVSNVEEKVGDRGALGRTSGNYVTVIGHNPDEGKTRVKLPSGAKKVVKSSVRGMIGIVAGGGRTDKPLLKASRAKHKFAVKRNSWPKTRGVAMNPVDHPHGGGNHQHIGKASTISRYAAQGQKAGLIAARRTGLLRGTQKTKD |
| 416 | Unigene0003204 | 32 | 20605 | 4.4 | MTSLQEIKKNCRKVVCIGRNYADHVTELNNQRPKQPFFFLKPPSSILLPDEGPILRPKGVTLHFEIELGIVIGKTVKDLSENDEAGWKDAIGSYITGIDMTGRNVQEEAKKRGLPWSIAKGFDTFLPIAGPVRKEDIKDPHNVELYLKVNGETKQNDNTELMLFRIGRQLADISKVMTLEEG |
| 417 | Unigene0009401 | 32 | 18818 | 4.8 | CRFLEHCLSGVRTLUGSRLTGQLUGKLFVRSDSKIFRFQNGKSESLFLQRKNPRRIAWTVLCRRARRKGISEEQAKTRRRKQVKAQRAVVGASLDVIKERRSQRPEARSAARKQAIEDGKKKRAATESAKKAEKAKGAAKSAAGQTTGRVVGKQGAKGSAPKVKATTR |
| 417 | Unigene0009402 | 32 | 17963 | 5 | MRTYDDTFSGQKIYPGKGKLFVRSDSKIFRFQNGKSESLFLQRKNPRRIAWTVLCRRARRKGISEEQAKTRRRKQVKAQRAVVGASLDVIKERRSQRPEARSAARKQAIEDGKKKRAATESAKKAEKAKGAAKSAAGQTTGRVVGKQGAKGSAPKVKATTR |
| 418 | Unigene0005156 | 32 | 27978 | 3.2 | MHIESIPMWEGTGNNYAYLVTDDKTKEGVIIDPANPPEVLPSLKKAVDGGVKLTNIINTHHHHDHAGGNKEILKAYKLPIIGGKDCDQVTKTPGHGETFTIGEGIKVKALHTPCHTQDSICYLFEDGNDRAVFTGDTLFIGGCGRFFEGTAEEMDAALNKTLAALPDDTKVFPGHEYTKGNVKFGIKVLESEPVKKLESFANSNKQTQGKFTIGDEKQHNVFMRLDDPTVQKFTGKTERVDVMAALREAKNNS |
| 418 | Unigene0005157 | 32 | 27978 | 3.2 | MHIESIPMWEGTGNNYAYLVTDDKTKEGVIIDPANPPEVLPSLKKAVDGGVKLTNIINTHHHHDHAGGNKEILKAYKLPIIGGKDCDQVTKTPGHGETFTIGEGIKVKALHTPCHTQDSICYLFEDGNDRAVFTGDTLFIGGCGRFFEGTAEEMDAALNKTLAALPDDTKVFPGHEYTKGNVKFGIKVLESEPVKKLESFANSNKQTQGKFTIGDEKQHNVFMRLDDPTVQKFTGKTERVDVMAALREAKNNS |
| 419 | Unigene0008373 | 31 | 9248 | 11.5 | SAVRITRALPKTPVASRSFSVAACRMAGGDTGATRSGGAAASDSFTKREEANEAAFIKKAEREKLEALKKKIADSEAQLAKDRKKAD |
| 419 | Unigene0008372 | 31 | 9248 | 11.5 | SAVRITRALPKTPVASRSFSVAACRMAGGDTGATRSGGAAASDSFTKREEANEAAFIKKAEREKLEALKKKIADSEAQLAKDRKKAD |
| 420 | Unigene0011599 | 31 | 13990 | 23 | PGTFEISNFVFGCTVGCDYNFDLTVSGSAENHPAIEPTYVKCSGNLENTDYVQCGDVSETQRLYAYIEKANNKLHLQYEVQNYNTGAIYRYYGEEIVYSATGEDADKQEECFEVKENRATGV |
| 421 | Unigene0008602 | 31 | 25193 | 9.2 | MSAVKNIVKALESFTSCDVADALTKLKVPHGGFLPGLTMWSPRRQEGPTKIIGPAYTVKYVRKNYENDPKPKGHYIDTVPEGSILFISAPPKMINACYGGLMSTRAQYLGAAGTIIDGRFRDLQEHRDLNFPVFAKDVGTTAPAEVARVSEINGPVRFNSEEQDCYINPGDILIADLNGVVCLPKDLAEQALDLIASQVEADENVAKDIRNGVLVGESMKKHRANVKQP |
| 422 | Unigene0003357 | 30 | 18896 | 7.8 | MLIPKEDRKKIHEYLFKEGVAVAKKDFNLPKHGEIDTKNLYVIKACQSLTSRGYLKTQFSWQYYYYTLTPEGLDYLREWLHLPAEIVPQTHVKQQRSHAPPRGMLGGDDRERRGGGRGGPRGDREGGYRRRDFGDRENKDGAAPGGFNPEFRGGFGRGRGAPPAQS |
| 423 | Unigene0014457 | 30 | 31464 | 3.5 | MDALVAQYSRPAYQNEGYSEEDQQQLALDLQPQLSLNFALPPVANNAAWLRAMTDDHTNPQCPIKIAHGTTTLAFRFKGGIIVATDSRATAGNWIASQTVKKVIEINPVLLGTMAGGAADCQYWLAYLGMQCRLHELRHKRRISVAAASKILANLVYSYKGMGLSMGTMCAGVTPSEGPALYYIDSDGTRLPGNLFCVGSGQTFAYGVLDAEYKYDLEDEEALELGKRSILAATHRDAFSGGFINLYHVKEEGWVKHGFMDTNPIFWKTKLEKGEFSNVTANLD |
| 424 | Unigene0000116 | 30 | 59768 | 1.5 | DVITKTHVSQNSRNMTQFDRIDDEDLDYHGHLKSSKEDDRGFTRNDQKDMSRMGKKQELRRNFRGLSTIAFVVILQGTWEVLLAASYQGMYSGGLAGLFWSYVWTFCGFSLVVVSLAEMASMAPTSGGQYHWVSEFAPAKYQKLLSYFTGWMSTLSWQAGTASGPFLVGTMIQSVAAINNTSYTGTNWQGTLCVWAITILVLFANVYGGNAMPVFQNLMLILHVFGFLTIIVCIWVLAPRNAASVVFTEFYNGGDWQTMGLALMVGQISAIYACICSDAAAHMSEEIKDASVTVPRAMIGSYVLNGGLGIIFLVTFLFSVVDLEGALEADYPFLYVFQNAFSIQAVNALSSIVIILIFAGTLSYNLSTSRQTWSFARDNGLPFSGWISKVNTKLEVPANAVIVTCAFTIVLSFINFGSDVAFNAIISLNLVSLMITYQVSIACVLYRRLYQPELLPKARWSLGKYGVAINIGGLAYSSFAFFWCFWPNIRNPSLADFNWSVLMFVVVGLIAAIDWVVRARHVYTGPVVLVEGFRDH |
| 425 | Unigene0008325 | 30 | 40021 | 6.5 | AAPHIARDGLSCREINIPVDVSANNRDLPDTLDLSSLLSLSVLNDLVAGLGSTLVSGNYSIAARYCEPSTKVESRNKTLQVLVHGIQYDRNYWFGLAAPGTKAGQDEYSWAVYAANEGYATLSIDRLCTGNSSRPLSLTQCQAPLEAETLHAIIEAARSGTLPDVQQSFDKIVYTGHSYGSLIGNALALQHPEDIDTYVFTGFSLDALLGATGFALLPGFLPAAVVAPERFGSDDLGYLLATNEEGAKKVFYYGDYDENIFSEDFAGRQAVPLSEIATVPLGQLPAPEYQGSVFVLNGNEDAIFCVGLLDTLLGVPGDCSNGFVSRVKNGYPKAKAFGSHITANTGHAMNNHKTAHESFKAAHDWLAQQGF |
| 426 | Unigene0013408 | 30 | 138829 | 0.9 | MGATTNGEVNGTSRGIRIAIDRGGTFTDCVGNPGTGKMEDDVLIKLLSVDPQNYDDAPLEGIRRLLEKFTGQDIPRGQPLDTSKIESIRMGTTVATNALLERKGEDIAMVVTKGFKDCLEIGNQSRPNIFDLAIRKPEVLYKKVVEVDERVTLEDYAEDPERHMTEAEAVDGAKSNAELVKGLSGETVRILQRPDEERIREQLQEVYESGLKSIAVCLMHGYTYPQHEALVGKIARDIGFEHVSLSSELMPMIKLVPRATSACADAYLTPAIRKYIDGFQKGFEGGLGTESVKKESGSKGARCEFMQSDGGLVDVDGFSGLRAILSGPAGGVVGYALTSYDPETQIPVIGFDMGGTSTDVSRYGNGRYDHVFETTTAGVTIQSPQLDINTVAAGGGSRLFFRNGLFVVGPESASAHPGPACYRKGGPLTITDANLLLGRLLPDFFPKIFGKNEDEGLDEQASETAFKELTEKINKENAAGNKEKEMNMDEVAYGFLKIANETMTRPIRSLTEARGHDTSKHRLATFGGAGGQHAVAIAEALGITQILVHRYSSVLSAYGMALADVVDEQQEPESRIWSDKDQETRQYLQDKMAELKKKSTATLKDQGFSEDQIHFEEYLNMRYRGTESALMIVRPSKQEASKDYDGDDWAFGQAFIKQHEQEFGFTLPDRDIIIDDVRARGIGKTFEGLEKTVDQQLKEVKPKDLQKGDKTYGSKSVYFEGGRQDTAIYKLEDLAVGDRIKGPAIIADGTQTIVVTPNASALLLDTHVVINLGEVDAQEKKVDTKNVDPIMLSIFAHRFMAIAEQMGRALQKTSVSTNVKERLDYSCALFDADGGLVANAPHLPVHLGSMSTCVRKQAEIWKGKLKKGDVLVSNHPMFGGTHLPDITVITPTFSGDKIVFYVASRAHHADIGGILPGSMPPHSRELFQEGAAIKTEKLVSEGHFNEERITELLLTEPAQYPGCSGTRCLADSLNDLKAQIAANQKGINLISTLIEDYTEDVVQFYMHSIQDNAELSVRNLLKDVSSRFEGQDLSAIDFMDDGSPIQLKITIDAEKGEAVFDFTGTGPEVYANWNAPEAVTYSAIIYCLRCLIKEDIPLNQGCLKPVKVIIPPKSFLSPSGTAAVVGGNVLTSQRVTDVVLKAFRACAASQGDTNNLTFGFGGNVSGQEAVKGFGYYETIAGGSGAGPTWDGTNGVHTHMTNTRITDAEVFERRYPVVLREFSLREGSHGKGQHKGGDGVIRDIEFRIPVQVSILSERRVYHPYGL |
| 427 | Unigene0012498 | 30 | 85255 | 0.9 | ATAPGETTAAPPPTTRRTTRSQSAEPPQIEQTNAATAKNGEDASNPSAKRGGRQTKTKDVVEELPTVDETSLFVGEDADAEAEEDVDARLNEQLNGTPRRNSIGSAFSGTTAKTSFSQDEINKLDHEVICDVLPDILSAAERLTQLLVPNGANTPPETWKEIKRTGSKHNKLFTTRWESLRIHKPSVTSVEYIEPRYVLRALLNVASMAEVQPAVWRPDNVLYKINMALMLRTVLVQCDPADWTEEATGALESLDMAFPACIAGGEFKFAALGLWLELAAHVTIHRLEAAIESEPNFSAHEEIDNVFYDADSEFKHAKTLGLASANEEEFAQAMEMVNQRISVLKEPFGQDDGKDATAANGYLKAQFRWDDFRSHALRYFDARIAEVNRRIEAAGGVQTIVSGLEEEVERRASEKIAEQMKGTYKKPGTPRQSLGGKSAIALLKGHEKRISEQVPAAGAPAPVASMHPTNDTLQDSDVITHDFATGQRSEVDQDSFEGPTEVQSTAQQALASFAATQKQNARKAKARLLDRQDNAVRVSSEMYMDTQTQASAATGKRSRAQMEEEVPEDFDPTQDDGFENDTRNHNNAAQRRAEVAFEGRAPQRFPTIEPRDSADAGPLTYEWISGTAPSPSKRQRKNPGSAIPPPRPAPAPEDGELPMSEFYKQAKLDAKYNRVLAPAAPVRTERTPWSDEEEAALIDLIVDHVADDEKIGYSNLKAIDQSLEESGEAKLSRRSAEDIRFKARNMKLTLLLAERKLPRNWDKVILDKKAID |
| 428 | Unigene0012620 | 30 | 16728 | 6.7 | MPTRLTKTRKHRGHVSAGHGRVGKHRKHPGGRGLAGGQHHHRTNMDKYHPGYFGKVGMRYFHKLQNQFWKPVINLDKLWSLVPQEQREKALAGGKSDTAPVLDLLPLGYSKVLGKGRIPEVPLIVRARYFSKDAERKIKEAGGVVQLVA |
| 429 | Unigene0006620 | 30 | 43006 | 1.8 | KTTELSQFQVGHTSPSIKLVASTISEVESLAPWLLESMQLGKQVSILYGLPISPSAIPRLAGVTRILGPGTVGLFVDHPDHVNLIEQVSDDIWPGRVPVWVTIDVGYHREGVPAGSKQLADLAQALKGAKKAVLAGIYTHMGHSYSASSPEDALNFMSQEILGSEEGATSFLNSVGHDVESSSKVVLSLGATPTATAIQNLTEGSEAVQQYRSTIERVQQSFDVEFHAGVYPVMDMQQLATRARPQTSARTGQSLLSFRDLGFRVLTEVASTYFDRSDKPEALIAAGGIVLGREPCKSYPGWAVVTPWPAASGQTYDPEGDKTGWIVGRIAQEHGILTWEGPKDNFRTLGVGDKLLLWPNHACIAGVNFGWYLVVDSDKANPDRIEDVWIRWRGW |
| 429 | Unigene0006619 | 30 | 50304 | 1.5 | MTTPTSYLYPTPPAAALKLQFVGKEVKDIQAPAAILDVAVIRRNCKLMLDTAEKLNVGFRAHVKTHKTTELSQFQVGHTSPSIKLVASTISEVESLAPWLLESMQLGKQVSILYGLPISPSAIPRLAGVTRILGPGTVGLFVDHPDHVNLIEQVSDDIWPGRVPVWVTIDVGYHREGVPAGSKQLADLAQALKGAKKAVLAGIYTHMGHSYSASSPEDALNFMSQEILGSEEGATSFLNSVGHDVESSSKVVLSLGATPTATAIQNLTEGSEAVQQYRSTIERVQQSFDVEFHAGVYPVMDMQQLATRARPQTSARTGQSLLSFRDLGFRVLTEVASTYFDRSDKPEALIAAGGIVLGREPCKSYPGWAVVTPWPAASGQTYDPEGDKTGWIVGRIAQEHGILTWEGPKDNFRTLGVGDKLLLWPNHACIAGVNFGWYLVVDSDKANPDRIEDVWIRWRGW |
| 430 | Unigene0012166 | 30 | 68653 | 1.4 | LPVFTWRLRQQLHTFTSAAYKHIQQIDQHLLTCARLELQHGAQKGSTNPRPNNHNQQHDNHLKPKLDYNKDTQLPAAAPPPSMKVSLLAAIPVIGAAFAAPAATGDSKAVSVRIHSSDGTESTHSITLGEYVQVEGKDIDRIDLTSSASCTDGPLTCQPTWYFSSSGPIVGLGFTASEPGYPRSTFGNGEVNSVGLTCSCSPLLLSARDDEASSVLIKITNVDGTIRSGPLVLDEYKEFSSTDIAEVSIGGWSPGLNLLNIHCQPTWHFSDSGPIVGLDFAGNEAGTPDRVFGNGRVDTVGLRCSSSPSLMAREDKDGYITINVLGGHGVKRVDWIVAGQVREYNAIAFKDTLVSVEIDSADPDSEGVTCQPTWYFSDTGPVVGPTIAPSQPGSPDQTFGNGHCLKVSIKCSRSTQLVARADDSKPSVDIVFRESDGKSGSVTAPVGVGAGVNATGKDIVTLELGTGHGVQSSKVVCLPVWLFDTGASQAGSPLKKDRAAFLSDTKAGYAIMTKDRPKVTRVEVFCDVSAMKPRDAVPTIDITAHESKGKNSTASIHFGQAADLRGKNITSVEIKGADASVDIEKINCQPIWYINRGSGQLGYTFNSTNPGNPGYFLHGNDAVTRVAVTCAWRATSADR |
| 431 | Unigene0009803 | 30 | 39592 | 3.2 | MCRWFAYISPSEPTLLADVLITPSNAITKQCSEHYLPYLLPHGEEKELDDAPDELLRMRNSLLNMDGLGVAWYTPSAAAYTNKKSGMRPALYKSQSPPTNDFNFRSLCENTESNCLFAHIRATSGSVVTPVNSHPFTFGRHTFMHNGVISDFTRVRRDITDLLAYDAYENVRGSTDSEHAAAMYMTFLCGGPEKCTKDAWEKTYPVDAMRTALQSTVISILEQQAKELGDKKTPNSLNFCATDGTQLVACRFRNSFSQQPPSLYWSEFAGRTLNRKYPGDPDGLRENTDQKVGEEERIGKHTIVASEPTTYDEKEWHLIGRNCILLVDEKGVETEVPLEYDEQKLNA |
| 432 | Unigene0003708 | 30 | 7899 | 9.9 | MPYKINVSLQKGADAQHYDTAKKNVEDQGGKIVNEFKLIKGFTAEFPDDKVHSLSSNEHINVEKDGVVTTQ |
| 433 | Unigene0000702 | 30 | 50904 | 1.8 | MREVISLNVGQAGCQIANSCWELYCLEHGIQPDGYLTEERKAADPDQGFSTFFSETGQGKYVPRTIYCDLEPNVVDEVRTGTYRSLFHPELMITGKEDASNNYARGHYTVGKELIDQVLDKVRHVADNCSGLQGFLVFHSFGGGTGSGFGALLMERLSVDYGKKCKLEFCVYPAPQVATSVVEPYNSILTTHTTLEHSDCSFMVDNEAIYDICRRNLGIERPNYENLNRLIAQVVSSITASLRFDGSLNVDLNEFQTNLVPYPRIHFPLVAYAPIVSAAKAAHEANSVQEISMSCFEPNSQMVKCDPRNGKYMATCLLYRGDVVPKDVHQAVATLKTKRTIQFVDWCPTGFKIGICYQPPQMVPNGDLAKVNRAVCMLSNTTAIAEAWSALSHKFDLMYSKRAFVHWYVGEGMEEGEFSEAREDLAALERDYEEVAADSAEGEEGAEAEY |
| 434 | Unigene0003166 | 30 | 29374 | 3.1 | MAVGKNKRLSKGKKGLKKRTDPFARKDWFSVKAPSTFNIRDVGKTLVNRSTGLKNANDALKGRIFEVSLADLQKDEDHAFRKVKLRVDEVQGKNCLTNFHGLDFTSDKLRSLVRKWQTLIEANVVVKTTDDYLLRLFCIAFTKRRPNQIKKTTYARSSQIRAIRKKMTDIMQREAQSCTLSQLTTKLIPEVIGREIEKSTQGIYPLQNVHVRKVKLLKSPKFDLGNLLSLHGESSTDDAGQKVEGARDFKETVLESV |
| 435 | Unigene0006313 | 29 | 48708 | 1.8 | MERVHQIASHFASGQTAQQAMAPKDVLIVGSGVFGLGTAYALAQREDFKKTKITVLERTQFPAQDASSVDSSRIIRADYEDVLYAQLMEEAMPHWKGEFGEDGRFSQSGLAIMSNGRNESAETFLEAALKNVTEKLGLQIGKDITVFDNEADARKIMGTTGGLIGTRGYVNWTSGWAHAENGLKFLRKKVEALNRVHFQTAEVQRLLFDSSYTSVLGVQLADNSTLTADLTILATGAWTPKLLDTRGICSATGQVLTFVDITDEEQARLTQKGQPVILRENDGLFIIPPVENKLKIARHGYGYANFVTIPHPEKPASSGEKITVSIPKTKLDDPDMDIPAEGKKVCRDFLRDAVPALADRPFSSTRICWYTDTPSGDWLITYHPKYKGLFVATGGSGHGYKFLPTIGERIVDVIQGRERDELGRQLAERWKWPEKREK |
| 436 | Unigene0011141 | 29 | 53455 | 1.6 | MKGFLGLSAAAVVAASPVVVDSIHNDAAPIISSTNAKEIPNSYMIKFKKHVTTNLAADHHDWVQDLHFSTQSAKTELRKRSQTPMVDEIFHGLKHTYNIAGSLLGYSGHFDEETIEQIRRHPDVEMVERDQEVHTLGGEPEEIEKNAPWGLARISHRDGLSFGTFNKYLYADHGGEGVDVYVIDTGTNVDHVDFEGRASWGKTIPSGDADEDGNGHGTHCSGTVAGKKYGVAKKAHVKAVKVLRSNGSGSMSDVVKGVEYAAESHAEQVSIAKKGKRKGFKGSTANMSLGGGKSPLLDQAVNAAVDAGIHFAVAAGNDNADSCNYSPAAAENAVTVGASTLADERAYFSNYGKCNDIFAPGLNIQSTWIGSKHAINTISGTSMASPHIAGLLAYLLSLQPAKDSAYAVADITPKKLKANLISIATEDALSDVPSNTKNILAWNGGGISNYSDIVEQGSYKVHRVAETISEKATDLSEKMAELEHKIEADIKDFLRDIKS |
| 437 | Unigene0003958 | 29 | 34266 | 5.8 | GHDDVASADVSDTRGVRTTAQFSSKIFAKDGGKDHLWASDNLSNYSFSQDKQNFKADGCSMDLSEDGKSYHIKSNTNKTAIVDVKWTQSAPGFVVGKNGKTLFGTDKEKPWGSMRHAFWPRCRVEGTIMTKDGPFDVKGTGIFIHALQGMKPHFAAAKWNFINYQSPNYSAIMMEFTTPPSYGSTSVNVGGIAKDGELIFASSEPGVKVEHTGIKGDEENDWPEPSAVAATWDGTTKDGKAVHAELKNDLSRTDRVDVMGEVPKFVKQIVAGAAGTKPYIYQYNPKLKLQLKIGDEVIEDEGHLFMEATFIS |
| 437 | Unigene0003959 | 29 | 41204 | 4.8 | MNWLKKSVGLTDHHYGPEGVHSVAQQAKETPYTELTKNDLAWEVMSGTNVETKTFYVATDSGHIAMVQVIYSDVMGVRTTAQFSSKIFAKDGGKDHLWASDNLSNYSFSQDKQNFKADGCSMDLSEDGKSYHIKSNTNKTAIVDVKWTQSAPGFVVGKNGKTLFGTDKEKPWGSMRHAFWPRCRVEGTIMTKDGPFDVKGTGIFIHALQGMKPHFAAAKWNFINYQSPNYSAIMMEFTTPPSYGSTSVNVGGIAKDGELIFASSEPGVKVEHTGIKGDEENDWPEPSAVAATWDGTTKDGKAVHAELKNDLSRTDRVDVMGEVPKFVKQIVAGAAGTKPYIYQYNPKLKLQLKIGDEVIEDEGHLFMEATFIS |
| 438 | Unigene0010175 | 29 | 28224 | 6.4 | MGKVKYILLDCDNTLCQSERLAFEACADLTNEVLKKYSVDATYTTDSLLEDFVGHNFRNMLIGLQKKHNFSMPQDEVDKYVDMELDRVTAKLSEKCVECPGVTEQLEWAKAQGYPMSVVSTSAKPRVVASLEKCNLMRFFSDELVFSAATSMPDGPSSKPDPKIYLYACKQLGVKPEECLTVEDSKSGATAGMRAGIPLIGYVGIYGLEDGAEKEKQMAETLTEVTKADFIMRDWKEFPEAVKHIESKL |
| 439 | Unigene0004517 | 29 | 29777 | 2.5 | MSDISIDGFFSLDGKVAIVTGGSRGLGLHTATAFLLAGAKTVFISARKAGGEQGIDQAVDKLNKLCSSKNLKGRALGLPANVAQEEDIKRLVKEVQKHESKLDILVANAGATWGGPFEPTPDWSSQKILDLNVRGVFNLARLFAPMLAAAGKPEDPSRIVIMSSVAGTNVSHVGDNGTIMYSASKAAAHHLGRNMAVELGPRNITVNTVAPGFFPSKLASGLIEILGGEEELSRSNPRKRLGEPSDIAGVMLFLCSKAANYVSGEYISVDGGARLGSGRLTKL |
| 440 | Unigene0000574 | 29 | 65234 | 1.4 | MGSYNFTWSHPADEVYVTGTFDNWSKSVKLDKKDQLHEKLVNLPSTEKVYYKFVADGNWSHDHTAKTETDHEGNLNNVLLPEDIQPVNSMAHNLSSVAPGATTASLAGQQPLEKEKDKESGVAASAPGAFPETPAADSKEQEQTFSVNPIPASAGIGNPISVPAGQQLPDQSTFNNNTIASTVRDDPLLKSSTDDQTVSVAPLPATSGIGNPITLAPGEKVPDSSTFTTNTIDGNVKLDKASYENSGASVPAVPDTSADGSNATASAAIVGGLGPQTTNIIPESSLGMGKDVPAPIEGQAVNSVGPASTTNQLAGQVPLEPRGVPEVVKESQQEAGVAPEASANPEAVAEKKELEAELKDKIGKPEDQTSSAIGGAFGGAAAGAGAAALGAAAVARQKTHEVTGTDPVSVLPASVQKTVDENTKPSGGDTTRPAADAVPTEVKESQKEAHVAPEASANPEAVQEKADVEKELLQKVPESQAGGDPAPTVTATTTAVAPAAEMTSASGAPQLGDPTAGVAALSLDDKSKGLNAPATSQAQPNQPMDSRDVSPMSKPNPATATENQTAPVVTTGVNSGEAPAASKPIGTPRTGASPSTPAKRNSIMERFKGGKEGTPDSAKTEASSTKKKGRFSRLIEKLK |
| 441 | Unigene0008058 | 29 | 57698 | 2.9 | LFGLANRVHGDIIVPADASKNISWPYHTFKTVNFTPPVIEIFGTPSSDSAKDGYLFFAPDGGTAFQAAPIIATSNGDLIWNGPKGKAFGFDVQQYKDEDVLVYWNGTAFPEPVGRGHGSIHILNKHYEEIAKVSLPGNFVDLEEGQTFESNIDLHEMYLTEKGTILLLGNNVTQADLRSVGGGEKGWVAEAQVYEIDVATNEVLFSWKSLEHLEELPFSASVYPLGSEEFDGSEQKKAWGYFHINSVAPLGNGGYILSSRYLSSAIALDKTGKVIWRVQGIEGNAFTLGEGADFRYQHHIRVVNETDDCVVIRLHDNHNCPIDNNTVPASGKFLEINLKDKHVSLLGPKYYNASGPIFPTAQGSFSEMQNGNFFTGNGWIPVLEEFSPNGTTVATYQFGNATLRPVGGYFSGGRGTLSYRGFKRPWVGCPKNSKPQILAEYIGAMDAPKGTRVHISWNGATEVKEWELWGGIASVSHIQNVEKNGFETVVEIADVQFVKAKAILKHGTSGHCEDGVESDLVFTRA |
| 442 | Unigene0002700 | 29 | 86321 | 2.3 | LRRDLEKRSMMAGERTPLIQTVPVAEERDRYPHARVRRICTTVISSVILLAALVFLGFAVTGNGDILTGKPRTQPEHFATAELPHPAWPQSHGLGYKELKKVLLETPDAKKAREWSQYYTAGPHLAGKNESQAQWTKDLWKQFGVDHAEVVSYDVYINYPLGHRLALLQDGEVAFEATLEEDVLEEDPTTNLTDRVPVFHGYSADGDVTGQFVFANYGTWDDYEDLINAGIDLKGKIIIAKYGRNFRGLKVARASQLGAIGVVMYSDPGDDGEITEENGYKTYPEGPARNPSSVQRGSVQYLSFAPGDPTTIGYPSKPGVPRQSVEGHIPDIPSIPVSYKEALPILKALNGHGPKASFFSKYWQGGGLGYKGVDYNIGPSPESLTINLVNRQDYVTTPLWNAIGVINGTVSDEVVVIGNHRDAWIAGGAGDPNSGSAALNEVIRSFGKALEAGWRPHRTIVFASWDGEEYGLIGSTEWVEEYLPWLSASTVAYINVDVGAQGPNFKASAAPVLNKAIYEVTSQVQSPNQTVKGTTVRDTWNGHISTMGSGSDFTAFQDFAGISSIDIGFGNGPNDAVYHYHSNYDSFYWMDNYGDPGFKYHITIAKIISLLAAKLVEEPIVQFNATDYATGVEKYLDSVKNVAEEQQWDLSTVKDAFKQLDEAASYFHDASVEFDASATRLEEHLSDVTISDKRKKAIYQAVKKTNKKYKFLDRQFLHAGGLDERSWFKHVVFAPGRWTGYAGATFPGLVESLEDHDKKNLQKWADIIAARIYAAADLLLEK |
| 443 | Unigene0008387 | 29 | 67502 | 1.7 | MGIYTKLPEEIRQVDVIVAGGGTAGCVIASRLAEADPELSILVIEQGMNNHNIPEVVYPALFPQNLKEGSKTALFWQGNFAPQLGNRAPVVPSGGTLGGGSSINWMVYTRAQRSDFDSWNMPGWSANDLFPYLRKFETYHGRGEREHHGYDGPINISKGTFTAKRAENDFIEAAAKLGYRELKDLQNLDANDGVERWLRYVGPDGRRQDAAHRFLHPKLQSGGYPNLHVLTETHVVRVLIEGGRACGVEYQPNPKFSTEDTKRKIRASKMVCVSAGANATPLILERSGVGDPNVLRRAGVQLIQPLRGVGDQYQDHHLSLWAYRTDLSPREAINGFNDGRFDVHEAIRKNDELLGWNSMDASGKFRPTEADLKDLSPAFRRAWDRDFRNAPDRPLMIIALYNSFFGDYATLPDDAEYVSMACWTAYPYSRGSIHITGPEISDPIDFDVGYLKDADDVDVQKHIWSYKLQREMTRRMSLYRGEEAGSHPAFPKHSKAAIVEFADGPVPANAPKIQYTKEDDKIIEQKVRETVATTWHSLGTCKMAPREQGGVVDSTLSVYGIKGLKLADLSVPPENVGANTNNTALMIGEKAADIFIKELGLKARL |
| 444 | Unigene0004813 | 29 | 43040 | 2.1 | MCISVLSTSHPEYPFVLISNRDEYINRPTLVADWWDEPYKHILGGRDTQRKERGTWLGITKQGRIAILTNFREEGVAVNKDKSRGAITNSYLCIHPDSTETDEEYVRRLLNDVGIHDVGGFTLIFGKLRKPNQVPSPENEVGIVRAESSTAVTRLNILRDHQSPNTNPSSPSRTTPKKNPASPSLCGSAASGLAVISNRTETASSLKRIATHIGETHGLSNSHFGDVTWPKVVHGELRLQQTIKMDVLRGGDEAKFIEGLFDILCVDTLPNRKETENWDEYVRHMRDSIMIPPAKGELAKQMAGYIPASTSLKASAGTTNPPPESSTAQAASGSVAETSYGTSKQTVILVNKQGKVTYVERTLYDQHGTPIPEGERVVRFEFDIEGWS |
| 444 | Unigene0004812 | 29 | 40624 | 2.2 | MCISVLSTSHPEYPFVLISNRDEYINRPTLVADWWDEPYKHILGGRDTQRKERGTWLGITKQGRIAILTNFREEGVAVNKDKSRGAITNSYLCIHPDSTETDEEYVRRLLNDVGIHDVGGFTLIFGKLRKPNQVPSPENESPNTNPSSPSRTTPKKNPASPSLCGSAASGLAVISNRTETASSLKRIATHIGETHGLSNSHFGDVTWPKVVHGELRLQQTIKMDVLRGGDEAKFIEGLFDILCVDTLPNRKETENWDEYVRHMRDSIMIPPAKGELAKQMAGYIPASTSLKASAGTTNPPPESSTAQAASGSVAETSYGTSKQTVILVNKQGKVTYVERTLYDQHGTPIPEGERVVRFEFDIEGWS |
| 445 | Unigene0011178 | 29 | 100316 | 0.8 | SSNQRRRTGWRRLIPTRFCCGLVSLFVVTLVLLLSAGSFWAYKVVPELGQSPPWYPTPFGGTVEGWAEAYEKAARLVGQMSLVEKVNITTGTGWQMGPCVGNTGQVDRLGFPSLCLQDGPLGLRFVENATSFPAGITVGATWNKDLMYQRGKAHGFEAQLKGVNVLLGPSMGPLGRLPAGGRNWEGFGADPVLQGIAAAQTIRGIQESGVIATAKHFVGNEQEHFRQAWEWGTPNAISSNIDDRTLHELYAWPFAESIRAGVASIMCSYNQVNNSYACQNSKLMNGVLKDELGFQGFVQSDWLAQKSGVASALAGLDMSMPGDGLKWQDGVALWGGELSKAALNGSVPMERLNDMVLRVVAAWYQLGQDDSSVWGNNSLSNSATFSSWTNEERGRLHPGASGNNDTDVVNYFVPVRQTEEGGNHDEVARQIAAEGVVLVKNEGFTLPLSKNGANFRRAESDGKVKVGIFGEDAFANPRGPNACEDRACNEWTLGSGWGSGASLFPHLIAPFDALNKMFDEDKVNITAYKKQAAKHAGAIAEKQDLCMVFVTADAGEGFLSYGDIRGDRNDLQVQKHGNTLIVEVAKKCGGKTVVVVHSVGPTLVEEWIDLPGVHAVLMAHLPGQESGNALADVIFGHVSPSGRLPYTIAKKEGDYGPTSKILTFPNHVVPQQNFTEGLYIDYRYFDKHDIEPRFEFGFGLSYTEFELSSLTAEVLGATSTTPATRPRAAAHPPRLDKSIPDPATAFFPEGFNRLKKYIYPYIDKDDKPRTGGQPDYPPSFDPAKPHPLSEAGGAEGGNPDLFTPVLEVSAQVSNTGDFAASAVVQLYISYPDNVKDQDGQIVDMPVKVLRAFDKFHLETDGEEKQKDVKFTLTRKDLSYWDIKLQNWVMPKGEYTISVGFSSRNLPLQFKVDPFAA |
| 445 | Unigene0006595 | 29 | 89743 | 0.9 | IMVRSEKLQAAAAFLASLALVHGQNETTNPGVPAWNHEDYTTSPPVYPSPNITGIGWEEALEKAKEWVAQLTTEEKSALVTGTEGPCVGNIAPIPRLGFNGLCLQDGPLAIRQAVYANVYPAGLTVGASWDKKLAYERAVHIADEYREKGSQVILGPVAGPLGRSGLGGRNWEGFSVDPYLTGVFMYETIKGHQDRGVQACAKHLIGNEQETQRNPTTNDNNNTIEAISSNIDDRTMHELYLWPFQDAVHAGVASFMCSYNRLNASYACQNSKLLNGILKEELGFPGWVVSDWMATHAGYHAADAGLDMNMPGGIAFQVSQPSFWGANLTTSVNNGSLAASRLDDMAHRIMAPYFYLGQDQDYPLIDPSTPSTQRTWNTSQYRYNWTYGDEVVDLRNAERVKHIRELGSAGSVLLKNVNNTLPLKNPKNIGVFGNDAGDIDDGLYFAQLTQEIGYEFGVLPTAGGSGTGRHTYVVTPLEAIKAKAGEGALVQYILNNTIIAEENGFLRVLPSPPDVCLVFLKTWASEAYDRTSLLPDFNGTGVVEAVAKECANTVVITHSAGLNVLPFADHPNVTAIVAAHLGGQEVGNSIVDLLWGEINPSGKLPYTIAKGVNDYDFVPIINSTELLETDDPNAWQDDFEEGLLTDYHHFDYYNLSVQYEFGFGLSYTTFELEGAEITPLTSSPISALPEDLPVAVGGNPALWEKLYSITTTVTNTGDIAGATVPQLYLALPQIANSAITPPRVLRGFEKIHLEPGESQEVTFELNRRDISHWDIYSQQWKISEGVKIGVEVGFSSRDIIATGEFTPIEKAAYRPRRV |
| 445 | Unigene0011179 | 29 | 100316 | 0.8 | SSNQRRRTGWRRLIPTRFCCGLVSLFVVTLVLLLSAGSFWAYKVVPELGQSPPWYPTPFGGTVEGWAEAYEKAARLVGQMSLVEKVNITTGTGWQMGPCVGNTGQVDRLGFPSLCLQDGPLGLRFVENATSFPAGITVGATWNKDLMYQRGKAHGFEAQLKGVNVLLGPSMGPLGRLPAGGRNWEGFGADPVLQGIAAAQTIRGIQESGVIATAKHFVGNEQEHFRQAWEWGTPNAISSNIDDRTLHELYAWPFAESIRAGVASIMCSYNQVNNSYACQNSKLMNGVLKDELGFQGFVQSDWLAQKSGVASALAGLDMSMPGDGLKWQDGVALWGGELSKAALNGSVPMERLNDMVLRVVAAWYQLGQDDSSVWGNNSLSNSATFSSWTNEERGRLHPGASGNNDTDVVNYFVPVRQTEEGGNHDEVARQIAAEGVVLVKNEGFTLPLSKNGANFRRAESDGKVKVGIFGEDAFANPRGPNACEDRACNEWTLGSGWGSGASLFPHLIAPFDALNKMFDEDKVNITAYKKQAAKHAGAIAEKQDLCMVFVTADAGEGFLSYGDIRGDRNDLQVQKHGNTLIVEVAKKCGGKTVVVVHSVGPTLVEEWIDLPGVHAVLMAHLPGQESGNALADVIFGHVSPSGRLPYTIAKKEGDYGPTSKILTFPNHVVPQQNFTEGLYIDYRYFDKHDIEPRFEFGFGLSYTEFELSSLTAEVLGATSTTPATRPRAAAHPPRLDKSIPDPATAFFPEGFNRLKKYIYPYIDKDDKPRTGGQPDYPPSFDPAKPHPLSEAGGAEGGNPDLFTPVLEVSAQVSNTGDFAASAVVQLYISYPDNVKDQDGQIVDMPVKVLRAFDKFHLETDGEEKQKDVKFTLTRKDLSYWDIKLQNWVMPKGEYTISVGFSSRNLPLQFKVDPFAA |
| 446 | Unigene0014007 | 28 | 27285 | 4.6 | MPTKNVVFDIVGTLVSFDAFYARIDEVIGDKLRSKGISPQLFGFSWQTSAEVDFTFLSISGRHTSYSELMKAVFYRTLWFAGISNPREFATDDERDRCQEGYSLLGLREGAKECIEILRNGGFQVWCLTTADIPRVQGYFRRGGVEMAAENFVSCDTQGVAKPALAAYKPVYEKFAEEDQKWFAAAHMWDVSSARLVGFKGAYCRAYEKEDCLEIFGGEMEVMADDLKQMAERIVEAAQ |
| 447 | Unigene0001455 | 28 | 14410 | 5.5 | GKSTGGKAPRKQLASKAARKSAPSTGGVKKPHRYKPGTVALREIRRYQKSTELLIRKLPFQRLVREIAQDFKSDLRFQSSAIGALQESVEAYLVSLFEDTNLCAIHAKRVTIQSKDIQLARRLRGERS |
| 447 | Unigene0001456 | 28 | 18005 | 4.3 | HPATSSPPQHASTTPLHPSHQSSHTMARTKQTARKSTGGKAPRKQLASKAARKSAPSTGGVKKPHRYKPGTVALREIRRYQKSTELLIRKLPFQRLVREIAQDFKSDLRFQSSAIGALQESVEAYLVSLFEDTNLCAIHAKRVTIQSKDIQLARRLRGERS |
| 448 | Unigene0004786 | 28 | 29171 | 11 | MSQQALNKIAPNSPSRQKPNETEQQIATALYELESNIPDMKAALRPLQFVSAREVSUAKEEKSTKRHVHKRWICNGHERGRHVSSIQQRRGGGHWAAFALTPGFLQIEVGHGRKAIVIFVPVPLLQGWHRSQQRLTRELEKKFSDRHVLIVASRRILPRPKRSNRSRTSQTQKRPRSRTLTAVHDAILADLVYPVEIVGKRLRTKEDGSKILKVILDEKERGGVDYRLDTYSEVYKRLTGKGVNFEFPQAPAEY |
| 448 | Unigene0004787 | 28 | 23151 | 13.9 | MSQQALNKIAPNSPSRQKPNETEQQIATALYELESNIPDMKAALRPLQFVSAREIEVGHGRKAIVIFVPVPLLQGWHRSQQRLTRELEKKFSDRHVLIVASRRILPRPKRSNRSRTSQTQKRPRSRTLTAVHDAILADLVYPVEIVGKRLRTKEDGSKILKVILDEKERGGVDYRLDTYSEVYKRLTGKGVNFEFPQAPAEY |
| 449 | Unigene0002350 | 28 | 22330 | 4.2 | MSVPFGIIISGRPVDVSPQAITEAQYAFRIPPAPAFSHIVVFLLPGITLPPGAAASVYVQIPPSQDFKLLGAIGPGKESAIFKISGLKSATNVNQNAMLDDPTAQTGAVPQGDVVIGVSIEPEAQVAAQLATLQSGGQSNNAQLSSALVKLGSSPEGKATTKVLAQRIIGNAFNFLASFGSDTVPLKAFQDWWQKFEKKIDLDPSFLEREGAE |
| 450 | Unigene0000008 | 28 | 56150 | 1.2 | MQWILFLIAFICFLVAAHELIRPVDKKWSSFRRYKLPAGPRGSPIVGNMLEFWRSRSAGTLGKTFLELQSYGEMTTLHMGSTLYVLLNTNRVTKEIIDKRAKVTHERPHMPISGTLISKGMRSVTQPTRIWSELRKQMRPLLNDVWTKKFEAWQDYESMHLLRSYLDDSTQWYLHNSRYATGVMYGVIAGTRLTKTDEEMTLYRKTTMEFLASIQSTVVDFFPWLEHVPKPMQFWRRKWELIGDDHYRVFMSWWDPIKDLATREKASSSWAKDVLLSNNSQFRGSDDEAGYLTNTVISAGGDNPRIAINTCIMASILHPEAALQCRQEIETICCQKGSIRLPNVADVPQMTYTCAFIKEVLRWRPVVPMVPPHTASEDFVFEDCVFPKGTNFLINIPSVCRDYPEPDAFKPERWLDGNQARLTYDFWGFGGGRRICIGYKAVQTALFLPFARLLLCFEFLKDGEFNDRKVNSWSSTAPFPV |
| 451 | Unigene0004942 | 28 | 24503 | 4.5 | MASQQKDETQIALAKKLNHVPWCDQYERMISGMLYDSFTPDLSKARHKVRKFCHNYNTTFPSGDDDTPETLAKNRYEGLHSVLGRVGEGAYLEPPFYLDYGCNTVIGDRFYSGFNFTILDCGLVTIGDRVMVGPNVSIFAATHETEVQSRRDDVEYAKPVVIGSDCWIGGHVVILPGVTIGEGCTIAASSVVSKDIPPWSVAMGSPAKVVKKVTPLDPIK |
| 452 | Unigene0001263 | 28 | 11760 | 11 | MAPKKSSKTHDSINAKLALTIKSGKVTLGYKSTLKTLRSGKAKLVIIAGNTPPLRKSELEYYSMLAKTAVHHFSGNNIELGTACGKLFRCSTMAILDAGDSDILSTTPQ |
| 452 | Unigene0001264 | 28 | 9566 | 13.5 | MAPKKSSKTHDSINAKLALTIKSGKVTLGYKSTLKTLRSGKAKLVIIAGNTPPLRKSELEYYSMLAKTAVHHFSGNNVSATGKRIPTFA |
| 453 | Unigene0014791 | 28 | 33764 | 2.3 | RYEIKIKIKEQVSLHFLTLTKEQVSLHFLQHHNQQQPNMKVTSLIAAAALLAGASATAEAEDKRGVCAVGFGGPCNGDNDPVNFDWLRTLQAQRDNKKARREDVSKVAKRQAGICAIGAGGPCNGESDPGNFDISKWRQRFGTVDFGGDIARRQYLPGQMIRAGPWREGKAPWELQEKLNQAFEDRQSNAKRDAQLCAIGYGGSCNGGAPAWRDLLPAARSAGELAQRDVVSSLARRACAAGYEGFCNDAFLAPPQGFDEELLRNSARPVQIASKRQVYAGGSQEWAERQAQVQELYRLASGRQ |
| 454 | Unigene0012429 | 28 | 39002 | 2.3 | MVLHNPNNWHWVNKDVSTWTKDYLEKDLVGIKAEKNGVNAEVSKVLSMDGDVEVAQRKGKVITIFDVRLQLEWTGNIPVKEEHENDDGTKEEREGTKDVSGTITIPEVAHDTEEDEYVFEVELYSSSLEKEPAKELVRKEITPQLRSHFQKLGPALIAEHGKDVQHAPGENPSAKFTAAKTYSNSGINKSAEKPASTTTTTNNGNKSVVNTTTLSDQQEFRTTADQVYSVFTDPQRIAAFTRGAPKLFEGAHEGAKFEIFGGNVSGSYVKLEKPTYLEQKWRLAQWPQGHYSTLKIRFEQNDVDAVTIMRVDWEGVPIGQEEPTKRNWDEYYIRSIKTTFGFGTIL |
| 455 | Unigene0006462 | 28 | 11176 | 12.5 | MSTAELASSYAALILADEGLEITADKLQALITAAKVPDIEPIWTSLFAKALEGKDVKDLLTNVGSGGGAAPAAAAGGAAAGGDAGGDAPAAEKEKEEEKEESDDDMGFGLFD |
| 456 | Unigene0014863 | 28 | 59737 | 1.3 | KVVQDALNAAGDKASGWAKPLKHLQDELKHLTAEAQSAWEEVAMMFPEEMSKASFFSTPKKHTRKHEREWDFVTKGEHVEQMFSVNSKGEKQREIDGHLEQFNLRTKKVDPKSLGVDTVKQYSGYLDNEEDDKHLFYWFFESRNDPKNDPVVLWLNGGPGCSSLTGLFMELGPSFIGKDRKPSYNPSSWNANASVIFLDQPVNVGYSYSGSAVSSTVAAGKDVYALLTLFFKQFPEYAKQPFHISGESYAGHYIPVFASEILSHKNRNINLQSVLIGNGLTDGYTQYEYYRPMACGDGGWPAVLDESSCQSMDNSLARCQSLIESCYKSESVWSCVPASIYCNNALIGPYQRTGQNPYDVREKCKGGNLCYDELNWIQEYLNRDDVMKALGAEVDSYDSCNMDINRNFLFNGDWMQPFHRLVPDILKEIPVLVYAGDADYICNWLGNLAWTQALEWPGQKAYAKAPMEDLTLQSDSKTKTGSVKSSGNFTFIRIHAAGHMVPYNQPEASLDFLNRWIGGEWVEN |
| 457 | Unigene0004757 | 28 | 27103 | 3.6 | MSKVILLTGASRGIGLSIAHFLLQQSHKLTLVARSEPALRELEKQYPNQTAILPGDFSDLSLGQKAVDLTIKQFGKLDGVVVNHGVLDPVAKVADAKAEEWRDGFTINFFSAVAIAKAAIPHLRESKGTIVFTSSGAATTSYQTWGAYGSSKAAMNHLAASIANEEEDITSISVRPGVVDTEMQRDIREKHNTVMTATDRAMFKGFKEEGKLLKPEQPGNVIARLVLDAPKDLTGKFVTWDGPELKAFQD |
| 457 | Unigene0004756 | 28 | 27103 | 3.6 | MSKVILLTGASRGIGLSIAHFLLQQSHKLTLVARSEPALRELEKQYPNQTAILPGDFSDLSLGQKAVDLTIKQFGKLDGVVVNHGVLDPVAKVADAKAEEWRDGFTINFFSAVAIAKAAIPHLRESKGTIVFTSSGAATTSYQTWGAYGSSKAAMNHLAASIANEEEDITSISVRPGVVDTEMQRDIREKHNTVMTATDRAMFKGFKEEGKLLKPEQPGNVIARLVLDAPKDLTGKFVTWDGPELKAFQD |
| 458 | Unigene0010591 | 28 | 18203 | 4.8 | HEHDHTHPPPAPEDAWKTSGVRVIPSDSLEPVGGQHTTPGMDRSAAINFARVGAQKLWAGTVHIHANAKTGAHHHGHLESVIYVLKGRARMRWGEKLEFTAEAGPGDFIYVPPYVPHQEINASADEKLECVLMRSDSEAIAVNLPDVEPVEKPETVKWIDPTHPE |
| 459 | Unigene0008899 | 28 | 22333 | 3.9 | IPSNRWITLTHRRLQTLPARLTATNTLVTTTSLPSWLKEPIVDRIYHLGKLFEGAPHGINHCLINEYLPGQGIMPHEDGPAYWPVVATVSLGGSLVLDVKGKSGKGEETGIGDADGPKEGGQSWRIFQEPRSLLVTTGEAYTETLHGIAEVTEDENLNAQTVANWELLGDRGVLEENGGKNPRTTRISLTYRDVKKVSTIGSKI |
| 459 | Unigene0008898 | 28 | 29326 | 3 | TMESARIPRVPPSMYYIPNFITPEEEVFILSQVKRKTPULSSQDIPPNQFQLTQNUPPLQIPSNRWITLTHRRLQTLPARLTATNTLVTTTSLPSWLKEPIVDRIYHLGKLFEGAPHGINHCLINEYLPGQGIMPHEDGPAYWPVVATVSLGGSLVLDVKGKSGKGEETGIGDADGPKEGGQSWRIFQEPRSLLVTTGEAYTETLHGIAEVTEDENLNAQTVANWELLGDRGVLEENGGKNPRTTRISLTYRDVKKVSTIGSKI |
| 459 | Unigene0008897 | 28 | 26068 | 3.4 | TMESARIPRVPPSMYYIPNFITPEEEVFILSQIPSNRWITLTHRRLQTLPARLTATNTLVTTTSLPSWLKEPIVDRIYHLGKLFEGAPHGINHCLINEYLPGQGIMPHEDGPAYWPVVATVSLGGSLVLDVKGKSGKGEETGIGDADGPKEGGQSWRIFQEPRSLLVTTGEAYTETLHGIAEVTEDENLNAQTVANWELLGDRGVLEENGGKNPRTTRISLTYRDVKKVSTIGSKI |
| 460 | Unigene0010540 | 28 | 26570 | 5 | MSISSRAATSLVRGATRQAVPALRTSTAIQKREASSTDASTSHAEFKSPFHRGASNKPDTTIIPSFKSYRNNTGETGNKLFQYFMVGAMGGVSALGAKNTVQDFLVNMSASADVLAMAKVEVDLATIPEGKNVIIKWRGKPVFIRHRTADEIKEAEDFDWKTLRDPQSDGDRVKKPEWLIMLGVCTHLGCVPIGEAGDYGGWFCPCHGSHYDISGRVRKGPAPLNLEVPEYDFPEDNALVIG |
| 461 | Unigene0006134 | 27 | 26330 | 2.5 | MSHRFCTEVSATCPVEATTYGYRPDIGGNSFLLAVFGLCTIAQVVLGIRYKIVAFSIVVAIGSFGETVGYGGRIMMNSNPWDSDGFRMQICCLILSPSFLAAGIYLTLKHIVQVLGPEKSRLKPRLYTWIFITCDIFSILLQAAGGGIAASGSGDIINTGNSVMIAGIAFQVATMFVCLVLAADFAFALLRSKTPRSDELEKGQPDATSRKGLYYYLVCFSVAFLAIFVRSIYRYVVM |
| 462 | Unigene0000715 | 27 | 47934 | 7.7 | MAVLPWLQAVLALPSQPSWMKFITPDYYPGAPYILPGPDTRTNICHVKPAVGGGDSTPNILSAFKKCGQNGKIIFDNTTYHIDSVMTTTNLSNVEVDIRGTLLWGTDIQYWLANSLPMGYQNQSTAWYFGGKDIYLHSSTQTGTFNGNGQVWYDFTNGASNYPRRPHQITFGSLHNSVIEDLSFLQSQMWTMTLIHSSSILLRNLYINSTDSQHRDKFGPLNTDGADTIYSHNITFANLTIDCGDDNISSKANSSNILVEDCIFHHGSGIALGSIGQYPGKYEFIENFTARNIEMFGPVRQGGYIKTWTGVQKGVPPNGGGGGTGYIRNVTFENFILHDAEIALDITQCVNFEGGTGDCDTSTFQISDLHWRDIRGTQKRKDAVTDFQCSAKAPCKGIEVTGLEMRAVDGGEKVTGHRCSNVVEPIGFTCD |
| 463 | Unigene0001681 | 27 | 22508 | 4.1 | MKILYIGILRNESKPAVELAAERDLSSFSRFTRSSVGEFLTVFAKTVAERTSPGQRQDVEEQSYTFHSYARSEGVCGIIISDHEYPKLVAHQLLSKILDEFLSKFPKSAWENSNGEVPFTQLKEYIVKYQDPQQADSIMKIQKELDETKIVLHKTIESVLERGEKIDSLVAKSDGLSAQSKMFYTQAKKQNSCCVVM |
| 464 | Unigene0009318 | 26 | 54344 | 1.7 | VMAPPNVDYTLYLVTDSTPAILGDKDLVEVVKNAVAGGATIIQYRDKTSETADLVRIARQLHEVTRAAGVPLLINDRIDVALAVGVEGVHIGQDDLDLKTARRVLGPDAIIGVTANSEEEAIIAAKDGADYLGLGTVFATPTKDNTKSIIGTAGVQLILTSLAGKGLHVKTVCIGGINALNVQRVLYQTASAQKQLDGVAVVSAIVAADDARQAASHLRELVRKPPLFAASSSRPVLSHEDLRIKAPELIKRMAGKKPLCHNMTNLVVQNFAANVALAVGASPIMSNNGLEASDLAGLGGSLVINMGTVTPDMRDNYLKALAAYNAVGGPTLYDPVGAGATQQRRDGVKTLLAGGYFTVIKGNEGEVRTVAGATGFQQHGVDSGASQLSLDEKVQLVKATAAREHNVVLMTGATDVISDGQRTITISNGHALLGEITGSGCTLGTTIASVLAVEREDPLLAAVTAILMYEIAGERAAVRDDVRGPGTFVPALIDELYRIRQESVQGNATWAEAAKIDII |
| 464 | Unigene0009319 | 26 | 50475 | 1.9 | GATIIQYRDKTSETADLVRIARQLHEVTRAAGVPLLINDRIDVALAVGVEGVHIGQDDLDLKTARRVLGPDAIIGVTANSEEEAIIAAKDGADYLGLGTVFATPTKDNTKSIIGTAGVQLILTSLAGKGLHVKTVCIGGINALNVQRVLYQTASAQKQLDGVAVVSAIVAADDARQAASHLRELVRKPPLFAASSSRPVLSHEDLRIKAPELIKRMAGKKPLCHNMTNLVVQNFAANVALAVGASPIMSNNGLEASDLAGLGGSLVINMGTVTPDMRDNYLKALAAYNAVGGPTLYDPVGAGATQQRRDGVKTLLAGGYFTVIKGNEGEVRTVAGATGFQQHGVDSGASQLSLDEKVQLVKATAAREHNVVLMTGATDVISDGQRTITISNGHALLGEITGSGCTLGTTIASVLAVEREDPLLAAVTAILMYEIAGERAAVRDDVRGPGTFVPALIDELYRIRQESVQGNATWAEAAKIDII |
| 465 | Unigene0007316 | 26 | 35863 | 3.3 | VADHYDGRRERRAGCPAHWQRAGUPSPDPISIGVIGGTGLQSLAGFTHVASLKVDTPWGSPSSPVSVLHHPSPSTGKPIPVAFISRHGLHHEFAPHEVPARANIAALRKLGVRTIIAFSAVGSLQEAIKPRDFVVPDQIIDRTKGIRPFTFFEGGMVGHVGFADPFDGKIADIVRRCGHSLEGDGVILHDRGTLICMEGPQFSTRAESNMYRSFGGSVINMSALPEAKLAKEAEIAYAMICMSTDYDCWHESAGDVTVEMVMGNMKANSENAYRFVGAVLNELSKEEHADVVSATHLAGQSKFAGSMTAPAGRSAKAKENLTWLFPDYFD |
| 465 | Unigene0007317 | 26 | 33429 | 3.6 | VADHYDDPISIGVIGGTGLQSLAGFTHVASLKVDTPWGSPSSPVSVLHHPSPSTGKPIPVAFISRHGLHHEFAPHEVPARANIAALRKLGVRTIIAFSAVGSLQEAIKPRDFVVPDQIIDRTKGIRPFTFFEGGMVGHVGFADPFDGKIADIVRRCGHSLEGDGVILHDRGTLICMEGPQFSTRAESNMYRSFGGSVINMSALPEAKLAKEAEIAYAMICMSTDYDCWHESAGDVTVEMVMGNMKANSENAYRFVGAVLNELSKEEHADVVSATHLAGQSKFAGSMTAPAGRSAKAKENLTWLFPDYFD |
| 466 | Unigene0010678 | 26 | 8474 | 13.2 | DKANHAVIFDKNTTEKLNKDVQSYRLITVAVLVDRLKINGSLARQALKDLEERGVIKQVVGHSACKIYTREVGGGE |
| 467 | Unigene0010090 | 26 | 57597 | 1.8 | MDAIKDTVNKLASSLHIGGGQQQPASQPPTEQEFKELKERYEKAGQGQVFSFWEELNTQEQGELYQQAKSIDPENINKITKKTLHPAKTEGEDKKPELEPLPESATTSTIDSDENDLKKWYDSGLKLISEGKVGVVLMAGGQGTRLGSSAPKGCYDIELPSHKSLFQLQAERIWKLQHLASKIHSKEEVTIPWYIMTSGPTRKPTQEFFEEKKYFGLNRNNVLFFEQGILPCITMEGQILLESKSKIAVAPDGNGGLYNGLINAGIVHDMEKRGVKHIHAYCVDNCLVRVADPTFIGFSAEKNVSIATKVVRKRNAKESVGLILQKNGKPDVVEYSEIDAETAEAKDPKDNNLLKFRAANIVNHYYSFEFLDSIPQWAHRLPHHIAKKKIPTVDEKGNPVKPEKPNGIKMEQFVFDCFPFLEMDKFASMEVKREDEFSPLKNAKGTGEDDPETSRKDILLQGKRFLEEAGAVVVSEKQDEGVEVSPLISYSGEGLEYLKGREIKAPAVIEKE |
| 468 | Unigene0008211 | 26 | 10031 | 15.4 | MSTGNPNYGVGGTAKDGGLSTADTVRDGGSDGGNRGSDDTYVPSSSDDGGRQTRSKTSAIASDVSAREDKTSKSGGQVGSAVASEGEGGSGAHVGNPNRNVGGT |
| 469 | Unigene0006001 | 26 | 12456 | 4.5 | EVADFPFFDIETLSAPTQVEVKYAEDFTAFDNPYSDGPWTFSNGLSTSFRTETFEFSKPGRQQAYFLQGGQRWQTIRLLTNTTVTFRSVGLNSTSKNEPSSAFAGQLHTSN |
| 470 | Unigene0011404 | 26 | 39261 | 3.3 | MAPSVLIDNTPDFDMTDDFVAPVKQTNASSSNRTLLLAPPSVASHPSALTEVAEAYDRSQTDIQMLDRLAVGLVSLPATTYDVVLLLSDVGSSNEGTKLLDRAVMQRIVDSLKPGGRLKSQDGQLGAVQSSEKTEAILAGLMDGNGDGMMKPDSAASTQTVKLSFGKKKKADAAAVPANELEAANTGKRKMGDVQVQHTNGNGAAPAVKSTPAGVGFIENEADWDSADDEFPDDDELERAGKIDPDTLLTEEDRQKPLNIPEACKPNGTKRRRACKDCTCGLAERIAAEDNAKRAEADKNLQKLNVAAPTLAADDLTEVDFTVQGKVGSCGNCALGDAFRCDGCPYIGLPAFKPGEEVRLLNNVVQL |
| 471 | Unigene0002106 | 26 | 24453 | 3.1 | PKVLFVLSSHDQMGDTGKPTGWYLPEFAHPYYKLEGKAEIVIASPKGGAAPLDPASVEMFKSDEESTKFLKEKEALWKNTQKLSDFVGKANTFDAVFYVGGHGPMFDLATDPVSHQLIKEFYESGKVVSAVCHGPAAFVNVKLSDGSWLVQGQEVTGFTNTEEDQVQLSAAMPFMLETELEAHGGKVVKAEPWQPKVVKSGKDNKLITGQNPASASPIGEAILAAL |
| 472 | Unigene0001596 | 26 | 30344 | 3 | MAAAAASVVPGLDQRPIKNTICLFDVDGTLTPARRTVSPEMLQLLSALRHKVAIGFVGGSDLAKQQEQLGTASIPVTSLFDFCFAENGLTAYRMGQPLASHSFIKWIGEEQYKKLVRYILHYIADLDIPVKRGTFIEFRNGMINVSPVGRNASVQERNDYEKFDLEHGIRKTFVESLKKEFPDLGLTYSIGGQISFDVFPKGWDKTYCLQHVENEKNLPGGVEYTTIHFFGDKTYQGGNDYEIYEDPRTVGHSVTNPDETAAELKKLFGL |
| 473 | Unigene0014201 | 26 | 34719 | 3.8 | WGSLGHQTVAYLAQHYVSNTTAHWAQAILNDTSSSYLANIATWADSYRYTAEGEFSASFHYIDALDTPPTSCNVDYERDCPPEGCIVSAIANYTRRVSSPAELEPLQVNYALRWIVHFSGDIAQPLHVENYEIGGNGVAVTFDGNETNLHAVWDTYIPEKMRGGYGLEKAKEWAEVLADEIQKGEFKDVAKGWLQGIDVNDPVATSMAWARDGNSYVCSVVMPDGGEVLEGKELYPEYYESATATVQQQVAKAGYRLAAYLDAIAETQAGAPSYYRRGVHADMKKRDLSGRDFLPKTQGLSTVQRARLGKRD |
| 474 | Unigene0012944 | 26 | 102110 | 1.2 | MPIMASAAATNDAKPTRERKPSSSAPITDFQGPVGPSGISRPKHKRTVTGFGPQEIKSVEASIPEPQREAWRKYSASEFKSKDEFDRDVVRHIETTLARSLFNCDEAAAYSGTALAFRDRLIIEWNRTQQNQTYADPKRVYYLSLEFLMGRALDNAMLNTGMKDVAKDGLHDLGFRMEDIISQERDAALGNGGLGRLAACFLDSLATLNYPAWGYALRYRYGIFKQEIIDGYQVEIPDYWLDFNPWEFPRHDVTVDIQFYGNVRKYTDENGKQISVWENGEIVTAVAYDAPIPGYNTSTTNNLRLWSSKASGGEFDFTKFNSGEYEASVADQQRAETISAVLYPNDSLERGKELRLKQQYFWCAASLFDIVRRFKKSKKAWKEFPNQVAIQLNDTHPTLAIPELQRILIDQEGLDWDDAWSIVQKTFGYTNHTVLPEALEKWSVPLFQHLLPRHLQIIYDINLNFLQYVERNFPKDREMLGRVSIIEESQPKMVRMAYLAVIGSHKVNGVAELHSDLIKTTIFKDFVKIYGPDKFTNVTNGITPRRWLHQANPRLSELIASKLGGHDFLSDLTLLNKLEAYTDDKDFRKEFQEIKYANKVRLAKYIKEANGITVNPASLFDIQVKRMHEYKRQQLNIFGVIHRYLELKEMSPEDRKKVQPRVSIFGGKAAPGYWMAKTVIHLINQVSQVVNNDKDIGDLLKVVFLEDYNVSKAEIIVPASDISEHISTAGTEASGTSNMKFVLNGGLIIGTCDGANIEITREIGEDNIFLFGNLAEDVEDLRHSHFYSEFHLDPMLERVFKTIKEGRFGDAGQFSALVNSIVDHGDYYLVSDDFKSYVDTQNLIDEAYKNQEEWLTKTITSVARMGFFSSDRCIDEYAEMIWNVEPLPPKANGA |
| 475 | Unigene0003153 | 26 | 47054 | 1.8 | MTVADSIKDAAQSAKEAIGLSNGSSGESKSARKKKAKAEAAAASNTTPSAATAVPKPSSQENSVAAHSTTEQDEHLKDLNKQIRNTNKKLQGMQKLDAILEAHPGVSLDELLAQRKINQDQKAQAQKKPELQAHLAQLEEQAEAYRAVDARYQDKFNKLREELETKHKQEAKKLREELAVERENASQNDLRKNLLVFSRFLRAAAAKRAAEVEEDTEENRAFEGALLLVYGGDDSAVDAAVNIIEGAEEKVPSIDGTLTETTYAQVKQTAVKHTPFQATEEWAEGVARETAGESGSDPTIANAGLTELEAPNTAAEVQPEPTSVQGTAGDSGNVAGERWNTDAAGTSAGAEKSGLEESYEIIPRPSDEVDVPAAASTRAQQGSSWAEESQEAATGNKAGENWDTKAPGESNGSWGAEPVVSANGWGDAPATDAAAPADDGFSPVA |
| 476 | Unigene0013179 | 26 | 36701 | 2.1 | PYQFARFLHCFITQLVLIILHRALLPTHPRYQSFRIELQKAYSSSTALYFPTFTHRLPADYPEKDALPVTGKGWSGYIIPGYDRTVLSRATDKENVTVILFAHGGGYARGEARMYVPYMKRWVACAKSKGLNIVFLSVEYPLSGEATHPAQRSAFLNAYRYLLDLGVPSGNIVFMGDSAGGGICINSALHASSENMPQPAASILISPWIDMSLSAYEGGNQAVMSDYFIMANEAVPVLTKAFLGNYAGTDGDANALYRPLNQLRGLNPQLIFVGAAEFALSDSKDWAQRCREAGVLCELHVEWGQTHIWAMGSKFIEPELRRKTDERI |
| 477 | Unigene0011713 | 26 | 30438 | 3.2 | IEIEALQLPSPVLAPDVWGNQKEQPATVNVKLLLHSTFQSASEKDKLDDSTIHYGNLAKAIRSKSTSSQTVEDVVSGTEQAVHQLASKSEGKFVVKESAVEVHLPKGSMLGSGAVVSRRTTWDQKGEKKQAQEGFEVRDVVIPTLIGVNAYERGLKQPLVVTVGLEWRTADQQSQQKEVLFALEKEVVNIVQETAYETLETLVEYVYAQLLRKTPQALSPGSRFRLRIEKPRAIAFADAPVIEIVRTVPAEQAAQSSTSSHDALSVTLAGLSVVKPYSG |
| 478 | Unigene0001244 | 25 | 48910 | 2.1 | LGNSAVRGNFWYTTSVQQTVATVISTVLDYGDREEVANVSTNYVPADQLYTSFGVYRIRTAIFGDDVVTVNRELEALGVPSTLIQGRDYAATIVYQANASFDHGVAYPTPFAIWNDVQVLYLDQTTSCIPTATGRPTISSLGGYGRIDIDRDEDGRYLSDRATKYYYTPSAYDSERYFYVQTLPLDGPQPSLGNYALPSGAIERYVSIQSSAYPWITDCTPADTPGEPTVHIAVNQLTDTSRVTVRMAGPTTDPTPRPTGDTPALPDTPSPAPPSEDDDEPIPVQTPAPPAEDDDGPVQVQPPANTQVPNSGGTPDTGSGANPPQNPTTGEQQPANPGETRPDNSSPNNAGPGGTSFEDTSPNAQQSPASSNPQSPNPNQNDQGPQSDDSDDIPSSPAVVVPGELPASGTVQGGAQGQTTAPGSGDDTSDESVPSGTGTDDQTSGATGSDDEVEDAPASGDSSATGDV |
| 479 | Unigene0010497 | 25 | 50786 | 2.4 | MLDAIVIGAGLSGLQAARSLQEAGLQVVVLEARNRVGGKVWSVPLASGRGVVDLGAAWVNDQLQPRITKYLKQWNLKRVEQRLGHTAIMQDSEGGISTFPHGIMPEFSEEEKKNLGVVRDHIQAESLKAGRPDSKDDAVSLDQYARNLGATDKTLKMVNLWARVMHGVESTEESAAFFIDYCRKNQGLLSIRADDKTGGNYQRLHRGTQSIANGLHELVGQHNVYFSSPVKFVEDNRTHVVVTTIGGKVFKARKVIISIPSTMYQDLTISPPLPAGLQELADNATMGDYNKMIVCYNTPWWRESSYNGFFMSYSGPSPLARDTSVEEVGLYCLTCFVNGDLGRKWSKLPPHERRATILKQLAKIYKAGTDSEVWRPIEMFDQIWKHEQYSKGALVPITKIGHLTKHADVYGKSVGNIHFCGTEYATEWKGYMEGALCSGENVAKEILNVVGSDAR |
| 480 | Unigene0004198 | 25 | 61654 | 1.3 | MGLEQMDGPYGYDARPSDARSAYANSAYATPLTEDMPDLGGVPDMPALPPNGFSKYANGLSKPANGDSKSVEHIKIPREIRAVKDAESYEPTGKNIVICLDGTGDQFDADNSNIVHFVSCLKKAVPEQVTYYQSGIGTYDGGGVTNGINAGLDMAVGSGLGVHIRDAYMFIQAHYKQGDKICLLGFSRGAYTARCLAGMIHKVGLLPAGNVSQVPFAYKYYKDDTDQGWEMSQEFRKTFCIPVNVYFVGVFDSVASVGFFPRKLPLSSTPTHRAEYFRHAMALDEHRAKFKACRYKVKDSMGEKYTVSAEEDVVDTDVREVWFTGCHADVGGGAVKNEVRHKLAQIPLRWMIRQCFESDTGIIFKTPNLANEGLDIHTLWPKYEPLSVPTGPPPPDMLDRYHKGLLGPIHRRSSLLEPVDHNDEYGMHHLKIYKETDSEKPSADLHEHWTPEQVEDYFDALQKVNDQLVDAKGWWVLELWPIKIRHQAKDSDAWVKKIGLNLGTYRAVQDKTPNLHWTVKHRTDQMDYRVRTRMDRKAEWRVVV |
| 481 | Unigene0007326 | 25 | 58394 | 2.1 | ILMYRKFAIASGFAVAALVKGNVAVAVAGNGNGDEYGGSMSKSSTSSDVPDYFVTKPELFPGPTPTGDAAFLAQTNPAPFAGTTYIPNSPLATQVPIVGNNDNGNIFQMHGQLSHYFPNPQGFGVDEYSLPKNASIVQLNMLSRHGSRYPTTGAGAAKIGQKILNYTTGLSGHAMFSGPLEFLNEWTYKLGAEILVPVGKQELFDSGTLHQIMYGHLYKNNGTKITARSTTQDRMLKSAEYFLAGFFGLEWTHNATLVLAIEEDAGVWNNTLAGYYNCNNSNTGVSAGGSNASTQWSSIYLADAPARLNNYSSGFNWTVADAYNAQSLCAYETVALGYSAFCGLFTYSEWEGYEYSIDLSFAGNNMFQSPTGRAVGIGYVVEILARLQHHLITEPVAQVNVTLDSNPATFPLGQTLNFDFSHDTNIAAILTAFGLTQFAPVLPTDRIERNRSLIVSHMEPFGARLDMEIIETPKPLSGNRKDGNSYEEGGVTRYIHFILNQRTIPLGKSLSKCGERDDGWCELTTFMEVQSKMLE |
| 482 | Unigene0011374 | 25 | 57781 | 1.6 | HGRTAPSTAYLHLLRNLLDAPAHACRPAITRRSAAPLQPATFTASTRHVQRHSQIARSDHDRIQRRPYTSSPRHQETFWRRLRQSRSATKPTANDNLPPLAGFLDDNAGLGGRIIKPSNELKLRCTEFDENGNVTLVNGEFRKSELIQKYSLLPRDLRKIDSSVLPHILVRPSAILINLLHLRCLIKHNRVLVFDVYGSTDSYAQSLFMYDLEGKLRQKQTTSAAGNLPYEFRALEAVLISVTSGLESEFEGVREPVVRVLRELEEDIDRDKLRNLLIYSKKLGTFEQKARLVRDAIDDLLEADDDLARMYLTEKAAGTEREDDNHEEVELLLESYHKVADEIVQVSSNIVSAIRNTEEIVRAILDANRNSLMLLDLKFSIGTLGISAGMFIAALYGMNLENFIEESNLGFFGVTAASTVLSVGACWWGLRKLRKVQRLSMWGEGGAVKNNVRSSRGRWSEVEGRSDGEKPLAEAVAGIKKAEGLRSWKVAQNERLKHMHGDATKSAPPLALR |
| 483 | Unigene0013827 | 25 | 90371 | 1.1 | MHLFNTLTLLSAVGSTVVQAQLLQIPAVQQIVDQALRIFDDYVDYDGVTPQQAQDTSRTSAPATANVLAVAAANSCDDYWMQAASHQGIAAFNSNPSGYQVFRNVKDFGARGDGVTDDTRAINNAISSGNRCAPGACGSSTTTPAVVYFPTGVYSVNASIIDYYYTQMIGNPKPGCMPTIRASRTFTGGLGVIDGNQYGANGLGYGATNVFWRQIRNLIIDTTLVPAANAITGLHWPTAQATSLQNLVFNMNSQRGTQHQGIFIEEGSGGFMNDLVFNGGLYGANWGNQQFTSRNLTFRNCVTAINQIWDWGWTYKSININNCTTGINMTNGAPNGLNIGSLTLLDSSITNTRVGIQTGRTRTSNPPTAGGLVVENVRFSNVPIAIRGPDGTRVGNVQSIAGYIEGHAYTPTGPTEVQQSLTLNVRPSALLQSDGKYYERSKPQYENLPLSSFVSARTAGARGDGRTDDTRALQAAINSARSQNKVLYLDHGNYIVSNTIYIPAGSKIVGETFSVIMSSRSFFNNLTNPQPVVQIGRPGEQGSIEWSDTIVSTLGQQKGAVMIQYNLVAPAGAPTGLWDVHVRIGGFAGSKLLVADCPKTPTVQVTPERIDQDCISGFLSLHITNGSTGLYLENVWLWVADHDIEDRALTQITIYTGRGLLDQSQGPVWMVGTSVEHHVRYEYQFSNARNVFAGQIQTETAYYQPNPNARNPFPSVSSLADPSFNDATITDQGATIPAANGWGLRIKDSTDILIYGAGLYSFFNNYSTQCSNQGSGSVCQNRITSIEGSNTRTSIYNLNTVGTRFPVTVDGVNRAYYNDNQGGFTQGIALFRN |
| 483 | Unigene0013826 | 25 | 90371 | 1.1 | MHLFNTLTLLSAVGSTVVQAQLLQIPAVQQIVDQALRIFDDYVDYDGVTPQQAQDTSRTSAPATANVLAVAAANSCDDYWMQAASHQGIAAFNSNPSGYQVFRNVKDFGARGDGVTDDTRAINNAISSGNRCAPGACGSSTTTPAVVYFPTGVYSVNASIIDYYYTQMIGNPKPGCMPTIRASRTFTGGLGVIDGNQYGANGLGYGATNVFWRQIRNLIIDTTLVPAANAITGLHWPTAQATSLQNLVFNMNSQRGTQHQGIFIEEGSGGFMNDLVFNGGLYGANWGNQQFTSRNLTFRNCVTAINQIWDWGWTYKSININNCTTGINMTNGAPNGLNIGSLTLLDSSITNTRVGIQTGRTRTSNPPTAGGLVVENVRFSNVPIAIRGPDGTRVGNVQSIAGYIEGHAYTPTGPTEVQQSLTLNVRPSALLQSDGKYYERSKPQYENLPLSSFVSARTAGARGDGRTDDTRALQAAINSARSQNKVLYLDHGNYIVSNTIYIPAGSKIVGETFSVIMSSRSFFNNLTNPQPVVQIGRPGEQGSIEWSDTIVSTLGQQKGAVMIQYNLVAPAGAPTGLWDVHVRIGGFAGSKLLVADCPKTPTVQVTPERIDQDCISGFLSLHITNGSTGLYLENVWLWVADHDIEDRALTQITIYTGRGLLDQSQGPVWMVGTSVEHHVRYEYQFSNARNVFAGQIQTETAYYQPNPNARNPFPSVSSLADPSFNDATITDQGATIPAANGWGLRIKDSTDILIYGAGLYSFFNNYSTQCSNQGSGSVCQNRITSIEGSNTRTSIYNLNTVGTRFPVTVDGVNRAYYNDNQGGFTQGIALFRN |
| 484 | Unigene0009439 | 25 | 45167 | 2.5 | MASKNATRALRASLRQLKAPQVQQRTFITAVQGSKQQGVRAAQRAATSAIVHQSRGKKTVDFAGDKEVVFERADWPREKLLDYFKNDTLALIGYGSQGHGQGLNLRDNGLNVIVGVRKNGSSWKEAQQDGWVEGKNLFDIDEAISRGTIVMNLLSDAAQSETWPHIKPQITKGKTLYFSHGFSPVFKDQTKVEVPKDVDVILVAPKGSGRTVRTLFREGRGINSSVAVWQDVTGKAEEKAIALGVAVGSGYMYKTTFEKEVYSDLYGERGCLMGGIHGMFLAQYEILRENGHSPSEAFNETVEEATQSLYPLIGANGMDYMYEACSTTARRGAIDWSKRFKEALKPVFADLYDSVKTGKETQRTMEYAGRPDYRQAFEKEMEEIRNLEIWRAGKAVRSLRPENQ |
| 485 | Unigene0001736 | 25 | 14392 | 8.5 | MGDSSVVAPSWRQVEVGRVVHFGSGPYAGRIAAIVQIIDHKRVLVDGPSKVSELATPRHSAPISSLSLTGLVIPKIAFGAGVTALSKQWEEFGVDKKWTESPYAKKIEKATRRKQLSDFERFKVMRLRKQ |
| 485 | Unigene0001735 | 25 | 15907 | 7.6 | MGDSSVVAPSWRQVEVGRVVHFGSGPYAGRIAAIVQIIDHKRVLVDGPSKVSELATPRHSAPISSLSLTGLVIPKIAFGAGVTALSKQWEEFGVDKKWTESPYAKKIEKATRRKQLSDFERFKVMRLRKQARFETRKALATAKA |
| 486 | Unigene0011287 | 25 | 44437 | 2 | MPPQTFFLVSLPTSISPANSRDEALTTLRSAVNPDNGTTYPFAIPEFKIGTLDALVQQADELAKLEQGCKGVVDKVGDSLRSLLEGDEEKLQEQKVVNDRPVENYLQSFQWNKVKYRADKPIAELIDSLQKEIAAVDNDVKAKFSQYNQTKTNLATLQRSQTGNLSQKSLNAVVNPDTLIQPDQSEYLQQHLVAVPSQLVKDFLKTYESIAPMVVPRSAQLLAKDDEFQLFVVTTFKKHASEFVHKCREHRWTPRELKFTDGGREAEEAELRKLEKEERKTWGEALRLGRTGYSDAVMGWIHVLTLRVFVETVLRYGLPLAYVCGLVKTTPKLSKKAKTGLDNRFSDLGGNALSRDKKGRPQQDDSAMQQEMAGAGLGGDQGYEPYVFYEFEII |
| 487 | Unigene0008456 | 25 | 110967 | 1.1 | MMGFNASWHSCLVLLLAAFTFITPAVSVKHENFKTCDQSGFCKRNRLYADTALAASKWEAPYSLDRSSVKFNHGLLTGTVYKKLAHSDESVRLPIKIAFYESGVARVTIDEEKRQKGQIELRHDSKIRKERYNEAESWVIVGGTIPSKGAALSNDAAKGTTKVVYGPASKFEAIIQHSPFAIEWKRDGETQVNFNGRGLLNVEHWRKKVDRPEPEKKEGEETAEKKHDNAEDESTWWDEAFGGNTDTKPRGPESVALDISFPGYDHVYGLAEHASPLSLRETRGGDGKYKEPYRLYNSDVFEYEMDSPMTLYGSIPLLQAHKKDSTVGVFWLNAAETWVDITKRSSLANKVGLSSGASTDSHFISESGLLDVFVFLGPTPQDVVGAYTELTGTQQLPQHFSIAYHQCRWNYVTDEDVKDVDKKFDKNNIPYDVIWLDIEYTEGKKYFTWDPLTFKDPLSMQKQLDEHERKLVAIIDPHIKNEANYPIVDELKSKNLAVHNKDGNIYEGWCWPGSSHWVDCFSPAARKWWAGLFQYSKFTGSAKNLWLWNDMNEPSVFNGPETTMPKDNVHHGNWEHRDVHNLNGLTLINATYEGLLARDKEEAKHNVRPFVLTRSFFSGSQRLGAMWTGDNQANWEHLEASIPMVLSMGISGFPFAGADVGGFFGNPEKDLLTRWYQAGIWYPFFRGHAHIDTRRREPYLVGQPYQEIITQALRLRYSLLPAWYTAFHESHVSGAPIVRPNYYVFPGDEKGFAIDDQLYLGSTGLLAKPVTKAEQPGTTIYLADKEKYYDYFDFWTYEGPGEVAVSSPLETIPLLMQGGHIIPRRDRPRRSSGLMKYDPLTLVVVIGNSGDAQGTLYLDDGESFDYKDGAYIHRKFHFDGKLQSLTSEDLNHGASSKSTKAYFKTMEKVRVEKIIVVGAPESWKGKTQVEVTESHAGKSEPARHATLEFHAKDDGKAPWAVLKNPQVKIQSDWKITFG |
| 487 | Unigene0008455 | 25 | 110967 | 1.1 | MMGFNASWHSCLVLLLAAFTFITPAVSVKHENFKTCDQSGFCKRNRLYADTALAASKWEAPYSLDRSSVKFNHGLLTGTVYKKLAHSDESVRLPIKIAFYESGVARVTIDEEKRQKGQIELRHDSKIRKERYNEAESWVIVGGTIPSKGAALSNDAAKGTTKVVYGPASKFEAIIQHSPFAIEWKRDGETQVNFNGRGLLNVEHWRKKVDRPEPEKKEGEETAEKKHDNAEDESTWWDEAFGGNTDTKPRGPESVALDISFPGYDHVYGLAEHASPLSLRETRGGDGKYKEPYRLYNSDVFEYEMDSPMTLYGSIPLLQAHKKDSTVGVFWLNAAETWVDITKRSSLANKVGLSSGASTDSHFISESGLLDVFVFLGPTPQDVVGAYTELTGTQQLPQHFSIAYHQCRWNYVTDEDVKDVDKKFDKNNIPYDVIWLDIEYTEGKKYFTWDPLTFKDPLSMQKQLDEHERKLVAIIDPHIKNEANYPIVDELKSKNLAVHNKDGNIYEGWCWPGSSHWVDCFSPAARKWWAGLFQYSKFTGSAKNLWLWNDMNEPSVFNGPETTMPKDNVHHGNWEHRDVHNLNGLTLINATYEGLLARDKEEAKHNVRPFVLTRSFFSGSQRLGAMWTGDNQANWEHLEASIPMVLSMGISGFPFAGADVGGFFGNPEKDLLTRWYQAGIWYPFFRGHAHIDTRRREPYLVGQPYQEIITQALRLRYSLLPAWYTAFHESHVSGAPIVRPNYYVFPGDEKGFAIDDQLYLGSTGLLAKPVTKAEQPGTTIYLADKEKYYDYFDFWTYEGPGEVAVSSPLETIPLLMQGGHIIPRRDRPRRSSGLMKYDPLTLVVVIGNSGDAQGTLYLDDGESFDYKDGAYIHRKFHFDGKLQSLTSEDLNHGASSKSTKAYFKTMEKVRVEKIIVVGAPESWKGKTQVEVTESHAGKSEPARHATLEFHAKDDGKAPWAVLKNPQVKIQSDWKITFG |
| 488 | Unigene0005195 | 25 | 11976 | 8.7 | MSRSFSSAARALYRFVWGRASRDETYEAQLKNALSKNSKLKDSDEIRFQUGHVIFKSERYRLTAVSGGEHVSKADPNLRVSGEIFKNDKRQTSVHAYKDGRVEY |
| 488 | Unigene0005196 | 25 | 9990 | 10.3 | MSRSFSSAARALYRFVWGRASRDETYEAQLKNALSKNSKLKDSDEIRFHGGEHVSKADPNLRVSGEIFKNDKRQTSVHAYKDGRVEY |
| 489 | Unigene0011564 | 25 | 124406 | 0.7 | TIEELDATVRAFYEGRGDQQKAAQASLNQFKENPDAWLMVDQILEKAQYPQTKYLGLQVLDNVIMTRWKVLPRDQCMGIRNFVVNVIIQQSSSEESLKKERALLNKLNLTLVSILKQEWPHNWPTFINEIVTSCRSSLPICENNMAILRLLSEEVFDFSAEQMTSTKTRQLKQSMCDEFTSIYQLCSEILRTADQPSLIKATLETLLRFLNWIPLGYIFETPPGTQMSLIELLRSRFLEVPEFRNITLKCLTEIGSLQTEQNWNEKLVEMFTETLTTISKIIPLTLDLKQTYASSNGRDQEFVQNLALFLCNFFSNHLSLIEALPNRDFLIHGHFYLIRISQIDDREIFKICLEYWTKLVCELYDEMQQIPITEMNPLINMSGMANGGGAMNPQILANYPLRKHKYTDVLSNLRQVMIEKMVRPEEVLIVENDEGEIVREFVKESDTIQLYKTTRECLVFLTHLDVVDTEQIMSEKLARQVDGTEWSWANCNTLCWAIGSISGAMNEETEKRFLVTVIKDLLGLTEMKRGKDNKAVVASNIMYIVGQYPRFLKAHWKFLKTVVNKLFEFMHETHEGVQDMACDTFIKIANKCKRHFVIQQPGETEPFIDEIVKTMRKITCDLSPQQVHTFYEACGYMISAQGHKNTQERLIGELMSLPNAAWDQIIQSAHQDPSILQNAETIKVVGNIMKTNVAACSSIGAYFYPQIGRIYMDMLTMYRASSQLIDEAVQRDGNIATKMPKVRGLRTIKKEILKLITTYVEKADDLEMVHTTLVPQLLEAILLDYKNNVPDAREAEVLAVITVLITKLQGMMTEQVPAILDNCFECTLDMINKDFSEYPEHRVEFFKLLRAINQRCFPALIKLDQAHFKLVIDSCMWASKHDNRAVEGEGLNMCIELVENMANHTDQQTCDAFFQSFYTTILQDVFFVLTDSDHKAGFKYQSMLLARLFWLVGANKISQPIYTPDQATAGTTNKDFLQNFVASLLSNAFPNLQAAQITNFIKQLFDSTEDLPKFKLILRDFLIQLKEFAGDNAELFQEDREKAIRDAKDAERERMSKVGGLLKPSELDEDDL |
| 490 | Unigene0000388 | 24 | 48835 | 2.1 | KAKYGGRIPTFRHENRRWPDNVLEKSPVLFSTDLRDGNQALRNPLTFEFKLRMYHLLLSMGFKEIEVGMPCANRSEYEFIRHLVDTDLIPAGVLIQAITPCSEPAVKRTIESLHGASQAVIFTYLPSSDNYRETVTGDSEKEWIARAVRVTKFARSITSTGMYSMRTKWTFNFGFEDYSNARLEAVVRCADAVKAAWSPSPQHKMLFAIAASVESSTANVFADQIERFCQNVSERSCWRLSVHTHNDRGGAVAAAELASLAGADRVEGCLFGNGERAGNMDLVTYGLNRMTEGIDCGIDFTRLRDARQVYQDVVGIPIHPRTPYSGSYFLKAFSGGHQDAIVKGIERRASAEKANTPNAFWPQWKVPYLPIDPVDIGEDIENSIEINSQSGKSGVRWVLGSRIGPVPITDAAKTAETVKERSVSLGRALSWQEVC |
| 491 | Unigene0007401 | 24 | 65238 | 1.5 | MEPENADRQLNQRQPEDPRYLNFKHSQPGSGLNKFSSTLTREHDFPAAQAMLYAAGVPNEKALKTYPQVGIASVWWEGNPCNTHLLDIGKEVKKAIEKQEMLAWQYNTIGVSDGITMGGEGMRFSLQSREIIADSVETVTCAQHHDACVAIPGCDKNMPGVVMGFARHNRPSIMIYGGSINPGYSKLQERPINIATVLEAHGAYIYDNLKNPKKPEQTKDDLLTDLERTSCPSQGACGGMFTANTMAMAIETIGLSLPGSSSTPATSPAKMRECAKVAEAIKICLEKDIKPRDCLTKKAFENAMTMMMALGGSTNGVLHLLAMAGTAELELTLDDFQRISDKVPFIADIAPSGKYLMVDLYEIGGIPSVQKLLIAAGLLDGSTITVTGKTLAENVESWPSLKQGQDLIRPIDKPIKATGHLEILRGNLAPNGAVAKITGKEGMKFVGKARVFNKEHELDDALNKGEIPRGENLVLVVRYEGPKGGPGMPEQLKASAAIMGAGLKNISLITDGRYSGASHGFIVGHISPEAAVGGPIAVVQDGDVITIDADNNRIDFDVSEEEITRRLGEWKPPKMPVTRGVLAKYARLVGDASQGAMTDLF |
| 492 | Unigene0013534 | 24 | 117435 | 0.9 | SADGSVKQTTTRAERRPMPGSFVDIPSDDSAKSDHANDSMATTPPKPLSRNNSDFPPTPPTMTSSELADPISAPRRLSPSPVFADRVRNELERHKSGLSTPVNLSNSPPTPDPSPPTRGGDGLTIPRPAIAKYESSYAESFQTATEGRTSQASMLPAGVESTPKQSWLDMARAMGLSNQVLAMQDEERVVNDTDSDKAARVKNYHLESDGSEGEKDNKTASYDASDERSTSDSNAKSSPVTRSVSQKLAVTAVPRLRRPFQPGDEIKRPDDFLLYASTGSIKKWKKPERMSKTPPPREMDSEFLRNAEPVFQPAAPEAPESGKDILQRLEDVIREDNADSAPLTALPPIPAEERQILTPREEDVKPLKEFTPSPPANTIPTPPSTAQKEKSLEENNVVYKMIQEENAKRHSAISDGSVPARVVAPAENKRKLRHSQKRDSLRGDVSLISSSDSHRLRHKRAMGSLGHSEDVASAKENVLSNGRPSTRGTEGKSADPVPRASRSTGEKIFSYSKMRDRSPKVAPASVLGNPHKLRRSLRHSSLDHTPRSVSDSARPLSVNTHAFLAEAEAAPPSPRLRRSFAENRLDRNNEVRRTSLGHVPEVTLAPVEPVQPAITEQPMNDVFKEETQQSPRLVTSERMTRRDRPESSEVSFNTTQSPRRKSMDYRSLHPTITPMSQASGTAMSEAELCEAKGVTFFPHNNKSLLVVQTARHVSIPDYPPIDEDDSDIDGNPIPHIKSPRFKAFVTPADEIGKPLHSVDSPLTNPRAAPEPPVIKFIPPTPADELERELGSEEVRRKENSRPGLQRSLSLKDRIRRFGDALNQPVPFGRQNSYRRSAPPRRIPQAEVQQRPTYLSSFWQPRDFWEGYSDDSDVEDEEDFEPLPAGGDTSDVGEEQQQKRRSAAFFLPRAMSKRLPGFRGSGGFLMGNSLGIDRHGSNNRRHYVERRANASHPSLVLRPDLIPDQHHRQPSSTSSMPSLRNRHSEELLRRLSISGPPQQQRRIIFKVPFTGGRRIEYVGFSVLGKKLAAKKKRAAERQAEERRMRVRESIGPRVYHEG |
| 493 | Unigene0011785 | 24 | 32255 | 2.3 | MSRPSTARGVSTTNIQGGSWRVLEGKIAIVTGASRGIGAAICENLAAKGASLVMNYTSDKSAAKTEELAKRLQEEHGIQTLPVQADMGNVNGPAHVINIAKNNFAHPKSGKFQIDIIINNAGVASKTDIEDCTPEEFDRLYHVNVRGPLLLIKAAVQYLPHDRSGRIVNLSSVSSSLGFNGDGMYGGTKAALEAMTRTWARELSERATVNAVNPGPVATDMYAGTTPEFQGKMAGWTRNTPLAAIRPDIDRKDLVDNAELAGGRPAYDYEIAGVVAMLCTPDSAWCTGSVVCANGGFKFSY |
| 494 | Unigene0004538 | 24 | 62413 | 1.2 | MSGNGPLYMGFDLSTQQLKGIVVDSNLKLIYEAKVDFDADLKKYGIQKGVLTNPAEGEVFAPPAMWLEAIDLVLDRLKNAGLDFGHVKGLSGAGMQHGTVFWSYDAESLLADLNPQHSLANQLQSGEGGKSAFAHEMSPNWQDASTQEQCDAFDAALGDAETLALVTGSKAHHRFSGPQIMRYRTKWSSHYKKTARISLVSSFLASVFLGRFAPIDISDVTGMNLWNIKDGKWDDALLELAAGGKSGIDDLRNKLGVVSEDGGQWLGTISSYFVAKYGFAKDCQIIPATGDNPSTILALPLRASDAMVSLGTSTTFLMSTPEYKPDPAYHFMNHPTTPGLYMFMLCYKNGGLAREQIRDQLSTSSDWESFNSTALSTPALSQRSPSEPLRLGLYFPRPEIVPNLPSGQWRFKYNPETQDLDEESSDFLPDDARNIIESQFLSLRLRSQALVHAEKDPATGKTLPPQPRRVYLVGGGSANPAIAKIAGEVLGSVEGVYKLDIGGNACALGAAYKAVWACEKAEGQTFEDLIGSRWDESSFVKKVADGYKEGLFEQYGLAVKGFEQMEKAVM |
| 494 | Unigene0004537 | 24 | 64199 | 1.2 | MSGNGPLYMVSASMRAATAHGUADSAUGFDLSTQQLKGIVVDSNLKLIYEAKVDFDADLKKYGIQKGVLTNPAEGEVFAPPAMWLEAIDLVLDRLKNAGLDFGHVKGLSGAGMQHGTVFWSYDAESLLADLNPQHSLANQLQSGEGGKSAFAHEMSPNWQDASTQEQCDAFDAALGDAETLALVTGSKAHHRFSGPQIMRYRTKWSSHYKKTARISLVSSFLASVFLGRFAPIDISDVTGMNLWNIKDGKWDDALLELAAGGKSGIDDLRNKLGVVSEDGGQWLGTISSYFVAKYGFAKDCQIIPATGDNPSTILALPLRASDAMVSLGTSTTFLMSTPEYKPDPAYHFMNHPTTPGLYMFMLCYKNGGLAREQIRDQLSTSSDWESFNSTALSTPALSQRSPSEPLRLGLYFPRPEIVPNLPSGQWRFKYNPETQDLDEESSDFLPDDARNIIESQFLSLRLRSQALVHAEKDPATGKTLPPQPRRVYLVGGGSANPAIAKIAGEVLGSVEGVYKLDIGGNACALGAAYKAVWACEKAEGQTFEDLIGSRWDESSFVKKVADGYKEGLFEQYGLAVKGFEQMEKAVM |
| 495 | Unigene0010870 | 24 | 57150 | 1.7 | MASFMRNAARLQPALRSSIRPATIVRRTMATQSPSHNASAAAAVRSAVSDSAEPSLFPDEPTGPIVRTQIPGPKSQEAIAELDEVFDTRSLNMMANYQNSFGNYLADLDGNVLLDVYAQIASIPVGYSNPNLLAAATTPEMASAIINRPALGNFPQHDWAHILKTGILKAAPKGCDQVFTAQAGSDANELAYKAAFMWKRRQERGGPEVDFTAEEISSAMNNQSPGSPNNMSILSFRTAFHGRLFGSLSTTRSKPIHKLDIPAFDWPQASFPALKYPLEEHAEENAKEEARCLAEVEDLLLNYHNKPAAVIVEPIQSEGGDNHASPAFFNGLRDVTRKHGVLLIVDEVQTGVGATGKFWAHEHWNLKHAPDMVTFSKKAQTAGYYFSNPELRPNKPYRQFNTWMGDPARALLFRAIINEIERLNLVENTAITGEYLYNGLESLGKQYPGEIENLRGKGQGTFIAWDSPRRDEILKKAKGVGINIGGSGERAVRLRPMLIFQKKHADIFLDALEKVFKS |
| 496 | Unigene0011934 | 24 | 18960 | 6.1 | DSVASFRRIPWTARLLDEPDVIARVPNSRLFKRDTEDSLYAEILKTDRTIRNCIVVYRKSTSPEDEISEATTLYEVGDGMNGHPEIIHGGITATMIDESMGIMQMENLERKRRKEKEVAEEERREPSEGLSAFTAYLNVQYLKPVRTPAVIAVTARRMKKEGRKE |
| 497 | Unigene0001598 | 24 | 14964 | 5.7 | MSANKRGGTSGQKLKMTLGLPVGAVMNCCDNSGARNLYIISVKGFGARLNRLPAAGVGDMVMATVKKGKPELRKKVMPAVIVRQSKPWRRADGIYLYFEDNAGVIVNPKGEMKGSAITGPVGKEAAELWPRIASNAGVVM |
| 498 | Unigene0010818 | 24 | 65589 | 1.2 | IMTTPTSEEKTPYHNEYGGDDEKQRRTSLADSAAGTDEELMAVRTQAWGKNGLIYMWIGIGLMWTNFELDNATIYNYQNFATSEFQQVSLLGALSTAGTIVSAVLKPPVAKVSDIIGRAETYCFAVALYILSYILCASSNEYNQYAASYIIYCIGQTSMQILNQLIVADITTSRWRGLANGLVNLPFMIIPWIAAFIADSAINTVGWRWGIGMFAIIMPVCSVVVIVPLLYFQRRMKQFGHSTKAQATFISFFSKIDVGGMTLLVAGFALVLLPLALAGTTPSRWDTAWVPALIAVGGVCVIALVGYEWKIAAHPLVPLHFFKNISLVLAWIMGLMDAFAFSATHTYIYTWAVVVHEFSVRDASFLTFTAGCMQVLTGLVAGALMYKTRRYKWLLVIGVTVRLLGYGVMLRLRGANNSVAELFIVQLIQGFGSGIVLGIFLVVAQIVVPRSELSQSTALELLFIYMGNALGSTAAGAIYTNSFAPRLRDYLPNSTDELVNTILNTFTEEPFANGTPERTAINAAYNDVVRYMTYAALAASIFGIILVWFLPNLQLSDKHNLAGEMEQIKAESSKRADEGILRWWWRTGRLW |
| 499 | Unigene0005231 | 24 | 70132 | 1.5 | MAQQQKRKQSSLPAGYKEDHSKGAMLRFEESLPRLPVPTLEETAKRYLKSVHPLLTPAEYDNTKMAVEAFVKPGAVGHTLQDRLKARAADPGTKNWLSEWWNEAAYLGYRDPIVPYVSYFYSHRDDRKRRDPAKRAAAIAFATLEFKKQVDTSALEPEYMKKLPMAMNSYIWMFNACRRPAKPSDYPVKYEAEQNQHILVIRKNAFFKVPHTHNGQNLSAKELEYQFRRVYEMAEKAPAVGIMTTENRDNWADMRERLVKADPANQVTLEAIESASFVVCLDDATPVTLHERSRQYWHGDGSNRWFDKPCQFIVNDNGTSGFNGEHSMMDGTPTSRLNDTILHWMFNDKLDFENPQFRSDIPDPAPLKFKLSGENYADIALAQTHHIGLMSQHELRVEAFQGYGKGLMKKFKCSPDAYVQMIIQLGYHKFYGKNRPTYESAATRRFQEGRTETCRTVSDEVVAFCNAMAHHLSTDEECRDLFRKAIGAHIEYITAASDGKGVDRHLFGLKKLLKDGEEVPQIFKDPAYTYSSSWFISSSQLSSEYWNGYGWSQVIDDGWGIAYMINENSVQFNIVSKNLGAERMAFFLNEAAQDIRAILESENTSAKAKL |
| 499 | Unigene0005232 | 24 | 70132 | 1.5 | MAQQQKRKQSSLPAGYKEDHSKGAMLRFEESLPRLPVPTLEETAKRYLKSVHPLLTPAEYDNTKMAVEAFVKPGAVGHTLQDRLKARAADPGTKNWLSEWWNEAAYLGYRDPIVPYVSYFYSHRDDRKRRDPAKRAAAIAFATLEFKKQVDTSALEPEYMKKLPMAMNSYIWMFNACRRPAKPSDYPVKYEAEQNQHILVIRKNAFFKVPHTHNGQNLSAKELEYQFRRVYEMAEKAPAVGIMTTENRDNWADMRERLVKADPANQVTLEAIESASFVVCLDDATPVTLHERSRQYWHGDGSNRWFDKPCQFIVNDNGTSGFNGEHSMMDGTPTSRLNDTILHWMFNDKLDFENPQFRSDIPDPAPLKFKLSGENYADIALAQTHHIGLMSQHELRVEAFQGYGKGLMKKFKCSPDAYVQMIIQLGYHKFYGKNRPTYESAATRRFQEGRTETCRTVSDEVVAFCNAMAHHLSTDEECRDLFRKAIGAHIEYITAASDGKGVDRHLFGLKKLLKDGEEVPQIFKDPAYTYSSSWFISSSQLSSEYWNGYGWSQVIDDGWGIAYMINENSVQFNIVSKNLGAERMAFFLNEAAQDIRAILESENTSAKAKL |
| 500 | Unigene0012321 | 24 | 99139 | 0.7 | MCRKEADTADANMDISKGREVLPKNVKPLHYNVTLEPNFETFKYEGTVEIELDVVEDTKSISVNALELDIKETKIEAGGQTITSSPTLSHDEDSQTTKIDFDQTIPAGQKAKLIHKFTGSLNDNMAGFYRSSYKGPNGEDRYIATTQMEPTDCRRAFPCFDEPALKATFTVTLVADEKLTCLSNMDESGTKKLDNGKKAVTFNKTPLMSTYLLAFIVGELQVVETNDFRVPVRVFCTPDKNIEHGQFSLKLAAQTLAFYEKEFDSKFPLPKMDMVAIPDFAAGAMENWGLVTYRVVDLLLDEKHVSASTKQRVAEVVQHELAHQWFGNLVTMDFWDGLWLNEGFATWMSWYSCNVFYPEWKVWEGYVTDNLQSALGLDSLRSSHPIEVPVKRADEINQIFDAISYSKGSCVIRMVSKHLGEDVFMEGIRRYLKKHAYGNTTTGDLWAALSDASGKDVERIADIWTKNIGFPVVTVTEDAKNSKIHVKQNRFLRTADVKPEEDQTLYPVFLGLRTKNGVDEELTLNKREADFAVPDLDFYKLNADHSGIYRTSYPAERLQKLGQNAKAGLLTVEDRAGMIADAGALSAAGYQKTDGLLSLLKGFDKEPDMVVWDEITARIGALRSTWIFEDEKPKEALKAFQRNLSSQKAHELGWTFTGNEGHIEQQFKALMFGNAASAGDEETKKAAFDMFGKFVAGDRAALHPNLRASVYAIVLQYGGKAEYDALVKEYETATSSDERNAALRSLGRARDPELIQRTLAYSIGKHVKEQDIYLPLAGLRAHQEGIEAFWAWMKENWVLLKEKMPPSFTLLGSVVSMATSSFTREDQLRDVEKFFEGKSTKGFDRNLAQSFDAIKAKIGWLQRDKAAVEKWLKDEKYL |
| 501 | Unigene0007360 | 23 | 14205 | 7.1 | MSSKTQTKKPAGKSGRSAIEDVVAREYTIHLHKRVHGVSFKKRAPRAIKEIRAFAEKAMGTSDVRLDPQLNKKVWESGIKGVPFRLRVRISRKRNDEEGAKEKLYSYVQAVNVKNPKGLLTAVVED |
| 502 | Unigene0010961 | 23 | 30043 | 3.2 | MTSIGTGYDLSNSVFSPDGRNFQVEYAVKAVENGGTAIGIRCKDGVVLALEKLVTSKLMKKDANNRIATVDRNIGVVHSGLLPDGRHFVSRARDEASQWRGTYKAPIPTSSLANRMGAYVQAYTLYSSVRPFGITAIVGGWDAETEVDVDGAVGSGPKVGAGGKTQGVKEGGPYLYMIEPSGLYWGYYGAATGKGRQTAKAELEKLKLDPKDGECISLEQGVKEAARIIYAAHEDSKDKEFELELTWISSVNGPTKGRHEAVPKEMKEEAERLAKIAL |
| 503 | Unigene0009436 | 23 | 29405 | 7.2 | VKYPYDRRIIRELPLIGKGAKNSSICHVQNYLKQYGYLSRECDCDGGSLDEVTSDALATFQKNFNLTDSPVGEFDNDTKQTMVKSRCGLPDILSELDFRTTGPWTRSNLTYAFGGLSRDLPTSSSRDAVRRAMDTWTNAGVGLTFVEVLAHQQPDLSIEWRPAADPDHSMVGGVLAHADFPPGFSIIVSRPPLPLHFDDSEHTWVDGTEANGFDIQTVALHELGHCLGLFHTSVAGSVMFPTTSSGHTMRELKDDDEAAIRSLY |
| 504 | Unigene0001564 | 23 | 34550 | 2.6 | MVAIAIAGASGSVAQEVIDGLVATGKHEILLLSRNPPKTESRSGCRWLQTSYTDTAQLAGALQGIETVLSFITTQSDPGNATQRALIDASILAGVKRFAPSEWATSHNTHMPWYSGKDDIRAYLESINQTEKRLSYTLFQPGLFPNYFLYPHKHPSSPHFNSFETHIDFANCRALVLSPDGENDKLTLTTLEDLVKVVVRAVEYDGEWPIIGGIKGTEISVGELIKIGERVRGRPFDVEYLQISDLEAGNITSSWLPVIDHPAFTPEQARALAEKLLSGMVLGIHAGALNVSDEWNKRLPDMHFTQAEAFLKE |
| 505 | Unigene0001312 | 23 | 84621 | 1.2 | TGDVHKHDIEGGRGRAAVEALKEDTGIHHDTAWASANAVPPAATLAEVPRHENGIGHGPQVRSTTEGLDVVCGPLLNYKHTSNEHTDQPQWHGSVLIVTTPGQQPGPLTLRCVGAWNGGAAATNGAVAGGQDRTFQAEKLYEDPTRGFWRYVIDVPFQEAEATWAYSIRDMVSSTDKSPITKPLRFVVPSKHESMRIMFHSCNGFSVGTDMNTWTGPVLWNDVLRVHNEQPLHIMIGGGDQIYNDSVRVDGPLRPWTDIANPKKRRDFPFNEEMRKKCDDYYYENYVRWYGTEEFALANGQIPQLNIWDDHDIIDGFGSYTDHFMKCSVFRGIGGVAHKYYLLFQHHIPPPPSTYSTDAPQTTKTTGHTTSPDAVQLKDTFVMVPKREDPSWIIGTRPGPYVEERSRSIYCQLGKRIAFLGIDARTERTRHQINYPETYDQIFKRANDEIARSNGAIKHLILLLGVPIAYPRLQWLENIISSPIIGPIKFLNKRFGVAGSLFNQFDGGVDLLDDLDDHYTAHQHKKERKALIHRLQQISKDHSVRITILGGDVHLAALGRFYSKAHLNIPAERDFRYMPNIVSSAITNKPPPQAVANLLARRNKIHHLDHETDETLLEMFNKNPGAGEEGVEDKTAKNNHATMPSRNYAIITESHSHTGAPANGLATNGTIDGVGDPSNNADFTLPKNMREPIHKGEVQAGTEHPAAGGIKKTGLAGRDGLDVCIRVEVKPGAKDGRTEGYGISIPALESGAYKGQGTTW |
| 506 | Unigene0001424 | 23 | 93580 | 1.1 | MVNFTVEEIRTLMDDPKNIRNMSVIAHVDHGKSTLTDSLVQRAGIISAKNAGSARFTDTRADEQERGVTIKSTAISLYGSLSDQEDLKDITISTDKSEKNDFLINLIDSPGHVDFSSEVTAALRVTDGALVVVDTIEGVCVQTETVLRQALGERIKPVVIINKVDRALLELQLSKEDLYQNFSRVIESVNVVIATYYDKALGDVQVYPQKGTIAFGSGLHGWAFTIRQFAVKYAKKFGVDKNKMMDRLWGDSFFNAKTKKWTKSPEGAERAFNQFCLDPIFRIFDSIMNFKKDEIPKLLEKLEVKLVGDEKDLEGKALLKVVMRKFLPAADALMEMMILHLPSPATAQRYRMETLYEGPPDDASAIGIRDCDPKGPLMLYVSKMVPTSDKGRFYAFGRVFSGTARSGLKVRIQGPNYVPGSKSDLFIKSIQRTILMMGRYTDPIEDVPAGNILGLVGIDQFLLKSGTLTTDETSHNLKVMKFSVSPVVQRSVEVKNANDLPKLVEGLKRLSKSDPCVLTFISESGEHVVAGAGELHLEICLKDLEEDHAGVPLRISDPVVQYRETVGGDSSMTALSKSPNKHNRLYVTATPMAEEVAKDIESGKIGPRDDFKARARILADDHGWDVTDARKIWCFGPDTNGANLLVDQTKAVQYLSEIKDSVVSGFQWATKEGPVAEEPMRNVRFNIMDVTLHTDAIHRGGGQIIPTARRVLYAATLLADPGLLEPVFLVEIQVPEQAMGGIYGVLTRRRGHVFEEAQRPGTPLFNIKAYLPVNESFGFNADLRSNTSGQAFPQSVFDHWQILPGGSALDPTSQPGKIVEDMRKRKGIKPQVPGYENYYDKL |
| 507 | Unigene0011006 | 23 | 62515 | 2 | MASTLTPVNPPLPGTNPAEAFQLARESEKPDLEQQLFDEQIQQVKQWWASPRYKGIKRPYTPEDVVSKRGTLQQVYPSSLTARKLFNLLEERAAEGKPVHTMGAIDPIQMTQQAPHQEILYVSGWACSSVLTTTNEVSPDFGDYPYNTVPNQVQRLFKAQQLHDRRHYDERMKLSPEERRKKPYIDYLRPIVADGDTGHGGLSAVLKLAKLFAENGAAAVHFEDQLHGGKKCGHLAGKVLVPFGDHINRLVATRFQWDLMGCENLVIARTDSESGKLISSAIDTRDHEFILGVTEDVEPLADTLQTMELEGASGPEIDKFEAEWVKKHKMVTFDEAAEAHIKASGKDAEAYLKQTRENRNLPLHKRRQAAAAVAGSPVLFSWDVPRTREGFYHYKAGLAAATKRAIAFGPYADLLWLETKDPNVKEAASFAKEIRDVLPGKKLVYNLSPSFNWSAHGFTDAQLKSFIWDLAEHGFVLQLISLAGLHSTATITHELARAYKTEGMKAYVETVQRREKETGCDVLTHQKWSGATYLDGIIGAIQAGSSGSRSMGEGNTEGQF |
| 508 | Unigene0012922 | 23 | 42603 | 2.1 | MADSKIVSNTATLEKYLKLDQRDNIIAEYVWIDASGGVRSKCKTLPKSSYTGLDSLPEWNFDGSSTAQAPGDNSDVYLRPVAVYPDPFRLGDNILVLAETWMSDGKPNAFNFRHDAAVVMEKHKGEKFWFGLEQEYTLLDFEGWPYGWPKGGFPAPQGPYYCGVGTGKVFCRDIVEAHYKACLYAGINISGTNAEVMPAQWEYQVGPCEGIDLGDQLWMSRFLLHRVAEDFGAKITFAPKPIPGDWNGAGLHTNVSTEAMRNEGGMKAIEAAMEKLAGRHKEHMLVYGEGNEARMTGAHETASYDKFTWGIANRGSSVRVNRAVAEEGKGYFEDRRPASNGDPYQITGMIVETICGKVEGADVFKKSQQPAEQVDTVVPIVKP |
| 509 | Unigene0000781 | 23 | 31715 | 3.2 | AYSLVTRKPLNNGHSMPSIHLGVYLMSGKEASQAVGHALDAGYRAVDSAQMYHNEREVGNAVQTWLKSHPEVKREDIHYTTKLASNGDYERARKSITKSVKECGLGYVDLFLLHSPYGGKQARLESWRAVEDAIEAGEVRSGGVSNYGEKHIDELLSSKPRILPTVNQIEVHPFNTRSALTQYCAKNNIVVEAYAPLARALRMKHPVIVELAKKYGCTSGQLMVRWSLQHGYVPLPKSVRKERIIENAQIEQFEISEDDVRRMDELDEYLVTDWDPLDAD |
| 510 | Unigene0014245 | 22 | 64391 | 1.6 | MANPPHGGVLKDLIARDAPRRQQLSEEAEKLPAIVLYERQLCDLELILNGGFSPLEGFMSEKDYNGVVENNRLADGNLFSIPINLDVNQNLIDEVGIKAGARITLRDSRDDRNLAILTVEDVYRPDKQKEAKEVFGGDPDHPAVKYLFDQTGEFYVGGKVEAIDRLQHYDYVGLRYTPAELRAHFDKLGWSKVVAFQTRNPMHRAHRELTVRAARQRQANVLIHPVVGMTKPGDIDHFTRVRVYQALLPRYPNGMAVLGLLPLAMRMGGPREAIWHAIIRKNHGATHFIVGRDHAGPGKNSKGEEIYGPYDAQYAVEKYRDELGIEVVPFQQMTYLPDTDEYRPKDEVPKEIKTLDISGTELRRRLRTGGDIPEWFSYPEVVKVLRESHPPRNKQGFTVFLTGLYNSGKDAIARALNVTLNQQGGRSVSLLLGETVRSELSSELGFSPEDRDKNIQRIGFVASELTRAGAAVIAGPIAPYAKSRKAAREIVEKHGSFYLVHVATPIEHAEKTDRRGVYAKARAGVIKGFTGVDDPYEEPQETEADLTVDLTKINVRTAVHQIVLLLEAEGLLDQL |
| 511 | Unigene0013848 | 22 | 53708 | 2.5 | MAPQLQNFYQTVDSLAEPFIDRLRKAVAIPSISAEEERRPDVIKMGHWLKEQLEGLGAHMEARELGPQPHKEHLTLPPAIIGRYPAQKDPSKRTILVYGHYDVQPADLSDGWATEPFELSVDDKGRMYGRGSTDDKGPVLGWLNVIESHKKAGIDFPVNLLMCFEGMEEYGSEGLDDFIIAECKPGKFFEDADAVCISDNYWLGTEKPCLTYGLRGCSYYSIEISGPGQDLHSGVFGGTAQEPMTDLVRVMGSLVDTDGNIQIEGIKELVAPLTKEEESLYTNIAFTMDNLYESLGSQTGIFPDKERTLMGRWRYPSLSLHGVEGAFSAPGAKTVIPAKVTGKFSIRTVPNMEPEDVDKLVYAHVDKVFKSLNSKNKINCYLQHAGKWWVASPKHWNFTAAAKAVEDVWHTKPDLTREGGSIPVTLTFEQATGKNVLLLPMGSSTDAAHSINEKLDRRNYIEGIKLLGAYLHYVAEEPMEG |
| 512 | Unigene0007985 | 22 | 22213 | 4.4 | GHTFSLMPLGDSITWGTGSSDGNGYRGNLLWALGRYGARVDFIGSQHNGKMNDNDNEGYPGRWIHDIQGFAEAAHTHGYNPRVILLHAGTNDCQQNPVSGDDLRARLETLTRRLTTLWPQATVIVASIIASTDANVNANVQKLNAQIPGMVQRLARQGMRVAHADMSKALTPADMSDKLHPNDKGYVKMSHVWLGALNDNSGKG |
| 513 | Unigene0002722 | 22 | 40855 | 2.7 | SATMTGSQVAPKRLAIGVVGLGRIGRQHALNALHYVPRTELLCACSPMDADHEWANQNLVPYGVKIYRSFDDMIQHEGLEALLIASTTVVHYEHVVAGIEKGLHILVEKPLAMNSEQSRKILEKANQQQYADLKVMTAFSRRFDASYQNAVTAIQQGRIGTPAMIRCDNRDKYDRSEFYMRYIMANPGIFIDTGVHDIDLTFSFLGSEARLKSCHAIGTIALHKELAQIQDVDNAVGTVEWYPSQPGGIAPISYYTISRISQPGLDNPTEIIGTEGTLKINLHPRRDLVEVADKNGIGNGVMPDFYERYEKAFVTELSVFADAVLDDKPLPYELDVAIKGMELAEALQESLRTGKKIEWDENGQR |
| 514 | Unigene0005274 | 22 | 35966 | 2.5 | GLGDSSMLERIDALFACGAGEHINIPQIVVVGDQSSGKSSVLSGLIRKDLPRDSGLCTRFATKIVFRRAKERGIRASIIPDEDAGVEHRSAIAAWGELSVDSFDDFTFADIMKEVHNVIGIASPNDVTSGTERKPTFSGDVLQLEISGPDEEHLSVIDVPGIFRDSTPGLTTKADIQLVRSMVTRYMENPRSVMLTVVPANVDVATQEILHMANELNPEGDRTLGVLTKPDLVDAGAEHRIIDLVEGRARQIKLGWHILRNPGQNDLGNPAFDRQREEIDFFRTKRPWSDLNHENVGIEALRNRLKDVLSGLIRREFTNVRIRL |
| 514 | Unigene0005275 | 22 | 81159 | 1.1 | GLGDSSMLERIDALFACGAGEHINIPQIVVVGDQSSGKSSVLSGLIRKDLPRDSGLCTRFATKIVFRRAKERGIRASIIPDEDAGVEHRSAIAAWGELSVDSFDDFTFADIMKEVHNVIGIASPNDVTSGTERKPTFSGDVLQLEISGPDEEHLSVIDVPGIFRDSTPGLTTKADIQLVRSMVTRYMENPRSVMLTVVPANVDVATQEILHMANELNPEGDRTLGVLTKPDLVDAGAEHRIIDLVEGRARQIKLGWHILRNPGQNDLGNPAFDRQREEIDFFRTKRPWSDLNHENVGIEALRNRLKDVLSGLIRREFTNVRLEVQKNLDSAESKLKALGPERNSRAAQAAYLTGISTRFQHLVDMALSAKFGTDGLFEEHDDMKIAPAVVLRMEEFGTDMMHFGHQHSFQAREQHQRGNLGFQKDNRVTNHRHPQNIPSSSCVRKWTKSTDLEGLLSPPKTLHRSHGERTDKWLQELYRHSRGFELGTFDPVILASAMRKQTINWESISFGVVSDIAVITHAFISKALVCLCPNERFRDDLYNLLFEDLKTRYEKATQQLRFILTVELEGTPMTQSHYFIDLLEESRQARFIANLEDLAINDNEHGKVVRLNELMMKHPMSNDQYLVRDIHDILQSYYEVARKRFTDNVCKQAIDYFLVTGPDTPLRVFSPIFVSQLTDEQLERIAKEEPQVHKQRSKLVNDIDSLRKAKRVLA |
| 515 | Unigene0010436 | 22 | 35954 | 2.1 | KLDWPIAKVPSLAEHFGAPSQGPFNKETQMVPIIGLESASERVNAMNLPQPDPFSQPSLLVSNQSLNTFTSTQPPALVKAIGASLGLGSNELHNIVNWELELFDTQPATVIGLHKELISAGRIDDKLCSWAALQALIESSSYGTEDSSLIKVVGLFDDEEIGSLLRQGARGNFLPITLERAVGALSGHTPGSDLMGRTYANSFMVSSDVTHAVNPNFTEVYLANHAPHLNVGVAIAADSNGHMTTDSVSTTILKRCADRVGAQLQVFQIRNDSRSGGTVGPMLSSAMGMRAIDAGLAQLSMHSIRATTGALDPGLGVQLFKGFLDEFEEVDREVRD |
| 516 | Unigene0002238 | 22 | 43788 | 2.3 | MATEAVSTAPAAADDNSHIVIKARHETANTQTFHVGTRRSILARKQADEVVKALREAWPEHNFEIHAMSTTGDNNQKTALHKFNEKALWTQELEVLLEDGSLDLIVHSLKDMPTQLPVGLSLGCVTQREDARDALVLKPGLVDKVKNLKDLPAGAVVGTSSLRRTAQLKRFYPQLQFQDVRGNIGTRLAKLDDPNSDYSAICIAVAGLERLGLSHRISSYLSKENGGMLHAVGQGALGIETRSDDERTKELLSKIGDDRSWRQALVERSLMRTLEGGCSVPIGVETEWIAKSRMHDQHAGVGIKPAEDYNQLSGEATTAGGTKQLEVERSDELLMRAIVVSLDGKDAAEVETRRRIITHEQAHEFGFDVAKMLVEKGADKILEQVQLNRGIIKSQGGA |
| 517 | Unigene0004321 | 22 | 42487 | 3.3 | MAAALPPKLKAAAPDVGRFATRAAQLEKFRPIVAYWCEYYILQAILSRQLHTTDEECTTYALGLMDKLEAYKAENATNDAVVDDVAAKAYIENFALETFSRGEDAQRNNKATKQTADTFQASATFMDLLTIWGSLETEFAAKSKFAKFHALRIARAIKAGEDPNASNPVIEEPPSAPQAPAEDDLETQLKALENEEKAHQPPTVESAPDSRIPSRPQSTFQTSPLAQPTLPTIDVPPTQPAEHDVSPIEPADVNSRAGSVGGGYFPAVPGEPSDPAAPIEPPHISSDPADFYNAPQLPPSAPTSEDFSGLPQGSAASSHAAPPPPAALPAFSPPPAAVPVPPLRVPAPTTRPTGGYNTDDESIMAAQKHAKWAISALNFEDVDTAVKELRIALNSLGAS |
| 517 | Unigene0004320 | 22 | 44535 | 3.1 | MAAALPPKLKAAAPDVGRFATRAAQLEKFRPIVAYWCMUTFKRWYRLRUPGPGEYYILQAILSRQLHTTDEECTTYALGLMDKLEAYKAENATNDAVVDDVAAKAYIENFALETFSRGEDAQRNNKATKQTADTFQASATFMDLLTIWGSLETEFAAKSKFAKFHALRIARAIKAGEDPNASNPVIEEPPSAPQAPAEDDLETQLKALENEEKAHQPPTVESAPDSRIPSRPQSTFQTSPLAQPTLPTIDVPPTQPAEHDVSPIEPADVNSRAGSVGGGYFPAVPGEPSDPAAPIEPPHISSDPADFYNAPQLPPSAPTSEDFSGLPQGSAASSHAAPPPPAALPAFSPPPAAVPVPPLRVPAPTTRPTGGYNTDDESIMAAQKHAKWAISALNFEDVDTAVKELRIALNSLGAS |
| 518 | Unigene0003355 | 22 | 43211 | 2.7 | VSQREYGEETVFWSMKQAEQFSARRRTLSIFFADDGFEGCESQTWIDNTPKLWQDLHNVLRFCDPHSIALNIDSEIAFAGGLHAGEYKKLLEVVPAPWNERFVSKPLVAVEYIATMPKSRLHWYRKLMETAWAVIDEGFSERVIFPGRTTAEDVEWWFREKLQSLNYTTWFHPDVTILPGWLTAEDKLQRSSAEKENTIQHGDLLHVDFGLTALGLNTDTQHLAYVLPPGASESDIPASLLSGLKKGNKLQDIVRSHMIPGLSGNEILRNALESMREANISGKIYSHPIGDWGHSAGTLIGMTNLQDGVPVLGDLPLLKDMYYSVELYVEHWVEERNETIVFPLEEDVYWDSEKESWEWVWGRQEKFHLVRSKER |
| 518 | Unigene0003356 | 22 | 50358 | 2.3 | IISIMLKSVSLLLCFVLGVAASDPKVHHLPTLREQAKIQDSWRQERLVTIPAILRKHGVDAWIVSQREYGEETVFWSMKQAEQFSARRRTLSIFFADDGFEGCESQTWIDNTPKLWQDLHNVLRFCDPHSIALNIDSEIAFAGGLHAGEYKKLLEVVPAPWNERFVSKPLVAVEYIATMPKSRLHWYRKLMETAWAVIDEGFSERVIFPGRTTAEDVEWWFREKLQSLNYTTWFHPDVTILPGWLTAEDKLQRSSAEKENTIQHGDLLHVDFGLTALGLNTDTQHLAYVLPPGASESDIPASLLSGLKKGNKLQDIVRSHMIPGLSGNEILRNALESMREANISGKIYSHPIGDWGHSAGTLIGMTNLQDGVPVLGDLPLLKDMYYSVELYVEHWVEERNETIVFPLEEDVYWDSEKESWEWVWGRQEKFHLVRSKER |
| 519 | Unigene0011518 | 22 | 36527 | 2.6 | MQSIRQPLTRALRNAPAGLTKSVAPASYATISAQDAPGQPSGYAKTGLNLRITKDTKVLFQGFTGKQGTFHAQQAIEYGTNVVGGTNPKKAGSTHLERPVFATVEDARKQTGADASVIFVPPPLAAAGIEEALQAEIPLAVCITEGIPQHDMVRITDMLKTQDKTRLVGPNCPGIIAPGQCKMGIMPGFIHKRGRIGIVSRSGTLTYEAVNQTTLAGLGQSLVIGIGGDPFSGTNFIDCLRVFLADEDTDGIIMIGEIGGSAEEDAAEFLKEYNTGGKDGRGKPVVSFIAGISAPPGRRMGHAGAIVSGGKGGADSKISALEGAGVTVEKSPASLGKTLRDQFIQRDLL |
| 520 | Unigene0011505 | 22 | 22307 | 5 | AYPPGGTYFDGKKSFTEVNIDASKGDAINTTEFLEAAEALTGLFDVLGGVAFGPVKSDIGGNIKKVRDRQLAAPVEGETLQDLVRNELKTKKHTATEGLVWLNRGLDFTAQSLRRNFDTPTEELAASFRDGYGKTLKQHHSFLVKPIFSAAMSATPYRKDFYAKLGDDQERVNKELNEWLKGLEKCVAILNTFLASKEAKW |
| 521 | Unigene0009716 | 21 | 37522 | 3.9 | MAASRSSDAFKFLPQGGLIQEFNVGGRNIVLGFESPEPYKHRSSQFFGENIGRVANRISGAKFSLNGKSYDVAANDGVNALHGGREGWGKKTFDGPHNVSRNGRDALEWKYVSKDGEEGYPGTVELRMWYYPTVEKDDGKEKTSLEIEYEVELIGDEVEETVVSLTNHSYFNISDGPTYEGTRVVTQTNLHLPVDAGSIPTGAIEPYPEFEANKEFILGASKPDPDHCFIMNPDPSSIPLDTRNEPMKKLIELSHPNTKIHFEALSTEPAFQFYAGRGIQAPPAKEGLPSRGPRSGLCIEASRYVNAINQEEWRQQVVLKKGQLWGSRTIYRAWVE |
| 522 | Unigene0006770 | 21 | 56960 | 1.2 | MRLPFSSSDEKAAHSPTSSERFERPNDITYQPEGYDEDLHVECPPHTTERKLVTRIDIHIIPVLCILYLLAFLDRVNIANANVLGLSKELGLEGNEYNTALVIFFVPYVLFEIPSNILLKKFRPNVWLSLNMFLFGFVTIMQGLVKNYESLLVTRFFLGLFETGMFPGCFYLIGMWYRRHEAQKRYTFFFSSTTLAGAFGGLLAAAIGKMDGLQGYQGWRWIFILEGALTVAVSFLFFFLIPSFPEDSKWLAEDEKAYVAARLQKDQGRSAVERSITAKDVGNVFKDYKVIVAGFMYFGLIVPAYGYAYFAPGIIQSYGYDPIQTQLHSVPPWAAAFGYAMLLAVLSDATKHRFGFAVFSICISIAGFAILISVHNNNTVQYAALFLVTMGTYSAMPVIVCWFNMNLGGHHRRSVGSAWQVGFGNIGGIIAVFAFLRKDAPKYVTGYSICIAFTILSIISCIIYGFACWSQNRQRDKSVTDVGLTEHEKTELGDLSPTYRYLL |
| 522 | Unigene0006771 | 21 | 56960 | 1.2 | MRLPFSSSDEKAAHSPTSSERFERPNDITYQPEGYDEDLHVECPPHTTERKLVTRIDIHIIPVLCILYLLAFLDRVNIANANVLGLSKELGLEGNEYNTALVIFFVPYVLFEIPSNILLKKFRPNVWLSLNMFLFGFVTIMQGLVKNYESLLVTRFFLGLFETGMFPGCFYLIGMWYRRHEAQKRYTFFFSSTTLAGAFGGLLAAAIGKMDGLQGYQGWRWIFILEGALTVAVSFLFFFLIPSFPEDSKWLAEDEKAYVAARLQKDQGRSAVERSITAKDVGNVFKDYKVIVAGFMYFGLIVPAYGYAYFAPGIIQSYGYDPIQTQLHSVPPWAAAFGYAMLLAVLSDATKHRFGFAVFSICISIAGFAILISVHNNNTVQYAALFLVTMGTYSAMPVIVCWFNMNLGGHHRRSVGSAWQVGFGNIGGIIAVFAFLRKDAPKYVTGYSICIAFTILSIISCIIYGFACWSQNRQRDKSVTDVGLTEHEKTELGDLSPTYRYLL |
| 523 | Unigene0009826 | 21 | 12640 | 7.5 | MVYTLVVHLHAKAGEENVQKLVAKLQEASQVYSKDNETLGWFVMQDHKDPQSFTIVERYAHESSQKYHLENPYWQTFDKYVIPLLDREMDLRRYNELDTSKEVKVE |
| 524 | Unigene0014924 | 21 | 89224 | 1.4 | DPVDATTAPPARKRRRRTAASGATDDCFTCRKRSVKCDRTRPYCAQCIQIGKECSGYKTTLTWGVGVASRGKLRGLSCPIAGTSGEAADTTRPSEANTTRRRKSSVSRPNAPAPVDIPRNNAASFHNNHVAAVTSMPSTTHAGFQPTLPTINAGQQGWNINGVGLNRPHPLRHHSLQHIHTARGPQYDSGPNSASSGDSFPDHGFSSPPDYPPSSDLNGYSESYPPGTSASYTSQPLSSSIDSLSSHFNSSSVGSFGDHMSSSMESINSAGSFGQSTPTETPLYSTSPFQQDEILFSPMSESMFQQNPFEQIDFNNFTEEEEDTNQTQLSIFDARWSTPFFTLSPRMQCLMEYYDRHICPYLIAFDGPNNPYRKHILQLAMQNEGLQNAIAALATNNIRMRKQPPPRRRGFVEEITDAFDGTAKPKELNEPTAEETCYKQMSIDQLNMQLTDTRAAQDDSVIATLLILCLFHVCDSGFSKFKTQLAGVQKLLSLRAPSGQSEFTGWVEMFFIWFDVMTSAVNDRETQIKSESLDMLDYSSNLGALEHFSGCDGRLFKLIARLGRLSLLAQGRPVRPQNNMPRPPPTHNAHSAPLRKRPSSSRTHSPFHMDSLESNGWGTPVISSDDEMAGSPDENIFTTPNNSYPDSRAEFWSEWNDIRARLNAWQMDTTPSRQNTPPLDSREMELGQRDLLHINESFRYSALLYTERLGSPLLPSSHPNFQKYVSNALFHITALDITSCVNKFLLWPLFIVGTECVDEGHRNIIRARCIEVQIESGFWNNISGLDVLEKVWKE |
| 525 | Unigene0008619 | 21 | 34292 | 4.1 | NATMPRLEFVTLDVFTKERYAGNPLAVVKVPEGVEVSTEVMQKIAREFNLSETIIVHDAKTGADGLPEWRVRIFLTYAEIPFAGHPTIGAAVYALGNLSAESSSDSRRGRLLANAGPMELTYNPSTGVATAGIAHNFHEHVQYPFTVEEVQALQPALKTAVKLSQQNIAVVSPVKGMNFIAVELPDLKALAAISTSSKPTPKLDDDWNVGFSGSYYYVYTSSPTKSSDGIEKITVQSRMIEGLFEDPATGSAGCGFACYVALKRGSSKITRFSITQGVEMGRKSDIEVTLTLTEDLKAIEHVELGGSAVKVMEGFVE |
| 526 | Unigene0004921 | 21 | 68361 | 1.6 | MESAHKDPALDPARQHSHGHLHHGQHGHQHGTDVPSYTVGTTNERPTIPDPSAQEHGISHGPGYGSEKKGDYNIDVERGNTSPITPTKSLEASEEQPSKKRSFYRKYRIFIHLTLWLLATAWWVASLVLHRNDKNWVVPFLIWLALTIRFVTLHTGTSYVTRPMRFVWRNTCGRATEMIPERFRLWLGAAGTFAVLLVGSFASEESQDNTRENRAISLFGMLVLIAGLYATSRNRKAINWHTVISGFLLQYIIALFVLRTGAGYDIFEFISSLARSLLGFAANGLSFLTNEEWVADTPWFLVTVLPAIIFFVALVQLLYYVGLIQWFVGKFAVFFFWSMRISGAEAVVASATPFIGQGESAMLIKPFVPHLTMCELHQVMTSGFATIAGSVLVAYIGMGLNAQALISSCVMSIPASLAASKMRYPEEEESLTAGKVVVPDDDTHKAANALHAFANGAWLGLKIAAMILTTLLCIIAFVALINGILTWVGRYINLDGNYDLTIELILGYLLYPVAFLLGVPRGPDLLKVARLIGVKVVTNEFVAFTQLTSADYDDLAPRSRLIATYALCGFGNIGSLGTQIGVLSQIAPGRAGDVSRLAISALITGVLSTLSSASIAGLVIQNE |
| 527 | Unigene0011654 | 21 | 43081 | 2.8 | MATNATAPHAGPKGTFLFTSESVGRGHPDKIADQVSDAVLDACLAEDPASKVACETATKTGMVMVFGEITTKTRLDYQKVIREAIKDIGYDDSAKGFDYKTCNVLVAIEEQSPDIAQGLHYEEVLEKLGAGDQGIMFGYATDETPELLPLTILLAHKLNSRMEEARKSGELPWLRPDTKTQVTVEYEHDNGAVVPKRVDTVVVSAQHAETISTEELRKEILEKVIKVAIPSNLLDEKTVYHIQPSGLFIIGGPQGDAGLTGRKIIVDTYGGWGAHGGGAFSGKDYSKVDRSAAYLARWVAKSIVKAGLARRALVQFSYAIGVAEPLSIFVETYGTSTKTSDELVEIVRNNFDMRPGVIVQELDLAKPIYRETAKNGHFTNQNFSWEKPKALKF |
| 528 | Unigene0000602 | 21 | 22007 | 5.8 | MGWLDKVPRTEGGGLWIGGYAALYSQQPLFQQSKITHVISVLDYELYEAEYLNAYKRMHIKLDDDPNENLLEHLPKTTAFVDEALSKGGAVFVHCAMGKSRSATVVIAYLMWKYALTPDEALEQLREGRGVCEPNPGFMEQLDVYQRMLQAENQETAKQIYETWVNERDVYRDWYSFRSYRRQAPVPKL |
| 529 | Unigene0003914 | 21 | 30446 | 2.6 | SLTDLIQVIPPDNEDEDAEDIFAFAAGLIFTDDLRNFHGDDRSLLVYKSARFGDIELRAADPVQEDDRKLFAQYLWNAGIKMAEMISHESDERWCVKDKRVLELGAGVGLGGIVACLAGASDTVISDYPADVLLENIRRNVKHALPDSFSGQCDVQGYAWGDVESDFAKQNVHSFDRIIAADCYWMPHQHENLVKSMLHMLSLDPAARVLAIAGFHTGRAKLAGFFEEAVEQGLDVEDIYEEDAKGVKREWLPERDGGREDHTERKRWL |
| 530 | Unigene0000674 | 21 | 17684 | 7.8 | SELPSSLRASWCRGQTIACPQICGGRAETNTCDAEQLTYDCSCPDGEARNISDYTQTLPFFVCEQWRTNCVLNHPDDRDGQAACESVTCGTQNATEAEGGDSSSASASVTPTSTSTDTAETGASETTGTDSSATPSASDSAAMHLAQNYGTGVLVTGLLAVFGLAM |
| 531 | Unigene0013460 | 21 | 41192 | 1.7 | PIPRDAAHPPATGLLFRYWVESDQPTHNSEEGFIARKYFQSNVVHFRPPKSSDVDLTDVFNHMDRKMKDTPFVSTCNRLVWTLRLAFSEMRKGVKNGRITLIDPSKLDKRAVFWARPFHDELTQKMAPWSNGAQRYCGTYEFLVWGAIHKESIVHTIKVKDLLRLSKTSPAVDHILRADILSEKSALVRKEDEFIRQKVPLNTFSVKAIAEIARFLGLNRASPLDHISHIVADVIQGWHLQVVPRNSQEWRVLATLFARTYLGAPAGLVQEQTVRMAFLDGVRFGQGEFUARHKPETVAAMTKRAKLVGLECPARIITDELDAIKVMAWSQEQRVGKLLKSRKEQLMLEEGFDESSEED |
| 532 | Unigene0010660 | 21 | 11612 | 8.7 | MVAQSAEFKKAAEESKKLKSKPTNDQLLKLYSLYKIGTGEDFSKATAPGMFDMTGKAKYNAWKAEVEAGTKPEEAQKKYVEFVEELKSKLGFNAUAHQETKNGQ |
| 533 | Unigene0005540 | 21 | 13476 | 8.6 | MGSNRLTFRRRVSYNTKSNKTRIIRTPGGELRYLHIKKRGTAPKCGDCGIKLAGVPALRPREYAQISHPKKTVQRAYGGSRCANCVKDRVTRAFLIEEQKIVKKVMKEAEKKQARR |
| 534 | Unigene0011080 | 21 | 61209 | 1.1 | MSGIEIAGLVLATIPLAISGLEHYADGVGTIRKLGHIREHFHYLRTDLEVEQVIFMNTLETLLSDTLGPQICNHLLTQLGGSSWYDSDVDRRLREGLQRSYDVFFKSVQRIEEALRDLMEALQLDGNGKGPFSMERRSIAEGLKRLKFGLRMKKYQEQLDVVKGCNGRLLKLAAQSSTLSRQTRRPVRAIPHYQDLRNHAVSIFESLSAGLSRSCKEQHRVNLCMKPFAAQTDDEELQLADSPTFKVVFEHCGADRPAGSAVWDLEETDIRMLNLAPHPGNGVQSLQRLAQTRSGKVAKAQQYNSPLHEISDICTGLSGLKGLRQAACLGYMADIQNNFQLSLFWPNRRMIIGASLSAISLAEVLDGPQQLWVSDLDKRNLALSLASGMLTLSGTAWLSNGQWGKRDITVFRQPSGVLLSRPFVTQQLYGPSRVSQAYFETCSLIRDEATFALGVLLIELCMGHTLDDLRAPEDLTPDGLKHKLSDFLTAARLVDEVYGIAGSHWGDVVRRCVHCEFDQRNVGLGNADFRQAVYERVVAVLEKD |
| 535 | Unigene0008089 | 21 | 33309 | 2.9 | MPITEGAPIHGNPAHLLPPSWRKSIALWLEEDTPSFDYGGFVVGDGPAEARLLAKSPGIVAGVPFFDEVFKQLDCTVEWFVKEGDGVGKDKKQHVATVRGPVRCILLGERVALNLLSRCSGVAWKSHSLLQLLRKAGYNNILAGTRKTTPGFRLVEKYGMLVGGCDQHRHDLSTMTMLKDNHIWACGGSIPTAVAAAKAAGGFAVKVEVECQSEEEANTAIEAGADVVMLDNFTPEGVRVASKNLKDRWGRGVNARALVEVSGGLTEENVADYVCDDIDIISSSSIHQGVKHVDFSLKIIPKEAST |
| 535 | Unigene0008090 | 21 | 33309 | 2.9 | MPITEGAPIHGNPAHLLPPSWRKSIALWLEEDTPSFDYGGFVVGDGPAEARLLAKSPGIVAGVPFFDEVFKQLDCTVEWFVKEGDGVGKDKKQHVATVRGPVRCILLGERVALNLLSRCSGVAWKSHSLLQLLRKAGYNNILAGTRKTTPGFRLVEKYGMLVGGCDQHRHDLSTMTMLKDNHIWACGGSIPTAVAAAKAAGGFAVKVEVECQSEEEANTAIEAGADVVMLDNFTPEGVRVASKNLKDRWGRGVNARALVEVSGGLTEENVADYVCDDIDIISSSSIHQGVKHVDFSLKIIPKEAST |
| 536 | Unigene0005975 | 21 | 97062 | 1.2 | PGKKATGADVSPADATSSTPTSTGPLDNAESLSGKDSLYSNDVQRDIILSYLFQRQCTSMWIQDVAGTNEGVMLKNARNDFLTMPLTLKGSLLQQAVSALNTKAAMTVKSRLVFTLVQRLSDDESGIPVRDNQRIQVVNDFIELSRARKHQYAAFVRSEGLLVVWDDDPSNLIHRVESIESDLLRVVWGLSRNIEEEKTALISVTASDEELQAGEESRPTRLYWSIMSGCTLCLLTVLFGRRLQNTFIQVATLGNYSSLAFLALTPIIVLFSLFFAFVIVGFFFRLVGPVKQLHENSETFSAVKSPRLTSKNLPHVTIQCPTYKEGLEAVLKPTVSSLMKAISTYELQGGSANIFVNDDGLQLLDEEQRHIRTQFYEDNNIGFVARPAHDPNGTGFVRRGKFKKASNMNYAMDFSNRIEDKLQNVIRDEKWTTLDEAAAYEQALQEVLDEQPEDQPKGWASGNIRMGDYILIIDSDTRIPEDCLLDAVSEMELSPDVAIIQFSSGVMQVSHDFFENAMKFFTDLIYTSIKFGVAAGDIAPFVGHNALLRWSALQHVAFEDKESTLPELYWSESTVSEDFDMALRLQIEGYHLRYASYFGDGFQEGVSLTVYDELTRWEKYAYGCDELMFHPLKYWLFRGPLTPLFRKFLASEMPLSSKINVIAYLGTYYALGAAWVFTLANYFVLGLYNGHYDTWYVDSWQIFISILFVFAIAANFGLAVSRYRSGEREFLPALIESFQWCLMFLVFFGGISFHISQALLCHLVGIDMQWGATAKEVTRTNFFIEAPIVLRKFKYSMIFCWFMIVAMIIMAIGPFIPWSWNITEFTAIFPLALMLVCHILMPIVLNPGLLAFTW |
| 537 | Unigene0009684 | 21 | 107705 | 1 | MSTWSAQLRRSVNFLLVDPRAAALYWGPSKTLIYNEPYHAVLAERHPWALGKTADQVWPEGYRELSDAFAKAEATGQSSRGDREAFFLNRRGFAQEMYGSWSMLPVIGGSGILGFYNNVLDLTDRVHEERQMATLLKLERQTSDAKGLHEFWPAIIDGLEVNREEAPFVALYAAVPRGQAHSSRAPSVSSSSGLMEPSSLLPTRHWSLEGTLGVTAISEVFPSQINTEWAVQHITPSFKDALLTGQPQVITILPDSHMYPAARSRAYGDQCSSAMLIPLGRSQEGDGVGFLILGISSRRHYDLNYKQFIRLLARQLVSTLAAVRQSEEDAKKANMQAELAALDRILLSEKLALTEQQARDNERRFRSIAAHMPMAMYELSPAGEIVYANDSYFELLGVQQHSLTPYFWEQLIHNDDKLMHEEKIRQLMAGESVRFEVRLNRKYVAGDALSGETIEGETWFLSAAFCVKSDDGIVQCIQGTLIDISRQKLLEAFLAKRLDEAIELKRQQEHFMDFVGHEIRNPLSATSLCASSIQGTLEDLLKNTTLEDTTETITIDKETIQTQIENTEIITTCIQHQKRIIDDILTLSKLDSDLLVITPCETKPTDLIAQSLRLFEAEVQVADTRLEYVIDQTYRDLGIEWLMLDPSRLLQILINLITNAIRFTKNEVNRTITVTLAASDTRPQSSVAGIHYLNQPNAPSSTDEDVQTDDSVYLLVAVSDTGCGMKPDELERIFLRFQQSSHKTHIEYGGSGLGLFISRELARLQGGQIGVHSQHGQGSTFEFYIKTRKCNAPKSNLGEANQDHESQQVAQPIRRKSYVQQSHAEHPTHILLVEDNLVNQKVMDRQLRRAGYTVHVSNHGGEALEHIRRSKYCRNDGVELDVVLMDIEMPVMGGLDCTKRIRSMELRGEIARHMPIVAITANARGEQQTQALDAGMDAVVTKPFQMGDLLSVVEKI |
| 537 | Unigene0009685 | 21 | 107705 | 1 | MSTWSAQLRRSVNFLLVDPRAAALYWGPSKTLIYNEPYHAVLAERHPWALGKTADQVWPEGYRELSDAFAKAEATGQSSRGDREAFFLNRRGFAQEMYGSWSMLPVIGGSGILGFYNNVLDLTDRVHEERQMATLLKLERQTSDAKGLHEFWPAIIDGLEVNREEAPFVALYAAVPRGQAHSSRAPSVSSSSGLMEPSSLLPTRHWSLEGTLGVTAISEVFPSQINTEWAVQHITPSFKDALLTGQPQVITILPDSHMYPAARSRAYGDQCSSAMLIPLGRSQEGDGVGFLILGISSRRHYDLNYKQFIRLLARQLVSTLAAVRQSEEDAKKANMQAELAALDRILLSEKLALTEQQARDNERRFRSIAAHMPMAMYELSPAGEIVYANDSYFELLGVQQHSLTPYFWEQLIHNDDKLMHEEKIRQLMAGESVRFEVRLNRKYVAGDALSGETIEGETWFLSAAFCVKSDDGIVQCIQGTLIDISRQKLLEAFLAKRLDEAIELKRQQEHFMDFVGHEIRNPLSATSLCASSIQGTLEDLLKNTTLEDTTETITIDKETIQTQIENTEIITTCIQHQKRIIDDILTLSKLDSDLLVITPCETKPTDLIAQSLRLFEAEVQVADTRLEYVIDQTYRDLGIEWLMLDPSRLLQILINLITNAIRFTKNEVNRTITVTLAASDTRPQSSVAGIHYLNQPNAPSSTDEDVQTDDSVYLLVAVSDTGCGMKPDELERIFLRFQQSSHKTHIEYGGSGLGLFISRELARLQGGQIGVHSQHGQGSTFEFYIKTRKCNAPKSNLGEANQDHESQQVAQPIRRKSYVQQSHAEHPTHILLVEDNLVNQKVMDRQLRRAGYTVHVSNHGGEALEHIRRSKYCRNDGVELDVVLMDIEMPVMGGLDCTKRIRSMELRGEIARHMPIVAITANARGEQQTQALDAGMDAVVTKPFQMGDLLSVVEKI |
| 538 | Unigene0000188 | 20 | 43144 | 1.8 | MDGSERTPTPDASARERLPNLFEVLSRRTLAPVDLFSFYIYMRDVQRSVDYLDFWLDVSQHMSLCRHYVRELRRSVLVSTPELDKSGSKRSSQILDNYNGLERTSTEPGPSGAFNDKSKERQTPDQRLSAFLRAENGTGHSPQNSTGSHNNSDSGLEQPRPSYMTGQDGSPSSGNINSNNSPQHTVARADIRASAEKILYTFLLPGAEREIILPQGILNDITHAIENEGRDDPEVFDAAKDYVFQAMERDAFPGFLRAKALGNIVQPSMMLRLICGLVAIFGALWTAFVLIFLNRDRATRCWLILPFTVGIYLLATHQYMLDPILALLGFSEYTFFSFARIKEPFVKKLLSQRALMCLFWITLIDVALLCLFIFVPGKRL |
| 539 | Unigene0011950 | 20 | 12064 | 6.5 | ANTRTTKRTHHSDTKGQHYRANTANMVLAVDLLNPTPQAEARKHKLKTLVPGPRSFFMDVKCPGCFTITTVFSHAQTVVVCAGCSQVLCQPTGGKARLTEGCSFRRK |
| 540 | Unigene0003675 | 20 | 50029 | 4 | LQPLGGTLEADSDDDDSGDEISFSTVGLPRALFERRSEQSHTTRSAQYSHSAAPRPRITPTQYATPQSSQLHYRQISDPPEERVRKARKGNQTKHATHASSSKIGLIARDSPRRIPGQPWLEIIPLDYRASFDVAHSTAPVQDPEDFADDLSESQDLDEIMTDPVMGQPLFGEEKAQESAGSRIVHDFMPDATRLFEELNNEIKWQTMFHQTGQVPRLVCCQATVDEDGSMPVYRHPSDQTLPVERWTPTVERIRQEAEKVAGHPLNHALIQLYRDGNDFISEHSDKTLDIVANSSIINASFGAQRTMRIRTKRDKGNTTPVARTTYRVPMPHNSMIMMSLPTNAEYLHAINWDRRPACELTDAEKAYGGQRISLTFRHIGTYLNSDSTVIWGDGAVGKTKSAARPVVNGDPTESEKLIQAFGSENAASSINWSEIYDSGSDVLHLK |
| 541 | Unigene0010064 | 20 | 42964 | 2.8 | MGLKGVHFGGGNIGRGFVAEFLHNSGFEVVFVDVMDSTIESLQKNKSYEVTEIGPDGETKFTIDNYRALNSKYEMAAVVEEIATADTVTCAVGPNILKFIAEPIAKGIEARKSDTPLAVIACENAIGATDTLRGFIEQKLDEKTKSDIASKARFANSAIDRIVPIQDEGAGLNVKIEKFYEWCVEEKPFDGKPPAIKGVHYVDDLEPYIERKLFTVNTGHATAAYYGHQAGKKYIHEVLEDKKLHDIVQGALEETAHLICTKHSHITKEEQQQYVQQIVKRISNPVLKDNVERVGRAPLRKLSRKERFIGPAAQLAEKGEKVDSLLGSIEQALRFQNVEGDDESVELAKILKENDSKAATEKLTGLDEKHPLFAKVEERVKKVQSG |
| 542 | Unigene0000837 | 20 | 28141 | 4.9 | MASIGRLAGKVAIITGGASGIGRATALAYTKEGAKIVCADIRETTRYTGSDEENGTTVEQVTALGGQAIFIQVDVTKAESVESLVQSAVKEFGRLDIMVNNAGVSLEANNFRPVWEIETDIWQGTQDVNSTGVFFGTKYAVQQMLKQDPHPGGDRGWIINTSSILGYVAAEHTAAYCASKGAVLNFTRAAAIDCAPHRIHVNGLAPGYTQSSMTGPLWADPVAKAKLVEKHPFRGLGQPEDLAKAAVFLASEDAQWVTGHTLPV |
| 543 | Unigene0004412 | 20 | 52810 | 2.1 | MPVTQFNRAEKYRYQNGFGCYHETEAVDGALPIGANNPQKPAYGLHNEKLSGTAFTAPRHENKQSWLYRILPASAHSNFEPRESPSYDLNPGGLRDAKWHQIPNQLRWNPFDLDETVDWIHSLKLVAGAGDPTMKTGLGILIYAAGKDMDERHAFYSADGDLLIVPQHGALDIQTELGKLLVRPNEICVIPRGIRHRVTLPDGPVRGFVLELYQGHFELPDLGPIGSNGLANARDFQAPVAAFDEDTENEHTLFAKFSGTLFAAKQNHTPFDVVAWHGNYYPYKYDLGRFNTIGSISYDHPDPSIFTVLTAQSDHPGTAVADFVIFPPRWLVAEDTFRPPWYHRNTMSEFMGLIHGQYDAKTGGGFQPAGASLHNVMSAHGPDASTHEKASNAELKPAKVGDNSMAFMFESALMVGVTEWGLKQCNKVQEDYNAESWEPLKPHWPGKANLKKGGTNGAHAASTEEADKQQLPHYS |
| 544 | Unigene0012995 | 20 | 23637 | 3.5 | DQTPAHHDAPSLPGSPLPKRTKYTEPAANLSVASILNDQQATPTPQTAQLPNPLSEMSGTINATAGAPPQLSNPSAATLPPMSFEPPPPALQVKLLSETAKPPTRGSAFAAGYDLYASKEAVIPAKGKVLVDTDISIAVPVNTYGRVAPRSGLASKHSIDVGAGVIDADYRGPVKVLLFNFGEEDFKVAVHERVAQLIVERIYTPDVVVVQELEATVRGAGGFGSTG |
| 545 | Unigene0014344 | 19 | 119127 | 0.9 | MLLSQLSACSKRLATGSSARAFSSIAPKSAPRTALVAKRRELGVSSYNAFTSQYRNYAQAAERTDQGVDPSDSFLTGNTAGYVDEMYSEWKRDPSSVHVSWNHYFRNMESGDMPVSRAFQPPPTIIPQPAGGVIAPSGAVGVGEGQSSEVLNHLKVQLLVRAYQARGHHKAAIDPLGIKDLNRSTPKELDLATYNFSESDMEQEFSLGPGILPRFAKDGREKMTLREIIDACERLYCGPYGVEYIHIADREQCDWLRERIEVPSPYKYSVDEKRRILDRLIWSSSFETFLATKYPNDKRFGLEGGESLIPGMKALIDRSVDYGVKDIVIGMPHRGRLNVLSNVVRKPNESIFSEFGGSAEPSDEGSGDVKYHLGMNFERPTPSGKRVQLSLVANPSHLEAEDPVVLGKTRAILHFNKDEEQANSAMGVLLHGDAAFAAQGIVYETMGFVGLPAYQTGGTIHIVVNNQIGFTTDPRFARSTPYCSDIAKFVEAPIFHVNGDDVEALNFVCQLAADWRAKFKKDVVIDIVCYRKQGHNETDQPSFTQPLMYKKINEQLPVLDKYTQQLLEAKTFTKEDIEEHKAWVWGMLEESFAKSKDYQPNSREWLTSAWHGFKSPKELATEVLPHEPTAVDPETLKHVAKIIGQPPEGFHVHKNLKRILANRTKTVEEGKNIDMSTAEALAFGTLVREGHHVRVSGQDVERGTFSQRHAVLHDQENEDTYTPLKHMSEDQASFVIANSSLSEYGTLGFEYGYSLSSPTALVIWEAQFGDFANNAQCIIDQFIASGEVKWLQRSGLVVNLPHGYDGQGPEHSSGRMERFLQLCNEDPRIFPSPEKLDRQHQDCNMQIVYCTTPANNFHILRRQMNRQFRKPLISFFSKSLLRHPLARSKIEDFTGDSAFQWIIPDPAHDADAQFKIDSHDKIERVILCSGQVFAALFKHREQNNITNTAITRIEQLNPFPWAQLKENLDSYPNAKTIVWCQEEPLNAGAWSFTQPRIETLLNHTEHHDRKHVMYAGRNPSASVATGLKSSHTLEEQNLLKMAYEVKQDKLKGE |
| 546 | Unigene0009585 | 19 | 171432 | 0.6 | MSFLFKSSKKGGSGPVASSNAPSLHAVREHTPRDAGAPSQIPTLNGHQSNGAKPGSPTPPQSVNNSLNSISGFEKPSPQARPADERIQAPRDDGPRPEQKSLRERRETSQDAVGQRTPLQQTRPSQDASPYPWSQRRLNFTVAANPFPRYGAAVNSSSSKDGSIYLMGGLINGSTVKGDLWMVECGAAGMGSMTCFPVATTSEGPGPRVGHASLLVGNAFIVFGGDTKMDEGDQLDDTLYLLNTSTKQWSRALPAGPRPPGRYGHTLNILGSKIYIFGGQVEGYFFNDLVAFDLNALQQATNKWEILIQNTIDGGPPHGQIPPARTNHTMITWQDRLYLFGGTDGIHWYNDVWSYSPHTNTWTQLDCIGYIPAAREGHAAALVGDVMYIFGGRNEEGNDLGDLAAFRITSRRWYTFQNMGPSPSPRSGHSMTTVGKSIVVLAGEPSSAPRDPVELGLAYFLDTAKIRYPPDATSGTPPSGRRPSGERSGSLSNIRGPGPGPGQRAELMDRERSGSAGENRIRSPDSGSRLPRPGQPPPSGPPPQAPGQVRQIGPPATTRQPTRSAERALSPTNESGRPQQRFDSVAAIAGSAVSRNSPVPKDRSMPTSPTFEGDQTTAPPQAEEQIRSKPETYTPSQELPGSFPQTPGLESRNESRTGSRSTSRQAQNRFEEPSRPSVDNEPLSRVRKISEEHERPQDSGLGSSPALTSQYEELVRELELIKQKNAWYASELALARKSGYQARSTESPVFDESSKQTFNDDDKPLIEALLKMRGELAKMQETIEEQSKNAATRVAEIERQRDTAINEAVFAKARLAGSGGSQPDGMDRDNDISRRLAASLEAQSELKRQIDTLIQGAEAERKARELAEDTANAAQKRVLELDQYRQQHASEVESLRSELHESQKAHRETAASHTEILTQHKILEVDKNELRQKLESMTNQGSTQTEILASLEKAVQASKEKADLLQQQLDDARSSRDELESSHRELKSQHDQRVGELESTTRRLREAEELAEKHAEEARTHREAVMSGLQQVTSRDVGANGVQDERVEILQKQVETAQAQVRQNQAAADAASEKLRRAEERIAGLESYQEQASREGLSIRKQLQVAMKESQALGSEKAELEQKLERHMLETNALSVQHASLKDILNERGISAADKRRSRGLDSPGSHRFSTPDTSRVREMEQQLEAQAKNHEDLKQQFEEVSGRSDKMKREYEEKLAALDNDHQAAVKYLRGTEKMLSKMKQEIQRVKNENADMSKKLTKAKEDMESSARSTPATERKNEDWEGERQKLKQEVSAVQTKLQGSIDDLESRITKMQEQLRTTEQELSQSKTSQSTAQTELENLRSTIESSKADMERLQKENALLEERARDAENKVQLLLDQVESSVDNYRRQSRMRSPGGTPTLNGVVHQRAPSVSESSVDAGELPEHLKSHSRQISAGGDSTYSASDSATGNEGRNSMALDSLASELDALRTHWETTNKNYRLSDRFDYDKTPTTAGPTTAGGNSSLMDWRRGLELDDDDDNATRPGTSDGSKVSKSPDAQRKPSVDEPTPTATPR |
| 547 | Unigene0004045 | 19 | 21512 | 5.8 | SISTYDLTVPILVRNLELLNIIIDKAIKHAEDIGKDPESFLEAHIIDDMLPLSFQLQTCNRTAHGMLPLITTSAIEADKENKDNIEEPKTLAEHKTRINYTISLLRSLKKGDFVEASTETRLPVPPSYQAAFPAASKGYVKFTALSYLQKYVLPTFFFHMVTAYDILRKQGVPVGKFDFLGAEDFEAWEM |
| 547 | Unigene0004046 | 19 | 12444 | 10 | ASISTYDLTVPILVRNLELLNIIIDKAIKHAEDIGKDPESFLEAHIIDDMLPLSFQLQTCNRTAHGMLPLITTSAIEADKENKDNIEEPKTLAEHKTRINYTISLLRSLK |
| 548 | Unigene0001785 | 19 | 27861 | 3.6 | MPPISKKVHINVDLGEGYGNFKAGPDDELIPLIDHANVACGFHAGDPLIMSETVAKCKEHNILIGAHPGLPDIQGFGRREIKMSPEELTAMTRYQVGALQAFLAAADVPLNHVKPHGVLYGMMYRDKEICRAVYAGVPPGTRVFGLAGTFHEEVAKEMGLPFTAELYGDVKYSSDGKLVIDRKKKSWTSEDVHKHVSSQVENSSVTAVTGEEVQLPIGDHDVSLCCHSDSPGALEIVKAARQIVDDFNKKMKY |
| 549 | Unigene0004750 | 19 | 94669 | 0.8 | MASFLTSLNDRLALPEHQLSLVSICGILFVLAWVLLFFTELSSIKQAYRSANYLFKFAYSCFFKPHSGDGTGKQQDALESFYKSQAEIYDATRSRLLQGREDMLALVAAQVKYRRDTGQASAKPIWVDIGGGTGWNIEQMAEHVDVPTFFHAVYLVDLSTSLCEIARKRFQRLGWKNVHVICEDARVFRLSDYEAGVGDEKRDFSIGRSAYDDEARDSVGADVLTMSYSLSMIPEFHPAVDSVANLLAPNGIVGVVDFYVQNKIEFAGRNYTGGSIDRHCMWISRVFWRTWFELDRVNLDSARRDYIEYKFGTILSTNRRCHLLGFRIPYYIFVGCARTTINPLQQMAAVDAAVTESPFIAALDIHAQSLTRKNRRSSSAERRSKAYDTAVVNLAASLPLPAAYYQTNKTRVYYDDTLEKHRQFNDEYIYSFTWEDSRVDSRLLKITSDDVILALTSAGDNILSYALERPKRIHAVDLNPAQNHLLELKVAAFRGLEYQDFWRLFGDGKIDNFHKLLMERLSPHLSSQAFDFWLHAGPSTFDPRGKGLYFTGGSRHALKLVSWLGSLLGVRKDMNKLASASTLAEQREIWNRRVRKVLLSQLLAYFVVGSERFLWKALGVPGEQRAMIEEDFKLQLDRGDAVKADKSGEKSGTGEPGAVGATKSGKAIWNYGVQTLDPVANGTLLSEDNHYYLLCLLGHYTRRCHPTYLTPKSHLKLSAPGALENIRIHTDELAEVFARMQPEALTIAVLMDSMDWFTPGGPEAPAQCKAVNRALKLGGRVLIRSAGLNPWYMRVFEEYGFSPKRVAARIPPGTCTDRVNMYASTWICTKVVSL |
| 550 | Unigene0001890 | 19 | 44795 | 2.5 | MADKGLEDIDAGQIESNYDETTDSFDAMNLKSELLRGVYAYGFERPSAIQQRAIMPVIKGNDVIAQAQSGTGKTATFSISVLQKIDPNIKACQALILAPTRELAQQIQKVVVAIGDFMNIECHACIGGTSVRDDMKALQDGPQVVVGTPGRVHDMIQRRVLRTDSMKMFVLDEADEMLSRGFTEQIYDIFQLLPQSTQVVLLSATMPQDVLEVTTKFMRDPVRILVKKDELTLEGIKQFYIAVEKEDWKLDTLSDLYETVTITQAVIFCNTRRKVDWLTDKLTARDFTVSAMHGDMDQTQRDVIMKEFRSGSSRVLIATDLLARGIDVQQVSLVINYDLPANRENYIHRIGRGGRFGRKGVAINFVTADDVRMMREIEQFYSTQIEEMPMNVADLI |
| 551 | Unigene0010952 | 19 | 70495 | 1.4 | NTVTEKVSSLTTGAGSNDNGTDNANTGGTTPNLVLDEVTGEKVSKSELKKRIKAREKEAKKAAAPPKQAPASSKKKVGGAQDDEAQLNPNQYFEIRSRAVKRMKETSSPNPYPHKFHVTYDLRDFEKDFGHLKKGDVLKEKTISVGVRIYNIRTSGENLRFYEVAVNGAEIQIMAQNLESTSATPFAEQHDILRRGDIIGVTGYPGRTSPKREDNPGELSIFAQEVTLLAPCLHQIPSEHYGFRDQESRYRNRHLDMIMNKSTVDTFIARSKIVRYVRNYFDNNGFYELETPILLKSAGGATAKPFFTHHNDLNMTLALRIATELPLKQLVIGGIHRVYELGRQFRNEGIDLTHNPEFTTCEYYEAFADVYDVMERTEELVEGMVKFVTGGLQTKFTTVDGEVYDVNWAKPWKRIEMIPALEEACGEKFPPGDQLHTAETNEFLRRMLKKTGLECTPPLTNSRMIDKMVGEFIEEKCINPTFITGHPQVMSPLAKYHRTNAGLCERFEAFVCKKEIVNAYTELNDPFDQRLRFEEQANQKAQGDDEAQPLDEGFLNALEYGLPPTGGWGMGIDRMVMFLTNNYSIKEVLTFPFMKDEIQAPKPKAAEVVDVEPVPVEGTTHK |
| 552 | Unigene0013174 | 19 | 32262 | 3.4 | MSAVNLKEVHDFLVEIAYKAGEMITSATPAVSGHGTKKNSADLVTETDQAVEKMVSEQLGGKYPDFGFMGEETYTSGDQLEDRPTFIVDPIDGTTNFFHGHPYVAVSLGLAMNKKPVVGVIYNPFTQTLYTGIRGQGSYITSPQHKTPAKLPLRSPEPFQDLSHCLVAVEWGSDRDGSDYETKVNTFRKLCASKEAGGAMVHGIRSLGSAELNLCGVASGHLDVYWETGCWAWDVCAGWVILEEAGGKMVGAMKGDWDPSVDQRRYMAVRGGEGWEKIVEEFWGCVEGKIEVG |
| 553 | Unigene0010268 | 19 | 73924 | 2.6 | FIAALVPAGTALYVNGSVTAPCESPLYCQGEILREMQLAGAFEDSKTYVDLPTIRPLEEVIAAFNNLTKPLTNNTEFNDFLQEYFGEAGSELGEVNPDDLTTNATFLENVTNPDIRDFLQQVIDIWPELTREYVGSANCTGCVSSFIPLNRTFVVAGGRFREPYYWDSYWILVGLLRTQGSFTEIALNIIENFVDLVDRFGFVPNGARQYYLNRSQPPVLTLMVQAYVEYTNDTSILERVLPTLEKEHEFWGVNRTVEVDINGTTYELNHYAVENNQPRPESYIEDYHTATNASYYAESGIIYPATELNESQIAQLYSDLASGAESGWDYTSRWIGNPQDAIDDVYFPLRTLNTANIVPVDLNSILYANEAALSELYNLTGNSSAAEAWAEIAANRSDAMTAVLWDQEQWGYFDYNLTSSSRQIYIPADENAAQSETQGAPLGQQIFTHIAQFYPYWTGAAPAWIKQNPSAILRAYERIEAELERFSGVPGATNLETGEQWDEPNVWPPLTYILIDGLLNTPATFGEEDPSYNRTQELAFEIAQRYLDSAFCTWRTTGGATANLPKLESVNPEDDYDGAIFEKYNSTDITAFGGGGEYEVQLGFGWSNGVLIWVGDLFGDRLELPACEQELEEGGDANDGGNSRKIKKRDAKWRSKGH |
| 554 | Unigene0010459 | 19 | 30693 | 4 | MSFNDSSNLESQPTTWRRNDDPQYADDPEFQRYTDGLSNKLFSLTSNISRLSSQIGLLGTRRETDRVRERLQDLLSETQDAFKEVGEGLKKVQSWHDLNPTQKYTAGKLATEFRASLEEFQTLQRSALEKQRASQSAAKAALDSEGVTSPGGTQLTGQQQQLQQQEQLRLADQNEVDFQESLIIERESEIRNIESSVSELNELFRDVATMIHDQGGQLDIISENVTQTRDDTRNADQQLRTAARHQKSARGKACCLLIILAIVLAVVLLAVL |
| 555 | Unigene0013767 | 18 | 65553 | 2.3 | MFALRSGTLSAARPSWCARSQWHTRSLSLTARRRQATPIDNDASNLNNVSKNITQPKSQGASQAMLYATGLKEDDMNKAQVGISSVWFSGNPCNMHLLDLNHKIKESVQKAGLLGMQFNTIGVSDGISMGTKGMRYSLQSRDIIADSIETVMNGQWYDANISIPGCDKNMPGVVMAMGRVNRPSIMVYGGTTAAGCGLQPKNGKLDIVSAFQAYGQFLTGEISEVERADTIRHAIPHCGACGGMYTANTMASVIELIGMTLPGSSTNPAMSKAKQLEVQAVGPAIRNLLKEDIRPSDIITRDALEDAMVLLNITGGSTNAVLHLIAIAHSVGIELSIDDFQKVTDRTPFLADLKPSGKYVFQDLHEIGGTPSLIKFLMSEGLLHGGRITATGKTIKQNYENAPSFPESQDIIRPLNNPIKPTGHLCILRGSLAPEGAVGKITGKEGTVFTGKAKCYDAEDLFIEALERGEIKKGEKTVVIIRYEGPKGGPGMPEMLKPSSAIMGAGLGQDVALITDGRFSGGSHGFLIGHVTPEAQAGGPIGLVRDGDTITIDAENRVIDLVDVKDEELQKRKLEFKAPALKYQKGTLYKYAHVVKDASHGCITDGM |
| 556 | Unigene0002396 | 18 | 75237 | 1 | MQQRKEEILAKKAKLAELKRQRELRQRDLSTNRQSLDASDAFVAPSPQRSSATKREDIESLISSLVDRPSSATPVTPTSKLRSNTTSHHSEDGHTPPPRSSDGSNEPQTLSFAALTTVFEIPVESKPEYITYSKGVQTADAWQQSGGESPAEGSDPDTRSTSPSRRRRNLSPRARELQEEEIRQRLRQEIEEELRSLQAHEHVAPDKERFPARSLAPEELSAVTSSNDFLDFVERSSKVIERALDEEYDVLADYAAHAREADDDEDEYVGRRGRRVKEVASFDLQPEMGRKRMISDIDFSPKFPELLCTAYTKSQSTPHTPPGLLNVWNLHLKGRPEYSLHATSDLLSCRFHPFHPSLILGGTYSGQLCLWDTRARNQRTGEPVQKTPLTGGRTGHAHPIYSLATVGTQNASSVVSVSTDGVVCAWSTDILTQPQEYLELHAPNPSAYKTDDIAPTCISFPASDPTYFLAGTEQGSIFPVHRYDRAGAKSGVDARTSYRGHEAPVTGIDFHPARGKIDLGDLFVSCGLDWSVKIWRARPPSTTSSTISGNTEIVKPLLEIQREDSVYDAKWSPTKPGIFGCVDGRGRFDIYDINASSEVPVAGAKPSHQEKDIYALKSLNKLAWEKNQGRHAAVGGLDGVTTVFEVGSELGGVDTKSDAWLGVKKFVARQTKSKMEM |
| 557 | Unigene0014856 | 18 | 121670 | 0.8 | EATLSTAPASPNDTDHNNMTDHKHISEHTHTTTATAMMPPEHSTMAQMSFGPATQQTVTTVTTTTTVSLPPLVMKPPKDLYDRDPKQYPLAFTPTPSSIKRFCFDVNGRPTVFHESEDPAETMRRYKQLQAKIHRSNGSIRQEERHDGYETPVRALEEEQQGKEPQNGRSNSLKRSATPVSIAEAAELASLQRKTKKQRSSLHRTYSELTEQPSLNERSAARASQQLGNPSHPSPVTPDTDVMGALPRAAAGRQGPSKSRAGGKPSHSRVQELQDDSQDTQMADLEDQEEDDSELSVGSSYTSKKLPSLEKTAEAWAAQRASAAATPPITDTDLMPAPRAAESRRPNLPGILTSSQQDASLPSPSLSPITAAANLASRQNAFDRSFAEHNEENASVVSGLDLDDDSKTQSTKSFESQPSKGSSKEPPFKFTHGTQSQPSASGPTIMDIPTMVDAFDSMPEAMQTYLIYQFLRRCAKPTLQMVANVVNPALKCDPFNILPAELGLNITRFLDAQSMCRAAQVSKRWRKLINSDEKAWKDLLERDGYTLPDGEIARAVREGWGWQHPGEEGHEQDLSKANSPEPGDDSTLASEDELTASSSLTTRDRKRKVVKSSSAKKHKRRPVHASSSLTKPTATPNSKWLKLLGTAKGANAFAHAAMQAVPHPSVGLESLRNMHLFKSLYQRHYLIRKAWMDEESQPQHLAFRAHHRHVVTCLLFDSDKILTGSDDTKINVYDTKTGALRNRLEGHEGGVWALQYDGDILVSGSTDRSVRVWDIKSGRCLQIFQGHTSTVRCLVILQPVQIDTEPDGTPIMMPKEPLIITGSRDSTLRVWKLPKPGDRQIWHAGPPTNDHDNPYFLRTLSGHHNSVRAIAAHGDTLVSGSYDCTVRVWKISNGDLVHRLQGHTQKVYSVVLDHARNRCISGSMDNLVKVWDLVTGSCLFNLEGHTSLVGLLDLSDDRLVSAAADSTLRIWDPENGACKATLSAHTGAITCFQHDGQKVISGSDRTLKMWNVRNGECVRDLLTDLSGVWQVRFDERRCVAAVQRNQLTYIEVLDFGAARDGVSAEQRGRRVVVDARGREMADIDLDTTGSVHEDAAAA |
| 558 | Unigene0002173 | 18 | 52835 | 2.1 | LQRLPSNIQPFFAATKRFTPCNKRFNATVASSQPQRDDQDGPFDRRGGSLRAWQSTAKPDHFLSSDALRHPLPKFEDSASSTSRSLRYEGGELKIHDEIENRSIRIPGQWLRDNCACSQCRNTDTAQRQINVLKGDIDTTISNVHLQDSRNGQVTVTFSDGHQSTYSAQALLDRRSKNVTRSRTGVLPIKLWTADIASTRPAVQYTDLQNSTGMNELLQKIRTFGFCVVEEMPATPEATESLLNSIGPIRNTHYGGFYDFTSDLTMKDTAYTSEFLEPHTDNTYFTEPAGIQALHMLSHTQGSGGESSLTDGFNAATQLFVEDREAYLTLSEVGVYAHASGNEGITIQPSQAFPVLNHDPEEGFLNQVRWNNADRAGIAAEFDIVEKWYAAAEKFDRLLSDKSNQYWFQLKPGQTLLFDNWRVLHGRNEFTGKRRMCGGYINRDDFISKYRVTNMSKEQLEVSTVSG |
| 559 | Unigene0010277 | 18 | 17179 | 7.7 | MDHEKLARMQNAVRIGGKGTPRRKVKKVNRTGGGDDKKLQAALKKMNVQPIPAIEEVNMFKQDGNVIHFSAPKVHASVPSNTFAIYGNGEDKELTELVPGILNQLGPDSLASLRKLAESYQSMQKSAEGEDKKDDDDDEIPDLVEGENFESKNDVE |
| 560 | Unigene0003222 | 18 | 49495 | 2.4 | LPTRSATTRTSSTLDDDAIPDEDSSETSQLFHERLQAWKHAVKYVEDYIQATEKMHHDNAKDYNKVLKTVSSPLKEGHHFDQNLGGVAGLFDNVRSNTQALVNSHNETASTLKGTVLPIFERLHSEIKNKTKELTKGAGKGSKAVDKARGVSQKHIELLGQHTAAYDSSGGSVKAADDPYILQRQVYHRLNRQVLEENNNRDDMLSVQNSFAQFEAHIIATLQHGWQQFNQVVSTQADNTRSLYGDMTGTIQRIPADFEWNGFVKRNVGVLIDPNGGKRAVEQIKFPNQDHSATRPLIAGSLERKKMLKRYDAGFYVVTPSKYLHEFKTDDDFAKDPSPENSLYLPDCMIGAVDGVKFNVRGKDTSGNAMMNKMARAHEFAFKAHTPDDARKWHSVIASVAGQTSNEMPGNDESPISSPVTKSEEAAAPPVATAAEPVPTTTVAPGSAV |
| 561 | Unigene0013472 | 18 | 438515 | 0.2 | MGKIKKVATERHTSTLSPFVKDFVKQSIEIPLYQLPAKLDEFPQHWPLPRGDLYHWIALLDRFDHVLELFNKEYGLSEGPQTQQFERRVLQKGDAAEDTPYPAVGGQTQELDAAGYSAEGDRELVESILRFTRLLLEHCGNRSLYASSTHINDLLHTTSLSLLQQCLKLGLRLAQRYQVARYKNHNPAHQAVLLANHYNINLDNLHKIAMPFPKPHGQNNSSLQSSGKAKEKPATLPTFIPTDLVAIAKGPMPAAVRSTVSTVSLSYYEQASAEPSQTPSSHLSPTTPSTPTPARRASNLGPSRDRPSTGDRSASTEDSPVVPGKQREQDNSSPTSPKLFTILSTKVAQTPAWVLVKESLDKVPVDMQYEVLNRIRIAKAFASPEVNAQEILGVRLLAIANLVYALSESKLQERIGIPDAEEPRRYHLAQQLSDLLQPATDGQTPLTLQTETTVLMTLEALSKSRNKVSEVGDALAISVNHGVLYYELRKLISTLSVAPNANKAVELEESQWRDAVFDLINSLLQCNAQARYGEKMVAAGVMGILVEALQLRTSRAERFYERTLQFFDSFVHGIGTAFQTLADVRGLDIIADLTASEVNTALEKSRNGNGLPKEYKSRVVDYDIPFYQQSTLRQLFKNTVHMFEHNTGGHDRLLRNLIDTPQVLGALRSVIENSSIFGANVWSGAVNIISSFIHNEPTSYQVIGEAGLSRSLLESITQQKIPDELPPPGEIVSASETSPIVANDEGQPIYPSITGILPVGEVLCDVPTAFGAICLNENGMQLFQASGVLVKYFDIFLSPMHVRAMEDEGQTAATIGHAFDELARHQPRLKSQIMAAVFNMLKRLKELTAILSRGKQAGAKLWKAADGKLIVAGGSAALTAGAVDAATQTGSSEADGGSSSPLEVAAHDASIDEREYAPAICYLSACFKFLEGFFHNSGMCNLFCEESGAELLLDLAMSPSNPHDLVAFPLFSKLTQVLKTMCEAKAHLVLPSMIKRTQRAVADLKDFMNVDSRSSSFSSFLDLSQSSKSSLPDGTNGTAVVKALLNTHMLTHILGRTLAPPPYTLRHGSQNNHVFSSLNFTDVYIELVDDLSRLHASCLWEGLLMLRDMPDDKLTKSDPRAFHMRRVDNEGYVDFATDRSDPARLTLNGSSSDGSDEKKAPAAVLGLKNTRTIRYLLSQVPMGIESFFHSLGQAIVTKRATDAIVRQHAIFVAEHLAKAVLWELAYRRNKAHEEGMELKYVCHILKMIGRMLVRNSYSMETFGTKEALTLVMTKFYAAGGFDKLNEYLDRFVRLLVQRPDTDKADTSVKDAITAILGFYSQIARSKCINDASQSNNIVVKHSTQPDYFQPGQFVVEVRHAILPAISRIWRSEHIEKFSKGNVESVVTILRLILKAEGEDRALRRSDQASRRVLARRPGFRLRNEDHLSDLTAAGFDATLSREALYRCAGHHHQAEAYCRLRQKDPRVPRFSIPEGDSTATPSDSTGQGAQSDVQVHVGPAPGSDEIMQDEDALAQSSDHDMPSEDDFGSLGRIPAGMLRDGDDLAAAIADAGPIARELMASASQPSAPTLEGTDTQQPFVTIDDLNEEREKVREDLIDQCLNVLGTQDGITFELADLIHAAVAKAGEGANPRAEIGTTLVSSLISLQGDEGSPEAGKKIHAYAHLVALILQDRDFFDSTLDELKEYFGSLVSWVQLEPDQKAEDAPWVEMILLIIERVLAEDEQPIEIEWTPPPSEDPLKPLPKITEPEPVVSAESRTQLFDALEGLLPKVGKNKSLALSICRILVELTRRRDLAARLSTKQTLNKLFVMIRQLAAPLEDKLQSTFLMILRHMIEDDQTIRQIMRAEIRVAFDNHRSTRPMDTSSYTRSLYYLAIRDPQAFVDVTQELLEIARYDGNPHRGQHLSLKKSEASQNAPASPSAAKATRSVPEGQKATESKVPSAEVSDGVVQFLLRELFIWKDVSDPSPGKDTLPENSADEDRTADVEMADVSPAIANFTATVQKPTDAKNERPAFKPEEHPTYLYRCFLLQCVAELVACYNSSKLEFINFKHKTNTQPATPSKPRSGTLNYLLNSLLPVGTLEHRDDVAHRKKAATSNWAINVLVSLCTKTQERQSSSVPSFDSVDFAEQEPELTQVRKFVLEHALRAFKEATASSEPLDHRYSRLLSLGELFNRMLNGKVDLQRSNVVAAGSFSAHQQIGRLMYEKNFISALTSAIAELDLNFPNAKRAVKYILGPLKQLTELGVTLSQSSDISSNVTGGSTGEDDTLSTTSVSDEDEGREQTPDLLRNTTLGEDDDESDDEDDDDDEGMEYDEYDEEMEYEEEEMPEHGDVVSDEDEDGLDGMGEIEGRPGDMEIDLDVMEEDDEDDDDSDDSEESSEDDDDDMDPGDEMDYSHMEEVTGDDENASLGEQDAGDWEEEGLEFEHHDIAVDGGSPHGGPLEHIAHVLAGDDVSESGEHDHIVRIDMGDGEEEFFEDEMPPEDDDDDEEEEPDYGEDIVYEPEIEDDEDEEADGGWAWDAQPAPAVLRGVPHHHHHHAHLHRGGFNEMLGMINDPLRPMGMRSHRSTPHARDEDGINPLLQRDNGQQRERDAFDPLSGRPTARRTFLPRVGPEAILQDLVATVGPNVGGQPFVLDPGAWQGIGALAPGFTVHTRGGGHAMLDIDTTRSLREQLGGSADGRRWAEMLSGGRAIDRGSGGHEEAQAIEFRACSTANRWQEEARLLFPGKFHEKALRVINMLLRALVPAAMEARRIREKEEAERRAAAEKAEEEARKKAEAEKAEKEAQEKQEREEREARERAEQEAQAHQQAEQAASTGDATVSMEGVEHAGDEATNEPAPVAPEATQPGERVTITIRGRELDITTLGIDREYIEALPEDMREEVIMTQFAEQRSQAVQSGEPQSEISREFLEALPREIQQELLRSEQIERRRRERDEERQRRAQEGGQASAAPAQPEEMNNADFMAMLDPNLRQAVLLDADETVLAALPDDLQAEARALLGDRRPRGDATGRLGRAMEGAARILNGPGSNGRADAAAREPSRPRRPVAQMLDKAGIATLLRLMFVSLNHKAKSNLHGILADVCKNTQNRAEVISILLSILQDGTADVNAVERSFAHLSLRAKQPAGPKTPQPLKRSLTGPQTTTPSTDLSPLNIVQQCLGTLNALANENPRVPSFFLNEHETITSQRVTPAKKGKDRESKAGRFPLNALLTLLDRKLITENTAVMETLASLLSHVTHPLTILLKRAKEAQDSAPEEATNTTAPADQSGSTTNATDVPMQEASAAGDTSVPSEEPPKESTEAKKKHRDLVPPEVPEENIGLVVNILAARECPTKTFSDTLDIIKNLSSIPGAKEVFGKELIRQAQELGDGLLTDLEHLAKQIRAAENGTDLQGMALASFSSAGAKQKKLLRVLVALDHLFDPKRIPQATAASDAGSEQKLKDDILSTLYRSPTFEKLWTSLSACLAAIRQRGNMVNVATILLPLIESLMVMCRNSALKENAANASSSVDVDVSTPPPDTRVESLFFSFTEEHRKILNELIRNNPKLMNGNLAILAKNSKVLEFDNKRAYFSRKLHDRRAEVRVSHPSLQLNVRRDQVFHDSFKSLYYKSPNEIKYGKLNIRFHGEEGIDAGGVSREWFAAMARQMFNPDYCLFNPVAADRTTFHPNTLSEVNPEHLLFFKFIGRIIGKALYENRVLDCHFSRAVYRKILGKSVSLKDMETLDLDYYKSLVWILENDITDITFETFSVDIDRFGSTETVDLVENGREIPVTEENKHEYVRHVVEYRLIRSVQAQLDEFLTGFHDIIPPELISIFNEQELELLISGLPDIDVDDWKNNTEYTNYTPTSPQVQWFWRAVRSFDKEEKAKLLQFVTGTSKVPLNGFKELEGMNGFSRFNIHRDYSSKEKLPSSHTCFNQLDLPEYDSYEHLRHQLYTAITAGSEYFGFA |
| 562 | Unigene0013545 | 17 | 43978 | 2.1 | MAEAPIVLDGGTGFLKAGYAGQNFPDHQYPSIVGRPILRTEEQAPGDIQLKDVMCGDEAAAARSMLQITYPMENGIVKRWEDMQHLWDYTFFEKMQLDPTGRKILLTEPPMNPLKNREQMCEVMFERYNFGGVYVAIQAVLALYAQGLSSGVVVDSGDGVTHIVPVYESTVLNHLTRRLDVAGRDVTRNLISLLLRRGYALNRTADFETVRAIKEKLCYVSYDLALDQRLSEDTTVLVESYTLPDGRVIRVGSERFEAPECLFQPHLVDVEQPGIAEFLFNTIQSADVDIRSSLYKAIVLSGGSSMYPGLPSRMEKEIKQLWLTKVLGGNPERLSKFKVRIEDPPRRRHMVFLGGAVLANIMADKENMWVSKQEWEEQGSAALKKLGER |
| 563 | Unigene0002831 | 17 | 17558 | 4.5 | IVTCIALIFPGSFTTIDNFAASHLNNFFSSTGGKTSSGDKTFANGGKTFTDNQFFTNHKSSTNGKTSTNDHTFTNNGKTRTCSGSDFKNFRIKGEGCEKICAPENMKHFEDLGRMGRKMGRTASKTHECWGVGAEMDDEFKEHMGEFGETMRKAFEG |
| 563 | Unigene0002830 | 17 | 17558 | 4.5 | IVTCIALIFPGSFTTIDNFAASHLNNFFSSTGGKTSSGDKTFANGGKTFTDNQFFTNHKSSTNGKTSTNDHTFTNNGKTRTCSGSDFKNFRIKGEGCEKICAPENMKHFEDLGRMGRKMGRTASKTHECWGVGAEMDDEFKEHMGEFGETMRKAFEG |
| 564 | Unigene0010635 | 17 | 17712 | 7.4 | MADNDAQHEHTFESADAGSSATFPMQCSALRKNGHVVIKNRPCKIVDMSTSKTGKHGHAKVHLVAIDIFTGKKLEDLSPSTHNMDVPNVSRREFQLLDVTDDGFLSLMSDDGSTKDDVKVPDGEAGEKINKLFNEEGKDVNVIILTAMGEEACIDAKEAPKG |
| 565 | Unigene0007242 | 17 | 124194 | 0.6 | EDPSLAFRPASIAPAEASSQQSDAAPQRGGRGGGRGRGRGRGGRGGVQGSDKRSTVNGRTFGGQLTRTETDQSDASNAASLQGAAPVFVPGQQHVQNVTQKQPIERPHRQPKQRRMSKSQAPDIATRTHEDIDNRHYECAICTSEVLRNSKVWSCHTCWTVFHLKCIKQWSSNVGASAAQQNSDGVPQMRQWRCPGCNLPKDDLPHSYTCWCEKEADPRPLQGIPPHSCGQTCGKERVRKCPHPCQLTCHAGPCPPCTHMGPTQQCYCGTHESTKRCLETNYEAGWSCGEVCGSTLSCGEHKCERPCHAGSCGPCEVRLPARCYCGRMEKDVLCNDRGVPIQSNRKHVLDASLAPGPANEDVPSDTSESQSILESWTGVFDCGSICGRAFDCGTHQCEKRCHPQDGEAGHCPRSPDVVTHCSCGKTPLHELSDAPRTSCQDDIPSCSKPCDKVLSCGHLCPQICHVGSCGSCLKMVSINCRCGRTSHSTVCHQGRDEPPQCMRVCRITLNCGRHMCDEHCCTGERKAHERQASRKKGRPLGSAPRTAEEGYEAEHICTRQCGRLLKCGNHVCEELCHKGPCGSCREAIFEEVACNCERTVLQPPLPCGTQPPPCRYPCERPKACGHPQVAHNCHQDDDTCPKCPFLTPKMCMCGKKALKNQQCWLRDVSCGTVCGQKLKCGVHFCRKQCHRKGQCEDAGGQACQQPCGKEKKVCGHPDEAPCHAPFACKESKPCNSKIFITCECQAQKQEVKCGASTSGEGNTLKSLPCNEECARLERNRRLAVALNIDQTTHVEGEDHIPYSTETLNLFAAHPKWCQTQEREFRVFATSPDEKRLRFKPMQAQQRSFVHSLAEDFGLDSESVDPEPHRHVMIWKTPRFVSAPNKTLADALRLRQQAQRSANASTNASDNEGAPAKPTARSLEPFNSFRITNARFGLTIDEIRAEINAVLHSAYPFTFDVEFLPSEDIVLKAITRTLPPQDLERMLFNLKEPLGTRIANRGFGSMQLCTTDTSLNITRFESDGAPGDGWSRVAAKKSAPKMALASSTFGSTNAFSALSGNKVTFAKKAPTLKAKAPAAVAPVIVDDW |
| 566 | Unigene0010423 | 17 | 20942 | 9 | MLTYLLLTLAAAAVAFAAPQSPAAYQPLSKPISNGEFGLTMRIIDEVGEPRAVALVATKNGTNEELVLVGQPLASNSGTPAYTTNTNAEDANGFDFVALNVDVDGTSYGLFAADVGSVYGIQTTVIAVKGMQQEEWLVSDEDDGVYRKLAAANNQFMTCAGTVNGQPAVVLSWGINKSNGISPDGCDSTTVKKNCNVPGS |
| 567 | Unigene0005576 | 16 | 25792 | 5.4 | RFVSHHDVTIGVEFGSRIVPVGPPASLEYNINNPASAPKPATTPANNAKSKGKPQEPVQKHMKLSLWDTAGQETYKSITRSYFRGASGALLVFDITRRGTFASVTSWLHDLRQIAEDDIIVVLVGNKSDLAASSTVSGDSSAPNKRQVTKEEAEEWCRENKVMQYVETSAKSGENVERAFLEVAERIYQNIEAGKYDLNDRRSGVKGPGAGGGSAGRGVQLARSDNNAAKKAGIAGGCC |
| 567 | Unigene0005575 | 16 | 29266 | 4.8 | MAQTVGYDYISKIVSLGDSGSGKSSLTIRLCEGRFVSHHDVTIGVEFGSRIVPVGPPASLEYNINNPASAPKPATTPANNAKSKGKPQEPVQKHMKLSLWDTAGQETYKSITRSYFRGASGALLVFDITRRGTFASVTSWLHDLRQIAEDDIIVVLVGNKSDLAASSTVSGDSSAPNKRQVTKEEAEEWCRENKVMQYVETSAKSGENVERAFLEVAERIYQNIEAGKYDLNDRRSGVKGPGAGGGSAGRGVQLARSDNNAAKKAGIAGGCC |
| 568 | Unigene0005190 | 15 | 211977 | 0.3 | RKKFSSPFLEAAHKKQVRLCAAFGGQGPDNGTCLESIRRLRAHGDAAVSTLLKLAHTTLGDLGDEEHPSRFHDSRGFSLEAWLDDADAVPDNDYIALAPVSFPLITLISLIEYTLTCRALGVHPGQLRELLTNVTGHSQGMFAAAAVAVARDWPSFFDACEAILCLSFWVGLESHHVAPKSKLPAASASDCIAHGEGQPSYMLSVTGLPRPQLEAVIRRVNSNNTDSGDIHLALINSSKHRFAIAGKPSALRTMCMHLRQIKAPEGLDQRRLPFHKRKPIVQVQFMPISAPFHTGFLERVDQLVLQRLDGALALGSAEIPLLNPSNGHHITDLASPAELSQLIRSVTVDRVDWATTCLALPVSHLLAFGPGRSASLINETTDGTGLQVVQVTDLLPALRDSDSNGDIFADLRCSSLQSWRQEFAPEVVIDQHGCKNLVTRMSLLLGAPPIMVAGMTPTTVHPDFCAAIMDAGYHVELAGGGYSSESKFETALRELSAKTPGRRGITCNVLYISPKTIAWQISAIRRLIREGIRIDGITIGAGIPSPEVIKEYIETIGLKHISFKPGTEEAIREVVDIARQNPTFNIGLQWTGGRAGGHHSLEDFHDPILKTYSLIRSCQNIVLIAGSGLGEASEINPYFTGEWASEFGRPAMPFDGVLLGSRVMVAREARTSAAAKRLIVDAEGVEDAHWHKAADANTGGVITITSEMGQPIHMLATRGVMLWKELDETLFSIKDAKQRIDYLQSHRERIISRLNQDFQKPWFAIDATGRAVEINDLTYAETLSRFCKLTHVEHQNRWIDPSYHSLAKEVVHMAAERFGKVIDITHCDHPRDLLSSIEVELNEGANETLYPVDARKLLALFRRRGQKPMPFVPKLDENFETWFKKDSLWQAEDIDAVLDQDVQRVCIIHGPVAARHSKNDGESVKDILDNIYRPLVEDLSHEERLARPVDSQNDDAVHIALPPKAVSEVPGLKISTAGQKTTYELLKTGKLPEPSAMLQLLSESVGVEWFTACLLDETVVRGSAAMPNPIRQALQLQAGDIVDVIRCAQSRDIASVSFLSPSGIPKRLHRALTVEALGQGRLGVSVSLDADTAIRIPFEYRVGVPETRLSLVTDWYLRTVQDMYAELWGDGSEIRTTAGLGSEFSCKEVTVQHDEVQNYVRVIQQSCPPQLLDWQPGHSSSVPLDYCIVIGWSALTKPLLLTELSCDLLKLVHQSVNFRYVDGASPIEVGDVLQTTSRITKLSSRSNGTQVEISATIHRSSEAIVDLTCEFFIQGKATEHAKQFTSIREPDMVVDIHSPVVAALITSRKWLIREHLGTSSGLIGRRLVFQLTTHTTTSPCSNAASGSLQAHGLVSLCNGDGSLEPFGRVYFECEDNSFNPVKDFLQRYGTLKDERHDLENPGWEGGPSSLVMRAPLRSSSYSAVSRDGNPIHTSPFLARFAGLPGTIVHGMHLSAMARRATEWLIGDHEQSRFHSWTASFEATVRPGDMLRTEFQHVAMENGKMVLEVNIYNNVTGERVMRSTAVIEQARTAYVFCGQGSQEKGMGMSLYESSPAARALFNRGEAYLREHYGFSILKIIRENPTGLTISFGGKRGRRIRQNYLTMGQRDTSSLPYSCVVPSLTPQSASYTFHYPKGLLYSTQFSQPALALMEMAQYEHLRSQGILQQTSLFAGHSLGEYAALGSCSTVLPFERLMTLVFYRGLKMQNALQRDIEGKTDYSMVAVDPSRISKDFSEATFRHLVEAIAAQSGLLLEVVNFNIQDKQYVCAGHFRTLYILSKICDELSIIRDAAAFITDPTKFSALVRNHIEASSILTNSSVLERGKATIPLSGVDLPFHSTMLRGHIEDYREYLDHVLKVGDIKVEELVGRWIPNVVGKPFAVSKDYVEEVAKITNSDRLGHLVSVMA |
| 569 | Unigene0012451 | 15 | 40705 | 5.2 | VDPENKHKVEDYFHVEFTPDAPGSCHPWETKVREAWEETIQMLRFATGKAKMLRSSKPAPAMAERRDEWIRAEQTFRALFGKPDAKPEDFEGAEFNDKPMAEVIEDQIGSILFDHLQLPQKPYLRCDQDYFLYVKPDHVDPRDAGTPEEGKHTFVKPSKNNVPCPNGAWILQGFPKQQTFWQCYPDPVPRVEDRAMDDVCSDDPMMGKSLAKSIWFEYTPSTNRWNWDSDRRIVTLCPALLNRADWSASATSSSIKEGDRLGYPIAVTWVHEMFHYVSGWFDERAITAEGEPWEERRTYGWEKAVNLGQHAAQRTIFSPENMDLFAVAMYFKEWHWGSGRAKKCPEED |
| 570 | Unigene0009901 | 15 | 42084 | 2.1 | PTSLTEVFGLGNNAYFSNGKIDRLAFLRAQYDLLDKASRHPSAKYLTFNELNALRDSDGNFAYVGYEDVKDLMGEPFAKDEVAQIQSFDPAEYHPRLVFLGLDLEAGCKTPAVTLGMYHGVPYFALDVSSSYYDNFRNRQTEQGKTHVPTRVDLKLSRDDSSILSHARSLLDWNTRNRFCGACGGKTLSTHGGSKIVCPPADAGVSRRACPTRTGLHNQAFPRTDPTVVIAPISADAKRILLGRGKRWPENYFSCLSGFVEPGESLEVATRREAFEETGVRLGHVQIHSSQPWPYPSTLLIGAMGQCIEGGEDITYPESELEEAKWFELDEIEHALNNGANAMWEPPIKGYVGPRVPPAQLMAHQTLRGVLKLFKK |
| 571 | Unigene0009648 | 15 | 267623 | 0.7 | LVPFLASSRSRRGSLASISSKKGVDKETLAQALDEIHTTASRSDGLTSFHDFDGEGKTGGAKELVSNGITGMYNRLRQTVAGGSSSGAKDTGKSVPKSIESVDAGSTLSASSSLQTTRSRTKEVNGKYTVSDQLPTANKSTSPSPQMRTVPSKDVSEGKPAAPQLHVPTADAQPVPVAGPRDQRETAVDATKLPPVQAKSKPDGEAPTKEDVEKARNAWANGDRSAEGTDALARVLSHHETSLTNTTSKQISSAQLRDDLEEAIDDSDYVDESEALFPPTSQALPTDAARKAELPSVESQKPQRPPLPRVGVSHLPGFDPSRTSSTMDGSEGSSAPSSRNESHHKPRLEPAASLNAGLSRKRSTLKPTSSSSQSQSLHPKRRVLDKRFWMQDEGAKVCFSCGQSFSTFRRKHHCRTCGQIFDAKCTSLHPGRQFGQVSDVRLCKPCESMILGSDDDSTVFTDEDRDDVTRSPLGQTHNTHLNETDGGFDESTFSRTDTEVMTPSIGIPVSRRNREAKRRSAILEFDDSRTLARPSSSHSLISLSRRPRSSSHRRRHSKHHPSSRLARSSVDIERGPFHQGSTEDAEKRQLPTFHKDNIIDPDLADFLSDEGSDDGQHQSLMTALEGPSPSPGERDRLGFTGLFTSAIRKGRSRGTDRRTAPGSSIQGLKGDGVKAGLPRPRSRHLSDGSAVHASPRRRQSNHFASADAAQTKARPSTPSGQTTHAKVTRSPALQQNRPTDDLELYPASDEHARKLLAQLLKDARVAHVHAWQQALLPVLMQCTNDVEPDVQAGDDMDIRHYIKLKKIPGGRPKDTGYVSGIVFSKNIALKSMSRTLTDPRILIVNFSIEYARHEAHFMSLEPILAQETEYLKNLVKRIAQLKPDVLLVQKHVSGKALLMLEQEGITVAYNIKESVLAAIARVTMTAPVKSVDKLTMMNPDSLGRCDSFDVKTYLTDSVRKTYIFLSGCQPDLGCTVILRGADTKTLRKIKRIAEFMCYVAYNLKLENSLMRDEFVSIPRTVDSQNGTHDHPSTSTVASEDRQSTINETLQHEIEKGNAKLHFKYEEQEQESRRRVLSASPFVVFMQPYLLTQLRDLERRLATYKILRDQYAAADEDEDEPNDEKADRDIIENFSLVKPDMVNAPASKDQPKAVREYLHAVHEAQLQKAEQTFQVQERQWDTFQNGEVDPFDPFSHQKIVVLYSTVSSITSAPCTGPELLGLGFYASYSRTDPDYDDDLTLGQYVQEMCHEASKPCTECGKPMSDHHRQYVHGYGQLTISTQRQMAKVRGMHDTILMWSTCRLCRHETTVTQMSSHTWKYSFAKYLELSFWSSHLHPRAGLCKHDIHRDFLRCFGFLDTVVRVQYDPIDIYDVIVPKAQVTWKVEADLTVKNEQYLHCEERLQAFTDSVRQRLNTITVDNLNEKTAEAASALVDELRSRLEADEQELKEKLQLKYAKSRYYELIPLNRALRFMDEKAIAWDDEFARFEERYFPSEADIRKIAADQMKKMFLESQPIRNMIGSETGSDTEDGIDVSRKGKSQIKDEFLSEKAQHVLSSAMHEHRAAADEDEGLRHLVNNEHGLGPELTRSPTPRQEQEEAVEREDVKHLDLAVPSQSPPPVLVGDTLDELSPRRSPETHLDQAQQQDEPFATQPKPLSSGLLERIEQIRNNKVAAGTDEIPETKIPRLADLQQKKRDASPSPSPKPVPPPFLRAKSQPGHPAHAHRLSADAAINTQSIDAVATAQEQLSNSDALAAEKRLNERLGVARLANKAGKIVPSLIPRSVPIKQEENGPTTVSALARHFEQMSREFEKERLKERRQRAMRSRQARANPLASSRPVVEIYQNAVDAVGERPPSLEDDGELSQAHQRAARQEMDEIVAQNQPEDVAGAELDEHGNRSDDQAEYHEGEAPSMSGETAVDGDDEASDNENIRSRSRDVSDPSSSLQSPSTSGPDLHDLGPELQIGEQRKTQWFKYLSEFWSKRSASGWTNLEYPLHATEHVFEDSDIIVREDEPSSVIALSLACADYRAKEKGFRNAPNRQPLSKHGHAHTASTASTANLSREEGQRADIEASLLGDTATHMKYSFAHGQVKASCKIFYAESFDALRRRCGVADRFMESMSRCLKFDSKGGKTKSLFLRTLDSRFIIKSLQEVELKAFTKFAPDYFNFMSYTLFHGVPSVIAKMFGLFQVNIRNPATGVDFSYYLLVMENLFYERNPNRRFDLKGSMRNRKIESTGQADEVLLDENLVETIFESPLFVREHSRKLLQASVFNDTLWLCKQNVMDYSLMAGFDDIRKELVVGIIDCIRTYTWDKKLESWIKDRGKNKPTITSPKDYRNRFRVSMMQYVLQAPNCWWSFQANLGTPKTLREGEGQGAAVEGDVT |
| 572 | Unigene0010826 | 15 | 65152 | 1.4 | DPVATPKDTGAHHGDNDKDEPMYTSTTTSVRHNKNGATSSVYSGNKIRHLKKEDGIPLWRKDIQYDFLKLVFDDKTPCFTKYSDGSKGHTFADIYIDAMAKSSKCSKILKEKLLQDQAGAISMAMVCLLVNVGRMNTTLNFFPEMRAQLRTYHSIPALQADQDPNAYKQLQDAPRLKSILKGATEDEPQPSTMDEIKNASIPRTNPVNLIFVMSQYAPKISEVHFPPPRDFFDLVMRPTLSSESRAKAFLWLVWWYLESDFSIEATRTNPFGQGTYGEGEEETDALPLKVPTLIGLTDEQAEQENVDTESEKHFGEVKRKERIAILASEPSPAMTALKRARKEKGLTTGHNPAPSDDEGSDAGFHRMAGATSVGRSALHHETASDYTRSPSPAAHGFQAVNKPLGGMGIGNLLNSDDVGPDPSPSHPPAAVPVKKGPGRGNWRRNKAGKDANATAAGAASGQHVPLLPNTGGQVQFVNNGPNGPVQAHGGSFAYANSSKAGMSPGGQTLSFTNPSTHIPTPSYQAQKRHRGITQHQSAVINHRKMQIDYTLDRRIRKAHERARDKREAESPIIRAWKRLKYLPHDYDSEEE |
| 573 | Unigene0014891 | 15 | 125667 | 0.8 | MEADWTETIRLSYPGALASARSSRVTAFCFDPTEELFWVGNDAGIITSFYGPDLQRYTSYRAHSSNASRTAPGSAPVKQILFCSRGVVSVSSKSVHLSSRRGIAQWHISLPEMVDLRCMTFANRDATDLIVAGCQTQLFRIDIDKGIVTETIAPATSIPFTSMRTASQTICAAAHDGTIHLLDPKSLAVIHSWKAYAGTVNDMDARGDYLLTCGWAQSQHQGLALERLVRVFDLKTRKPARPLPFQGGAANVRLHPKLSSTCIVLSQNGALHSIDIQNPEMPSMKFASVQDGHFVGLEIMPSGKGFALADSNNNLVLWGSPSKLQFTEYAKATDFPDSIIGNKQVDWSTGPINLIGMPHYREPLLSSWPNNLVHQVGVQTPRLDNLDASFRNCDFGKVGNNPRTRLRHQATEPAQQMLPDPLSAPKFLSEKSRQENTGPDTHRRMSEDLLNALDKMKVDGKASKDARFLYRNVEIKYSRFGVEDFDFRYFNKTTYAGLETHIANSYANPLLQLLRFTVTARNVSLHHTARDCRLETCLMCELGFLVDMLEKATAPNCQATNFLRALSKQPNALAQNIIETQATSVSLTTMMQNLTRFLFLRLEDNFKQVAPSLDQFHLAFGTIGLESTQCAQCHYESRVDKVWYAHDLVYPSRPAKARSTRQYFSQILKASIERHSQHRGWCLRCNSYKNMVSHRAVHCLPAVLTLNAAITTGDSRQLWATPDILPREIGVIVNNGRFFCYEGEDLQLHLQRAQYNITVYELVGVVADVLPSENEKSHLVATIDVGLASTDTTRERDWHLFNDFLVHPVSQEDALHFNPQWKLPSVITYQAKTMSHVVDQTWKTAIDTSILFRSPAQPGIGESYHFRALSADEALPTTGIHVAIDAEFVRLLREEIDVGPTGKRTMTRPARSGLARVSVLRADGHDRELPFIDDYIAIEDPIDDYLTQFSGLHDGDLTPGRSRYKLSVLKDVYKKLWILVNLGCSFIGHGLSSDLRIINIHVPEAQLVDTQELFSLGSRTQRKLSLRFLAWAVLHEDIQQDVHDSIEDARTALRLWLKYLEFSDAGILEIWKDKIMAAGRVNNFRPPAAENPSAPSTPSRKPVHAVLPGSALGTP |
| 574 | Unigene0007471 | 14 | 31660 | 4.8 | ETGCCFHATADGGPGGVVEQLFDGQNRIGQTGLKEGEYCINTQGGLVDSSGRGCILTPPTSQWQCDAGAQPSKGFYVGADGSLGYNSSTQFWTCPTGDNAGWNIYGNQPIPNEPKCVATKLFADKCKASEPPADKPAPPPSSQCPGYLPKAYEFPHLIVPVNEGERDKCYGTSYNGTAGRGLSSIFNFDIPISAKGKTCDIRFLFPTQRQLETSAYTFADSSDGFQFALLSGVATEQSTFANLPRVKQDLGQQALAPGAVFDIASMACAAGERISVWLQAKGNSYIDFFQE |
| 574 | Unigene0007470 | 14 | 21796 | 7 | ETGCCFHATADGGPGGVVEQLFDGQNRIGQTGLKEGEYCINTQGGLVDSSGRGCILTPPTSQWQCDAGAQPSKGFYVGADGSLGYNSSTQFWTCPTGDNAGWNIYGNQPIPNEPKCVATKLFADKCKASEPPADKPAPPPSSQCPGYLPKAYEFPHLIVPVNEGERDKCYGTSYNGTAGRGLSSIFNFDIPISAKGKTCEL |
| 575 | Unigene0005207 | 14 | 91597 | 2.2 | EDDPQDVSMSDGEVIRSTETHGSLLAQSGGDFTGKQAIGSFHADELLLRPPHADTLLKGKGIRRGSNAFDHQLFARKDGPKPAKFEQLPYGTLQTGLVYDVRMRFHVEAEPSEDDLHPEDPRRIHAIFEAFVNAGLAWRDGDSGPANDYYMGRIDARMVTRDEVCLVHTRNHWNWVQSLSVMSSADLKDERQHPPHMNDSIYLSNSTPYCAALSAGGAIEACRAIILGKVKNVFAVIRPPGHHAEREDAKGFCFYDNVSIATKACQKEFGDQCRKVLILDWDVHHGNGIQQANYYDPNVLYISLHVHKRGNFYPEHSYRDNRVAYGDHLHCGEGAGLGKNVNIPWSRQGMGDADYLYAFQQVVMPIATEFNPDLVIIAAGFDAAEGDMLGGCKVTPAGYAHMTHMLMSLADGKIAVCLEGGYNLESIARSATAVARTLMGEPPDRLENTVATISGIDDVKLVARQQSRFWTCLYPKDMSHRLKGPLRCERMHNVVRGWQAKTMWDEYEMTPLFVHHEQLAKEFEDQVLATPNYSTAQALFVVLHDPPEVLASPDPRTGKIELHNTWLTYVDTAYKEGLAVVDVNLPKYVTDYDEDSQEHQPNESTDYRIKEASQLLKYLWDNYVELSECTHVYLMGTNTGHGAIINLLKNNQETFLKKYNDREEDNKLLKVISFVEDVPLMSCKSLVNGDEELAHWYHRNSLVLVGSEHAYFASDFARKPKRRFGQVVKSDSNTITEMLLQHKDAVFEILLEDAEEWRSAQHENGADEMDAVPSPLASPRKLPSVGLSPTSTAAMPVFSRPVLENGNGSPR |
| 575 | Unigene0005208 | 14 | 92153 | 2.2 | EDDPQDVSMSDGEVIRSTETHGSLLAQSGGDFTGKQAIGSFHADELLLRPPHADTLLKGKGIRRGSNAFDHQLFARKDGPKPAKFEQLPYGTLQTGLVYDVRMRFHVEAEPSEDDLHPEDPRRIHAIFEAFVNAGLAWRDGDSGPANDYYMGRIDARMVTRDEVCLVHTRNHWNWVQSLSVMSSADLKDERQHPPHMNDSIYLSNSTPYCAALSAGGAIEACRAIILGKVKNVFAVIRPPGHHAEREDAKGFCFYDNVSIATKACQKEFGDQCRKVLILDWDVHHGNGIQQANYYDPNVLYISLHVHKRGNFYPEHSYRDNRVAYGDHLHCGEGAGLGKNVNIPWSRQGMGDADYLYAFQQVVMPIATEFNPDLVIIAAGFDAAEGDMLGGCKVTPAGYAHMTHMLMSLADGKIAVCLEGGYNLESIARSATAVARTLMGEPPDRLENTVATISGIDDVKLVARQQSRFWTCLYPKDMSHRLKGPLRCERMHNVVRGWQAKTMWDEYEMTPLFVHHEQLAKEFEDQVLATPNYSTAQALFVVLHDPPEVLASPDPRTGKIELHNTWLTDIVKTYVDTAYKEGLAVVDVNLPKYVTDYDEDSQEHQPNESTDYRIKEASQLLKYLWDNYVELSECTHVYLMGTNTGHGAIINLLKNNQETFLKKYNDREEDNKLLKVISFVEDVPLMSCKSLVNGDEELAHWYHRNSLVLVGSEHAYFASDFARKPKRRFGQVVKSDSNTITEMLLQHKDAVFEILLEDAEEWRSAQHENGADEMDAVPSPLASPRKLPSVGLSPTSTAAMPVFSRPVLENGNGSPR |
| 576 | Unigene0005422 | 13 | 182331 | 0.7 | QFPVAAQSTLKSVDGRRVSARHSNVGTPGLSGSEAERAETTHSRGRSQSTTAASRPPSPPWKRFEAQGPTSIYIDGQRKSGRANKDLVAVPKRISPRSKKQVDRLGVQKHADIKPSASKSMGGSTRKSEPGSEKLKKQANGLERPSSSTDSAQRIADLQAQIAALQPSRSFAQDPTSPTTNGVKRNPGRPKSKIKREASPALRRKSHRTGDASPDSPTLAKPSPRLKLKVAPRVFIPPPFPQARVPSPILPPKLSIWQLLDNLELQERQAPSAENDRGPLSVEKLAEDEAKYVSREAATRRRILEATQTGGPLSVENLSLFQDDQQLEPPQQYGHRDHLTAHALYLRTLQIREKTLHRTLAKKIAQEALEKWKERNGPTEEDLIAERNKIIDHVKKQIVTDMKAKWEMVEAHVKELKRRAWEKEQERVRAEKLKEKLEYSENLLAKQRGEADSDSGMDEDSVSDAEESGQESEEDSEENMDSSDESEEEGAMSEEAFAAYKAKRDAELEAERQASRGVEPPDKLDSKVEPPDNEDRNAETNATSPTREQKDSNDEERDNGDATPEAEGRGDAMDIDDAAPSSRPKKSGLAALFQDDDDADDDETSDESVDMDSEDYDSDEDMSSTGDEADNNDNDEEENGTDDTEQPPELRNSLMALYSEKELLKQKEGGLPTPMTSVENGGDDENARPQSEPRSEAQADAGASTDEKPIATGIDEQPGVGDSEDKPVPPLQTNGGGDAMETEQIGEVDGASETSEPGFTKTLVPVPNLLRGTLRSYQHAGVDWLASLYRNGTNGILADEMGLGKTIQTIALLAHLAEVHEVWETHLVIVPTSVILNWVTEFQKFLPGFRVLGYYGSASERELKRKGWTNDPHHEDRSKRGYNVVITSYNVAAQDINAIRGQQWHYLILDEAHNIRNFNSQRWQLLIRLRTKARLLLTGTPLQNDLAEVWSLLTFLTAGDESRSHGELEEFLSHWKDPVKEIFDQGVQKLTENAQRVVDQLHISLRPFLLRRKKDEVEKDLPKKTESVVVCKLSKRQRQLYQDYMGLASTRDTLAKGSGVQAGAVLLSLRRVCNHPDLFDPRPIQTSFAMECSPLEGYAESEHLIRRMSGPRDNIPAKLLIVSNTSLRRSAMNRSRQLAVGDELSRQLSEVESTIPNEEPDRATIAGSRALQRLRMRQRRLQQLRSCIAATESALDQEPLLPTDLREVVTVSNNKPYMFKSKSQPLVKTWQGHTRLGRRPLRFEHLSDWHVANDTQLQRDIATSDSYAEDMKELIVRFAFVPPAATVPILDYAIPRKAQEAMRSSPLYPADHDYGHEARVRTSIAFPDKRLLIYDAGKLQRLTYLLRDLQSRGSRSLIFTQMTGTLDVLERFLSLMGLPYLRLDGSTPVERRQLYSSEFNRADSKYQCMILSSRAGGVGLNLTGASSVIFYDLDWNPQMDRQCMDRAHRIGQTKDVEVYKLVSEKTVEENILRRANQKSLLDQTVIQDGHFTTEYQLKRSSDDKEDEIDDAIGRLLGGDEQATTTALASVEDKEDVQAAEKAGKEDRTDDVDFGDRSSKGPSKANTPGPGAAEDEIDEVAEELKGHVDLYMIKQMEHLLQGWVYTPPPARLDKHGRDRSHRPKKRIR |
| 576 | Unigene0005423 | 13 | 190685 | 0.7 | DDPAADPTTIKSEPLDHGSDGQRPSADADAAAPPPTLPPDYAPPATKRRRESEVELHTPEPAQHSPPKKRKRAASPPWQFPVAAQSTLKSVDGRRVSARHSNVGTPGLSGSEAERAETTHSRGRSQSTTAASRPPSPPWKRFEAQGPTSIYIDGQRKSGRANKDLVAVPKRISPRSKKQVDRLGVQKHADIKPSASKSMGGSTRKSEPGSEKLKKQANGLERPSSSTDSAQRIADLQAQIAALQPSRSFAQDPTSPTTNGVKRNPGRPKSKIKREASPALRRKSHRTGDASPDSPTLAKPSPRLKLKVAPRVFIPPPFPQARVPSPILPPKLSIWQLLDNLELQERQAPSAENDRGPLSVEKLAEDEAKYVSREAATRRRILEATQTGGPLSVENLSLFQDDQQLEPPQQYGHRDHLTAHALYLRTLQIREKTLHRTLAKKIAQEALEKWKERNGPTEEDLIAERNKIIDHVKKQIVTDMKAKWEMVEAHVKELKRRAWEKEQERVRAEKLKEKLEYSENLLAKQRGEADSDSGMDEDSVSDAEESGQESEEDSEENMDSSDESEEEGAMSEEAFAAYKAKRDAELEAERQASRGVEPPDKLDSKVEPPDNEDRNAETNATSPTREQKDSNDEERDNGDATPEAEGRGDAMDIDDAAPSSRPKKSGLAALFQDDDDADDDETSDESVDMDSEDYDSDEDMSSTGDEADNNDNDEEENGTDDTEQPPELRNSLMALYSEKELLKQKEGGLPTPMTSVENGGDDENARPQSEPRSEAQADAGASTDEKPIATGIDEQPGVGDSEDKPVPPLQTNGGGDAMETEQIGEVDGASETSEPGFTKTLVPVPNLLRGTLRSYQHAGVDWLASLYRNGTNGILADEMGLGKTIQTIALLAHLAEVHEVWETHLVIVPTSVILNWVTEFQKFLPGFRVLGYYGSASERELKRKGWTNDPHHEDRSKRGYNVVITSYNVAAQDINAIRGQQWHYLILDEAHNIRNFNSQRWQLLIRLRTKARLLLTGTPLQNDLAEVWSLLTFLTAGDESRSHGELEEFLSHWKDPVKEIFDQGVQKLTENAQRVVDQLHISLRPFLLRRKKDEVEKDLPKKTESVVVCKLSKRQRQLYQDYMGLASTRDTLAKGSGVQAGAVLLSLRRVCNHPDLFDPRPIQTSFAMECSPLEGYAESEHLIRRMSGPRDNIPAKLLIVSNTSLRRSAMNRSRQLAVGDELSRQLSEVESTIPNEEPDRATIAGSRALQRLRMRQRRLQQLRSCIAATESALDQEPLLPTDLREVVTVSNNKPYMFKSKSQPLVKTWQGHTRLGRRPLRFEHLSDWHVANDTQLQRDIATSDSYAEDMKELIVRFAFVPPAATVPILDYAIPRKAQEAMRSSPLYPADHDYGHEARVRTSIAFPDKRLLIYDAGKLQRLTYLLRDLQSRGSRSLIFTQMTGTLDVLERFLSLMGLPYLRLDGSTPVERRQLYSSEFNRADSKYQCMILSSRAGGVGLNLTGASSVIFYDLDWNPQMDRQCMDRAHRIGQTKDVEVYKLVSEKTVEENILRRANQKSLLDQTVIQDGHFTTEYQLKRSSDDKEDEIDDAIGRLLGGDEQATTTALASVEDKEDVQAAEKAGKEDRTDDVDFGDRSSKGPSKANTPGPGAAEDEIDEVAEELKGHVDLYMIKQMEHLLQGWVYTPPPARLDKHGRDRSHRPKKRIR |

^a^ Protein grouping number, protein sharing peptides and quantitative information with the same grouping number

^b^ Protein accession number

^c^ Protein identification score

^d^ Protein quality

^e^ Sequence coverage of proteins

^f^ Protein sequence
